# Development and Validation of the CHDSI Questionnaire: A New Tool for Measuring Disease-Specific Quality of Life in Children and Adolescents with Congenital Heart Defects

**DOI:** 10.3390/medicina61071311

**Published:** 2025-07-21

**Authors:** Paul C. Helm, Ulrike M. M. Bauer, Peter Ewert, Julia Remmele

**Affiliations:** 1 National Register for Congenital Heart Defects, 13353 Berlin, Germany; 2 Competence Network for Congenital Heart Defects, 13353 Berlin, Germany; 3 Department of Congenital Heart Disease and Pediatric Cardiology, German Heart Center, TUM University Hospital, 80636 Munich, Germany; 4 DZHK (German Centre for Cardiovascular Research), Partner Site Munich Heart Alliance, 80802 Munich, Germany

**Keywords:** CHDSI, questionnaire, congenital heart defects (CHD), young CHD patients, disease-specific quality of life (DsQoL)

## Abstract

*Background and Objectives*: Congenital heart defects (CHD) affect around 1% of the population, making them the most common congenital disease worldwide. Thanks to advances in treatment, over 90% of affected children are able to reach adulthood, shifting focus to long-term outcomes such as disease-specific quality of life (DsQoL). To date, there has been no validated, standardized instrument for assessing DsQoL in young German CHD patients. This study introduces the Congenital Heart Disease Specific Inventory (CHDSI), the first freely available German-language instrument for measuring DsQoL in children and adolescents with CHD. *Materials and Methods*: The CHDSI was developed at the German Heart Center Munich in collaboration with affected children and adolescents and validated nationwide via the National Register for Congenital Heart Defects (NRCHD) with 1201 participants (46 kindergarten children, 530 children, 625 adolescents). Two age-specific versions (36/37 items) and a 31-item preschool version were created, alongside a 6-item short form (CHDSI-SF) for rapid screening. Reliability was assessed using Cronbach’s alpha and split-half methods; construct validity via confirmatory factor analysis (CFA) using DWLS; and score interpretation through standardized stanine scales. The small sample size of kindergarten children precluded a model test for this group. The standard values given for this subsample should therefore be interpreted with caution. *Results*: The CHDSI showed excellent internal consistency (Cronbach’s α = 0.856 to 0.900) and high split-half reliability (>0.95). CFA confirmed a robust six-factor structure with excellent model fit (CFI and TLI ≥ 0.991, RMSEA ≤ 0.05). Subscales showed strong discriminant validity, and significant differences were found by CHD severity and sex. *Conclusions*: The CHDSI is a psychometrically valid, age-appropriate, and freely available instrument for assessing DsQoL in children and adolescents with CHD. It provides valuable support for clinical decision-making and research. Further studies should explore international validation and cultural adaptation.

## 1. Introduction

Congenital heart defects (CHD) affect approximately 1% of the population, are the most common congenital defects, and contribute to increased morbidity and mortality [1,2,3]. Around 6000 children are born with CHD in Germany each year [4,5]. Due to advances in prenatal diagnostics, pediatric cardiology, and cardiac surgery, over 90% of these patients survive into adulthood [2,5,6,7,8,9,10,11,12,13,14], reducing mortality without altering prevalence [2,10,11,12]. These advances have brought new challenges, particularly neurodevelopmental impairments and reduced quality of life (QoL), shifting clinical and research priorities toward developmental outcomes and QoL-focused care.

Studies on health-related QoL (HrQoL) in children and adolescents with CHD provide mixed results: some report impairments compared to healthy peers [15,16,17], while others categorize self-reported HrQoL as comparable [15,18,19]. These discrepancies may be due to the timing and context of the assessment—for example, anxiety is higher during hospitalization than routine follow-up, influencing HrQoL scores [20]—as well as the use of different measurement tools and environmental factors. A French-Belgian study of children and adolescents with CHD (ages 8 to 18 years, N = 282) and healthy controls (N = 180) using the KIDSCREEN-52 reported significantly lower scores in physical well-being, social support, autonomy, and financial resources in the CHD group, with disease severity correlating with poorer outcomes [15]. Similarly, a population-based cohort study (N = 598 children with CHD aged 8 years) using the PedsQL found that children who had undergone cardiac interventions—particularly palliative procedures—showed significantly lower QoL, with moderate to large effect sizes [21].

The World Health Organization (WHO) defines health as comprehensive well-being—not just the absence of disease [22]—positioning HrQoL as a key component of overall QoL [23,24]. The importance of QoL and HrQoL in CHD is underscored by their association with educational attainment and workforce participation [25,26,27,28,29], factors that are critical for those with chronic illness. Just as HrQoL is a component of overall QoL, the same applies to disease-specific QoL (DsQoL). In our view, DsQoL—like HrQoL—should be understood as a subset of QoL. However, unlike HrQoL, which focuses on general health-related aspects, DsQoL addresses disease-specific issues that are relevant to a particular patient group, reflecting the unique challenges, concerns, and burdens associated with their illness. Addressing DsQoL involves understanding the unique experiences of children and adolescents with CHD and integrating these insights into treatment planning [27,28,30], as well as considering life circumstances [31,32]. Although standardized tools like the SF-36 Health Survey are widely used, they do not fully differentiate between QoL and HrQoL [33].

Comprehensive care extends beyond survival and medical management to include patient education about risks, prognosis, and life impact, as well as addressing knowledge gaps regarding daily life challenges [29]. Modern medicine increasingly emphasizes the patient’s perspective and functional well-being as well as longevity [25,26,27,28,29,30,34,35,36,37,38,39,40,41,42]. Patient-reported outcomes are often missing from medical records, highlighting the need for standardized questionnaires to capture the multidimensional concept of QoL—physical, mental, and social—and to evaluate whether treatments simply prolong life or enhance its quality [23,43,44,45,46,47,48,49,50,51].

Pediatric HrQoL can be measured using validated tools. To contextualize the development of a new German-language questionnaire for children and adolescents with CHD, two widely established instruments are briefly introduced: the Pediatric Quality of Life Inventory (PedsQL) [52] and the Pediatric Cardiac Quality of Life Inventory (PCQLI) [53].

The PedsQL 4.0 Generic Core Scales have undergone extensive psychometric validation for reliability and validity in diverse pediatric populations [52]. A heterogeneous sample (N = 1677) showed high internal consistency (α = 0.88 child, 0.90 parent). The factor analysis largely supported its four-domain model, although the school function was split into the item’s attendance and performance. Scores are scaled 0 to 100 (higher = better HrQoL) with standardized scoring algorithms enabling cross-group comparisons. Additional studies [54,55,56,57] have validated the PedsQL across U.S. populations. While a German version showed reliability in disease-specific contexts of cancer and epilepsy [58], no normative data exist for the general German pediatric CHD population. The PedsQL Cardiac Module was validated in mixed samples of patients with acquired heart disease and CHD [59,60,61], but to our knowledge, it still lacks standardized normative data for a substantial CHD-specific population.

The PCQLI was developed for children with congenital and acquired heart disease and validated in a multicenter U.S. sample of 655 patient–parent pairs [53]. It comprises two subscales—impact of the disease and psychosocial impact—explaining 55 to 60% of variance, with high internal consistency (α = 0.78 to 0.91) and strong subscale correlations (r = 0.81 to 0.96). Scores are transformed into a scale from 0 to 100. Validation in a larger U.S. cohort (N = 1605) confirmed strong correlations with PedsQL scores (r = 0.70 to 0.76) and high test–retest reliability (r = 0.78 to 0.90) [62]. However, limitations include a lack of clear CHD severity classification, overreliance on maternal reports, and absence of validation outside the U.S.

While both instruments offer valuable insights, neither the PedsQL nor the PCQLI provides standardized normative data for CHD populations in Germany or internationally. To address this gap, we developed the Congenital Heart Disease Specific Inventory (CHDSI)—the first instrument to explicitly measure DsQoL in children and adolescents with CHD. It is designed for both clinical and research use.

## 2. Materials and Methods

To highlight the relevance of our newly developed CHDSI, we start with a concise tabular comparison of the CHDSI, PedsQL, and PCQLI, which underscores their differences in measured constructs, dimensions, and disease-specific orientation (see Table 1).

The initial CHDSI was piloted and refined at the German Heart Center Munich (DHM) after developing a preliminary questionnaire for children and adolescents with CHD. Nationwide validation and standardization were then conducted via the National Register for Congenital Heart Defects (NRCHD) [63]. The study aimed to develop a tool for measuring DsQoL in children and adolescents according to age, sex, and CHD severity and to offer a validated questionnaire for research, interventions, and clinical practice. In close collaboration and exchange with children and adolescents with CHD, two final versions were created: a 36-item version for children (6 to <14 years) and a 37-item version for adolescents (14 to <18 years). In addition, a reduced version with 31 items was suggested for preschool children. Model fit was assessed using confirmatory factor analysis (CFA), and no formal hypotheses were formulated.

The paper-and-pencil version of the CHDSI is available as Supplementary Material free of charge for non-commercial use (scientific research and routine clinical practice). Commercial use requires prior written authorization from the authors. The Supplementary Materials also include an SPSS data sheet, analysis syntax for the CHDSI, and a detailed manual in German.

### 2.1. Development Steps

Developed in two phases at the DHM, the CHDSI comprises separate questionnaires for children (6 to <12 years) and adolescents (12 to <18 years). In collaboration with patients, initial surveys (seven sets of six questions on school, family, leisure, illness, treatment, recovery, and socio-demographics) with free-text fields were refined in a pretest of 80 patients. All CHDSI items were checked for clarity in pilot tests. Participant feedback during pilot testing confirmed that negatively worded items were well understood and contributed appropriately to each subscale. Revised versions tested on 73 patients produced two age-specific, 43-item questionnaires (6 to <14 and 14 to <18 years, plus a preschool subset). Each questionnaire version concludes with a free-text field for respondents to add any additional information. The CHDSI was subsequently validated on a large German sample.

### 2.2. Validation and Standardization Process

The CHDSI was validated and standardized nationwide online. Participants were recruited through the NRCHD—an organization that registers approximately 55,000 CHD patients across all severities [63]. To be eligible, participants needed a postal or email address and had to be between 6 and <18 years old. Invitations were sent to legal guardians who forwarded them to their children. Consent was obtained when participants completed the online questionnaire after receiving an invitation.

### 2.3. Patient Collective

Of 11,919 CHD patients (ages 6 to <18 years) registered in the NRCHD, 11,906 (99.9%) were contacted via legal guardians. Of these, 2083 (17.5%) participated, and 1201 (10.1%) fully completed the survey. The sex ratio was nearly balanced. Each participant received a study number and an age-appropriate CHDSI online questionnaire. Only participants who fully completed the CHDSI (N = 1201) were included in the statistical analyses, resulting in no missing data and eliminating the need for listwise deletion, imputation, or related procedures.

### 2.4. Research Infrastructure

Founded in 2003, the NRCHD represents German CHD patients and supports targeted cohort formation. The NRCHD provides representative data for clinically apparent CHD [63] from over 30 universities, clinics, and research institutes and all pediatric heart centers across Germany. Contributors include pediatric cardiologists, cardiologists, rehabilitation centers, and researchers nationwide. The NRCHD also benefits from broad collaboration with general hospitals and outpatient physicians from various specialties. The study was conducted in accordance with the Declaration of Helsinki. Ethics approval (EA2/253/21, Charité Berlin) and patient/guardian consent were secured, and the NRCHD data protection concept (No. 531.390) ensured GDPR compliance. The data collected and analyzed cannot be shared for data protection reasons. Detailed medical data are available only if patients or guardians provide reports or waive confidentiality. All NRCHD-registered patients aged 6 to under 18 years with a current email or postal address were eligible to participate, regardless of the availability of detailed medical data on their CHD. Of the 1201 CHDSI participants, 110 (9.2%) could not be clearly classified into one of the three CHD severity categories (simple, moderate, complex). CHD diagnoses were based on the International Pediatric and Congenital Cardiac Code (IPCCC) [64], and CHD severity was assigned using the Warnes et al. classification [65]. This system classifies CHD by anatomical complexity, clinical progression, and care needs: simple CHD generally has favorable outcomes with minimal follow-up; moderate cases require ongoing treatment and monitoring; and complex CHD involves lifelong, specialized care. The Warnes classification is widely used to guide clinical management, research, and resource planning [65].

### 2.5. Overview of Questions, Question Complexes, and Postulated Scales

The questionnaire starts with an introduction, 11 demographic items, a sample question, and 43 rating items organized into seven complexes (six complexes of 6 items and one of 7). Some items serve as indicators outside the scales (Table 2 lists the latent variables and corresponding items).

**Figure 1:**
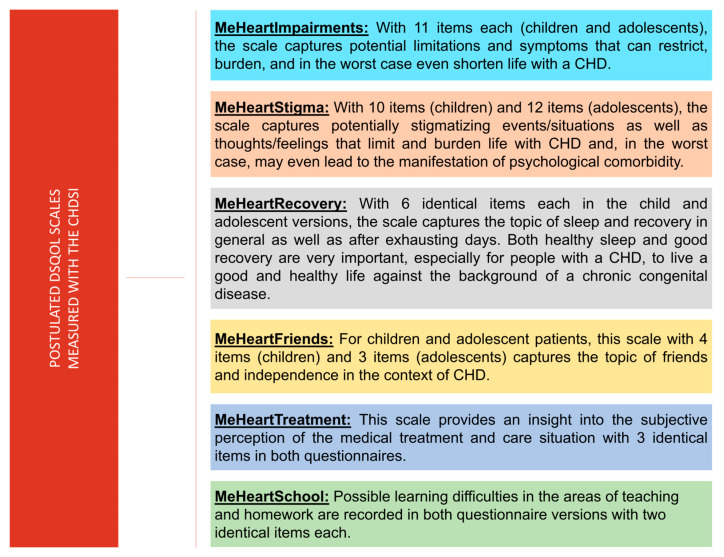
Postulated DsQoL-scales. This figure presents the postulated domains of the CHDSI questionnaire designed to assess disease-specific quality of life (DsQoL) in children and adolescents with congenital heart disease (CHD). Each colored block represents a scale: MeHeartImpairments, MeHeartStigma, MeHeartRecovery, MeHeartFriends, MeHeartTreatment, and MeHeartSchool. These scales capture physical, emotional, social, and educational burdens using age-appropriate item sets. For example, MeHeartImpairments (11 items) measures functional limitations, while MeHeartStigma (10–12 items) explores psychosocial impacts. Other scales assess sleep/recovery (MeHeartRecovery), peer relations (MeHeartFriends), healthcare experiences (MeHeartTreatment), and academic challenges (MeHeartSchool). Together, they provide a comprehensive DsQoL profile, with implications for individualized patient care and intervention.

**Figure 2:**
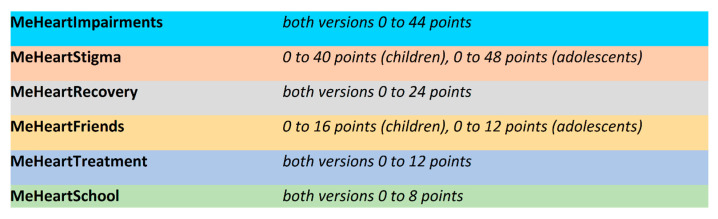
Score ranges of the constructed scales. This figure displays the score ranges for the six disease-specific quality of life (DsQoL) scales used in evaluating children and adolescents with congenital heart disease (CHD). Each scale varies in its item count and maximum score, reflecting the weight of its respective domain. For example, MeHeartImpairments has a maximum of 44 points across both age groups, while MeHeartStigma ranges from 40 (children) to 48 points (adolescents). Other domains such as MeHeartRecovery, MeHeartFriends, MeHeartTreatment, and MeHeartSchool have progressively lower point ceilings. Lower scores generally reflect greater burden or difficulty. This scoring framework supports a nuanced interpretation of CHD-related impacts.

Among the 43 items, 31 (kindergarten), 36 (school children), and 37 (adolescents) align with the DsQoL model, while 7 (children) and 6 (adolescents) do not. The constructed scales (see Figure 1) range from 2 to 12 items (see Figure 2).

The six domains of the CHDSI were developed through a rigorous mixed-methods process involving focus groups with children and adolescents with CHD, consultations with clinicians, item generation, and pilot testing. The domain names reflect the key themes mentioned by young patients themselves. These domains were mapped to established QoL frameworks, highlighting both areas of alignment and novel dimensions uniquely captured by the CHDSI. Although a separate cross-validation or holdout sampling was not used, age-specific confirmatory factor analyses (CFA) and subgroup analyses based on CHD severity were conducted, demonstrating the stability and discriminant validity of the domain structure across independent subsamples.

The CHDSI features two main versions: 36 items for school children and 37 for adolescents with CHD. Due to insufficient data for kindergarten, the model could not be tested for this group. In both versions, 36 (children) and 37 (adolescents) out of 43 items can be assigned to the six scales measuring comparable DsQoL sub-constructs (see Table 2). Preliminary analyses and feedback indicate that children and adolescents perceive DsQoL aspects differently, with four scales being identical and slight differences existing in the other two.

### 2.6. Data Analyses and Statistics

The online survey assigned participants to children and adolescent groups. Two CHDSI versions were created and administered via EFS-Survey, and the collected data were analyzed using SPSS (version 25) and RStudio (version 2022.07.0).

### 2.7. Scales and Scores

The 43 DsQoL items use a five-point scale based on six latent variables (see Figure 1 and Table 2). Although a metric interpretation is conceivable [66], the items are treated as ordinal. For each item, the corresponding responses (e.g., “strongly agree” and “does not apply at all”) are scored on a scale from 4 (very happy smiley) to 0 (very sad smiley). Constructed scale scores range from 0 to 48 (see Figure 2).

Total DsQoL scores range from 0 to 144 for children (0 to 124 for kindergarten) and 0 to 148 for adolescents. Both subscale and total scores are treated as interval scales. An individual’s percentage score is calculated by multiplying their score by 100 divided by the maximum score.

### 2.8. Statistical Methods Used

Descriptive statistics (mean, SD, median, range, percentages) summarized the sample and DsQoL data. Pearson or Spearman correlations were computed per Cohen’s criteria (±0.10 weak, ±0.30 medium, ±0.50 strong) [67]. Group differences were assessed using χ^2^, Mann–Whitney U, or *t*-tests—even with non-normal data, since *t*-tests are robust for small samples (>30) and for large samples with non-normal data [68,69,70,71,72,73,74,75]. For example, Bühner and Ziegler judge a violation of the normal distribution of the residuals from 100 cases as rather unproblematic [76]. Reliability was evaluated via split-half (odd-even split, target r > 0.80) with the Spearman–Brown correction [77,78,79,80,81]. In practice, reliability is often tested in the form of internal consistency, as a single test point is sufficient [82]. Internal consistency was measured via Cronbach’s alpha (acceptable > 0.60, good > 0.70, very good > 0.80, excellent > 0.90) [83]. The mean inter-item correlation (MIC) was maintained between 0.30 and 0.60 (ideally between 0.40 and 0.50) [79,84,85,86]. Construct validity was tested using CFA to examine the DsQoL construct and instrument structure [87,88]. As questionnaire data are ordinal rather than interval data and often not normally distributed, the DWLS method (diagonally weighted least squares) was used—effective with samples ≥ 200 [89,90,91,92,93,94,95,96,97,98,99,100]. CFA was performed in RStudio (version 2022.07.0) using lavaan with the syntax ‘*ordered* = *TRUE.*’.

### 2.9. Testing the Postulated Model Using CFA

A CFA was conducted in R using DWLS estimation and the lavaan package to test the model. A non-significant χ^2^ (*p* > 0.05) indicates acceptable fit [101,102], with the normalized χ^2^ rated as follows: <1 excellent, 1 to <2 very good, 2 to <3 good, 3 good <4 acceptable, and 4 to 5 borderline. An RMSEA (root mean square error of approximation) [103] of <0.07 is acceptable, <0.06 good, and <0.05 very good [104]. The CFI (Comparative Fit Index) and TLI (Tucker–Lewis Index), which compare the target model to independent and null models, require values of ≥0.95 for very good fit, 0.90 to 0.95 for good, and 0.80 to 0.90 for poor fit [105,106]. A good model fit requires CFI and TLI > 0.95, RMSEA < 0.06, relative/normalized χ^2^ < 3, and factor loadings > 0.50. Items with factor loadings < 0.50 must be excluded [107], and model/scale revisions are necessary if Cronbach’s alpha < 0.60, split-half reliability < 0.80 (for scales with 2 items or entire questionnaire), MIC < 0.30, CFI and TLI < 0.95, RMSEA > 0.06, or normalized χ^2^ > 3.

### 2.10. Calculation of Standard Values for Total Score and Subscales

The CHDSI, applicable only to individuals aged 6 to <18 years, yields total and subscale scores that are converted into standardized stanine values. Children score between 0 and 144 (124 for kindergarten age) and adolescents between 0 and 148, with results presented as percentages from 0% (worst DsQoL) to 100% (best DsQoL). The participants rate the items on a five-point Likert scale, and raw scores are transformed into stanine scores (1–9) using SPSS (version 25) following Tent and Stelzl [108]; stanines 4 to 6 are considered normal/average. Standardization makes it possible to compare the individual results with a representative sample [78]. It should be made clear that the data from a standardized sample do not necessarily have to follow a normal distribution. There is often no normal distribution despite large samples [109].

The 9-point stanine scale, normally distributed with a mean of 5 and SD of 2, interprets values of 2 to 3 as low, 1 as very low, 7 to 8 as high, and 9 as very high [110,111]. Based on corresponding percentile ranks (4%, 11%, 23%, 40%, 60%, 77%, 89%, 96%, 100%) [79,108], stanine levels are grouped into critical (1–3) and non-critical (4–9) ranges. A critical DsQoL score strongly suggests impaired QoL and necessitates further psychological assessment, with the overall DsQoL score being the most reliable measure and subscales providing additional insights.

### 2.11. Correlation Analyses

To examine whether patient age, number of siblings, parental occupational and educational status, as well as self-reported knowledge about their CHD were significantly associated with overall DsQoL or any of its subscales, correlation analyses were conducted. To determine a possible correlation between the DsQoL values of the young CHD patients and the parents’ occupational/educational level, response categories for maternal and paternal school education, vocational qualification, and employment status were each grouped into three categories: for school education, no diploma was scored as 0, low-level education as 1, and high-level education as 2; for vocational qualification, no qualification was scored as 0, non-academic qualification as 1, and academic qualification as 2; and for employment status, not employed was scored as 0, part-time employment as 1, and full-time employment as 2. The six individual values were then summed (range 0 to 12) and grouped into three levels of parental occupational/educational status: low (0–7), medium (8–9), and high (10–12). For a total of 855 parent pairs (34 from the preschool sample, 350 from the children’s sample, and 471 from the adolescent sample), sufficient information was available to compute these scores. Due to the small sample size, children in the preschool group were not included in the subsequent correlation analyses.

### 2.12. Adequate Sample Size

Depending on the model’s complexity, sample sizes may need to exceed 250 [105,112]. For a CFA with 36/37 items, 360/370 participants (10 per item) are recommended [113]. Comrey and Lee classify norm sample quality as follows: 100 = poor, 200 = fair, 300 = good, 500 = very good, and ≥1000 = excellent [114]. Thus, a sample size of over 500 people is required for the standardization/validation of the questionnaire.

A comparison between the invited CHD patients and those who fully completed the questionnaire is presented in Table 3. As the sample sizes for the CHD severity subgroups are below 500, we recommend using the normative values based on CHD severity only if there is a clear clinical or scientific benefit. Otherwise, the overall normative values should be used to ensure the most accurate and reliable screening results.

## 3. Results

**Figure 3:**
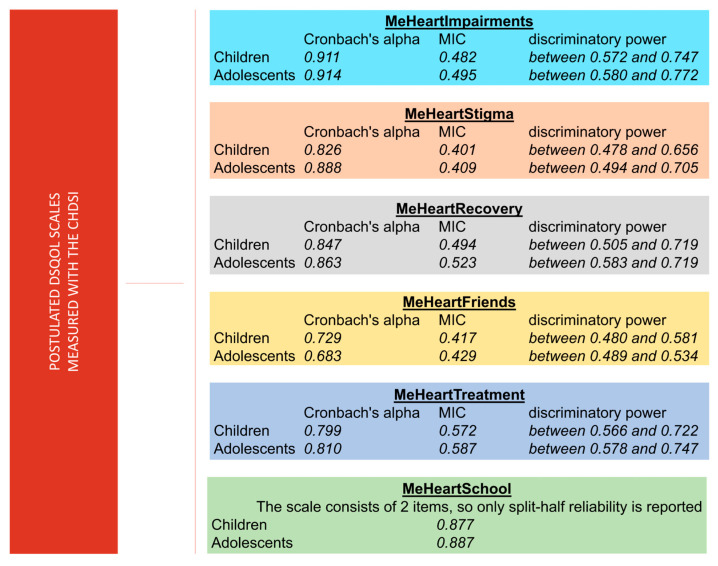
Quality criteria of the CHDSI subscales. This figure summarizes key psychometric properties—internal consistency (Cronbach’s alpha), mean inter-item correlation (MIC), and discriminatory power—of the disease-specific quality of life (DsQoL) scales used for children and adolescents with congenital heart disease (CHD). All scales demonstrate acceptable to excellent internal consistency, with Cronbach’s alpha values ranging from 0.683 (MeHeartFriends, adolescents) to 0.914 (MeHeartImpairments, adolescents). MIC values generally fall within the optimal range (0.401 to 0.587), indicating good inter-item cohesion. Discriminatory power values reflect the scales’ ability to differentiate between participants and range from 0.480 to 0.747 across scales. For MeHeartSchool, which includes only two items, split-half reliability is reported, showing strong consistency (0.877–0.887). These results support the psychometric soundness of the scales for assessing DsQoL dimensions in pediatric CHD populations.

Cronbach’s alpha for the kindergarten version (N = 46, 31 items, five scales) was α = 0.856 (16 items) and α = 0.886 (15 items). For the children’s version (N = 530, 36 items, six scales), α = 0.900 (18 items) and α = 0.885 (18 items), with split-half reliabilities of 0.956 (equal length). For the adolescent version (N = 625, 37 items, six scales), α = 0.890 (19 items) and α = 0.899 (18 items), with split-half reliability (unequal length) of 0.966. DsQoL subscales showed Cronbach’s alpha from 0.683 to 0.914, MIC values from 0.401 to 0.587, and discriminatory power from 0.478 to 0.772 (see Figure 3).

### 3.1. Mean Value Differences

In children (N = 530; 282 females; 119 simple, 194 moderate, 173 complex CHD), simple CHD patients had higher DsQoL total scores than moderate and complex CHD groups, with differences on four and five subscales, respectively. Moderate CHD patients scored higher than complex CHD patients in total score and on three subscales. In adolescents (N = 625; 327 females; 195 simple, 207 moderate, 165 complex CHD), males scored higher than females in the DsQoL total score and on four subscales. Adolescents with simple CHD performed better than those with moderate (DsQoL total score and two subscales) and complex CHD (DsQoL total score and three subscales). Moderate CHD patients scored higher than complex CHD patients in two subscales. The results of the mean comparisons are presented in detail in Table 4.

### 3.2. Model Testing

For the children’s CHDSI, CFA confirmed the six subscales despite a significant χ^2^ (1276.050, df = 579, *p* < 0.001) because the relative χ^2^ (2.2), fit indices (CFI = 0.993, TLI = 0.993, RMSEA = 0.048), and factor loadings (0.626 to 0.932) were strong. Similarly, for the adolescent version, despite a significant χ^2^ (1580.638, df = 614, *p* < 0.001) with a relative χ^2^ of 2.6, excellent fit indices (CFI = 0.992, TLI = 0.991, RMSEA = 0.05) and factor loadings (0.614 to 0.939) confirmed a good model fit. A detailed overview can be found in Table 5.

### 3.3. Standard Values for Total Score and Subscales

The CHDSI was standardized online in 2022 with a German sample of 1201 participants (52% female): 576 children (6 to <13 years, mean = 10.14 ± 2.41) and 625 adolescents (14 to <18 years, mean = 15.17 ± 1.07). For children, total DsQoL scores ranged from 18.06% (26 points out of 144) to 100% (144/144). Among kindergarteners (N = 46), scores ranged from 34.68% (43 points out of 124) to 99.19% (123/124). Adolescents’ scores ranged from 15.54% (23 points out of 148) to 100% (148/148). Norm ranges for total and subscale scores are detailed in Table 6.

### 3.4. Correlation Analyses

Among the 350 parent pairs in the children’s sample, 40.6% were classified as having a low, 37.4% a medium, and 22% a high occupational/educational status. In the adolescent sample (n = 471), 36.5% of parents were in the low, 34% in the medium, and 29.5% in the high category.

In the children’s sample (N = 530), a significant positive correlation was found between age and self-reported knowledge about their CHD (*p* < 0.001; r = 0.168), suggesting that older children tend to have a better understanding of their condition. Weak negative correlations were observed between the number of siblings and both the overall DsQoL score (*p* < 0.01; r = −0.118) and the MeHeartImpairments (*p* < 0.01; r = −0.132) and MeHeartSchool (*p* < 0.05; r = −0.105) subscales, indicating that having one or more siblings may be associated with slightly lower DsQoL scores in these areas.

Among the children in the reduced sample (N = 350), a weak but significant correlation was found between parental occupational/educational status and both self-reported knowledge about the CHD (*p* < 0.01; r = 0.159) and the overall DsQoL score (*p* < 0.001; r = 0.256), as well as five of the six subscales: MeHeartImpairments (*p* < 0.001; r = 0.265), MeHeartStigma (*p* < 0.001; r = 0.263), MeHeartRecovery (*p* < 0.01; r = 0.162), MeHeartFriends (*p* < 0.01; r = 0.163), and MeHeartSchool (*p* < 0.01; r = 0.176). Higher parental occupational/educational levels were associated with higher subjective knowledge about the CHD and better DsQoL ratings in most areas.

Across the full children’s sample (N = 530), self-reported knowledge about the CHD showed small to moderate significant correlations with the overall DsQoL score (*p* < 0.001; r = 0.275) and all six subscales: MeHeartImpairments (*p* < 0.001; r = 0.196), MeHeartStigma (*p* < 0.001; r = 0.162), MeHeartRecovery (*p* < 0.001; r = 0.276), MeHeartFriends (*p* < 0.001; r = 0.231), MeHeartTreatment (*p* < 0.001; r = 0.326), and MeHeartSchool (*p* < 0.001; r = 0.225). Thus, higher subjective knowledge was consistently associated with better DsQoL scores.

In the adolescent sample (N = 625), a weak but significant negative correlation was found between age and the subscale MeHeartStigma (*p* < 0.01; r = −0.135), suggesting that older adolescents may report more experiences of stigma or be more aware of stigma-related aspects of their condition, which could contribute to slightly lower scores in this subdomain. No significant associations were found between the number of siblings and either the overall DsQoL score or any of the subscales. Parental occupational/educational status (N = 471) showed one significant relationship in the MeHeartSchool subscale (*p* < 0.001; r = 0.170), where higher parental status was associated with better school-related DsQoL.

In terms of self-reported knowledge about the CHD, adolescents (N = 625) showed small but significant correlations with the overall DsQoL score (*p* < 0.001; r = 0.289) and all six subscales: MeHeartImpairments (*p* < 0.001; r = 0.234), MeHeartStigma (*p* < 0.001; r = 0.184), MeHeartRecovery (*p* < 0.001; r = 0.240), MeHeartFriends (*p* < 0.001; r = 0.251), MeHeartTreatment (*p* < 0.001; r = 0.267), and MeHeartSchool (*p* < 0.001; r = 0.245). Greater subjective knowledge was associated with more favorable DsQoL outcomes across the board.

### 3.5. Short Version of the CHDSI

Time constraints in clinical practice often require rapid assessments. Short questionnaires reduce time, burden, and costs and are helpful in studies. A six-item short version questionnaire was employed for both children and adolescents with CHD (CHDSI-SF; SF = Short Form). Each subscale is represented by its highest-loading item, yielding a total score of 0 to 24. For children, reliability (α = 0.749) and strong correlation with the long version (r = 0.916, *p* < 0.01) support cut-offs of 0 to 15 (critical) and 16 to 24 (non-critical). Adolescents show similar reliability (α = 0.750) and correlation (r = 0.914, *p* < 0.01) with slightly different cut-offs: 0 to 16 for males, 0 to 14 for females, and 0 to 15 overall. These short versions provide reliable overall CHDSI estimates but lack detailed subscale or severity analysis.

## 4. Discussion

To the best of our knowledge, the CHDSI is the first reliable, validated, and standardized instrument specifically designed to assess DsQoL in children and adolescents with CHD. Compared to widely used tools such as the PedsQL [52] and the PCQLI [53], the CHDSI offers several distinct advantages: it is normed on a recent, nationally large German CHD sample (N = 1201); it targets six empirically derived domains that are not fully addressed by existing instruments (e.g., stigma, recovery, healthcare experiences); and it provides both a comprehensive version and a validated short form suitable for DsQoL screening in clinical practice.

The CHDSI’s domain structure is uniquely aligned with the lived experiences of CHD patients, encompassing physical functioning, stigma, sleep/recovery, peer relationships, treatment experiences, and school functioning, each contextualized within the specific challenges of living with CHD. In contrast, the PedsQL includes broad generic domains (physical, emotional, social, school) and offers an optional cardiac module [52], while the PCQLI consolidates responses into two scored subscales [53] and does not explicitly capture aspects like recovery or stigma.

From a psychometric perspective, the CHDSI demonstrates high internal consistency and confirms a robust six-factor structure with excellent model fit across stratified age groups. While the PedsQL also exhibits strong reliability and well-established validity across diverse populations [52,54,55,56,57,58,59,60,61], and the PCQLI shows high reliability and good discriminative validity based on clinical severity [53,62], neither the PedsQL nor the PCQLI matches the domain disease-specific depth of the CHDSI.

In terms of clinical usability, the CHDSI is well suited for integration into routine care: the full version takes 5 to 10 min to complete, while the six-item CHDSI-SF allows for rapid screening in just 1 to 2 min. Although the PedsQL and PCQLI require similar administration times [52,53], neither the PedsQL nor the PCQLI offers an integrated solution that combines both in-depth disease-specific assessment and efficient screening within a single tool. Finally, the CHDSI is freely accessible (see Supplementary Materials), available in German, and supported by stanine norms and scoring tools, enhancing its suitability for widespread clinical adoption without the need for licensing agreements.

### 4.1. Model Fit

The CHDSI demonstrated excellent internal consistency, with Cronbach’s alpha values ranging from 0.856 to 0.914. These values exceed commonly accepted thresholds: while Peterson [115] considers α = 0.50 acceptable in certain contexts, Bortz and Döring [86] recommend a minimum of 0.80 for psychological instruments. Although higher alpha values suggest greater homogeneity, they can also be inflated by longer scales. While a high alpha helps protect against items that deviate from the overall pattern, Cortina [116] and Cronbach [117] emphasize that interpretability is more crucial than merely achieving an alpha > 0.70, especially since alpha can increase with more items [116,118,119]. According to Schmitt [118], there is no general level above which alpha becomes acceptable, as instruments with low alpha can also prove useful in certain circumstances.

Beyond internal consistency, the CHDSI demonstrated strong item-level discriminatory power, with most items exceeding the threshold of 0.50 recommended by Bortz and Döring [86] and falling well within Fisseni’s [120] “high” range. According to Fisseni, values below 0.30 are considered low, 0.30 to 0.50 medium, and above 0.50 high. High discriminatory power enhances the sensitivity of the instrument to differences in patient experiences and contributes to greater test homogeneity and interpretability [121].

For CFA, the DWLS method was selected due to the ordinal nature of Likert-type items and violations of multivariate normality assumptions. Although ML (maximum likelihood) estimation is frequently used [122], it assumes interval-scaled observed variables and can underestimate correlations in ordinal, skewed data [93,95,123,124,125]. ML can remain robust under mild skewness [126,127,128], but when data are clearly non-normal—as in this case—distribution-free methods like WLS [122,129] or ULS [122,130,131] are preferred. The choice of DWLS therefore strengthens the validity of parameter estimates and fit indices, especially in psychometric applications with ordinal data.

The χ^2^ statistic, while widely used [132,133], is well known for its sensitivity to sample size and assumption violations [134,135,136,137]. To mitigate this, the relative χ^2^ (χ^2^/df) was used, following Wheaton et al. [138,139], with interpretive thresholds ranging from <1 (excellent) to <5 (borderline). The observed values fell within the acceptable-to-excellent range, indicating good overall model fit.

The RMSEA (root mean square error of approximation) results were similarly positive. Recognized for prioritizing model parsimony [140], RMSEA is widely accepted as one of the most informative fit indices. Earlier standards considered values up to 0.10 acceptable [141], but more recent recommendations suggest cut-offs of 0.06 [105] or 0.07 [142]. The observed RMSEA values meet these modern standards, suggesting that the model structure balances complexity with explanatory power.

The CFI (Comparative Fit Index) and TLI (Tucker–Lewis Index) also indicated good model fit. These indices are relatively robust to sample size fluctuations [143] but can be affected when variable correlations approach zero [144,145]. Still, with CFI and TLI values generally exceeding the 0.95 benchmark recommended by Finney and DiStefano [146], model fit is considered strong. In line with current standard cut-offs [105,112,147,148,149], our CFA—using DWLS and based on two large samples—meets the criteria for robust goodness-of-fit evaluation.

### 4.2. Factors Influencing DsQoL

In adolescents, parental occupational and educational status had limited influence on DsQoL—likely due to greater autonomy and stronger peer- or school-related factors during this stage. The only significant association emerged in the school-related subscale, suggesting ongoing parental influence on academic support. A negative correlation between age and perceived stigma implies that older adolescents may be more aware of their condition’s social implications.

Parental occupational/educational status differed slightly between the child and adolescent groups, possibly because parents tend to complete education and establish careers after early parenthood. This may explain the slightly higher parental levels in the adolescent sample.

In children, higher parental occupation/education levels and older age were significantly associated with better DsQoL and greater subjective knowledge about the CHD. These findings underline the importance of cognitive maturity and a supportive home environment in shaping children’s understanding and management of their condition.

This is supported by Wang et al. [150], who found that parental hope, spirituality, and occupational prestige predicted better QoL in Chinese adolescents with CHD. Similarly, Abassi et al. (2020) reported that while young children with CHD rated their QoL similarly to healthy peers, parents reported lower QoL in cases with higher disease severity or more invasive procedures [151]. Drakouli et al. also found that QoL in children with CHD was shaped by factors such as parental support, socioeconomic status, emotional well-being, and a sense of coherence [17], highlighting the need for child-centered tools like the CHDSI.

Though not designed for family-wide assessment, the CHDSI provides essential insights into the child’s perspective. Interestingly, a negative correlation between number of siblings and DsQoL in the children’s group suggests that shared parental resources may impact perceived DsQoL—a factor worth considering in interventions.

Across both groups, self-reported CHD knowledge was associated with higher DsQoL. This highlights the value of health literacy and psychoeducation in empowering patients and improving outcomes. Stronger effects in younger children suggest greater dependence on parental involvement early on, while adolescents may benefit more from peer support and independent coping.

Finally, as Bartoletti et al. noted, while survival in CHD has improved, QoL outcomes vary and depend on psychosocial factors like coping, support, and coherence [152]. The CHDSI enables more targeted, multifactorial research into these influences and supports individualized, holistic care for children and adolescents with CHD.

### 4.3. Representativeness and Generalizability

Excluding the small kindergarten sample (N = 46), both the children (N = 530) and adolescent (N = 625) groups in this study were sufficiently large to ensure adequate statistical power for detecting meaningful correlations. While the literature offers limited and sometimes contradictory guidance on required sample sizes for CFA [105,112,113,114], it is well established that CFA results (e.g., parameter estimates, chi-square tests, and fit indices) are sensitive to sample size. Both groups exceeded the commonly recommended minimum of >500, thus meeting standard methodological requirements.

At present, variables such as migration background, ethnicity, and religion are not routinely collected in many German health studies due to historical, legal, and institutional reasons. This limitation was also present in the development of the CHDSI. A comparable situation is seen in a multisite German validation of the preschool-specific CHD QoL instrument (P-PCQLI), which similarly did not report subgroup analyses by ethnicity or immigrant status [153]. In a nationwide study of preschool children in Germany (N = 283, 81% with migrant background), Villalonga-Olives et al. [154] found no significant differences in self-reported HrQoL using the Kiddy-KINDL instrument; in fact, migrant children reported slightly higher scores. These findings imply that, particularly in early childhood, migration background may not negatively affect perceived QoL. While existing evidence suggests that differences in pediatric QoL by migration background may be less pronounced than assumed, dedicated studies in diverse subgroups are needed to enhance understanding. As Germany becomes increasingly diverse, future CHDSI validation studies should explicitly assess its performance in minority and immigrant subgroups to ensure cross-cultural validity.

The overall response rate of 10.1% might initially raise concerns about non-response bias. However, Wu et al. (2022) concluded that an 80% response rate is not required for survey validity [155]. Similarly, Fosnacht et al. (2017) found that in online surveys with sample sizes exceeding 500, response rates as low as 5 to 10% can still yield reliable estimates; higher rates are mainly relevant for smaller samples [156]. In the present study, participant demographics closely mirrored those of the invited population in key areas: mean age was 12.76 years in both groups, and the sex distribution was comparable (48% male among participants vs. 50.9% in the invited group), suggesting minimal demographic bias.

However, a potential participation bias was observed in relation to CHD severity. Response rates were higher among patients with moderate (13.49%) and complex CHD (13.96%) compared to those with simple (6.97%) or unclassified conditions (6.46%). This suggests a potential overrepresentation of more severely affected individuals in the analytical sample. Therefore, while representativeness is supported for the total sample, caution is warranted when interpreting subgroup findings by CHD severity, particularly due to smaller sample sizes. Additionally, patients with unclassified CHD were excluded from severity-based analyses to maintain analytical clarity and consistency.

### 4.4. Additional Benefits of the CHDSI

The CHDSI enables individualized, patient-centered care. The CHDSI is offered free of charge to cardiac centers, clinics, and general practitioners (see Supplementary Materials) to provide a more holistic view of children and adolescents with CHD.

The CHDSI is intended to identify specific areas of DsQoL impairment, such as CHD or treatment-related problems that affect or disrupt the children and adolescents’ everyday lives. It also enables practitioners to address patient fears or concerns on a more individual basis and thus improve the doctor–patient relationship. In particular, a free-text field, which was explicitly requested by the children and adolescents, gives CHD patients the opportunity to communicate stressful or otherwise unspoken concerns and thus deepen the clinicians’ understanding of their life situation. This is a starting point for addressing acute patient life issues (e.g., sports restrictions due to parent’s fear), as well as for tailored psychosocial or educational approaches that can be initiated if necessary.

The CHDSI-SF offers the opportunity for rapid screening in under two minutes, facilitating its integration into routine clinical workflows. If the CHDSI or CHDSI-SF is used routinely, practitioners are able to notice and respond to DsQoL changes more quickly.

In research, the CHDSI can be used in clinical studies on the current status of children and adolescents with CHD to get a better impression of which areas and issues particularly affect them. In addition, the CHDSI can be used in longitudinal studies to identify particularly vulnerable periods or phases of life or to monitor DsQoL over the treatment span. The CHDSI also has the potential to inform the design of personalized treatment strategies, school integration measures, and broader care models that respond to both medical and psychosocial needs.

In summary, the CHDSI enhances the possibility of measuring DsQoL in pediatric cardiology by offering a disease-specific, psychometrically robust, and clinically feasible instrument grounded in the lived experiences of children and adolescents with CHD.

### 4.5. Limitations

The CHDSI was developed, validated, and standardized using an online sample. While online participation—often from home—offers advantages in reach and accessibility, future validation studies in clinical settings (e.g., hospitals or outpatient care) are necessary to confirm or refine the current norm ranges and ensure applicability in real-world practice.

Cultural and religious factors were not explicitly addressed during the development process, limiting the cross-cultural comparability of the CHDSI. Although the nationwide sample likely reflects aspects of Germany’s diversity, further studies in varied cultural contexts are essential to assess the tool’s generalizability and to support appropriate cultural adaptations.

The kindergarten version of the CHDSI was based on a small sample (N = 46), which restricts the reliability of normative data for this group. Larger-scale studies are needed to strengthen the validity and interpretability of the results for younger children.

Additionally, the analytic sample included a higher proportion of individuals with moderate and complex CHD, potentially biasing the normative data toward more clinically apparent cases. However, this limitation is minor, as the CHDSI is primarily intended for use in clinically significant cases (e.g., patients who attend at least regular check-up examinations). Future research should aim for more balanced representation across CHD severity groups to improve generalizability.

Finally, as the CHDSI was developed in German, its use in other languages or cultural contexts will require careful translation, cultural adaptation, and psychometric revalidation. This will help ensure the tool remains accurate, meaningful, and clinically useful across diverse settings.

To enhance the CHDSI’s utility, future research should also explore its application in longitudinal studies to track changes in DsQoL over time and evaluate the effectiveness of interventions aimed at improving outcomes for children and adolescents with CHD.

## 5. Conclusions

The CHDSI is the first validated German-language questionnaire for assessing DsQoL in children and adolescents with CHD. It demonstrates strong psychometric properties, with high internal consistency at the total scale level and acceptable to excellent subscale reliabilities. CFA showed excellent model fit across child and adolescent samples, confirming the conceptual structure derived from prior qualitative research.

The CHDSI also showed good discriminative validity, distinguishing between patients based on sex and CHD complexity, highlighting its clinical sensitivity. Its potential as a reliable screening and monitoring tool makes it suitable for both clinical and research settings. Normative data from large samples of children and adolescents, with standardization tables stratified by sex and CHD severity, support meaningful score interpretation.

The CHDSI-SF for children as well as adolescents offers additional screening options, especially in everyday clinical practice. Nevertheless, the CHDSI full version remains the most comprehensive tool.

In the case of the CHDSI kindergarten version, the limited sample size reduces the overall reliability of the results for this subsample. Therefore, the kindergarten version should be evaluated on a larger patient group in the future to confirm or adapt the results.

Overall, the CHDSI enables systematic evaluation of DsQoL in pediatric CHD populations and holds promise for nationwide, long-term clinical and research use. Future international collaboration will be crucial to adapting and validating the CHDSI across languages and cultures, ensuring its role in improving global care for children and adolescents with CHD.

## Figures and Tables

**Table 1:** Overview comparison of CHDSI, PedsQL, and PCQLI.

Feature	CHDSI	PedsQL (Generic and Cardiac)	PCQLI (Pediatric Cardiac)
*Construct assessed*	DsQoL of young CHD patients.	Pediatric HrQoL	HrQoL in pediatric cardiac patients
*Domains* */subscales*	Six disease-specific domains (physical limitations, stigma, sleep/recovery, peer relationships, healthcare experiences, and academic challenges)	The Generic Core Module covers four domains: physical, emotional, social, and school function. The Cardiac Module assesses six additional dimensions.	There are two scored subscales, Disease Impact (physical) and Psychosocial Impact, measuring the impact of the disease.
*Age range*	Three age-specific versions: Preschool (31 items, 5 scales, for age 6), Children (36 items, 6 scales, ages 6 to under 14), and Adolescents (37 items, 6 scales, ages 14 to under 18)	Two main generic self-report versions: one for children aged 5–18 and a parent-proxy version for ages 2–18 (Cardiac Module is available for patients aged 5 to 18 years).	Two main versions: a self-report for 8 to 18 years and a parent-proxy version
*Normative sample*	Validated and standardized in CHD patients aged 6 to <18 years nationwide in Germany through the National Register for Congenital Heart Defects (data collected online in 2022; N = 1201)	Validated in U.S. pediatric populations in the early 2000s on a sample of 1677 participants (963 children aged 5 to 18 and 1629 parents of children aged 2 to 18).	Validation in the early 2010s using an pediatric U.S. cardiac cohort of 1605 patients (8 to 18 years)
*Internal consistency (Cronbach’s α)*	Cronbach’s alpha from 0.86 to 0.89 for kindergarten, 0.89 to 0.90 for children and adolescents (subscale alphas vary between 0.68 and 0.91)	Generic total score shows alpha range from 0.88 to 0.90 (subscale reliability varies between 0.70 and 0.90).	The total score alpha was up to 0.91 (disease-related subscale alphas around 0.90, psychosocial subscale around 0.85).
*Construct validity* */Factor structure*	CFA confirmed a six-domain model (CFI and TLI values above 0.99 and RMSEA around 0.05 with good discriminative validity for sex and CHD complexity).	Validation supports four generic domains (CFI between 0.945 and 0.956, NFI between 0.928 and 0.950, and RMSEA between 0.066 and 0.074 with good discrimination between healthy chronic conditions, disease severity, and health status).	Two primary subscales “Impact of Disease” (ID) and “Psychosocial Impact” (PI) together accounted for 55 to 60 percent of the total variance (PCA); there was a moderate to strong correlation (r ≈ 0.70) with PedsQL total scores in multicenter studies.
*Administration time*	Full versions take 5 to 10 min and the short form takes 1 to 2 min.	PedsQL Generic Core (5 min), Cardiac Module adds 2 min	Full version takes 5 to 10 min
*Clinical vs. Research use*	Designed for both routine clinical follow-up and research use in young CHD patients (stanine norms and a short form support efficient screening and help guide intervention decisions).	Widely used in both clinical practice and research (suitable for various conditions and comparative trials)	Primarily used in pediatric cardiology research and clinical settings (focuses on heart-specific effects, well suited for longitudinal follow-up)

CHDSI = Congenital Heart Disease Specific Inventory; PedsQL = Pediatric Quality of Life Inventory; PCQLI = Pediatric Cardiac Quality of Life Inventory; CHD = congenital heart disease/defect; DsQoL = disease-specific quality of life; HrQoL = health-related quality of life; PCA = principal components analysis; CFA = confirmatory factor analysis; CFI = Comparative Fit Index; TLI = Tucker–Lewis Index; NNFI = Non-Normed Fit Index; RMSEA = root mean square error of approximation.

**Table 2:** Postulated scales/latent variables.

Scale	Children	Adolescents
**MeHeartImpairments**		
	I could do everything without getting out of breath.	I could do everything without getting out of breath.
	I had no physical pain.	I had no physical pain.
	I have rarely felt ill.	I have rarely felt ill.
	I was able to do my hobbies without any problems.	I was able to do my hobbies without any problems.
	I felt physically fresh and alert.	I felt physically fresh and alert.
	… quickly run out of breath.	… quickly run out of breath.
	… often have to deal with dizziness.	… often have to deal with dizziness.
	… also have pain in the chest.	… also feel pain in the chest.
	… noticed that I get tired quickly.	… noticed that I get tired quickly.
	… often feel a strange palpitation.	… often feel a strange palpitation.
	I could do all the physical activities I wanted.	I could do all the physical activities I wanted.
**MeHeartStigma**		
	… my parents worried a lot, I’m sorry about that.	… my parents worried a lot, I’m sorry about that.
	* … teachers and classmates behave strangely toward me at school.	… teachers and classmates behave strangely toward me at school.
	… my parents and other adults are overprotective of me.	… my parents and other adults are overprotective of me.
	*… teachers and classmates made a special effort for me without me wanting it.	… teachers and classmates made a special effort for me without me wanting it.
	… other children made fun of me.	… others made fun of me.
	** I missed a lot of lessons because of medical examinations.	I missed a lot of lessons because of medical examinations.
	I felt helpless and sad.	I felt helpless and sad.
	** I was worried about whether I would make it through school.	I was worried about whether I would manage school/training.
	I was worried about my future.	I wondered whether I would find a boyfriend/girlfriend with my heart defect.
	** I couldn’t do everything in PE lessons.	I felt restricted in my independence due to my heart defect.
		I was worried about what job I could do with my heart defect.
		I wasn’t worried about my future at all.
**MeHeartRecovery**		
	I couldn’t fall asleep well.	I couldn’t fall asleep well.
	I often woke up at night.	I often woke up at night.
	I struggled to get out of bed in the morning.	I struggled to get out of bed in the morning.
	I woke up feeling refreshed.	I woke up feeling refreshed.
	I had a peaceful, sound sleep.	I had a peaceful, sound sleep.
	I recovered well after a strenuous day.	I recovered well after a strenuous day.
**MeHeartFriends**		
	I was able to take part in activities with friends.	I was able to take part in activities with friends.
	I had difficulties finding friends.	I had no difficulty making friends.
	I felt equally good compared to my friends.	I felt just as independent as my friends.
	I felt comfortable as I am.	
**MeHeartTreatment**		
	I’m not afraid of the hospital.	I’m not afraid of the hospital.
	I find visits to the doctor unpleasant.	I find visits to the doctor unpleasant.
	I don’t like having my heart examined.	I don’t like having my heart examined.
**MeHeartSchool**		
	** I did well with the homework.	I did well with the homework.
	** I kept up well with the lesson material.	I kept up well with the lesson material.

* Children who do not yet go to school are asked to replace “school” with “kindergarten”, “teacher” with “educator”, and classmates with “other children” for these questions. ** These items are not relevant for the evaluation of children who are not yet at school.

**Table 3:** Invited vs. participating CHD patients.

		Invited CHD Patients N = 11,906	Participated Patients N = 1201
Overall response rate		10.1%	
Sex	Male	50.9%	48%
	Female	49.1%	52%
Age (mean ± standard deviation)	In years	12.76 ± 3.05	12.76 ± 3.12
CHD severity/complexity	Simple	317 out of 4554 (6.97%)	
	Moderate	417 out of 3091 (13.49%)	
	Complex	357 out of 2557 CHD (13.96%)	
	Unclassified	110 out of 1704 CHD (6.46%)	

CHD = congenital heart disease/defect.

**Table 4:** Significant mean differences in DsQoL total score and subscales.

CHDSI-Version	Comparison	Scale	*p*-Value	Mean ± SD (Group A)	Mean ± SD (Group B)
**Children**	Simple CHD (Group A) vs. Moderate CHD (Group B)	DsQoL Total	<0.01	121.35 ± 20.77	114.00 ± 23.20
		MeHeartImpairments	<0.05	36.99 ± 8.68	34.76 ± 8.85
		MeHeartStigma	<0.001	36.26 ± 5.29	33.45 ± 6.90
		MeHeartFriends	<0.05	14.19 ± 2.50	13.56 ± 2.79
		MeHeartSchool	<0.01	6.98 ± 1.64	6.39 ± 1.93
	Simple CHD (Group A) vs. Complex CHD (Group B)	DsQoL Total	<0.001	121.35 ± 20.77	107.09 ± 23.65
		MeHeartImpairments	<0.001	36.99 ± 8.68	31.93 ± 9.29
		MeHeartStigma	<0.001	36.26 ± 5.29	31.27 ± 7.16
		MeHeartRecovery	<0.01	18.60 ± 5.18	16.94 ± 5.26
		MeHeartFriends	<0.001	14.19 ± 2.50	12.89 ± 3.22
		MeHeartSchool	<0.01	6.98 ± 1.64	6.29 ± 1.97
	Moderate CHD (Group A) vs. Complex CHD (Group B)	DsQoL Total	<0.01	114.00 ± 23.20	107.09 ± 23.65
		MeHeartImpairments	<0.01	34.76 ± 8.85	31.93 ± 9.29
		MeHeartStigma	<0.01	33.45 ± 6.90	31.27 ± 7.16
		MeHeartFriends	<0.05	13.56 ± 2.79	12.89 ± 3.22
**Adolescents**	Male (Group A) vs. Female (Group B)	DsQoL Total	<0.001	118.40 ± 22.43	109.32 ± 26.46
		MeHeartImpairments	<0.001	34.57 ± 8.70	30.18 ± 10.50
		MeHeartStigma	<0.05	40.17 ± 8.01	38.79 ± 9.41
		MeHeartRecovery	<0.001	18.31 ± 4.81	16.53 ± 5.84
		MeHeartTreatment	<0.001	8.82 ± 2.98	7.68 ± 3.40
	Simple CHD (Group A) vs. Moderate CHD (Group B)	DsQoL Total	<0.001	120.70 ± 22.27	113.09 ± 24.30
		MeHeartStigma	<0.001	42.81 ± 7.25	39.11 ± 8.74
		MeHeartFriends	<0.05	10.46 ± 1.96	9.90 ± 2.66
	Simple CHD (Group A) vs. Complex CHD (Group B)	DsQoL Total	<0.001	120.70 ± 22.27	108.38 ± 25.11
		MeHeartImpairments	<0.001	34.36 ± 9.53	30.48 ± 9.79
		MeHeartStigma	<0.001	42.81 ± 7.25	36.46 ± 9.09
		MeHeartFriends	<0.001	10.46 ± 1.96	9.58 ± 2.64
	Moderate CHD (Group A) vs. Complex CHD (Group B)	MeHeartImpairments	<0.05	32.79 ± 9.44	30.48 ± 9.79
		MeHeartStigma	<0.01	39.11 ± 8.74	36.46 ± 9.09

CHD = congenital heart disease/defect; SD = standard deviation.

**Table 5:** Overview of CFA estimation parameters; children’s version of the CHDSI (N = 530) and adolescent version of the CHDSI (N = 625).

	Children’s Version of the CHDSI		Adolescent Version of the CHDSI	
Estimator	DWLS		DWLS	
Optimization method	NLMINB		NLMINB	
Number of model parameters	195		200	
Number of observations	530		625	
**Model Test User Model:**				
	***Standard***	***Robust***	***Standard***	***Robust***
Test statistic	1276.050	1492.876	1580.638	1806.846
Degrees of freedom	579	579	614	614
*p*-Value (chi-square)	<0.001	<0.001	<0.001	<0.001
Scaling correction factor		1.072		1.061
Shift parameter		302.352		316.623
**Model Test Baseline Model:**				
	***Standard***	***Robust***	***Standard***	***Robust***
Test statistic	103,271.246	23,869.066	120,589.394	27,437.643
Degrees of freedom	630	630	666	666
*p*-Value	<0.001	<0.001	<0.001	<0.001
Scaling correction factor		4.417		4.479
**User Model versus Baseline Model:**				
	***Standard***	***Robust***	***Standard***	***Robust***
Comparative Fit Index (CFI)	0.993	0.961	0.992	0.955
Tucker–Lewis Index (TLI)	0.993	0.957	0.991	0.952
**Root Mean Square Error of Approximation:**				
	***Standard***	***Robust***	***Standard***	***Robust***
RMSEA	0.048	0.055	0.050	0.056
90 Percent confidence interval—lower	0.044	0.051	0.047	0.053
90 Percent confidence interval—upper	0.051	0.058	0.053	0.059
*p*-value RMSEA ≤ 0.05	0.855	0.013	0.446	0.001
**Standardized Root Mean Square Residual:**				
	***Standard***	***Robust***	***Standard***	***Robust***
SRMR	0.052	0.052	0.054	0.054
**Parameter Estimates:**				
Standard errors	Robust.sem		Robust.sem	
Information	Expected		Expected	
Information saturated (h1) model	Unstructured		Unstructured	

DWLS = diagonally weighted least squares; NLMINB = nonlinear minimization using bounds; CFI = Comparative Fit Index; TLI = Tucker–Lewis Index; RMSEA = root mean square error of approximation; SRMR = standardized root mean square residual.

**Table 6:** Score range and standardization values.

Interpretation of the DsQoL Scores Achieved						
***Kindergarten Children***						
	total N = 46					
**DsQoL total score (0**–**124 points)**						
Critical range	0–84					
Uncritical range	85–124					
**MeHeartImpairments (0**–**44 points)**						
Critical range	0–28					
Uncritical range	29–44					
**MeHeartStigma (0**–**28 points)**						
Critical range	0–18					
Uncritical range	19–28					
**MeHeartRecovery (0**–**24 points)**						
Critical range	0–15					
Uncritical range	16–24					
**MeHeartFriends (0**–**16 points)**						
Critical range	0–11					
Uncritical range	12–16					
**MeHeartTreatment (0**–**12 points)**						
Critical range	0–4					
Uncritical range	5–12					
***Children***						
	total N = 530	male n = 248	female n = 282	simple CHD n = 119	moderate CHD n = 194	complex CHD n = 173
**DsQoL total score (0**–**144 points)**						
Critical range	0–95	0–96	0–95	0–108	0–96	0–89
Uncritical range	96–144	97–144	96–144	109–144	97–144	90–144
**MeHeartImpairments (0**–**44 points)**						
Critical range	0–27	0–26	0–27	0–29	0–28	0–24
Uncritical range	28–44	27–44	28–44	30–44	29–44	25–44
**MeHeartStigma (0**–**40 points)**						
Critical range	0–28	0–28	0–28	0–34	0–29	0–24
Uncritical range	29–40	29–40	29–40	35–40	30–40	25–40
**MeHeartRecovery (0**–**24 points)**						
Critical range	0–13	0–14	0–13	0–14	0–14	0–13
Uncritical range	14–24	15–24	14–24	15–24	15–24	14–24
**MeHeartFriends (0**–**16 points)**						
Critical range	0–11	0–11	0–11	0–12	0–11	0–10
Uncritical range	12–16	12–16	12–16	13–16	12–16	11–16
**MeHeartTreatment (0**–**12 points)**						
Critical range	0–5	0–5	0–5	0–5	0–5	0–5
Uncritical range	6–12	6–12	6–12	6–12	6–12	6–12
**MeHeartSchool (0**–**8 points)**						
Critical range	0–5	0–5	0–5	0–6	0–5	0–5
Uncritical range	6–8	6–8	6–8	7–8	6–8	6–8
***Adolescents***						
	total N = 625	male n = 298	female n = 327	simple CHD n = 195	moderate CHD n = 207	complex CHD n = 165
**DsQoL total score (0**–**148 points)**						
Critical range	0–93	0–101	0–88	0–107	0–93	0–87
Uncritical range	94–148	102–148	89–148	108–148	94–148	88–148
**MeHeartImpairments (0**–**44 points)**						
Critical range	0–24	0–27	0–21	0–28	0–24	0–23
Uncritical range	25–44	28–44	22–44	29–44	25–44	24–44
**MeHeartStigma (0**–**48 points)**						
Critical range	0–33	0–34	0–32	0–40	0–33	0–29
Uncritical range	34–48	35–48	33–48	41–48	34–48	30–48
**MeHeartRecovery (0**–**24 points)**						
Critical range	0–13	0–14	0–12	0–14	0–13	0–13
Uncritical range	14–24	15–24	13–24	15–24	14–24	14–24
**MeHeartFriends (0**–**12 points)**						
Critical range	0–8	0–8	0–8	0–9	0–8	0–7
Uncritical range	9–12	9–12	9–12	10–12	9–12	8–12
**MeHeartTreatment (0**–**12 points)**						
Critical range	0–5	0–6	0–4	0–6	0–4	0–5
Uncritical range	6–12	7–12	5–12	7–12	5–12	6–12
**MeHeartSchool (0**–**8 points)**						
Critical range	0–5	0–5	0–5	0–5	0–5	0–5
Uncritical range	6–8	6–8	6–8	6–8	6–8	6–8

DsQoL = disease-specific quality of life; CHD = congenital heart defect.

## Data Availability

Data cannot be shared for data protection reasons.

## Supplementary Materials

The following supporting information can be downloaded at: https://www.mdpi.com/article/10.3390/medicina61071311/s1. As supplementary material; all questionnaires for kindergarten children, children, and adolescents; and a comprehensive manual for questionnaire development (each in German; German was also the validation/norming language), as well as corresponding SPSS syntax files and SPSS data sets (each bilingual in German and English), are available.
