# Supplementary material for: Development and Validation of the CHDSI Questionnaire: A New Tool for Measuring Disease-Specific Quality of Life in Children and Adolescents with Congenital Heart Defects
Source: Medicina (Kaunas). 2025 Jul 21;61(7):1311. doi: 10.3390/medicina61071311 (PMC12297989; doi:10.3390/medicina61071311)
Supplement: Supplementary file 1 [file medicina-61-01311-s001.zip › 10. Supplemental Material 2a - CHDSI Questionaire (Long - Adolescents) in German.pdf]

CHDSI

Congenital **H**eart **D**isease **S**pecific **I**nventory
Krankheitsspezifische Lebensqualität von Kindern und Jugendlichen mit angeborenem Herzfehler
von 14 bis 17 Jahre

Vorname: ____________________ Nachname: ________________________

Alter: ________ Geschlecht: ________ Geschwister: Mädchen ___ Alter: ______
Junge ____ Alter: ______

Kindergartenjahre: ___ Schulart: ________________ Klasse: __ Ausbildung: ____________

Schulabschluss Eltern: Mama ________________ Papa ____________________

Berufsausbildung Eltern: Mama ________________ Papa ____________________

Berufstätig: Mama ○ Papa ○ beide ○ keiner ○

Berufstätigkeit Eltern: Vollzeit Mama ○ Papa ○ | Teilzeit Mama ○ Papa ○ | Hausfrau/-mann Mama ○ Papa ○ | Andere: ______________

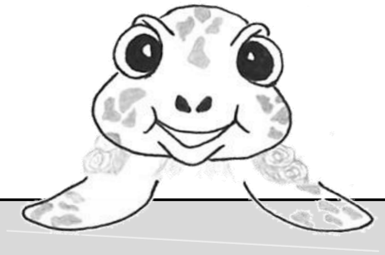

Hier oben steht immer ein Bereich um den es in den Fragen geht

Dann folgt die Frage:

Dann machst Du Dein Kreuz wo Du es am Besten passend findest

**Welche Aussage trifft auf Dich zu?**	**trifft voll zu**	**trifft zu**	**weiß nicht**	**trifft nicht zu**	**trifft gar nicht zu**
Ich esse gerne Eis	X	○	○	○	○
Ich lese gerne	○	○	X	○	○

Vorsicht manche Fragen sind umgedreht, das erkennst Du an den Smileys:

**Welche Aussage trifft auf Dich zu?**	**trifft voll zu**	**trifft zu**	**weiß nicht**	**trifft nicht zu**	**trifft gar nicht zu**
Ich gehe nicht gerne spazieren	○	X	○	○	○
Ich bin oft traurig	○	○	○	X	○

Copyright © 2025 by J. Remmele & P. C. Helm

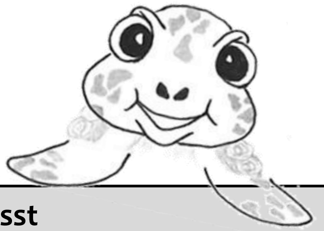

In den letzten Wochen hat mich mein Herz in meiner Selbstständigkeit beeinflusst

Welche Aussage trifft auf Dich zu?	trifft gar nicht zu	trifft nicht zu	weiß nicht	trifft zu	trifft voll zu
1. Ich fühlte mich genauso selbstständig wie meine Freunde.	○	○	○	○	○
2. Ich hatte keine Schwierigkeiten Freunde zu finden.	○	○	○	○	○
3 Ich konnte an Aktivitäten mit Freunden teilnehmen.	○	○	○	○	○

	trifft voll zu	trifft zu	weiß nicht	trifft nicht zu	trifft gar nicht zu
4. Ich habe mich gefragt ob ich mit meinem Herzfehler eine/n feste/n Freund/in finde.	○	○	○	○	○
5. Ich habe mich hilflos und traurig gefühlt.	○	○	○	○	○
6. Ich fühlte mich durch meinen Herzfehler in meiner Selbstständigkeit eingeschränkt.	○	○	○	○	○

Wegen meinem Herz haben sich in den letzten Wochen …

Welche Aussage trifft auf Dich zu?		trifft voll zu	trifft zu	weiß nicht	trifft nicht zu	trifft gar nicht zu
7. … meine Eltern viele Sorgen gemacht, das tut mir leid.		○	○	○	○	○
8. … in der Schule/Ausbildung Lehrer und Mitschüler mir gegenüber komisch verhalten.		○	○	○	○	○
9. … meine Eltern mir gegenüber anders verhalten als meinen Geschwistern gegenüber.	○ habe keine	○	○	○	○	○
10. … meine Eltern und andere Erwachsene übervorsichtig mir gegenüber verhalten.		○	○	○	○	○
11. … Lehrer und Mitschüler besonders um mich bemüht, ohne dass ich das wollte.		○	○	○	○	○
12. … Andere über mich lustig gemacht.		○	○	○	○	○

Copyright © 2025 by J. Remmele & P. C. Helm

In den letzten Wochen hat mich mein Herz in der Schule / Ausbildung beeinflusst

Welche Aussage trifft auf Dich zu?	trifft voll zu	trifft zu	weiß nicht	trifft nicht zu	trifft gar nicht zu
13. Ich habe viel Unterricht verpasst wegen Untersuchungen.	○	○	○	○	○
14. Ich war besorgt, welchen Beruf ich mit meinem Herzfehler machen kann.	○	○	○	○	○
15. Ich habe mir Gedanken gemacht, ob ich die Schule / Ausbildung schaffe.	○	○	○	○	○

	trifft gar nicht zu	trifft nicht zu	weiß nicht	trifft zu	trifft voll zu
16. Ich habe mir überhaupt keine Sorgen um meine Zukunft gemacht.	○	○	○	○	○
17. Ich bin mit den Hausaufgaben gut zurechtgekommen.	○	○	○	○	○
18. Ich bin mit dem Unterrichtsstoff gut mitgekommen.	○	○	○	○	○

In den letzten Wochen hat mich mein Herz körperlich eingeschränkt

Welche Aussage trifft auf Dich zu?	trifft gar nicht zu	trifft nicht zu	weiß nicht	trifft zu	trifft voll zu
19. Ich konnte alle körperlichen Aktivitäten machen wie ich wollte.	○	○	○	○	○
20. Ich konnte alles machen ohne außer Puste zu geraten.	○	○	○	○	○
21. Ich hatte keine körperlichen Schmerzen.	○	○	○	○	○
22. Ich habe mich selten krank gefühlt.	○	○	○	○	○
23. Ich konnte problemlos meine Hobbies machen.	○	○	○	○	○
24. Ich fühlte mich körperlich frisch und munter.	○	○	○	○	○

Copyright © 2025 by J. Remmele & P. C. Helm

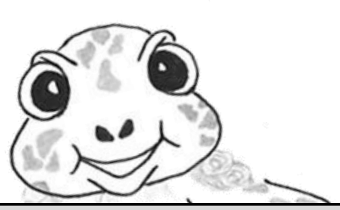

In den letzten Wochen hatte ich wegen meinem Herz …

Welche Aussage trifft auf Dich zu?	trifft voll zu	trifft zu	weiß nicht	trifft nicht zu	trifft gar nicht zu
25. … schnell keine Puste mehr.	○	○	○	○	○
26. … abends öfter dicke Beine und Füße.	○	○	○	○	○
27. … häufig mit Schwindel zu tun.	○	○	○	○	○
28. … auch Schmerzen in der Brust.	○	○	○	○	○
29. … gemerkt, dass ich schnell müde werde.	○	○	○	○	○
30. … öfter komisches Herzklopfen.	○	○	○	○	○

Wie sieht es mit der Erholung von Dir und Deinem Herz in den letzten Wochen aus?

Welche Aussage trifft auf Dich zu?	trifft voll zu	trifft zu	weiß nicht	trifft nicht zu	trifft gar nicht zu
31. Ich konnte nicht gut einschlafen.	○	○	○	○	○
32. Ich bin nachts häufig aufgewacht.	○	○	○	○	○
33. Ich bin morgens schwer aus dem Bett gekommen.	○	○	○	○	○

	trifft gar nicht zu	trifft nicht zu	weiß nicht	trifft zu	trifft voll zu
34. Ich bin erholt aufgewacht.	○	○	○	○	○
35. Ich hatte einen ruhigen festen Schlaf.	○	○	○	○	○
36. Ich habe mich gut nach anstrengenden Tagen erholt.	○	○	○	○	○

Copyright © 2025 by J. Remmele & P. C. Helm

Du und Dein Herz allgemein

Welche Aussage trifft auf Dich zu?		trifft gar nicht zu	trifft nicht zu	weiß nicht	trifft zu	trifft voll zu
37. Ich weiß gut Bescheid über mein Herz und was ihm fehlt.		○	○	○	○	○
38. Vor dem Krankenhaus habe ich keine Angst.		○	○	○	○	○
39. Ich vertrage meine Medikamente gut.	○ nehme keine	○	○	○	○	○

		trifft voll zu	trifft zu	weiß nicht	trifft nicht zu	trifft gar nicht zu
40. Ich finde Arztbesuche unangenehm.		○	○	○	○	○
41. Ich mag es nicht am Herzen untersucht zu werden.		○	○	○	○	○
42. Ich finde es nervig Medikamente zu nehmen.	○ nehme keine	○	○	○	○	○
43. Ich finde meine Operationsnarbe sehr unangenehm.	○ habe keine	○	○	○	○	○

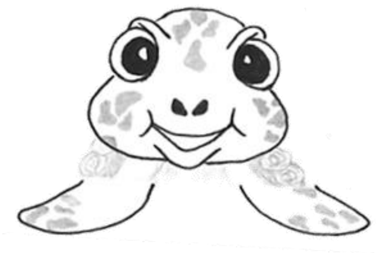

Gibt es etwas, das Du wichtig findest und hier noch nicht dabei war?

____

____

____

____

____

____

____

Vielen Dank, dass du die Fragen beantwortet hast!

Copyright © 2025 by J. Remmele & P. C. Helm
