# Supplementary material for: Development and Validation of the CHDSI Questionnaire: A New Tool for Measuring Disease-Specific Quality of Life in Children and Adolescents with Congenital Heart Defects
Source: Medicina (Kaunas). 2025 Jul 21;61(7):1311. doi: 10.3390/medicina61071311 (PMC12297989; doi:10.3390/medicina61071311)
Supplement: Supplementary file 1 [file medicina-61-01311-s001.zip › 11. Supplemental Material 2b - CHDSI-SF Questionaire (Short - Adolescents) in German.pdf]

CHDSI Kurzversion

Congenital **H**eart **D**isease **S**pecific **I**nventory
Krankheitsspezifische Lebensqualität von Kindern und Jugendlichen mit angeborenem Herzfehler
von 14 bis 17 Jahre

Vorname: ______________________ Nachname: ______________________

Alter: ________ Geschlecht: ________ Geschwister: Mädchen ____ Alter: ________
Junge ____ Alter: ________

Kindergartenjahre: ____ Schulart: ______________ Klasse: ___ Ausbildung: ______________

Schulabschluss Eltern: Mama ______________ Papa ______________

Berufsausbildung Eltern: Mama ______________ Papa ______________

Berufstätig: Mama ○ Papa ○ beide ○ keiner ○

Berufstätigkeit Eltern:

Vollzeit	Teilzeit	Hausfrau/-mann	Andere: ______________
Mama ○	Mama ○	Mama ○	
Papa ○	Papa ○	Papa ○	

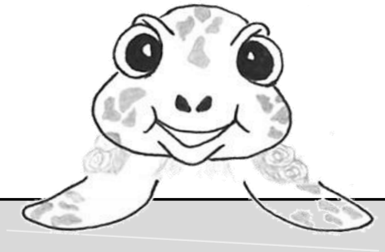

Hier oben steht immer ein Bereich um den es in den Fragen geht

Dann folgt die Frage:

Dann machst Du Dein Kreuz wo Du es am Besten passend findest

**Welche Aussage trifft auf Dich zu?**	trifft voll zu	trifft zu	weiß nicht	trifft nicht zu	trifft gar nicht zu
Ich esse gerne Eis	X	○	○	○	○
Ich lese gerne	○	○	X	○	○

Vorsicht manche Fragen sind umgedreht, das erkennst Du an den Smileys:

**Welche Aussage trifft auf Dich zu?**	trifft voll zu	trifft zu	weiß nicht	trifft nicht zu	trifft gar nicht zu
Ich gehe nicht gerne spazieren	○	X	○	○	○
Ich bin oft traurig	○	○	○	X	○

Copyright © 2025 by Julia Remmele

In den letzten Wochen hat mich mein Herz in meiner Selbstständigkeit beeinflusst

**Welche Aussage trifft auf Dich zu?**	trifft gar nicht zu	trifft nicht zu	weiß nicht	trifft zu	trifft voll zu
Ich konnte an Aktivitäten mit Freunden teilnehmen.	○	○	○	○	○

**Welche Aussage trifft auf Dich zu?**	trifft voll zu	trifft zu	weiß nicht	trifft nicht zu	trifft gar nicht zu
Ich fühlte mich durch meinen Herzfehler in meiner Selbstständigkeit eingeschränkt.	○	○	○	○	○

In den letzten Wochen hat mich mein Herz in der Schule / Ausbildung beeinflusst

**Welche Aussage trifft auf Dich zu?**	trifft gar nicht zu	trifft nicht zu	weiß nicht	trifft zu	trifft voll zu
Ich bin mit dem Unterrichtsstoff gut mitgekommen.	○	○	○	○	○

In den letzten Wochen hat mich mein Herz körperlich eingeschränkt

**Welche Aussage trifft auf Dich zu?**	trifft gar nicht zu	trifft nicht zu	weiß nicht	trifft zu	trifft voll zu
Ich fühlte mich körperlich frisch und munter.	○	○	○	○	○

Wie sieht es mit der Erholung von Dir und Deinem Herz in den letzten Wochen aus?

**Welche Aussage trifft auf Dich zu?**	trifft gar nicht zu	trifft nicht zu	weiß nicht	trifft zu	trifft voll zu
Ich habe mich gut nach anstrengenden Tagen erholt.	○	○	○	○	○

Du und Dein Herz allgemein

**Welche Aussage trifft auf Dich zu?**	trifft voll zu	trifft zu	weiß nicht	trifft nicht zu	trifft gar nicht zu
Ich finde Arztbesuche unangenehm.	○	○	○	○	○

Copyright © 2025 by J. Remmele & P. C. Helm
