# Supplementary material for: Development and Validation of the CHDSI Questionnaire: A New Tool for Measuring Disease-Specific Quality of Life in Children and Adolescents with Congenital Heart Defects
Source: Medicina (Kaunas). 2025 Jul 21;61(7):1311. doi: 10.3390/medicina61071311 (PMC12297989; doi:10.3390/medicina61071311)
Supplement: Supplementary file 1 [file medicina-61-01311-s001.zip › 12. Supplemental Material 3 - CHDSI Manual and Interpretation Guide.pdf]

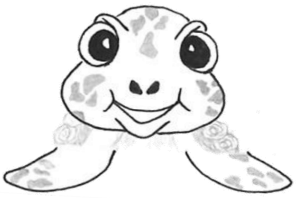

CHDSI

HANDBUCH & AUSWERTUNGSLEITFADEN

__C__ongenital __H__eart __D__isease __S__pecific __I__nventory

Krankheitsspezifische Lebensqualität von Kindern und Jugendlichen mit angeborenem Herzfehler

Paul C. Helm, Dr. rer. medic., Dipl.-Psych.

und

Julia Remmele, Dr. phil., M.Sc.

Copyright ©2025 by Julia Remmele, Dr. phil., M.Sc. & Paul C. Helm, Dr. rer. medic., Dipl.-Psych.

All rights reserved. No part of this manual covered by the copyrights hereon may be reproduced or transmitted in any form or by any means — electronic, mechanical, induding photocopy, recording, or any information storage or retrieval system — without permission of the copyright holder.

Requests for permission to reproduce or quote materials contained in this manual should be sent to Julia Remmele, Dr. phil., M.Sc., juliaxre@gmail.com. Reproduction of sample CHDSI items or specific normative data from this manual for purposes of documenting or interpreting a specific published study do not require permission, with proper citation of this manual as the source.

INHALTSVERZEICHNIS

1. EINLEITUNG .... *Seite 3*
2. HINTERGRUND .... *Seite 4*
3. ENTWICKLUNG DES CHDSI .... *Seite 8*
4. ANWENDUNGSMÖGLICHKEITEN DES CHDSI .... *Seite 25*
5. DATENANALYSE UND STATISTIK .... *Seite 26*
6. MODELLPASSUNG .... *Seite 31*
7. EINZELITEMS IM ÜBERBLICK .... *Seite 33*
8. SKALENPRÜFUNG .... *Seite 43*
9. MODELLPRÜFUNG .... *Seite 54*
10. NORMWERTE FÜR GESAMTSCORE UND SUBSKALEN .... *Seite 60*
11. KURZVERSION DES CHDSI .... *Seite 66*
12. ZUSAMMENFASSUNG, LIMITATIONEN UND AUSBLICK .... *Seite 69*
13. LITERATURVERZEICHNIS .... Seite 71
14. APPENDIX A: CHDSI Kinder (Langversion)
15. APPENDIX B: CHDSI Kinder (Kurzversion)
16. APPENDIX C: CHDSI Jugendliche (Langversion)
17. APPENDIX D: CHDSI Jugendliche (Kurzversion)

Vorbemerkung

Aus Gründen der besseren Lesbarkeit wird im Folgenden das generische Maskulinum verwendet. An dieser Stelle soll deutlich darauf hingewiesen werden, dass diese Form geschlechtsunabhängig zu verstehen ist.

1. EINLEITUNG

Das vorliegende Handbuch soll einen einführenden Überblick über den Stand der Forschung auf dem Gebiet der Lebensqualität (QoL) bei Patienten mit angeborenem Herzfehler (AHF) bieten, Anwendungsmöglichkeiten des *„Congenital Heart Disease Specific Inventory“* (CHDSI) beschreiben und über die Bedeutung der Einhaltung von Inhalts- und Bewertungsstandards als Grundlage für eine zuverlässige und gültige Interpretation der Skalenwerte informieren. Weiterhin wird über den Hintergrund und die Entwicklung des CHDSI berichtet.

Der CHDSI wurde so konzipiert, dass er sowohl selbst als auch in einem persönlichen Gespräch oder am Telefon ausgefüllt werden kann. Hierbei ist darauf hinzuweisen, dass der CHDSI nicht stellvertretend für das Kind oder den Jugendlichen mit AHF beantwortet werden sollte. Ein gemeinsames Ausfüllen beispielsweise mit den Eltern ist möglich, es sollte aber unbedingt darauf geachtet werden, das Kind bzw. den Jugendlichen nicht in seinen/ihren Antworten zu beeinflussen. Weicht die im Fragebogen gemachte Geschlechtsangabe von der des biologischen Geschlechts ab, ist bei einer geschlechtsspezifischen Auswertung das biologische Geschlecht zu verwenden. Ist das biologische Geschlecht zum Auswertungszeitpunkt nicht bekannt, kann für Patienten, die sich selbst als „männlich“ bzw. „weiblich“ einstufen auf die geschlechtsspezifischen Normwerte zurückgegriffen werden. Bei Patienten, die bei der Frage nach ihrem Geschlecht „divers“ auswählen, empfehlen wir die Verwendung der geschlechtsunspezifischen Allgemeinnormen.

Wir beschreiben die Anwendung psychometrischer Theorien und Methoden auf die Konstruktion und Prüfung der CHDSI-Skalen. Tests der internen Konsistenz der Items, Reliabilität und der Validität werden vor dem Hintergrund anderer veröffentlichter Ergebnisse zum Thema Fragebogenkonstruktion und unter Berücksichtigung weiterer Indikatoren für die Sicherung einer hohen Datenqualität zusammengefasst und beschrieben. Zusätzlich werden Empfehlungen für die Interpretation individueller Patientenwerte und Vorschläge zu erforderlichen Stichprobengrößen gemacht, die notwendig sind, um eine statistische Aussagekraft unter Verwendung des CHDSI beispielsweise in der Forschung zu erreichen.

Das Handbuch erläutert die Methoden der Skalenvalidierung und fasst verschiedene Validierungsstrategien zusammen. Wir stellen Leitlinien für die Interpretation von Unterschieden in den CHDSI-Subskalen vor und bieten Tabellen mit CHDSI-Normen, die mittels einer repräsentativen Stichprobe deutscher Kinder und Jugendlicher mit AHF erhoben wurden. Die Tabellen enthalten Normen und deskriptive Daten für zwei Altersgruppen, männliche und weibliche Patienten sowie klinische Populationen (simple, moderate und komplexe AHF).

Die Verwendung und Interpretation des CHDSI wird für verschiedene Anwendungen beispielhaft vorgestellt einschließlich der Möglichkeiten für die Implementierung des CHDSI in die klinische Praxis.

2. HINTERGRUND

In den letzten Jahrzehnten war eine der wichtigsten Entwicklungen im Gesundheitswesen die zunehmende Berücksichtigung und Anerkennung der Patientenperspektive bei der medizinischen Versorgung[1]. Die Relevanz der Wahrnehmung und Beurteilung der medizinischen Behandlung durch den Patienten wurde in der Fachliteratur schon zu Beginn des zwanzigsten Jahrhunderts beschrieben[2,3]. Zwar wurde die medizinische Behandlung bereits häufig und gut erbracht, doch gleichzeitig wuchs der Gedanke, dass nicht allein die medizinische Behandlung eine wichtige Rolle für eine gute Gesundheit spielt[3]. Fragen danach, ob das Behandlungsergebnis den grundlegenden Zielen der Lebensverlängerung, der Linderung von Leiden, der Wiederherstellung von Funktionen und der Verhinderung von Behinderungen entspricht[3] rückten zunehmend in den Fokus. Vielfach wurde bereits darauf hingewiesen, dass das Ziel der medizinischen Versorgung für die meisten Patienten die Erreichung einer höheren Lebensqualität[4] und die Erhaltung von Funktion und Wohlbefinden ist[5-9], doch noch immer werden Patienten nicht routinemäßig zu ihren Erfahrungen, Sorgen und Wünschen bezüglich ihrer medizinischen Behandlung befragt. Noch zu oft ist die Patientenperspektive kein obligatorischer Bestandteil von Krankenakten und steht somit häufig nicht für die Erstellung von Behandlungsplänen zur Verfügung. Aber nicht nur in der klinischen Praxis sind diese Informationen relevant, auch in der Forschung kann die Patientenperspektive wichtige Hinweise für eine erfolgreiche Behandlung geben.

Informationen über die Patientenperspektive werden zunehmend mit standardisierten Patientenbefragungen erhoben. Patientenstatus, Wohlbefinden und andere wichtige Aspekte der Gesundheitsversorgung können heutzutage gut mittels in der Forschung entwickelter Fragebögen erfasst werden. Die verfügbaren Daten verbessern das Verständnis der wichtigsten Gesundheitsdimensionen und die Validität spezifischer Messskalen in Bezug auf diese Dimensionen[10-12]. Zudem konnte der Nutzen standardisierter Gesundheitsumfragen in klinischen Studien nachgewiesen werden[13-15].

<u>Lebensqualität allgemein</u>

Der Begriff Lebensqualität (Quality of Life = QoL) beschreibt ein sehr komplexes, vielschichtiges und multidimensionales Konstrukt des menschlichen Seins[16] und ist nicht nur ein wichtiges Forschungsgebiet in der Medizin, sondern wird auch in der Psychologie, Soziologie und Wirtschaft untersucht. Das Interesse an der QoL begann im Zusammenhang mit Sozialberichten[17,18]. Es liegt keine einheitliche Definition von QoL vor. Mehrez und Gafni haben Lebensdauer, Lebenserwartung und Sterblichkeit als Merkmale der QoL definiert, während Morbidität und Gesundheitszustand als Parameter oder Indikatoren für die QoL verstanden wurden[19]. Andererseits wird QoL oft durch physische, psychische und soziale Aspekte definiert, wobei die Gesundheit selbst nur einen Teil der QoL darstellt[20].

Die Frage, ob medizinische Behandlungen zu einer besseren QoL oder sogar zu einem längeren Leben führen ist nicht neu[21]. Der Weltgesundheitsorganisation (WHO) zufolge lässt sich das Konstrukt der QoL nicht auf gesundheitliche Aspekte allein reduzieren. In ihrer Definition versteht die WHO Gesundheit als einen Zustand vollständigen körperlichen, geistigen und sozialen Wohlbefindens. Das Freisein von Krankheit oder Gebrechen allein ist nicht ausreichend[22].

Die Definition der menschlichen Gesundheit an sich umfasst verschiedene Konstrukte, die als Grundelemente einer guten und gesunden Lebensweise bezeichnet werden können[16]. Die Konzepte Gesundheit und QoL umfassen teils ähnliche, manchmal auch kongruente Dimensionen, die sich gegenseitig beeinflussen. Es besteht allerdings kein wissenschaftlicher Konsens bei der Definition von

QoL. Gleichwohl gibt es verschiedene Konstruktbeschreibungen und Instrumente zur Erfassung und zum Vergleich der individuellen QoL.

Eines der ersten Instrumente zur Messung der QoL wurde von Spitzer entwickelt (Spitzer's Quality of Life Index, QL-Index). Es umfasst die Dimensionen Aktivität, Selbstversorgung, Gesundheitszustand, soziale Unterstützung und Lebensausblick[23].

Die QoL hat vor allem im Kindesalter an Bedeutung gewonnen. Der KINDL®-Fragebogen wurde zur Messung der QoL und auch der gesundheitsbezogenen Lebensqualität (Health-related Qoality of Life = HrQoL) für den Einsatz bei erkrankten und gesunden Kindern und Jugendlichen entwickelt und 1998 gemeinsam von Ravens-Sieberer und Bullinger überarbeitet[24,25]. Das Ziel war die Erstellung eines kurzen, methodisch angemessenen und flexiblen Messinstruments. Es wurden zwei Hauptfragebögen entwickelt. Der eine erfasst die selbstberichtete HrQoL aus der Sicht der Kinder, der andere erhebt eine Fremdeinschätzung der HrQoL aus Elternsicht. Es gibt unter anderem eine Kurzform des KINDL® und eine Reihe krankheitsspezifischer Teile für Patienten mit Diabetes, Asthma, Neurodermitis und Adipositas. Die KINDL®-Fragebögen stehen in vielen Sprachen zur Verfügung: Englisch, Spanisch, Türkisch, Russisch, Niederländisch, Französisch und Italienisch.

Auf der Suche nach der treffenden Definition für QoL bzw. HrQoL wurden viele verschiedene Ansätze entwickelt. In einigen dieser Definitionen wird HrQoL als Teilmenge von QoL begriffen oder als aus der Perspektive des Individuums zu betrachtendes Konstrukt verstanden, was gemeinhin als subjektive QoL oder subjektives Wohlbefinden bezeichnet wird[26].

Unabhängig von der zugrundeliegenden Definition beeinflussen viele Faktoren das persönliche Empfinden von QoL, wobei die individuelle Lebenssituation eine wichtige Rolle spielt. Das Gleiche gilt für soziale oder gesellschaftliche, kulturelle und religiöse Faktoren, die unsere Wahrnehmung prägen und unsere Wertvorstellungen beeinflussen. So ist beispielsweise bekannt, dass der wirtschaftliche Status ebenso wie die Arbeits- und Familienverhältnisse Auswirkungen auf die QoL haben[27]. Es stellt sich also die Frage, ob die QoL verschiedener Individuen überhaupt vergleichbar ist, oder ob QoL immer nur bei einer Person zu verschiedenen Zeiten gemessen werden kann[28].

Um den dargestellten Herausforderungen zu begegnen, wurden forschungsbasierte Parameter für die QoL definiert, die im amerikanischen SF-36 Health Survey enthalten sind. Der SF-36 kann aufgrund seiner psychometrischen Qualität als ein international führendes Instrument angesehen werden[29] und stellt eine Art Konsens im Forschungsbereich dar. Diese Übereinkunft führte gleichwohl nicht zu einer Abgrenzung der Begriffe QoL und HrQoL. Sie werden in der wissenschaftlichen Literatur oftmals synonym verwendet.

Wir folgen der Argumentation, dass HrQoL als Teil von QoL zu begreifen ist und aus der Perspektive des Individuums zu betrachtendes Konstrukt verstanden werden sollte.

Lebensqualität und angeborene Herzfehler

Mit einer Prävalenz von etwa 1 % sind AHF die häufigste angeborene Erkrankung beim Menschen[30] und gehen mit einer erhöhten Morbidität und Mortalität einher[31,32]. In Deutschland werden jedes Jahr etwa 6.000 Kinder mit einem AHF geboren[33,34]. Heutzutage erreichen mehr als 90 % der betroffenen Patienten das Erwachsenenalter[31,34-41]. Die drastisch gesenkte Morbidität und die deutliche Erhöhung der Lebenserwartung sind primär auf den medizinischen Fortschritt in den Bereichen der pränatalen Diagnostik, der Kinderkardiologie und der Herzchirurgie zurückzuführen[31,42,43]. Folglich ist auch die wachsende Zahl an jugendlichen und erwachsenen

Patienten mit AHF vor allem auf die konstante Weiterentwicklung und Optimierung von Diagnostik und Behandlung zurückzuführen[36-38].

Die Überlebensrate für Neugeborene, Säuglinge und Kinder mit AHF hat sich signifikant verbessert[31,39-41]. Die kontinuierliche Verbesserung der medizinischen Behandlung und Diagnostik führt entsprechend zu einer Reduktion der Mortalität bei gleichbleibender Gesamtprävalenz. Die dadurch wachsende Zahl der Menschen mit AHF führt zu neuen Herausforderungen für die wissenschaftliche und die klinische Praxis. Entwicklungsfaktoren und die QoL rücken zunehmend in den Fokus.

Studien zeigten eine beeinträchtigte QoL von Kindern und Jugendlichen mit AHF im Vergleich zu gesunden Kontrollpersonen[44,45]. Drakouli et al. beschrieben in einer systematischen Übersichtsarbeit eine verminderte QoL in Bezug auf körperliche, psychosoziale, emotionale und schulische Aspekte bei Kindern mit AHF[46]. Andererseits gibt es Studien, die mittels Selbstauskunft der Kinder mit AHF eine gute HrQoL berichten[44,47,48].

Die unterschiedlichen Studienergebnisse auf dem Gebiet der HrQoL von Kindern und Jugendlichen mit AHF könnten mit der Situation und dem Zeitpunkt der Datenerfassung erklärt werden. Nach Müller et al. macht es beispielsweise einen großen Unterschied, ob die Fragebögen während eines Krankenhausaufenthalts oder im Rahmen einer Routineuntersuchung ausgefüllt werden. So wiesen Jugendliche und Erwachsene im Vergleich zu gesunden Kontrollpersonen während des Krankenhausaufenthalts eine erhöhte Ängstlichkeit auf[49], was wiederum zu unterschiedlichen Ergebnissen bei der Messung der HrQoL führen kann. Da in den meisten Studien zur Untersuchung der QoL bei Kindern unterschiedliche Messinstrumente verwendet wurden, sind die Ergebnisse nicht ohne weiteres vergleichbar. Hinzu kommen die unterschiedlichen Umgebungsbedingungen, die sich auf die QoL auswirken.

Validierte, normierte und in der klinischen Praxis etablierte Fragebögen zur Erfassung der krankheitsspezifischen Lebensqualität (Disease specific Quality of Life = DsQoL) und potentiell krankheitsspezifischer Einschränkungen für Kinder und Jugendliche mit AHF gibt es derzeit nach unserer Kenntnis nicht frei verfügbar in deutscher Sprache.

> Die DsQoL ist nach unserer Auffassung – ähnlich wie die HrQoL – als Teil der QoL zu verstehen. Im Vergleich mit der HrQoL fokussiert die DsQoL nicht primär auf allgemeine gesundheitsbezogene Aspekte der QoL sondern nimmt zusätzlich auch auf krankheitsspezifische Themen einer bestimmten Patientengruppe Rücksicht, die aufgrund ihrer Erkrankung unter anderem mit besonderen Herausforderungen, Problemen und Sorgen konfrontiert ist.

Die zunehmende Bedeutung der QoL bei Patienten mit AHF wird durch Studien untermauert[50-52]. Oftmals ist eine hohe QoL im weiteren Leben der jungen Patienten mit einer guten Bildung und einer aktiven Teilhabe am Arbeitsleben assoziiert[53,54]. Dies ist besonders für chronisch kranke Menschen sehr wichtig, zu denen auch Patienten mit AHF gehören. In der medizinischen Praxis spielen das Verständnis der DsQoL und die individuellen Patientenbedürfnisse eine immer wichtigere Rolle und sollten auch bei der Erstellung von Behandlungsplänen sowie der Planung von Untersuchungen und Interventionen/Operationen berücksichtigt werden[50,51,55].

Neben Informationen über den medizinischen Zustand und mögliche Risiken sowie über den prognostizierten weiteren Krankheitsverlauf ist es unerlässlich, die Patienten über mögliche Auswirkungen des AHF auf andere Aspekte des Lebens zu beraten und ggf. vorhandene Wissenslücken zu schließen[52]. Dies erfordert ein umfassendes Verständnis der typischen Lebenssituation von Kindern und Jugendlichen mit AHF und vor allem von Lebensfaktoren, die für diese jungen Patienten von besonderer persönlicher Bedeutung sind.

Nur mit einem validierten und normierten Messinstrument zur Erfassung der DsQoL kann die individuelle subjektive DsQoL zuverlässig erhoben werden. Ziel soll es sein ein Messinstrument zur Verfügung zu stellen, um unter Berücksichtigung der DsQoL ein umfassendes Behandlungskonzept erarbeiten zu können. Erst die Erfassung und Auseinandersetzung mit der individuellen subjektiven DsQoL von Kindern und Jugendlichen mit AHF im medizinischen Versorgungsprozess ermöglicht es, für den jeweiligen Patienten die bestmögliche allgemeine, gesundheitsbezogene und DsQoL zu erreichen.

3. ENTWICKLUNG DES CHDSI

Neben der QoL und der HrQoL ist auch die DsQoL– sowohl in der klinischen Praxis als auch in der medizinischen Forschung – ein wichtiger Faktor. Gleichzeitig sind die derzeit zur Verfügung stehenden Fragebögen zur Messung der QoL sehr allgemein gehalten. Mit ihnen lassen sich beispielsweise Vergleiche zwischen der QoL von Patienten mit AHF und der QoL der Allgemeinbevölkerung anstellen, was jedoch die Frage nach krankheisspezifischen Aspekten außer Acht lässt. Um diese Lücke zu schließen, wurde der CHDSI zur Erfassung der DsQoL von Kindern und Jugendlichen mit AHF entwickelt und kann sowohl in der klinischen Praxis als auch in wissenschaftlichen Kontexten Anwendung finden.

Eine erste Version des CHDSI wurde nach der Erstellung eines vorläufigen Fragebogenentwurfs anhand von Kindern und Jugendlichen mit AHF im Rahmen von Untersuchungen am Deutschen Herzzentrum München (DHM) pilotiert, mit den teilnehmenden Patienten diskutiert und anhand der gewonnenen Erkenntnisse optimiert. Die deutschlandweite Validierung des entwickelten Fragebogens mit abschließender onlinebasierter Validierung und Normierung erfolgte in Zusammenarbeit mit dem Nationalen Register für angeborene Herzfehler e. V. (NRAHF).

Kernziele der durchgeführten Studie:

1. Die Analyse der DsQoL von Kindern und Jugendlichen mit AHF in Abhängigkeit von Alter, biologischem Geschlecht und Schweregrad des AHF (Validierung und Normierung des Messinstruments)
2. Die Bereitstellung der Forschungsergebnisse sowie des validierten und normierten Fragebogens für zukünftige Forschungsprojekte, Interventionsprogramme und den klinischen Alltag.

Auf Grundlage der Vorarbeiten und in engem Austausch mit betroffenen Kindern und Jugendlichen mit AHF im Alter zwischen 6 und < 18 Jahren wurden zwei finale Fragebogenversionen (jeweils für Kinder und Jugendliche) entwickelt. Hierbei wurde ein aus 6 latenten Variablen bestehendes Konstrukt der DsQoL postuliert, welches bei Kindern (Schulkinder; 6 bis < 14 Jahre) mit 36 und bei Jugendlichen (14 bis < 18 Jahre) mit 37 manifesten Items erfasst werden soll. Ist ein Kind zwar schon sechs Jahre alt, geht aber noch nicht zur Schule, wurde ein reduziertes Konstrukt der DsQoL (5 latente Variablen) aus insgesamt 31 der 36 Items des Kinderfragebogens vorgeschlagen (vgl. Tabelle 1).

Es wurden weder Haupt- noch Sekundärhypothesen formuliert, sondern vielmehr die Passung des vorgeschlagenen Modells unter anderem mittels Konfirmatorischer Faktorenanalyse (CFA) überprüft.

Erste Entwicklungsschritte

Eine hohe QoL ist ein zentraler Baustein für eine altersentsprechende körperliche und geistige Entwicklung. Wir vertreten die Ansicht, dass bereits im Kindesalter die DsQoL beispielsweise bei der Erstellung von Behandlungsplänen oder der Planung von Untersuchungen und Interventionen/Operationen berücksichtigt werden sollte, um bei potentiellen Auffälligkeiten bis hin zu manifesten Einschränkungen bereits frühzeitig intervenieren zu können. Der CHDSI soll die Erfassung der DsQoL unter Berücksichtigung typischer Lebenssituationen von Kindern und Jugendlichen mit AHF unter Einbeziehung von allgemeinen und krankheitsspezifischen Lebensfaktoren ermöglichen, die für die jungen Patienten mit AHF von besonderer persönlicher Bedeutung sind.

Die Erstellung der ersten Version des CHDSI erfolgte am DHM. Entwickelt wurde eine Kinderversion (6 bis < 12 Jahre) und eine Jugendversion (12 bis < 18 Jahre). Beide Fragebogenversionen wurden in jeweils zwei Etappen erarbeitet, die erhobenen Daten wurden kontinuierlich analysiert und die Fragebögen wurden in engem Austausch mit den Patienten optimiert.

Die Entscheidung, zwei Fragebögen zu erstellen fußte auf der Erfahrung, dass einige Fragen für bestimmte Altersgruppen noch nicht bzw. nicht mehr zutreffen. Es wurde darauf geachtet, dass so viele Fragen wie möglich gleich lauten und auch die Anzahl der Fragenkomplexe identisch ist. Insgesamt bestanden die beiden ersten Fragebogenversionen aus 7 Fragenkomplexen mit jeweils 6 Items sowie einem einleitenden Abschnitt zur Erhebung soziodemographischer Daten. Die 7 Fragenkomplexe umfassten krankheits- und lebensbezogene Aspekte mit verschiedenen Schwerpunkten wie z. B. Schule, Familie, Freizeit/Freunde, Krankheit/Krankheitssymptome, Erfahrungen in der klinischen Behandlung und medizinischen Versorgung sowie Erholungsfähigkeit, immer im konkreten Kontext zum AHF. Nach jedem Fragenkomplex stand ein Freitextfeld zur Verfügung, um weitere möglicherweise relevante Themen der Kinder und Jugendlichen zu erfassen.

In der ersten Etappe wurden 40 Kinder und 40 Jugendliche in der Ambulanz des DHM angesprochen, um an der ersten Pretest-Phase des CHDSI teilzunehmen. Hierbei wurde den Probanden ein Papier-Bleistift-Fragebogen übergeben und um das Ausfüllen gebeten, nachdem sie sich eingehend mit der Studieninformation befasst hatten, Rückfragen geklärt wurden sowie eine schriftliche Einwilligung von den Kindern und ihren Eltern erbracht war. Anschließend wurden die Fragebögen in eine Onlinedatenbank überführt und von dort als SPSS-Datei zur weiteren Auswertung und Verarbeitung exportiert.

Anhand von ersten vorläufigen statistischen Itemanalysen, inhaltlicher Überlegungen und ständigem Austausch mit den befragten Kindern und Jugendlichen wurden Items gestrichen, angepasst oder ergänzt. Die nach jedem Fragenkomplex zur Verfügung gestellten Freitextfelder wurden grundsätzlich positiv wahrgenommen, gemeinsam mit den Kindern und Jugendlichen wurde jedoch entschieden, die Freitextfelder auf ein Freitextfeld am Ende der Fragebögen zu reduzieren. Eine komplette Entfernung von Freitextfeldern kam nicht in Frage, da einige Kinder und Jugendliche hier noch verschiedenste, sehr individuelle Problematiken thematisieren können und diese Gelegenheit auch nutzen.

In der zweiten Phase erhielten 32 Kinder und 41 jugendliche Patienten in der Ambulanz des DHM die überarbeiteten Fragebogenversionen. Nach den Auswertungen und abschließenden Gesprächen mit den Befragten lagen zwei Fragebögen bestehend aus jeweils 43 Items in 7 Fragekomplexen vor, wobei die Fragebogenversionen bezüglich der Altersspanne auf 6 bis 13,9 Jahre bzw. 14 bis 17,9 Jahre angepasst wurden. Bei Kindergartenkindern sollte stets nur ein Teil der Kinderfragen zur Bewertung der DsQoL berücksichtigt werden (vgl. Tabelle 1). Um die Ergebnisse der durchgeführten

Pretests zu bestätigen, wurde nun die Validierung und Normierung des CHDSI anhand einer deutschlandweiten repräsentativen Stichprobe durchgeführt.

Validierungs- und Normierungsprozess

Die Studienteilnehmer wurden mit der Online-Version des im DHM in Zusammenarbeit mit dem NRAHF entwickelten CHDSI befragt.

Das NRAHF wurde für die Durchführung derartiger Forschungsprojekte von den drei Fachgesellschaften *Deutsche Gesellschaft für Kardiologie, Herz- und Kreislaufforschung* (DGK), *Deutsche Gesellschaft für pädiatrische Kardiologie* (DGPK) und *Deutsche Gesellschaft für Thorax-, Herz- und Gefäßchirurgie* (DGTHG) 2003 als gemeinnütziger und wissenschaftlicher Verein etabliert. Die Vielzahl der registrierten Patienten kann als repräsentativ für die in Deutschland lebenden Patienten mit (klinisch apparenten) AHF gelten[56] und ermöglicht eine gezielte Kohortenbildung. Die Basisdaten können aufgrund des vorhandenen Ethikvotums des durchgeführten Forschungsprojekts (EA2/253/21, Ethikkommission Charité Berlin) und der Einverständniserklärung der Patienten/Erziehungsberechtigten um studienspezifische Parameter ergänzt werden. Dem NRAHF liegt zusätzlich ein Datenschutzkonzept zugrunde, das die Voraussetzungen für die erforderlichen Abfragen personenbezogener und medizinischer Daten bietet.

Auf der Basis der vorhandenen IT/Datenbank-Infrastruktur des NRAHF waren folgende Punkte zentraler Bestandteil des Datenschutzes:

- Pseudonymisierungsdienst (PSD) als zentrales Element für das Identitätsmanagement
- Datenbank zur Speicherung und Verwaltung personenidentifizierender Daten (IDAT)
- Datenbank zur Speicherung und Verwaltung medizinischer Daten der Teilnehmer (MDAT)

Das NRAHF erhebt die Daten pseudonymisiert-personenbezogen. Bei der Erfassung der personenidentifizierenden Informationen (Name, Geburtsname, Geburtsdatum, Geschlecht, Adresse) wird mittels eines Pseudonymisierungsdienstes (PID-Generator)[57] jedem Patienten ein Pseudonym (PID, 8-stelliger alphanumerischer Code) zugewiesen, der für die interne Abfrage der medizinischen Daten erforderlich und zur Sicherung des Datenschutzes nicht identisch mit der neunstelligen Studiennummer ist.

Zur Datenbank IDAT mit personenidentifizierenden Informationen, die z. B. zur Kontaktaufnahme genutzt wird, haben nur bestimmte NRAHF-Mitarbeiter Zugriffsrechte. Die medizinischen Daten (MDAT) werden getrennt von den personenidentifizierenden Daten in der Datenbank unter dem jeweiligen Pseudonym gespeichert. Die durchgeführten Auswertungen erfolgten entsprechend anhand der pseudonymisierten Daten ohne Personenbezug. Verantwortlich für die Datenhaltung im Sinne der DSGVO ist das NRAHF. Die Erfassung, Speicherung, Verarbeitung und Auswertung aller Daten erfolgt im Rahmen des Datenschutzkonzeptes des NRAHF (beim Berliner Beauftragten für Datenschutz und Informationsfreiheit registriert unter der Nr. 531.390). Sämtliche Mitarbeiter des NRAHF sind vertraglich zur Verschwiegenheit verpflichtet. Mit den Kooperationspartnern bestehen Verträge zur Auftragsdatenverarbeitung nach BDSG §11 bzw. DSGVO Art. 28.

Da die Studienteilnahme mittels eines neunstelligen individuellen studienspezifischen Teilnehmercodes (pseudonymisiert) erfolgte, konnten von den teilnehmenden Patienten einerseits die Angaben der Onlinebefragung und andererseits die medizinischen Behandlungsdaten erhoben werden. An dieser Stelle ist es wichtig darauf hinzuweisen, dass nicht von allen im NRAHF registrierten Patienten detaillierte medizinische Informationen vorliegen. Für die Erfassung von personenbezogenen medizinischen Daten ist die Entbindung von der Schweigepflicht der

behandelnden Ärzte durch den Patienten bzw. dessen Eltern erforderlich, um beispielsweise Arztbriefe anfordern zu können. Liegt eine solche Schweigepflichtentbindung nicht vor, werden die Patienten um die Zusendung eines aktuellen Arztbriefs gebeten. Dies ist absolut freiwillig und eine Nichtzusendung ist mit keinerlei Nachteilen für den registrierten Patienten verbunden. Folglich können auch Patienten an Befragungen teilnehmen, von denen lediglich bekannt ist, dass sie einen AHF haben. Von den 1.201 Patienten, die den CHDSI komplett ausgefüllt haben und die in die nachfolgenden statistischen Auswertungen einbezogen werden trifft dies in 110 Fällen (9,2 %) zu. Bei den Berechnungen der Normwerte in Abhängigkeit zum AHF-Schweregrad werden diese 110 Fälle entsprechend nicht berücksichtigt.

Das NRAHF als größtes AHF-Register Europas[56] umfasste zu Studienbeginn rund 55.000 Patienten mit AHF aller Schweregrade. Der multizentrische Forschungsansatz des NRAHF ermöglichte die deutschlandweite repräsentative onlinebasierte Datenerhebung und -auswertung zur Validierung und Normierung des CHDSI für Kinder und Jugendliche mit AHF. Die Projektkommunikation und -koordination wurde mit der etablierten Infrastruktur des NRAHF sichergestellt und das NRAHF stand allen Studienteilnehmern jederzeit für Fragen hinsichtlich der Studienteilnahme zur Verfügung. Alle Teilnehmer konnten jederzeit und ohne Angabe von Gründen die Löschung Ihrer abgegebenen Daten veranlassen.

Es bestand weder ein Risiko für die Studienteilnehmer, noch entstanden aus der Studienteilnahme Nachteile. Die einzige Belastung bestand im Zeitaufwand zum Ausfüllen des Fragebogens (ca. 15 Minuten). Es erfolgte keine Honorierung und die Online-Umfrage hat in keinerlei Weise in die Behandlung der Studienteilnehmer eingegriffen. Da die Befragung aus dem NRAHF heraus erfolgte, bestand kein Behandlungszusammenhang zwischen Studienleitung oder Mitgliedern des Forschungsteams und Patienten. Die Teilnahme war freiwillig und kostenlos. Als Einschlusskriterium galt das Vorliegen einer Post/E-Mail-Adresse zur Kontaktaufnahme sowie ein Alter des registrierten AHF-Patienten zwischen 6 bis < 18 Jahren. Nach Erhalt der Einladung konnten die Eingeladenen unmittelbar mit ihrer Umfrageteilnahme starten. Zum Befragungszeitpunkt waren 11.919 Kinder und Jugendliche mit AHF im Alter zwischen 6 und < 18 Jahren mit Kontaktdaten (E-Mail oder Postanschrift) registriert. Die Kinder/Jugendlichen wurden über die Erziehungsberechtigten kontaktiert und zur Teilnahme eingeladen. Das Ausfüllen des Online-Fragebogens galt als Einwilligung zur Studienteilnahme, da dieses das aktive Handeln der betreffenden Personen voraussetzt. Von den Eingeladenen konnten insgesamt 11.906 Patienten auch tatsächlich per Post oder E-Mail erreicht werden (99,9 %) und 2.083 nahmen an der Befragung teil (17,5 %), wobei 1.201 die Fragebögen auch vollständig beantworteten (10,1 %). Das Geschlechtsverhältnis der eingeladenen und erreichten Patienten war nahezu ausgeglichen (49,2 % weiblich) und entsprach in etwa dem der Allgemeinbevölkerung. Das Geschlechtsverhältnis der 1.201 Patienten, die alle Fragen beantworteten, war ebenfalls annähernd gleich (52 % weiblich). Die Studienteilnahme war ausschließlich auf elektronischem Weg möglich. Die teilnehmenden Patienten erhielten eine neunstellige Studiennummer und – je nach Alter – die Kinder- bzw. die Jugendversion des CHDSI in Form eines Onlinefragebogens zur Bearbeitung.

Mit diesem Handbuch werden die statistischen Analysen und Ergebnisse sowohl den Patienten als auch der Forschungscommunity zur Verfügung gestellt. Die Ergebnispräsentation erfolgt ausschließlich in anonymisierter Form. Ein Bezug zu Kontaktdaten der Eltern oder den Daten des Kindes/Jugendlichen kann nicht hergestellt werden.

Fragen, Fragenkomplexe und postulierte Skalen im Überblick

Der Fragebogen startet mit einer kurzen einleitenden Information, worum es in den nächsten ca. 15 Minuten geht. Auf den Einführungstext folgen insgesamt 11 Items zur Erfassung persönlicher demografischer Daten, eine Beispielfrage für die anschließenden Rating-Items sowie die 43 Rating-Items selbst, die auf insgesamt 7 Fragenkomplexe aufgeteilt sind (6 x 6 Items und 1 x 7 Items). Da einige Items beispielsweise sogenannte Single- bzw. Indikator-Items darstellen, die kein Bestandteil der postulierten Skalen sind und die konstruierten Skalen von verschieden vielen Items repräsentiert werden, entsprechen die 7 Fragenkomplexe des CHDSI nicht bzw. nur teilweise den sechs postulierten Faktoren/Dimensionen. Eine Auflistung der postulierten latenten Variablen sowie der dazugehörigen manifesten Items ist Tabelle 1 zu entnehmen. Von den 43 Items repräsentieren 31 (Kindergartenkinder), 36 (Schulkinder) bzw. 37 (Jugendliche) das Modell der DsQoL von jungen und jugendlichen Patienten mit AHF. Die 7 Items (Kinder) und die 6 Items (Jugendliche), sind als Single- bzw. Indikatoritems keiner der vorgeschlagenen Skalen zuzuordnen. Die konstruierten Skalen bestehen aus mindestens 2 und maximal 12 Items, wobei die postulierten Dimensionen der DsQoL bei Kindergartenkindern, Schulkindern und Jugendlichen zwar sehr ähnlich, allerdings nur in Teilen identisch sind, wie die nachfolgende übersichtsartige und vergleichende Darstellung der finalen Kinder- und Jugendversion des CHDSI zeigt.

Liebe Patientinnen und Patienten, liebe Eltern,
in diesem Fragebogen geht es um krankheitsspezifische Lebensqualität.

WICHTIGER HINWEIS
Zum Beantworten der Fragen ist es sehr wichtig, dass der Fragebogen von dem/der Patient/in *allein* ausgefüllt wird. Wenn dies nicht möglich ist, können die Fragen auch gemeinsam z. B. mit den Eltern beantwortet werden. Wird der Fragebogen zusammen mit den Eltern ausgefüllt, geht es ausschließlich um die Einschätzung des Kindes/Jugendlichen und nicht um die Meinung der Eltern. ***Ein Ausfüllen der Fragen ohne das Kind bzw. den Jugendlichen mit angeborenem Herzfehler (also stellvertretend) ist nicht möglich/sinnvoll.***
Da sich der Fragebogen also direkt an Kinder und Jugendliche mit angeborenem Herzfehler richtet, wird im Folgenden die Anrede „Du“ verwendet.

WAS WIRD GEFRAGT?
Die Fragen beschäftigen sich mit Deiner Lebenssituation und deiner Lebensqualität und wurden gemeinsam mit über 1.000 Kindern und Jugendlichen mit angeborenem Herzfehler erarbeitet. Die Beantwortung dauert etwa 10 bis 15 Minuten.

Wichtig ist, dass es keine „richtigen" oder „falschen" Antworten gibt. Versuche einfach, alle Fragen ehrlich zu beantworten und mach dir keine Gedanken, wenn Du bei einer Antwort unsicher bist. Wähle einfach immer die Antwort aus, die am besten zu Dir passt, denn Du selbst kannst Deine Gefühle und Gedanken am besten wahrnehmen und beurteilen.

Falls Du Fragen hast, zögere bitte nicht uns jederzeit anzusprechen.

Wir freuen uns auf Deine Teilnahme!

Wie alt bist Du genau?

Alter in Jahren: ___ ___

Welches Geschlecht hast Du?

□ männlich
□ weiblich
□ divers

Hast Du Geschwister? Wenn ja, wie alt sind sie und welches Geschlecht haben sie?
Wenn Du keine Geschwister hast, lass diese Felder einfach leer.

1. Geschwisterkind

Alter in Jahren: ___ ___
□ männlich
□ weiblich
□ divers

2. Geschwisterkind

Alter in Jahren: ___ ___
□ männlich
□ weiblich
□ divers

3. Geschwisterkind

Alter in Jahren: ___ ___
□ männlich
□ weiblich
□ divers

4. Geschwisterkind

Alter in Jahren: ___ ___
□ männlich
□ weiblich
□ divers

5. Geschwisterkind

Alter in Jahren: ___ ___
□ männlich
□ weiblich
□ divers

6. Geschwisterkind

Alter in Jahren: ___ ___
□ männlich
□ weiblich
□ divers

7. Geschwisterkind

Alter in Jahren: ___ ___
□ männlich
□ weiblich
□ divers

8. Geschwisterkind

Alter in Jahren: ___ ___
□ männlich
□ weiblich
□ divers

KINDER **Wie viele Jahre warst Du (bislang) im Kindergarten?**
Wenn Du noch in den Kindergarten gehst, gib bitte an seit wie vielen Jahren. Wenn du noch nie in den Kindergarten gegangen bist, lass dieses Feld einfach leer.

Anzahl der Kindergartenjahre: ___ ___

JUGENDLICHE **Wie viele Jahre warst Du im Kindergarten?**
Wenn du noch nie in den Kindergarten gegangen bist, lass dieses Feld einfach leer.

Anzahl der Kindergartenjahre: ___ ___

Auf was für eine Schule gehst Du im Moment?

KINDER

Beispiele: Grundschule, Integrationsschule, Förderschule, Hauptschule, Gymnasium...
Wenn Du noch nicht in die Schule gehst, schreib einfach, dass Du noch nicht zur Schule gehst.

Schulart: ___________________________________

JUGENDLICHE

Beispiele: Grundschule, Integrationsschule, Förderschule, Hauptschule, Gymnasium...
Wenn Du nicht (mehr) in die Schule gehst, schreib einfach, was Du stattdessen machst.

Schulart: ___________________________________

***Welchen höchsten allgemeinbildenden Schulabschluss hat Deine Mutter?**

□ Keinen Schulabschluss.
□ Hauptschulabschluss/Volksschulabschluss.
□ Realschulabschluss/Abschluss der polytechnischen Oberschule.
□ Fachabitur/Fachgebundene Hochschulreife.
□ Abitur/Allgemeine Hochschulreife.
□ Einen anderen Schulabschluss, und zwar: ___________________________________
□ Ich weiß es nicht.

***Welchen höchsten beruflichen Ausbildungsabschluss hat Deine Mutter?**

□ Keinen beruflichen Abschluss und ist nicht in einer beruflichen Ausbildung.
□ Noch in einer beruflichen oder universitären Ausbildung (Berufsvorbereitungsjahr, Auszubildender, Praktikant, Student).
□ Betriebliche Berufsausbildung (Lehre) abgeschlossen.
□ Ausbildung an einer Fach-, Meister-, Technikerschule, Berufs- oder Fachakademie abgeschlossen.
□ Bachelor an einer Fachhochschule oder an einer Universität.
□ Master/Diplom an einer Fachhochschule.
□ Master/Diplom/Magister/Staatsexamen/Promotion an einer Universität.
□ Einen anderen beruflichen Abschluss, und zwar: ___________________________________
□ Ich weiß es nicht.

***In welcher Erwerbssituation befindet sich Deine Mutter? Bitte beachte, dass unter Erwerbstätigkeit jede bezahlte bzw. mit einem Einkommen verbundene Tätigkeit verstanden wird.**

□ Vollzeiterwerbstätig.
□ Teilzeiterwerbstätig.
□ Geringfügig erwerbstätig, 450-Euro-Job, Minijob.
□ Nicht erwerbstätig.
□ Ich weiß es nicht.

**Dieselben Fragen werden auch zum Vater gestellt.*

KINDER **In den letzten Wochen hat mich mein Herz in meiner Selbstständigkeit beeinflusst**

Welche Aussage trifft auf Dich zu?

	**trifft voll zu**	**trifft zu**	**weiß nicht**	**trifft nicht zu**	**trifft gar nicht zu**
Ich fühlte mich im Vergleich mit Freunden gleich gut.	☐	☐	☐	☐	☐
Ich habe mich wohl gefühlt wie ich bin.	☐	☐	☐	☐	☐
Ich konnte an Aktivitäten mit Freunden teilnehmen.	☐	☐	☐	☐	☐
Ich habe mir Sorgen über meine Zukunft gemacht.	☐	☐	☐	☐	☐
Ich habe mich hilflos und traurig gefühlt.	☐	☐	☐	☐	☐
Ich hatte Schwierigkeiten Freunde zu finden.	☐	☐	☐	☐	☐

JUGENDLICHE **In den letzten Wochen hat mich mein Herz in meiner Selbstständigkeit beeinflusst**

Welche Aussage trifft auf Dich zu?

	**trifft voll zu**	**trifft zu**	**weiß nicht**	**trifft nicht zu**	**trifft gar nicht zu**
Ich fühlte mich genauso selbstständig wie meine Freunde.	☐	☐	☐	☐	☐
Ich hatte keine Schwierigkeiten Freunde zu finden.	☐	☐	☐	☐	☐
Ich konnte an Aktivitäten mit Freunden teilnehmen.	☐	☐	☐	☐	☐
Ich habe mich gefragt, ob ich mit meinem Herzfehler eine/n feste/n Freund/in finde.	☐	☐	☐	☐	☐
Ich habe mich hilflos und traurig gefühlt.	☐	☐	☐	☐	☐
Ich fühlte mich durch meinen Herzfehler in meiner Selbstständigkeit eingeschränkt.	☐	☐	☐	☐	☐

KINDER **Wegen meinem Herz haben sich in den letzten Wochen ...**
Welche Aussage trifft auf Dich zu? Wenn Du noch nicht in die Schule gehst, beziehen sich die Aussagen zur Schule natürlich auf die Erzieher und die anderen Kindergartenkinder.

		**trifft voll zu**	**trifft zu**	**weiß nicht**	**trifft nicht zu**	**trifft gar nicht zu**
... meine Eltern viele Sorgen gemacht, das tut mir leid.		☐	☐	☐	☐	☐
... in der Schule Lehrer und Mitschüler mir gegenüber komisch verhalten.		☐	☐	☐	☐	☐
... meine Eltern mir gegenüber anders verhalten als meinen Geschwistern gegenüber.	**habe keine** ☐	☐	☐	☐	☐	☐
... meine Eltern und andere Erwachsene übervorsichtig mir gegenüber verhalten.		☐	☐	☐	☐	☐
... Lehrer und Mitschüler besonders um mich bemüht, ohne dass ich das wollte.		☐	☐	☐	☐	☐
.. andere Kinder über mich lustig gemacht.		☐	☐	☐	☐	☐

JUGENDLICHE **Wegen meinem Herz haben sich in den letzten Wochen ...**
Welche Aussage trifft auf Dich zu?

		**trifft voll zu**	**trifft zu**	**weiß nicht**	**trifft nicht zu**	**trifft gar nicht zu**
... meine Eltern viele Sorgen gemacht, das tut mir leid.		☐	☐	☐	☐	☐
... in der Schule/Ausbildung Lehrer und Mitschüler mir gegenüber komisch verhalten.		☐	☐	☐	☐	☐
... meine Eltern mir gegenüber anders verhalten als meinen Geschwistern gegenüber.	**habe keine** ☐	☐	☐	☐	☐	☐
... meine Eltern und andere Erwachsene übervorsichtig mir gegenüber verhalten.		☐	☐	☐	☐	☐
... Lehrer und Mitschüler besonders um mich bemüht, ohne dass ich das wollte.		☐	☐	☐	☐	☐
... Andere über mich lustig gemacht.		☐	☐	☐	☐	☐

KINDER **In den letzten Wochen hat mich mein Herz in der Schule beeinflusst**
Welche Aussage trifft auf Dich zu?

	ich gehe (noch) nicht zur Schule	trifft voll zu	trifft zu	weiß nicht	trifft nicht zu	trifft gar nicht zu
Ich habe viel Unterricht verpasst wegen Untersuchungen.	☐	☐	☐	☐	☐	☐
Ich konnte im Sportunterricht nicht alles mitmachen.	☐	☐	☐	☐	☐	☐
Ich habe mir Gedanken gemacht, ob ich die Schule schaffe.	☐	☐	☐	☐	☐	☐
Ich bin mit dem Unterrichtsstoff gut mitgekommen.	☐	☐	☐	☐	☐	☐
Ich bin mit den Hausaufgaben gut zurechtgekommen.	☐	☐	☐	☐	☐	☐
Ich gehe grundsätzlich gerne zur Schule.	☐	☐	☐	☐	☐	☐

JUGENDLICHE **In den letzten Wochen hat mich mein Herz in der Schule/Ausbildung beeinflusst**
Welche Aussage trifft auf Dich zu?

	trifft voll zu	trifft zu	weiß nicht	trifft nicht zu	trifft gar nicht zu
Ich habe viel Unterricht verpasst wegen Untersuchungen.	☐	☐	☐	☐	☐
Ich war besorgt, welchen Beruf ich mit meinem Herzfehler machen kann.	☐	☐	☐	☐	☐
Ich habe mir Gedanken gemacht, ob ich die Schule/Ausbildung schaffe.	☐	☐	☐	☐	☐
Ich habe mir überhaupt keine Sorgen um meine Zukunft gemacht.	☐	☐	☐	☐	☐
Ich bin mit den Hausaufgaben gut zurechtgekommen.	☐	☐	☐	☐	☐
Ich bin mit dem Unterrichtsstoff gut mitgekommen.	☐	☐	☐	☐	☐

***In den letzten Wochen hat mich mein Herz körperlich eingeschränkt**
Welche Aussage trifft auf Dich zu?

	trifft voll zu	trifft zu	weiß nicht	trifft nicht zu	trifft gar nicht zu
Ich konnte alle körperlichen Aktivitäten machen wie ich wollte.	☐	☐	☐	☐	☐
Ich konnte alles machen ohne außer Puste zu geraten.	☐	☐	☐	☐	☐
Ich hatte keine körperlichen Schmerzen.	☐	☐	☐	☐	☐
Ich habe mich selten krank gefühlt.	☐	☐	☐	☐	☐
Ich konnte problemlos meine Hobbies machen.	☐	☐	☐	☐	☐
Ich fühlte mich körperlich frisch und munter.	☐	☐	☐	☐	☐

***In den letzten Wochen hatte ich wegen meinem Herz …**
Welche Aussage trifft auf Dich zu?

	trifft voll zu	trifft zu	weiß nicht	trifft nicht zu	trifft gar nicht zu
… schnell keine Puste mehr.	☐	☐	☐	☐	☐
... abends öfter dicke Beine und Füße.	☐	☐	☐	☐	☐
… häufig mit Schwindel zu tun.	☐	☐	☐	☐	☐
… auch Schmerzen in der Brust.	☐	☐	☐	☐	☐
… gemerkt, dass ich schnell müde werde.	☐	☐	☐	☐	☐
… öfter komisches Herzklopfen.	☐	☐	☐	☐	☐

**Dieselben Fragenkomplexe und Einzelitems sind bei Kindern und Jugendlichen identisch.*

***Wie sieht es mit der Erholung von Dir und Deinem Herz in den letzten Wochen aus?**

Welche Aussage trifft auf Dich zu?

	trifft voll zu	trifft zu	weiß nicht	trifft nicht zu	trifft gar nicht zu
Ich konnte nicht gut einschlafen.	☐	☐	☐	☐	☐
Ich bin nachts häufig aufgewacht.	☐	☐	☐	☐	☐
Ich bin morgens schwer aus dem Bett gekommen.	☐	☐	☐	☐	☐
Ich bin erholt aufgewacht.	☐	☐	☐	☐	☐
Ich hatte einen ruhigen festen Schlaf.	☐	☐	☐	☐	☐
Ich habe mich gut nach anstrengenden Tagen erholt.	☐	☐	☐	☐	☐

***Du und Dein Herz allgemein**

Welche Aussage trifft auf Dich zu?

		trifft voll zu	trifft zu	weiß nicht	trifft nicht zu	trifft gar nicht zu
Ich weiß gut Bescheid über mein Herz und was ihm fehlt.		☐	☐	☐	☐	☐
Vor dem Krankenhaus habe ich keine Angst.		☐	☐	☐	☐	☐
Ich vertrage meine Medikamente gut.	**nehme keine** ☐	☐	☐	☐	☐	☐
Ich finde Arztbesuche unangenehm.		☐	☐	☐	☐	☐
Ich mag es nicht am Herzen untersucht zu werden.		☐	☐	☐	☐	☐
Ich finde es nervig Medikamente zu nehmen.	**nehme keine** ☐	☐	☐	☐	☐	☐
Ich finde meine Operationsnarbe sehr unangenehm.	**habe keine** ☐	☐	☐	☐	☐	☐

**Dieselben Fragenkomplexe und Einzelitems sind bei Kindern und Jugendlichen identisch.*

Tabelle 1. *Postulierte Skalen/latente Variablen.*

Skala	Kinder	Jugendliche
**MeHeartImpairments**		
	Ich konnte alles machen ohne außer Puste zu geraten.	Ich konnte alles machen ohne außer Puste zu geraten.
	Ich hatte keine körperlichen Schmerzen.	Ich hatte keine körperlichen Schmerzen.
	Ich habe mich selten krank gefühlt.	Ich habe mich selten krank gefühlt.
	Ich konnte problemlos meine Hobbies machen.	Ich konnte problemlos meine Hobbies machen.
	Ich fühlte mich körperlich frisch und munter.	Ich fühlte mich körperlich frisch und munter.
	… schnell keine Puste mehr.	… schnell keine Puste mehr.
	… häufig mit Schwindel zu tun.	… häufig mit Schwindel zu tun.
	… auch Schmerzen in der Brust.	… auch Schmerzen in der Brust.
	… gemerkt, dass ich schnell müde werde.	… gemerkt, dass ich schnell müde werde.
	… öfter komisches Herzklopfen.	… öfter komisches Herzklopfen.
	Ich konnte alle körperlichen Aktivitäten machen wie ich wollte.	Ich konnte alle körperlichen Aktivitäten machen wie ich wollte.
**MeHeartStigma**		
	... meine Eltern viele Sorgen gemacht, das tut mir leid.	... meine Eltern viele Sorgen gemacht, das tut mir leid.
	*... in der Schule Lehrer und Mitschüler mir gegenüber komisch verhalten.	... in der Schule/Ausbildung Lehrer und Mitschüler mir gegenüber komisch verhalten.
	... meine Eltern und andere Erwachsene übervorsichtig mir gegenüber verhalten.	... meine Eltern und andere Erwachsene übervorsichtig mir gegenüber verhalten.
	*... Lehrer und Mitschüler besonders um mich bemüht, ohne dass ich das wollte.	... Lehrer und Mitschüler besonders um mich bemüht, ohne dass ich das wollte.
	... andere Kinder über mich lustig gemacht.	... Andere über mich lustig gemacht.
	**Ich habe viel Unterricht verpasst wegen Untersuchungen.	Ich habe viel Unterricht verpasst wegen Untersuchungen.
	Ich habe mich hilflos und traurig gefühlt.	Ich habe mich hilflos und traurig gefühlt.
	**Ich habe mir Gedanken gemacht, ob ich die Schule schaffe.	Ich habe mir Gedanken gemacht, ob ich die Schule/Ausbildung schaffe.
	Ich habe mir Sorgen über meine Zukunft gemacht.	Ich habe mich gefragt, ob ich mit meinem Herzfehler eine/n feste/n Freund/in finde.
	**Ich konnte im Sportunterricht nicht alles mitmachen.	Ich fühlte mich durch meinen Herzfehler in meiner Selbstständigkeit eingeschränkt.
		Ich war besorgt, welchen Beruf ich mit meinem Herzfehler machen kann.
		Ich habe mir überhaupt keine Sorgen um meine Zukunft gemacht.
**MeHeartRecovery**		
	Ich konnte nicht gut einschlafen.	Ich konnte nicht gut einschlafen.
	Ich bin nachts häufig aufgewacht.	Ich bin nachts häufig aufgewacht.
	Ich bin morgens schwer aus dem Bett gekommen.	Ich bin morgens schwer aus dem Bett gekommen.
	Ich bin erholt aufgewacht.	Ich bin erholt aufgewacht.
	Ich hatte einen ruhigen festen Schlaf.	Ich hatte einen ruhigen festen Schlaf.
	Ich habe mich gut nach anstrengenden Tagen erholt.	Ich habe mich gut nach anstrengenden Tagen erholt.
**MeHeartFriends**		
	Ich konnte an Aktivitäten mit Freunden teilnehmen.	Ich konnte an Aktivitäten mit Freunden teilnehmen.
	Ich hatte Schwierigkeiten Freunde zu finden.	Ich hatte keine Schwierigkeiten Freunde zu finden.
	Ich fühlte mich im Vergleich mit Freunden gleich gut.	Ich fühlte mich genauso selbstständig wie meine Freunde.
	Ich habe mich wohl gefühlt wie ich bin.	
**MeHeartTreatment**		
	Vor dem Krankenhaus habe ich keine Angst.	Vor dem Krankenhaus habe ich keine Angst.
	Ich finde Arztbesuche unangenehm.	Ich finde Arztbesuche unangenehm.
	Ich mag es nicht am Herzen untersucht zu werden.	Ich mag es nicht am Herzen untersucht zu werden.
**MeHeartSchool**		
	**Ich bin mit den Hausaufgaben gut zurechtgekommen.	Ich bin mit den Hausaufgaben gut zurechtgekommen.
	**Ich bin mit dem Unterrichtsstoff gut mitgekommen.	Ich bin mit dem Unterrichtsstoff gut mitgekommen.

**Kinder, die noch nicht in die Schule gehen werden gebeten, bei diesen Fragen „Schule" durch „Kindergarten", „Lehrer" durch „Erzieher" und Mitschüler durch „andere Kinder" zu ersetzen. **Diese Items sind bei Kindern, die noch nicht zur Schule gehen nicht auswertungsrelevant.*

Der Fokus liegt im Folgenden auf den zwei Hauptversionen des CHDSI mit 36 Items (Schulkinder) bzw. 37 Items (Jugendliche), da die Fallzahl der Kindergartenkinder nicht ausreichend hoch für eine umfassende Modellprüfung ist. Gleichwohl wird – sofern möglich und sinnvoll – auch eine statistische Auswertung und Beurteilung der fünf modifizierten Skalen der DsQoL von Kindergartenkindern durchgeführt. Wenn im weiteren Verlauf von Kindern gesprochen wird, sind hierbei stets Schulkinder und somit immer die Fragebogenversion mit 36 Items gemeint.

Wie Tabelle 1 zu entnehmen ist, sind bei beiden Fragebogenversionen von den insgesamt 43 Items 36 (Kinder) bzw. 37 (Jugendliche) jeweils einer der sechs postulierten Skalen zugeordnet. Die Skalen erfassen bei Kindern und Jugendlichen je ein vergleichbares Teilkonstrukt der DsQoL. In den durchgeführten statistischen Voranalysen während der Skalenkonstruktion und im Austausch mit den Kindern und Jugendlichen im Rahmen der Fragebogenentwicklung bestätigte sich – wie schon vorab vermutet – dass nicht alle Aspekte der DsQoL gleich wahrgenommen und bewertet werden. So umfassen vier der sechs Skalen die gleiche Anzahl identisch formulierter Items, während die Items zur Erfassung zwischen Kindern und Jugendlichen in zwei der sechs Skalen teils leicht unterschiedlich sind (vgl. Tabelle 1), da sich Wahrnehmung und Interpretation des Erlebten mit steigendem Alter verändern. So spielen für Kinder beispielsweise Sorgen darüber, welchen Beruf sie mit ihrem AHF einmal ausüben können, im Gegensatz zu jugendlichen AHF-Patienten keine Rolle. Entsprechend scheint die vorab getroffene Entscheidung zur Konstruktion von zwei altersspezifischen Messinstrumenten plausibel, da sich Kinder und Jugendliche in unterschiedlichen Lebensabschnitten mit oftmals vergleichbaren, im Detail allerdings auch voneinander abweichenden Bedürfnissen, Gedanken, Gefühlen, Herausforderungen und Problemen im Allgemeinen und vor dem Hintergrund des AHF im Speziellen befinden.

Insgesamt sechs identische Items für Kinder und Jugendliche sowie bei den Kindern ein siebtes Item sind – obgleich kein Bestandteil der konstruierten Skalen – als wichtig für die Beurteilung der DsQoL zu verstehen. Sie sind folglich nicht in Tabelle 1 aufgeführt und werden entsprechend auch nicht zur Überprüfung des vorgeschlagenen Modells mittels CFA, Skalenprüfung oder Reliabilitätsberechnung herangezogen. Im Laufe der Fragebogenentwicklung wurden diese Items allerdings sowohl von Befragten, Behandlern als auch Wissenschaftlern als hilfreich erachtet, um einen möglichst umfassenden Eindruck über die Lebens- und Behandlungssituation der jungen Patienten zu erhalten und die DsQoL so noch detaillierter beurteilen zu können, ohne wichtige Aspekte zu übersehen.

Die folgenden Items sind kein Bestandteil der konstruierten Skalen:

- <u>... meine Eltern mir gegenüber anders verhalten als meinen Geschwistern gegenüber.</u>
  Begründung: *Nicht alle Patienten haben ein bzw. mehrere Geschwisterkinder. Das Item kann jedoch als Indikator-Item, also als ein Single-Screening-Item für einen ersten Eindruck zu möglichen familiären Problemen verstanden werden.*
- <u>... abends öfter dicke Beine und Füße.</u>
  Begründung: *Nur ein kleiner Teil der Befragten mit spezifischen Diagnosen ist von diesen Symptomen betroffen. Das Item kann als Indikator-Item, also als ein Single-Screening-Item für einen ersten Eindruck zum medizinischen Zustand von Patienten mit spezifischen Diagnosen verstanden werden und ist für diese sowohl aus Behandler- als auch aus Patientenperspektive als hoch relevant beurteilt worden.*
- <u>Ich weiß gut Bescheid über mein Herz und was ihm fehlt.</u>
  Begründung: *Das Item kann als Indikator-Item, also als ein Single-Screening-Item für einen ersten Eindruck über den subjektiven Wissenstand des Patienten zum AHF verstanden werden. Es ermöglicht bei niedrigem Scoring die niederschwellige und direkte Zugänglichmachung von Informationsmaterial sowie das Angebot eines persönlichen Gesprächs zur Klärung von Unklarheiten und die Beantwortung von Fragen, um mögliche und für den weiteren Krankheitsverlauf potentiell negative Wissenslücken unmittelbar zu schließen.*
- <u>Ich vertrage meine Medikamente gut.</u>
  Begründung: *Nicht alle Patienten sind auf die regelmäßige Einnahme von Medikamenten angewiesen. Folglich ist dieses Item zwar für bestimmte, jedoch nicht für alle AHF-Patienten relevant, soll aber helfen einen Eindruck zur Verträglichkeit von ggf. eingenommenen*

Medikamenten zu bekommen, um bei Bedarf Probleme anzusprechen, Lösungen zu suchen und so im Idealfall auch die Compliance bei der Medikamenteneinnahme zu fördern.

- <u>Ich finde es nervig Medikamente zu nehmen.</u>
  Begründung: *Vgl. Begründung zum Item „Ich vertrage meine Medikamente gut".*
- <u>Ich finde meine Operationsnarbe sehr unangenehm.</u>
  Begründung: *Nicht alle Patienten mit AHF haben eine Operationsnarbe. Gleichwohl kann eine solche Narbe, sofern vorhanden, vor allem mit dem Beginn der Pubertät eine erhebliche psychische, in manchen Fällen aber auch körperliche Belastung darstellen. Das Item kann als Indikator-Item, also als ein Single-Screening-Item für Patienten gelten, die eine Operationsnarbe aufgrund einer Herzoperation haben und sich ggf. mit psychischen und/oder körperlichen Belastungen durch diese Operationsnarbe konfrontiert sehen.*
- <u>Ich gehe grundsätzlich gerne zur Schule.</u>
  Begründung: *Im Regelfall beziehen sich die Items auf die letzten Wochen vor dem Ausfüllen des Fragebogens. Es zeigte sich allerdings, dass für viele Kinder die Frage danach, ob sie grundsätzlich gerne zur Schule gehen (also nicht nur in den letzten Wochen) eine wichtige Frage war, die sich gleichzeitig keiner der konstruierten Skalen sinnvoll zuordnen ließ. Die Jugendlichen bewerteten das Item überwiegend als wenig relevant, weshalb dieses Item nur in die Kinderversion aufgenommen wurde. Das Item kann als Indikator-Item, also als ein Single-Screening-Item für grundsätzliche Sorgen, Probleme oder Ängste im Zusammenhang mit dem Schulbesuch verstanden werden und ermöglicht bei niedrigem Scoring beispielsweise im Arztgespräch die Identifizierung von Schwierigkeiten durch schulbezogene psychische und/oder körperliche Belastungen sowie eine gemeinsame Entwicklung angemessener Lösungsstrategien.*

Abgesehen von den beschriebenen Indikator-/Singleitems sind die jeweils verbliebenen 36 bzw. 37 Items einer der sechs postulierten latenten Variablen der DsQoL zuzuordnen. Es wurden folgende Skalen konstruiert:

MeHeartImpairments:	Die Skala erfasst mit jeweils 11 Items (Kinder und Jugendliche) potentielle Einschränkungen und Symptome, die das Leben mit einem AHF einschränken, belasten und im schlimmsten Fall sogar verkürzen können, sofern nicht zeitnah und zielorientiert interveniert wird.
MeHartStigma:	Die Skala erfasst mit 10 Items (Kinder) und 12 Items (Jugendliche) potentiell stigmatisierende Ereignisse/Situationen sowie Gedanken/Gefühle, die das Leben mit einem AHF einschränken und belasten und im schlimmsten Fall sogar zur Manifestation einer psychischen Komorbidität führen können, sofern nicht zeitnah und zielorientiert interveniert wird.
MeHeartRecovery:	Die Skala erfasst mit jeweils 6 identischen Items in der Kinder- und Jugendversion das Thema Schlaf und Erholung im Allgemeinen sowie nach anstrengenden Tagen. Sowohl ein gesunder Schlaf als auch eine gute Erholungsfähigkeit sind besonders für Menschen mit einem AHF sehr wichtig, um ein gutes und gesundes Leben vor dem Hintergrund der chronischen angeborenen Erkrankung führen zu können. Ein niedriges Scoring in dieser Subskala deutet auf Probleme im Bereich Erholung hin und ein frühzeitiges Erkennen und Reagieren kann helfen, einer Manifestation oder gar einer Chronifizierung z. B. von Schlafproblemen zu begegnen.
MeHeartFriends:	Für Kinder und jugendliche Patienten erfasst diese Skala mit 4 Items (Kinder) und 3 Items (Jugendliche) das Thema Freunde und Selbstständigkeit vor dem Hintergrund des AHF, denn Peer-Beziehungen und der Vergleich mit Gleichaltrigen spielen für Kinder und Jugendliche eine wichtige Rolle.
MeHeartTreatment:	Patienten mit AHF sollten grundsätzlich mindestens regelmäßige Routineuntersuchungen wahrnehmen. Die kontinuierliche medizinische Versorgung kann unter Umständen als sehr belastend und einschränkend, teils sogar als angstauslösend empfunden werden, was einer maximal guten Krankheitsbewältigung und auch einer hohen erforderlichen Compliance entgegenstehen kann. Daher gibt diese Skala einen Einblick in das subjektive Empfinden der medizinischen Behandlungs- und Versorgungssituation mit jeweils 3 identischen Items in beiden Fragebögen.
MeHeartSchool:	Schule kann für Kinder und Jugendliche mit AHF – ähnlich wie für herzgesunde Kinder/Jugendliche – zu einer herausfordernden Belastung werden. Mögliche Lernschwierigkeiten in den Bereichen Unterricht und Hausaufgaben werden in beiden Fragebogenversionen mit jeweils zwei identischen Items erfasst.

4. ANWENDUNGSMÖGLICHKEITEN DES CHDSI

Das zur Verfügung stellen eines validen und normierten Messinstruments zur Erfassung der individuellen DsQoL bei Kindern und Jugendlichen mit AHF bietet einen zusätzlichen Behandlungsansatz und schließt eine wichtige Lücke in derzeitigen Behandlungsplänen unter Berücksichtigung der Patientenbedürfnisse. Zudem können stigmatisierende, traumatisierende oder tabuisierte Sorgen, Wünsche oder Gedanken im Freitextfeld am Ende des Fragebogens thematisiert werden. So kann der CHDSI zukünftig für die strategische sowie operative Ausrichtung z. B. von Behandlungsplänen, Integrationskonzepten im Bereich Schul- und Vereinsleben, klinische Versorgungsstrategien und den alltäglichen Herausforderungen eines Lebens mit AHF sowie für den Einsatz im Rahmen von (klinischen) Studien mit jungen und jugendlichen AHF-Patienten herangezogen werden.

5. DATENANALYSE UND STATISTIK

Die Befragung wurde online durchgeführt. Die Teilnehmenden wurden zwei Gruppen zugeordnet (Kinder und Jugendliche). Die Probanden wurden per Post/E-Mail gebeten, an der Befragung teilzunehmen. Zur Entwicklung und Bereitstellung der zwei Fragebogenversionen und zur Datenerhebung selbst wurde die Software „EFS Survey" zur Erstellung und Durchführung von Online-Befragungen verwendet. Die erhobenen Daten wurden mit den Statistikprogrammen SPSS und RStudio ausgewertet.

Skalen und Scores

Zu Beginn beider Fragebögen werden demographische Standarddaten erhoben. Anschließend werden jeweils 43 Fragen gestellt. Bei diesen 43 Items handelt es sich um fünfstufige Skalenitems. Es wird ein Konstrukt der DsQoL von Kindern und Jugendlichen mit AHF aus 6 latenten Variablen postuliert, dass mittels 36 (Kinder) bzw. 37 (Jugendliche) der jeweils 43 Items gemessen wird. Mayer folgend sind die Voraussetzungen bei einem in diesem Fall fünfstufigen Antwortformat gegeben, um die Skalenitems als intervallskaliert zu behandeln, sofern das Antwortschema möglichst viele Antwortmöglichkeiten bietet, wobei es den Befragten noch problemlos möglich sein muss, zwischen den Antwortalternativen zu differenzieren[58]. Dieser Argumentation mag in bestimmten Fällen zu folgen sein, bei den Rating-Items des CHDSI mit fünf möglichen Antwortausprägungen (trifft voll zu, trifft zu, weiß nicht, trifft nicht zu, trifft gar nicht zu) liegt unserer Einschätzung nach jedoch kein Antwortformat vor, bei dem eine metrische Interpretation der Skalenitems uneingeschränkt sinnvoll erscheint. Die einzelnen Rating-Items werden daher – sofern statistisch möglich/sinnvoll/erforderlich – im Auswertungsprozess als ordinalskaliert behandelt.

Die fünf Stufen der 43 Rating-Items sind wie folgt zu kodieren:

- trifft voll zu (sehr glücklicher Smiley = 4)
- trifft gar nicht zu (sehr glücklicher Smiley = 4)
- trifft zu (glücklicher Smiley = 3)
- trifft nicht zu (glücklicher Smiley = 3)
- Weiß nicht (neutraler Smiley = 2)
- trifft zu (trauriger Smiley = 1)
- trifft nicht zu (trauriger Smiley = 1)
- trifft voll zu (sehr trauriger Smiley = 0)
- trifft gar nicht zu (sehr trauriger Smiley = 0)

Daraus ergeben sich für die konstruierten Skalen Scores, die minimal aus 0 Punkten und maximal aus 48 Punkten bestehen können folgende Punktzahlen:

MeHeartImpairments:	jeweils 0 bis 44 Punkte
MeHeartStigma:	0 bis 40 Punkte (Kinder), 0 bis 48 Punkte (Jugendliche)
MeHeartRecovery:	jeweils 0 bis 24 Punkte
MeHeartFriends:	0 bis 16 Punkte (Kinder), 0 bis 12 Punkte (Jugendliche)
MeHeartTreatment:	jeweils 0 bis 12 Punkte
MeHeartSchool:	jeweils 0 bis 8 Punkte

Insgesamt kann also bei den Kindern ein Gesamtscore zwischen 0 und 144 Punkten (maximal 124 Punkte bei Kindergartenkindern) und bei Jugendlichen zwischen 0 und 148 Punkten unter Berücksichtigung der beschriebenen Item-Kodierung (sehr trauriger Smiley = 0 bis sehr glücklicher Smiley = 4) erreicht werden. Sowohl die Summenscores der Subskalen als auch der Gesamtscore werden per fiat als intervallskaliert behandelt. Zur Darstellung des individuellen Levels der DsQoL in Prozent kann für jede erreichte Punktzahl ein dazugehöriger Prozentwert berechnet werden. Die folgenden Beispiele gelten für die Umrechnung des Gesamtscores der DsQoL in Prozentscores.

- Prozentscore Kindergartenkinder: *„Anzahl der Punkte" x 0,80645161*
- Prozentscore Kinder: *„Anzahl der Punkte" x 0,69444444*
- Prozentscore Jugendliche: *„Anzahl der Punkte" x 0,67567568*

Die Faktoren gelten nicht für die Umrechnung der Summenscores der Subskalen in Prozentscores (siehe nachfolgende Beispiele für die Umrechnung von Subskalensummenscores in Subskalenprozentscores).

Beispiele: Für Subskalen mit 12 Items (maximal 48 Punkte) ist der Faktor 2,08333333, mit 11 Items (maximal 44 Punkte) der Faktor 2,27272727 und mit 10 Items der Faktor 2,5 zu verwenden (Faktor = 100 / „maximal erreichbare Punktzahl der Skala").

EINGESETZTE STATISTISCHE VERFAHREN

Zur Beschreibung der Stichproben und der erhobenen DsQoL-Daten wird auf Verfahren der deskriptiven Statistik (Mittelwerte, Standardabweichung, Median, Minimum, Maximum, Berechnung prozentualer Anteile etc.) zurückgegriffen.

In Abhängigkeit des Skalenniveaus werden zur Berechnung von Korrelationen die Korrelationskoeffizienten nach Pearson bzw. Spearman verwendet. Nach Cohen können Korrelationen ab ± .10 als schwache Zusammenhänge, Korrelationen ab ± .30 als mittlere und ab ± .50 als starke Zusammenhänge interpretiert werden[59].

Bei der Beurteilung von Korrelationen folgen wir der Einteilung nach Cohen in schwache, mittlere und hohe Zusammenhänge.

Mögliche Gruppenunterschiede werden mit dem Chi-Quadrat-Test (χ^2-Test), dem Mann-Whitney-U-Test oder dem t-Test analysiert.

Neben der Normierung des CHDSI ist das zweite Kernziel die Validierung des CHDSI als Messinstrument für die DsQoL von Kindern und Jugendlichen mit AHF. Wenn wir von Validität sprechen, geht es einerseits um externe und andererseits um interne Validität. Die externe Validität prüft vereinfacht gesagt, ob sich die Forschungsergebnisse beispielsweise auf eine ganze Population, ähnliche Situationen oder Kontexte außerhalb derjenigen, in denen die Forschung stattgefunden hat übertragen lassen, bei der internen Validität steht das Messinstrument selbst im Fokus[60-62]. Gleiches gilt für die Reliabilität. Auch hier gibt es verschiedene Verfahren zur Bestimmung wie die Interne Konsistenz, Split-Half-Reliabilität oder die Test-Retest-Reliabilität[62-64].

Split-half-Reliabilität

Die Reliabilität wird unter anderem anhand der Split-Half-Reliabilität untersucht. Die Split-Half-Reliabilität ist ein statistisches Maß der Konsistenz der Ergebnisse eines Tests. Sie ist eine

ökonomische Alternative zu anderen Formen der Reliabilitätsmessung wie der Retest-Reliabilität und der Paralleltest-Reliabilität, da sie nur eine einzige Testdurchführung/Testform erfordert[65]. Die Items der zwei Fragebogenversionen (Kinder/Jugendliche) werden zur Berechnung jeweils in zwei Gruppen aufgeteilt. Hierbei gibt es verschiedene Vorgehensweisen (Einteilung nach geraden und ungeraden Itemnummern (odd-even split), Einteilung in eine erste und eine zweite Testhälfte, Einteilung nach der Methode der statistischen Itemzwillinge, zufällige Einteilung der Items). Die ungeraden Items wurden der ersten und die geraden Items der zweiten Testhälfte zugeordnet (odd-even-split). Diese beiden Testhälften sollten hoch korrelieren (r > .80)[66]. Die Korrelation zwischen den zwei Testhälften wird mit der Spearman-Brown Formel korrigiert, um eine Schätzung der Reliabilität der Messung des gesamten Instruments zu erhalten[67,68]. Die Spearman-Brown-Formel wird also als Korrekturfaktor verwendet, um die verminderte Split-Half-Reliabilität wieder auf die ursprüngliche Testlänge hochzurechnen, was zu einer Aufwertung der Testhalbierungs-Reliabilität führt[69].

Eine Split-half-Reliabilität ab .80 wird als ausreichend bewertet.

Interne Konsistenz, Trennschärfe und Homogenität

Die Interne Konsistenz der konstruierten Skalen wird durch Cronbachs Alpha spezifiziert. In der Praxis wird die Reliabilität oftmals in Form der internen Konsistenz geprüft, da ein einmaliger Testzeitpunkt ausreicht[70]. Cronbachs Alpha gibt an, wie zuverlässig und präzise die Items einer Skala eine latente Variable erfassen[71]. Nach Peterson ist für die Beurteilung von Cronbachs Alpha wesentlich, ob es sich um eine Voruntersuchung oder angewandte Forschung handelt, wobei unter Umständen auch ein Alpha von .50 noch als akzeptabel verstanden werden kann[63]. Bortz und Döring beschreiben eine hohe Interne Konsistenz von mindestens .80 für jeden Test als wünschenswert[72]. Ein hoher Alphawert bietet gewissermaßen einen Schutz davor, dass ein Item einer Skala einzigartig in dem Sinne ist, dass es ein Antwortmuster hervorruft, das sich von allen anderen Items derselben Skala erheblich unterscheidet[73]. Dies bedeutet gleichwohl nicht, dass alle Items einer Skala genau das gleiche Antwortmuster hervorrufen sollten, da dies bedeuten könnte, dass eine Skala redundante Items enthält. Nach Cortina war es lange üblich, dass ein *Alpha* > .70 ausreichte, um davon auszugehen, dass keine weitere Skalenentwicklung erforderlich war, was eine unangemessene Verwendung der Statistik darstellt, da Cronbachs Alpha tendenziell mit der Größe eines Instruments zunimmt[73,74]. Alpha auf .70 zu erhöhen ist beispielsweise möglich, indem man mehr Items zu einem Test hinzufügt, solange diese Items den bestehenden nicht völlig unähnlich sind. Gleichzeitig sollte nicht argumentiert werden, dass ein akzeptables Alpha hätte erreicht werden können, wären mehr Items aufgenommen worden um zu rechtfertigen, dass ein Alpha unter .70 nicht auf ein Problem mit dem Instrument hinweist[75]. Cronbach selbst argumentierte, dass der wichtigste Punkt die Interpretierbarkeit der erzielten Ergebnisse eines Messinstruments sein muss, was oft möglich ist, ohne dass dafür ein hohes Alpha erforderlich ist[64]. Nach Schmitt gibt es kein allgemeines Niveau, ab dem Alpha akzeptabel wird, denn auch Instrumente mit niedrigem Alpha können sich unter bestimmten Umständen als nützlich erweisen[74].

Wir interpretieren Cronbachs Alpha > .60 als akzeptabel, > .70 als gut, > .80 als sehr gut und > .90 als exzellent, wobei die beschriebenen Aspekte bei der Ergebnisbewertung stets berücksichtigt werden sollten.

Die Trennschärfe ist definiert als die Korrelation der Beantwortung eines Items mit dem Gesamtwert aller Items einer Skala[72]. Für jedes Item einer Skala wird ein korrigierter Trennschärfekoeffizient angegeben, der das betreffende Item aus der Korrelation ausschließt. Andernfalls würde sich die Korrelation durch die Einbeziehung des Items künstlich erhöhen. Die Ergebnisse der

Trennschärfenberechnung haben gegebenenfalls den Ausschluss einzelner Items zur Folge. Weist ein Item eine Trennschärfe nahe 0 auf, misst es offenbar etwas anderes als die anderen Items der Skala, weist es eine (hohe) negative Trennschärfe auf liegt dies in der Regel entweder daran, dass das Item „umgekehrt“ verstanden wurde oder bei der Auswertung nicht richtig gepolt war. Entsprechend sind positive Werte der Trennschärfe erforderlich. Nach Bortz und Döring ist eine Trennschärfe von < .50 als hoch zu interpretieren[72] und Fisseni[76] schlägt eine Einteilung in drei Kategorien vor: niedrig (< .30), mittel (.30 - .50), hoch (> .50).

Sofern nicht plausible inhaltliche Gründe dagegensprechen, werden Items mit einer Trennschärfe < .30 verworfen.

Die durchschnittliche Inter-Item-Korrelation einer Skala (MIC) sollte .20 nicht unter und .50 nicht überschreiten[68,77,78], wobei berücksichtigt werden muss, dass Items mit hoher Trennschärfe einen höheren MIC-Wert und somit einen homogeneren Test/Fragebogen bedeuten[79], was je nach Testart und zu prüfendem Modell bis zu einem gewissen Maß durchaus als wünschenswert zu verstehen sein kann.

Nach unserer Auffassung ist ein MIC-Wert zwischen .30 und .60 akzeptabel, ein Wert zwischen .40 und .60 vor dem Hintergrund der entwickelten Items, konstruierten Skalen und dem vorgeschlagenen Modell der DsQoL plausibel und ein MIC-Wert zwischen .40 und .50 wünschenswert, während ein MIC-Wert zwischen .60 und .80 als fragwürdig beurteilt wird sowie ein MIC-Wert von unter .20 oder über .80 zwingend eine umfassende Überarbeitung der entsprechenden Items und demnach der betroffenen Skala selbst zur Folge haben muss.

Um das Konstrukt der DsQoL und das postulierte Modell der latenten Variablen respektive der konstruierten Skalen zu überprüfen, wird eine konfirmatorische Faktorenanalyse (CFA) durchgeführt. Es ist ausdrücklich darauf hinzuweisen, dass die im Folgenden dargestellten Methoden der CFA keineswegs einen Anspruch auf Vollständigkeit erheben, da es sich hierbei um ein bücherfüllendes komplexes Thema handelt. Gleiches gilt im Übrigen auch für die bis hierhin vorgestellten statistischen Verfahren/Methoden.

Konfirmatorische Faktorenanalyse

Im Laufe der Zeit hat sich das dreiteilige Konzept der Validität (Inhaltsvalidität, Kriteriumsvalidität und Konstruktvalidität) zu einem einheitlichen Konzept entwickelt in dem die Konstruktvalidität eine wichtige Rolle spielt[80,81]. Die erhobenen empirischen Daten sollen Aufschluss über die Art der Beziehung zwischen den hypothetischen Variablen und die durch Messinstrumente ermittelten Werte geben[82]. Die Faktorenanalyse ist die am häufigsten angewandte Methode, um ein postuliertes Konstrukt und somit die Konstruktvalidität zu untersuchen und Informationen über die interne Struktur des Messinstruments zu erhalten[83]. Bei der CFA müssen Voraussetzungen wie eine angemessene Stichprobengröße, zufällige Stichprobenziehung, multivariate Normalität der beobachteten Variablen und Intervallskalierung der latenten sowie der beobachteten Variablen erfüllt sein[84]. Im Regelfall erfüllen die Daten diese Voraussetzungen jedoch nicht, denn überwiegend erfolgt die Datenerhebung mittels Fragebögen, bei denen das latente Konstrukt kontinuierlich ist, die beobachteten Variablen jedoch anhand einer Ordinalskala gemessen werden[85-90]. So ist vielfach mindestens eine der Voraussetzungen offenkundig nicht erfüllt, nämlich die kontinuierliche Natur sowohl der latenten als auch der beobachteten Variablen. Dennoch scheint es üblich, beobachtete Variablen so zu behandeln, als wären sie intervallskaliert. In den meisten Studien wird entsprechend die Maximum-Likelihood-Methode (ML) bei der Parameterschätzung der CFA verwendet[91]. Da angenommen wird, dass die beobachteten Variablen einer Intervallskala entsprechen, basiert die

ML-Schätzung auf einer Pearson-Korrelationsmatrix[88]. Zu den Problemen im Zusammenhang mit der Verwendung von Pearson-Korrelationen in Likert-Skalen[92] gehört eine geringere Korrelation zwischen den beobachteten Variablen[86,93,94]. Dieser Umstand wiederum verringert die Beziehung zwischen latenten Konstrukten und beobachteten Variablen. Zusätzlich stellt auch die Schiefe ordinaler Items ein weiteres Problem dar[88], denn die ML-Methode erfordert, dass die beobachteten Variablen der Normalverteilung folgen. Es wird argumentiert, dass die ML-Methode gegenüber der Nichteinhaltung dieser Annahme robust ist[95,96] und dass ML verwendet werden kann, wenn die univariate Schiefe in allen Variablen der durchgeführten Analyse kleiner als 1 ist[97]. Insbesondere wenn kontinuierliche Beobachtungsdaten nicht der Normalverteilung folgen, ist es gleichwohl ratsam, eine asymptotisch verteilungsfreie Methode wie WLS (weighted least squares) zu verwenden[91,98]. Auch die ULS-Methode (unweighted least squares) setzt keine bestimmte Verteilung der beobachteten Variablen voraus[91,99,100].

Der beste Ansatz für die Analyse ordinaler Variablen (mit wenigen Kategorien) scheint die Verwendung der DWLS-Methode zu sein, da dieser Ansatz gut funktioniert, wenn die Stichprobengröße ≥ 200 beträgt[89,101-103]. In den vorgenommenen Analysen wurde dem folgend der DWLS-Schätzer verwendet (lavaan package; RStudio), der mit dem Syntaxbefehl „ordered = TRUE“ zur Verfügung steht.

<u>Angemessener Stichprobenumfang</u>

Eine angemessene statistische Aussagekraft trägt dazu bei, wahre Zusammenhänge in einem Datensatz zu beobachten. Hierfür ist eine ausreichende, aber nicht übermäßige Stichprobengröße erforderlich. Die vorhandene Literatur bietet jedoch nur begrenzte und manchmal widersprüchliche Anleitungen zu diesem Thema. Bei der CFA sind Parameterschätzungen, Chi-Quadrat-Tests und Anpassungsindizes gleichermaßen empfindlich gegenüber der Stichprobengröße. Die statistische Aussagekraft und die Genauigkeit der Parameterschätzungen werden also auch vom Stichprobenumfang beeinflusst. Je nach Komplexität des Modells können Stichprobengrößen von mehr als 250 erforderlich sein[104,105]. Für eine CFA von 36 bzw. 37 Items sind entsprechend dem Vorschlag von Bentler und Chou insgesamt 360 bzw. 370 Teilnehmer für ein Mindestverhältnis von Probanden zu Items erforderlich (10 Probanden pro Item)[106]. Comrey und Lee schlagen folgende Einteilung der Güte von Normstichproben vor: 100 = poor, 200 = fair, 300 = good, 500 = very good, ≥1000 = excellent[107].

Da es sich um eine Fragebogennormierung/-validierung handelt, ist mindestens ein Stichprobenumfang von > 500 zu fordern, wobei anzumerken ist, dass die geforderten Stichprobengrößen auf den in der Literatur als gut klassifizierten Fallzahlen in Abhängigkeit zu den verwendeten statistischen Verfahren, Anforderungen an Normstichproben sowie ökonomisch vertretbarem Aufwand beruhen.

6. MODELLPASSUNG

Zur Überprüfung des postulierten Modells wurde eine CFA unter der Verwendung der DWLS-Schätzmethode mit dem Programm R mit dem Paket *„lavaan“* durchgeführt. Im Folgenden soll auf die zentralen Parameter der CFA eingegangen und ihre Interpretation vorgestellt werden, wobei nur ein grundlegender Überblick gegeben werden kann.

Model chi-square (χ^2)

Der χ^2-Wert ist das traditionelle Maß für die Bewertung der allgemeinen Modellanpassung[108]. Eine gute Modellanpassung würde bei einem Schwellenwert von .05 ein nicht signifikantes Ergebnis liefern[109]. Die χ^2-Statistik kann daher auch als Maß für eine schlechte Modellanpassung[110] oder als Maß für den Mangel an Modellanpassung[111] verstanden werden. Obwohl der χ^2-Test als Messinstrument für die Modellanpassung nach wie vor beliebt ist, gibt es eine Reihe schwerwiegender Einschränkungen bei seiner Verwendung. So geht der χ^2-Test von multivariater Normalität aus. Starke Abweichungen von der Normalität können zu einer Ablehnung des Modells führen, selbst wenn das Modell richtig spezifiziert ist[112]. Zusätzlich ist die χ^2-Statistik im Wesentlichen ein statistischer Signifikanztest und reagiert folglich sehr empfindlich auf die Stichprobengröße. Dies bedeutet, dass die χ^2-Statistik bei großen Stichproben fast immer das Modell zurückweist[113,114]. Andererseits ist die χ^2-Statistik bei kleinen Stichproben nicht sehr aussagekräftig und kann daher nicht zwischen gut und schlecht passenden Modellen unterscheiden[115]. Um diesem Problem zu begegnen wurde das relative/normierte χ^2 von Wheaton et al. (χ^2/df) vorgeschlagen. Obwohl es keinen Konsens über ein akzeptables Verhältnis für diese Statistik gibt, schlagen gängige Empfehlungen einem Wert von 5,0[116] bis zu einem Wert von 2,0[117] als Cut-Off-Wert vor.

Wir interpretieren einen relativen/normierten χ^2 unter 1 als exzellent, 1 bis < 2 als sehr gut, 2 bis < 3 als gut, 3 bis < 4 als akzeptabel und einen Wert von 4 bis 5 als grenzwertig.

Root mean square error of approximation (RMSEA)

Der RMSEA wurde 1980 von Steiger und Lind entwickelt[118]. Der RMSEA gibt an, wie gut das Modell mit unbekannten, aber optimal gewählten Parameterschätzungen an die Kovarianzmatrix der Populationen angepasst ist[119]. In den letzten Jahren wurde er als einer der informativsten Anpassungsindizes angesehen[120]. Der RMSEA begünstigt die Sparsamkeit, da er das Modell mit der geringsten Anzahl von Parametern auswählt. Bis Anfang der neunziger Jahre galt ein RMSEA im Bereich von .05 bis .10 als Hinweis auf eine gute Anpassung und Werte über .10 als Hinweis auf schlechte Anpassung[121]. Man ging dann davon aus, dass ein RMSEA zwischen .08 und .10 eine mittelmäßige Passung und ein Wert unter .08 eine gute Passung anzeigt. In jüngster Zeit scheint jedoch ein Grenzwert von .06[104] oder eine Obergrenze von .07[122] allgemeiner Konsens unter den Experten in diesem Bereich zu sein.

Wir interpretieren einen RMSEA von < .07 als akzeptabel, von < .06 als gut und von < .05 als sehr gut.

Comparative fit index (CFI) & Tucker Lewis index (TLI)

Der CFI vergleicht die Passung eines Zielmodells mit der Passung eines unabhängigen Modells, d. h. eines Modells, bei dem angenommen wird, dass die Variablen nicht korreliert sind. In diesem

Zusammenhang bezieht sich die Anpassung auf die Differenz zwischen der beobachteten und der vorhergesagten Kovarianzmatrix, die durch den χ^2-Index dargestellt wird.

Kurz gesagt, der CFI stellt das Verhältnis zwischen der Diskrepanz dieses Zielmodells und der Diskrepanz des Unabhängigkeitsmodells dar. Der CFI gibt also grob das Ausmaß an, in dem das Zielmodell besser ist als das Unabhängigkeitsmodell. Werte, die sich 1 nähern, weisen auf eine akzeptable Anpassung hin. Der CFI ist nicht allzu empfindlich gegenüber der Stichprobengröße[123]. Allerdings ist der CFI nicht effektiv, wenn die meisten Korrelationen zwischen den Variablen gegen 0 gehen, weil es dann weniger Kovarianz zu erklären gibt und der CFI ein verzerrtes Maß ist, das auf der Nicht-Zentralität beruht[124,125].

Der Tucker-Lewis-Index (TLI) ist auch als nicht-normierter Anpassungsindex (NNFI) bekannt. Vereinfacht gesagt vergleicht er das vorgeschlagene Modell mit dem Nullmodell. Je näher der TLI-Wert an 1 liegt, desto besser ist das Modell; TLI-Werte über .95 sind wünschenswert[104] (sehr gut: gleich oder größer als .95; gut: zwischen .90 und .95; schlecht: zwischen .80 und .90[126].

Cut-Off-Werte von CFI, TLI & RMSEA

Bisherige Studien deuten darauf hin, dass zumindest einige alternative Anpassungsindizes (TLI, CFI, RMSEA) unter Verwendung von Standard-Cutoffs[104] auch unter Verwendung der DWLS-Methode recht gut abschneiden, solange der Stichprobenumfang angemessen groß ist (N = 250 oder größer)[105,127-129]. Finney & DiStefano schlagen für die Bewertung der Modellanpassung mit dem DWLS-Ansatz einen TLI und CFI von > .95 und einen RMSEA von < .05 vor[130].

Für eine gute Modellpassung fordern wir mindestens einen CFI und einen TLI von > .95, einen RMSEA von < .06, einen relativen/normierten χ^2 von < 3 und Faktorladungen > .50.

Modellzurückweisung

Der Ausschluss von Items und somit eine Modell- und Skalenrevision wird zwingend erforderlich, wenn in der CFA Items mit Faktorladungen < .50 vorliegen[131]. Das Modell ist ebenfalls in den folgenden Fällen zurückzuweisen:

- Cronbachs Alpha für eine der konstruierten Skalen < .60
- Split-half-Reliabilität bei Skalen mit nur 2 Items < .80
- Split-half-Reliabilität für gesamten Fragebogen < .80
- Trennschärfe eines Items < .30
- CFI und TLI < .95
- RMSEA > .06
- relativen/normierten χ^2 von > 3

7. EINZELITEMS IM ÜBERBLICK

Einzelitems Kinder

In der Skala MeHeartImpairments fanden sich signifikante Geschlechtsunterschiede bei den Items 5 und 11 ($p < .05$). Signifikante Unterschiede im Antwortverhalten fanden sich ebenfalls zwischen Patienten mit simplem und mit moderatem AHF bei den Items 7 ($p < .001$), 2 ($p < .01$) sowie den Items 1, 3 und 5 ($p < .05$). Zwischen simplen und komplexen AHF fanden sich signifikante Gruppenunterschiede bei den Items 1-3, 5 und 7 ($p < .001$) sowie den Items 4, 6, 10 und 11 ($p < .01$). Auch im Vergleich zwischen dem Antwortverhalten von Befragten mit moderatem und komplexem AHF lagen signifikante Gruppenunterschiede bei Item 2 ($p < .001$) sowie den Items 1, 5, 7 ($p < .01$) und den Items 3, 10 und 11 ($p < .05$) vor. Eine Übersicht aller Einzelitems der Skala ist Tabelle 2.1 zu entnehmen.

In der Skala MeHeartStigma fanden sich signifikante Geschlechtsunterschiede bei den Items 2 und 5 ($p < .05$). Signifikante Unterschiede im Antwortverhalten fanden sich ebenfalls zwischen Patienten mit simplem und mit moderatem AHF bei Item 3 ($p < .001$) sowie den Items 4-7, 9 und 10 ($p < .01$). Zwischen simplen und komplexen AHF fanden sich signifikante Gruppenunterschiede bei den Items 3-10 Items ($p < .001$) sowie dem Item 2 ($p < .01$). Auch im Vergleich zwischen dem Antwortverhalten von Befragten mit moderatem und komplexem AHF lagen signifikante Gruppenunterschiede bei den Items 5 und 8 ($p < .01$) sowie den Items 3, 6, 7 und 9 ($p < .05$) vor. Eine Übersicht aller Einzelitems der Skala ist Tabelle 2.2 zu entnehmen.

Bei den Einzelitems der Skala MeHeartRecovery fanden sich keine signifikanten Geschlechtsunterschiede. Auch das Antwortverhalten von Patienten mit simplem und mit moderatem AHF unterschied sich nicht signifikant. Zwischen simplen und komplexen AHF fanden sich signifikante Gruppenunterschiede bei den Items 1 ($p < .01$), 2 und 5 ($p < .05$). Das Antwortverhalten von Befragten mit moderatem und komplexem AHF unterschied sich lediglich bei Item 2 ($p < .05$) signifikant. Eine Übersicht aller Einzelitems der Skala ist Tabelle 2.3 zu entnehmen.

In der Skala MeHeartFriends fanden sich keine signifikanten Geschlechtsunterschiede der Einzelitems. Signifikante Unterschiede im Antwortverhalten fanden sich zwischen Patienten mit simplem und mit moderatem AHF bei Item 3 ($p < .01$). Zwischen simplen und komplexen AHF fanden sich signifikante Gruppenunterschiede bei Item 3 ($p < .001$), den Items 1 und 4 ($p < .01$) sowie Item 2 ($p < .05$) und das Antwortverhalten von Befragten mit moderatem und komplexem AHF unterschied sich bei den Items 3 und 4 ($p < .05$) signifikant. Eine Übersicht aller Einzelitems der Skala ist Tabelle 2.4 zu entnehmen.

In der Skala MeHeartTreatment fanden sich bei keinem der Einzelitems signifikante Geschlechtsunterschiede. Auch im Antwortverhalten zwischen Patienten mit simplem und moderatem AHF, mit simplem und komplexem AHF und zwischen moderatem und komplexem AHF lagen keine signifikanten Gruppenunterschiede vor. Eine Übersicht aller Einzelitems der Skala ist Tabelle 2.5 zu entnehmen.

In der Skala MeHeartSchool fanden sich keine signifikanten Geschlechtsunterschiede bei den Einzelitems. Signifikante Unterschiede im Antwortverhalten fanden sich zwischen Patienten mit simplem und moderatem AHF bei Item 1 ($p < .001$) und Item 2 ($p < .05$) sowie zwischen Befragten mit simplem und komplexem AHF bei Item 1 ($p < .001$) und Item 2 ($p < .01$). Das Antwortverhalten von Befragten mit moderatem und komplexem AHF unterschied sich nicht signifikant. Eine Übersicht aller Einzelitems der Skala ist Tabelle 2.6 zu entnehmen.

Bei den Indikator-Items/Single-Screening-Items fanden sich signifikante Geschlechtsunterschiede lediglich bei Item 2 (*p* < .05). Signifikante Unterschiede im Antwortverhalten fanden sich zwischen Patienten mit simplem und moderatem AHF bei Item 1 (*p* < .05) sowie zwischen Befragten mit simplem und komplexem AHF bei Item 1, 5 (*p* < .001) und Item 4 (*p* < .05). Das Antwortverhalten von Befragten mit moderatem und komplexem AHF unterschied sich bei den Items 1, 5 (*p* < .01) und 2 (*p* < .05) signifikant. Eine Übersicht aller Indikator-Items/Single-Screening-Items ist Tabelle 2.7 zu entnehmen.

Tabelle 2.1. *Einzelitems der Skala MeHeartImpairments (Kinder: N = 530).*

	Median	trifft gar nicht zu	trifft nicht zu	weiß nicht	trifft zu	trifft voll zu
**MeHeartImpairments**						
FB:19 (1) Ich konnte alle körperlichen Aktivitäten machen wie ich wollte.	4/4/4<br>4/4/3	5,5/6,5/4,6<br>6,7/4,1/4,6	11,3/12,5/10,3<br>0,8/11,3/20,2	4,3/3,2/5,3<br>5/4,6/4	20,4/22,2/18,8<br>12,6/18,6/24,3	58,5/55,6/61<br>74,8/61,3/46,8
FB:20 (2) Ich konnte alles machen ohne außer Puste zu geraten.	3/3/3<br>4/3/2	7,9/9,7/6,4<br>2,5/7,7/9,8	25,5/24,2/26,6<br>16,8/19,6/37,6	7,5/8,9/6,4<br>4,2/6,7/11,6	26,6/28,2/25,2<br>21/32,5/24,3	32,5/29/35,5<br>55,5/33,5/16,8
FB:21 (3) Ich hatte keine körperlichen Schmerzen.	4/4/4<br>4/4/3	3,6/3,6/3,5<br>3,4/4,1/3,5	12,8/13,3/12,4<br>6,7/11,3/17,3	7,5/6,5/8,5<br>6,7/5,7/9,8	21,9/25,8/18,4<br>15,1/23,2/23,7	54,2/50,8/57,1<br>68,1/55,7/45,7
FB:22 (4) Ich habe mich selten krank gefühlt.	3,5/3/4<br>4/4/3	3,2/2,8/3,5<br>0,8/3,6/4,6	8,5/8,1/8,9<br>5,9/9,3/8,7	7/8,9/5,3<br>5,9/6,7/6,9	31,3/31,9/30,9<br>26,1/27,8/37	50/48,4/51,4<br>61,3/52,6/42,8
FB:23 (5) Ich konnte problemlos meine Hobbies machen.	4/4/4<br>4/4/3	2,1/1,6/2,5<br>0,8/2,1/2,9	7,4/8,9/6<br>2,5/6,2/12,1	4,9/4,8/5<br>4,2/3,1/6,4	24,7/29/20,9<br>16/24,2/30,1	60,9/55,6/65,6<br>76,5/64,4/48,6
FB:24 (6) Ich fühlte mich körperlich frisch und munter.	4/3/4<br>4/3,5/3	1,1/1,2/1,1<br>0,8/1/1,7	7,7/6,5/8,9<br>6,7/5,7/10,4	7,4/8,5/6,4<br>7,6/7,2/8,7	32,3/35,5/29,4<br>21/36,1/31,8	51,5/48,4/54,3<br>63,9/50/47,4
FB:25 (7) … schnell keine Puste mehr.	3/3/3<br>4/3/3	42,6/37,9/46,8<br>63,9/41,8/30,6	24,9/25,4/24,5<br>19,3/27,3/26,6	9,6/12,5/7,1<br>5,9/10,8/11,6	18,9/20,6/17,4<br>7,6/16,5/27,7	4/3,6/4,3<br>3,4/3,6/3,5
FB:27 (8) … häufig mit Schwindel zu tun.	4/4/4<br>4/4/4	64,5/63,3/65,6<br>63,9/68/61,3	18,1/19/17,4<br>19,3/16/20,8	6,8/8,1/5,7<br>5/6,7/8,1	8,7/8,5/8,9<br>7,6/8,2/8,1	1,9/1,2/2,5<br>4,2/1/1,7
FB:28 (9) … auch Schmerzen in der Brust.	4/4/4<br>4/4/4	58,3/53,6/62,4<br>66,4/58,2/56,6	19,2/22,6/16,3<br>14,3/20,6/20,8	7,9/10,1/6<br>8,4/7,2/8,1	12,8/12,9/12,8<br>7,6/13,4/12,7	1,7/0,8/2,5<br>3,4/0,5/1,7
FB:29 (10) … gemerkt, dass ich schnell müde werde.	3/3/3,5<br>4/3,5/3	48,7/47,2/50<br>61,3/50/39,9	24,2/26,6/22<br>16/27,8/27,2	7,9/6/9,6<br>9,2/5,7/9,8	15,5/17,3/13,8<br>10,1/11,9/20,2	3,8/2,8/4,6<br>3,4/4,6/2,9
FB:30 (11) … öfter komisches Herzklopfen.	4/4/4<br>4/4/3	56,4/50,8/61,3<br>65,5/59,3/49,1	21,1/23,8/18,8<br>18,5/21,6/22,5	10,8/11,7/9,9<br>8,4/9,3/13,3	9,1/12,5/6<br>4,2/8,2/11,6	2,6/1,2/3,9<br>3,4/1,5/3,5

Angabe von Median und Häufigkeiten in Prozent: gesamt (N =530) / männlich (n = 248) / weiblich (n = 282) / simple AHF (n = 119) / moderate AHF (n = 194) / komplexe AHF (n = 173); FB:x = Die Angabe der Nummer der tatsächlichen Frage im Fragebogen

Tabelle 2.2. *Einzelitems der Skala MeHeartStigma (Kinder: N = 530).*

	Median	trifft gar nicht zu	trifft nicht zu	weiß nicht	trifft zu	trifft voll zu
**MeHeartStigma**						
FB:4 (1) Ich habe mir Sorgen über meine Zukunft gemacht.	3/3/3<br>4/3/3	46,4/48,8/44,3<br>54,6/46,9/42,8	25,1/23,8/26,2<br>21,8/27,3/25,4	14,5/14,5/14,5<br>10,1/14,9/16,2	10,2/10,1/10,3<br>9,2/7,2/13,9	3,8/2,8/4,6<br>4,2/3,6/1,7
FB:5 (2) Ich habe mich hilflos und traurig gefühlt.	4/4/3<br>4/4/3	51,3/56/47,2<br>60,5/51,5/46,2	28,5/27,8/29,1<br>25,2/28,4/30,6	10,2/9,3/11<br>10,9/9,3/11,6	8,5/6/10,6<br>3,4/9,8/9,8	1,5/0,8/2,1<br>…/1/1,7
FB:7 (3) ... meine Eltern viele Sorgen gemacht, das tut mir leid.	4/3/4<br>4/4/3	53,2/49,2/56,7<br>73,1/53,1/42,2	18,3/20,6/16,3<br>13,4/20,1/22	14,2/16,9/11,7<br>6,7/16/15,6	10/9,3/10,6<br>5,9/6,7/13,9	4,3/4/4,6<br>0,8/4,1/6,4
FB:8 (4) ... in der Schule Lehrer und Mitschüler mir gegenüber komisch verhalten.	4/4/4<br>4/4/4	71,9/70,6/73<br>86,6/72,7/64,2	19,2/21/17,7<br>10,9/20,1/23,1	4,5/4,8/4,3<br>…/3,6/7,5	4,2/3,6/4,6<br>2,5/3,6/5,2	0,2/…/0,4<br>…/…/…
FB:10 (5) ... meine Eltern und andere Erwachsene übervorsichtig mir gegenüber verhalten.	4/4/4<br>4/4/3	61,7/56,9/66<br>79,8/63,9/46,8	22,5/25/20,2<br>15,1/21,1/30,6	5,3/7,3/3,5<br>1,7/4,6/7,5	8,9/9,7/8,2<br>3,4/7,7/13,3	1,7/1,2/2,1<br>…/2,6/1,7
FB:11 (6) ... Lehrer und Mitschüler besonders um mich bemüht, ohne dass ich das wollte.	4/4/4<br>4/4/4	65,8/63,3/68,1<br>84/67/54,3	21,7/23,8/19,9<br>11,8/21,6/27,7	6,8/8,5/5,3<br>2,5/6,2/10,4	4,5/3,6/5,3<br>1,7/3,6/5,8	1,1/0,8/1,4<br>…/1,5/1,7
FB:12 (7) ... andere Kinder über mich lustig gemacht.	4/4/4<br>4/4/4	74,2/70,6/77,3<br>87,4/73,7/65,3	13,8/16,5/11,3<br>6,7/17/15	5,1/5,6/4,6<br>2,5/3,6/9,2	6,2/7,3/5,3<br>2,5/5,2/9,8	0,8/…/1,4<br>0,8/0,5/0,6
FB:13 (8) Ich habe viel Unterricht verpasst wegen Untersuchungen.	4/4/4<br>4/4/4	70,2/69,4/70,9<br>82,4/74,7/57,8	20,9/21,8/20,2<br>15,1/16/31,2	2,1/1,2/2,8<br>0,8/2,6/2,9	5,1/6/4,3<br>1,7/4,1/6,9	1,7/1,6/1,8<br>…/2,6/1,2
FB:14 (9) Ich konnte im Sportunterricht nicht alles mitmachen.	4/4/4<br>4/4/3	61,3/57,3/64,9<br>79,8/63,4/49,7	14,7/17,3/12,4<br>13,4/13,4/17,3	2,6/2,8/2,5<br>0,8/1,5/5,2	14,7/14,5/14,9<br>4,2/14,9/19,1	6,6/8,1/5,3<br>1,7/6,7/8,7
FB:15 (10) Ich habe mir Gedanken gemacht, ob ich die Schule schaffe.	4/4/4<br>4/4/4	66/67,3/64,9<br>81,5/63,9/57,2	15,8/14,5/17<br>9,2/19,1/15,6	7,5/9,3/6<br>4,2/7,7/11,6	7,2/6/8,2<br>3,4/6,2/11,6	3,4/2,8/3,9<br>1,7/3,1/4

Angabe von Median und Häufigkeiten in Prozent: gesamt (N =530) / männlich (n = 248) / weiblich (n = 282) / simple AHF (n = 119) / moderate AHF (n = 194) / komplexe AHF (n = 173); FB:x = Die Angabe der Nummer der tatsächlichen Frage im Fragebogen

Tabelle 2.3. *Einzelitems der Skala MeHeartRecovery (Kinder: N = 530).*

	Median	trifft gar nicht zu	trifft nicht zu	weiß nicht	trifft zu	trifft voll zu
**MeHeartRecovery**						
FB:31 (1) Ich konnte nicht gut einschlafen.	3/3/3<br>4/3/3	46,6/46,4/46,8<br>57,1/45,4/38,7	30,4/31/29,8<br>23,5/30,9/35,8	5,5/4,4/6,4<br>7,6/4,1/6,4	12,6/13,7/11,7<br>7,6/16/15	4,9/4,4/5,3<br>4,2/3,6/4
FB:32 (2) Ich bin nachts häufig aufgewacht.	3/4/3<br>4/4/3	49,8/53,2/46,8<br>55,5/55,2/42,8	27,4/23,4/30,9<br>25,2/26,3/29,5	6/5,2/6,7<br>5,9/5,2/6,9	12,8/13,7/12,1<br>10,9/10,3/16,8	4/4,4/3,5<br>2,5/3,1/4
FB:33 (3) Ich bin morgens schwer aus dem Bett gekommen.	3/3/3<br>3/3/3	33,4/34,7/32,3<br>37,8/34,5/29,5	29,4/29,4/29,4<br>29,4/28,4/31,2	7,7/9,7/6<br>10,1/7,2/5,2	18,9/16,5/20,9<br>14,3/19,6/22	10,6/9,7/11,3<br>8,4/10,3/12,1
FB:34 (4) Ich bin erholt aufgewacht.	3/3/3<br>3/3/3	3,4/3,2/3,5<br>1,7/2,1/5,2	14,9/12,5/17<br>12,6/13,4/16,8	11,3/12,1/10,6<br>10,1/10,8/13,3	36,8/39,9/34<br>38,7/38,7/33,5	33,6/32,3/34,8<br>37/35,1/31,2
FB:35 (5) Ich hatte einen ruhigen festen Schlaf.	3/3/3<br>4/3/3	4/3,2/4,6<br>0,8/3,1/5,2	10,2/10,9/9,6<br>7,6/9,3/11,6	10,8/10,9/10,6<br>14,3/9,8/10,4	31,7/31,9/31,6<br>24,4/33,5/35,3	43,4/43,1/43,6<br>52,9/44,3/37,6
FB:36 (6) Ich habe mich gut nach anstrengenden Tagen erholt.	3/3/3<br>4/3/3	1,9/1,6/2,1<br>0,8/1/1,7	5,5/3,6/7,1<br>5,9/4,6/6,9	11,5/12,5/10,6<br>10,9/11,3/10,4	36/38,3/34<br>31,1/37,6/39,3	45,1/44/46,1<br>51,3/45,4/41,6

Angabe von Median und Häufigkeiten in Prozent: gesamt (N =530) / männlich (n = 248) / weiblich (n = 282) / simple AHF (n = 119) / moderate AHF (n = 194) / komplexe AHF (n = 173); FB:x = Die Angabe der Nummer der tatsächlichen Frage im Fragebogen

Tabelle 2.4. *Einzelitems der Skala MeHeartFriends (Kinder: N = 530).*

	Median	trifft gar nicht zu	trifft nicht zu	weiß nicht	trifft zu	trifft voll zu
**MeHeartFriends**						
FB:1 (1) Ich fühlte mich im Vergleich mit Freunden gleich gut.	4/4/4<br>4/4/3	5,5/7,3/3,9<br>9,2/5,2/3,5	5,7/8,1/3,5<br>0,8/4,6/9,2	10,8/10,5/11<br>5/9,8/15	24,9/23,4/26,2<br>20,2/26,8/24,9	53,2/50,8/55,3<br>64,7/53,6/47,4
FB:2 (2) Ich habe mich wohl gefühlt wie ich bin.	4/4/4<br>4/4/4	0,9/0,4/1,4<br>0,8/…/1,7	4,7/5,2/4,3<br>2,5/3,6/6,4	4,7/5,6/3,9<br>3,4/5,7/3,5	25,3/23/27,3<br>21/28,9/27,7	64,3/65,7/63,1<br>72,3/61,9/60,7
FB:3 (3) Ich konnte an Aktivitäten mit Freunden teilnehmen.	4/4/4<br>4/4/4	1,5/1,6/1,4<br>0,8/2,6/0,6	5,3/5,6/5<br>0,8/4,6/9,8	3,6/4,4/2,8<br>2,5/3,6/4,6	23,6/25,4/22<br>12,6/23,2/29,5	66/62,9/68,8<br>83,2/66/55,5
FB:6 (4) Ich hatte Schwierigkeiten Freunde zu finden.	4/4/4<br>4/4/4	60,9/61,7/60,3<br>68,9/63,4/54,9	22,8/23,4/22,3<br>20,2/24,7/23,1	6/5,2/6,7<br>5/5,2/7,5	7,7/7,7/7,8<br>4,2/4,1/12,7	2,5/2/2,8<br>1,7/2,6/1,7

Angabe von Median und Häufigkeiten in Prozent: gesamt (N =530) / männlich (n = 248) / weiblich (n = 282) / simple AHF (n = 119) / moderate AHF (n = 194) / komplexe AHF (n = 173); FB:x = Die Angabe der Nummer der tatsächlichen Frage im Fragebogen

Tabelle 2.5. *Einzelitems der Skala MeHeartTreatment (Kinder: N = 530).*

	Median	trifft gar nicht zu	trifft nicht zu	weiß nicht	trifft zu	trifft voll zu
**MeHeartTreatment**						
[FB:38] (1) Vor dem Krankenhaus habe ich keine Angst.	3/3/3<br>3/3/3	9,1/11,7/6,7<br>5/11,3/8,1	19,4/17,7/20,9<br>14,3/15,5/26	11,5/10,9/12,1<br>18,5/10,8/8,7	25,3/22,6/27,7<br>21,8/28,4/25,4	34,7/37,1/32,6<br>40,3/34/31,8
[FB:40] (2) Ich finde Arztbesuche unangenehm.	3/3/3<br>3/3/3	30/31/29,1<br>33,6/29,9/27,7	34,2/34,3/34<br>29,4/36,1/35,3	10,4/8,9/11,7<br>16/6,7/10,4	16,4/16,1/16,7<br>14,3/17/17,3	9,1/9,7/8,5<br>6,7/10,3/9,2
[FB:41] (3) Ich mag es nicht am Herzen untersucht zu werden.	3/3/3<br>3/3/3	35,3/37,9/33<br>38,7/35,6/33,5	33,6/32,7/34,4<br>30,3/33/37	10,6/9,7/11,3<br>15,1/8,8/10,4	12,5/11,3/13,5<br>10,1/13,4/9,8	8,1/8,5/7,8<br>5,9/9,3/9,2

Angabe von Median und Häufigkeiten in Prozent: gesamt (N =530) / männlich (n = 248) / weiblich (n = 282) / simple AHF (n = 119) / moderate AHF (n = 194) / komplexe AHF (n = 173); FB:x = Die Angabe der Nummer der tatsächlichen Frage im Fragebogen

Tabelle 2.6. *Einzelitems der Skala MeHeartSchool (Kinder: N = 530).*

	Median	trifft gar nicht zu	trifft nicht zu	weiß nicht	trifft zu	trifft voll zu
**MeHeartSchool**						
[FB:16] (1) Ich bin mit dem Unterrichtsstoff gut mitgekommen.	4/3/4<br>4/3/3	1,5/1,6/1,4<br>1,7/0,5/2,9	7,5/7,3/7,8<br>3,4/10,3/8,1	8,1/9,7/6,7<br>5/8,8/8,7	29,2/31,9/27<br>20,2/31,4/32,4	53,6/49,6/57,1<br>69,7/49/48
[FB:17] (2) Ich bin mit den Hausaufgaben gut zurechtgekommen.	4/3/4<br>4/4/3	2,1/2,4/1,8<br>0,8/1,5/2,9	7,2/8,9/5,7<br>3,4/9,8/8,1	6,6/8,5/5<br>9,2/5,7/5,8	32,5/31,9/33<br>22,7/32,5/38,2	51,7/48,4/54,6<br>63,9/50,5/45,1

Angabe von Median und Häufigkeiten in Prozent: gesamt (N =530) / männlich (n = 248) / weiblich (n = 282) / simple AHF (n = 119) / moderate AHF (n = 194) / komplexe AHF (n = 173); FB:x = Die Angabe der Nummer der tatsächlichen Frage im Fragebogen

Tabelle 2.7. *Indikator-Items/Single-Screening-Items (Kinder: N = 530).*

	Median	habe keine/ nehme keine	trifft gar nicht zu	trifft nicht zu	weiß nicht	trifft zu	trifft voll zu
[FB:9] (1) ... meine Eltern mir gegenüber anders verhalten als meinen Geschwistern gegenüber.*	4/4/4<br>4/4/4	11,3/13,3/9,6<br>10,1/10,8/12,1	71,3/68,4/73,7<br>85/74/59,9	17,4/18,6/16,5<br>10,3/16,8/24,3	5,3/6/4,7<br>2,8/4,6/7,9	4,3/4,2/4,3<br>0,9/4/5,9	1,7/2,8/0,8<br>0,9/0,6/2
[FB:18] (2) Ich gehe grundsätzlich gerne zur Schule.	3/3/3<br>3/3/3		3,4/4/2,8<br>1,7/2,6/5,2	6,2/9,3/3,5<br>5,9/5,2/9,2	8,7/8,9/8,5<br>8,4/9,3/9,2	37,4/37,5/37,2<br>42/33,5/38,2	44,3/40,3/47,9<br>42/49,5/38,2
[FB:26] (3) ... abends öfter dicke Beine und Füße.	4/4/4<br>4/4/4		80,9/79,8/81,9<br>82,4/80,9/80,9	13,6/14,1/13,1<br>10,1/13,4/15,6	3,4/4/2,8<br>4,2/3,6/2,3	1,1/0,4/1,8<br>0,8/1,5/0,6	0,9/1,6/0,4<br>2,5/0,5/0,6
[FB:37] (4) Ich weiß gut Bescheid über mein Herz und was ihm fehlt.	3/3/3<br>3/3/3		2,3/3,2/1,4<br>1,7/2,1/3,5	7,7/6,9/8,5<br>5,9/10,3/7,5	16,4/15,7/17<br>17,6/17,5/15	33,2/30,2/35,8<br>26,9/28,4/41,6	40,4/44/37,2<br>47,9/41,8/32,4
[FB:39] (5) Ich vertrage meine Medikamente gut.*	4/4/4<br>3/4/4	65,7/61,3/69,5<br>91,6/74,7/37,6	1,1/…/2,3<br>…/…/0,9	2,2/2,1/2,3<br>…/6,1/…	7,7/6,3/9,3<br>40/10,2/4,6	25,8/27,1/24,4<br>40/30,6/21,3	63,2/64,6/61,6<br>20/53,1/73,1
[FB:42] (6) Ich finde es nervig Medikamente zu nehmen.*	3/2/3<br>3/2/3	63,8/58,9/68,1<br>89,1/73,7/35,8	22,9/23,5/22,2<br>30,8/21,6/25,2	27,6/24,5/31,1<br>23,1/17,6/28,8	12/10,8/13,3<br>15,4/13,7/10,8	20,3/24,5/15,6<br>15,4/25,5/18,9	17,2/16,7/17,8<br>15,4/21,6/16,2
[FB:43] (7) Ich finde meine Operationsnarbe sehr unangenehm.*	3/3/3<br>3/3/3	24/19,8/27,7<br>65,5/19,1/1,7	44,4/48,7/40,2<br>43,9/45,2/44,7	29,5/26,1/32,8<br>24,4/34,4/27,6	10,7/12,1/9,3<br>14,6/8,9/12,4	8,7/8/9,3<br>9,8/7/10	6,7/5/8,3<br>7,3/4,5/5,3

*Angabe von Median und Häufigkeiten in Prozent: gesamt (N =530) / männlich (n = 248) / weiblich (n = 282) / simple AHF (n = 119) / moderate AHF (n = 194) / komplexe AHF (n = 173) *Bei diesen Items beziehen sich die Prozentangaben für die Kategorie „habe keine/nehme keine" auf die Gesamt- bzw. Teilstichproben, die Angaben zu Median und den Ratingkategorien (trifft gar nicht zu – trifft voll zu) beziehen sich entsprechend auf die reduzierten Gesamt- und Teilstichproben; FB:x = Die Angabe der Nummer der tatsächlichen Frage im Fragebogen*

Einzelitems Kindergartenkinder

Die Einzelitems der Kindergartenkinder werden aufgrund der geringen Fallzahl (N = 46) nur für die Gesamtgruppe der Kindergartenkinder dargestellt. Eine weitere Unterteilung nach Geschlecht oder Schweregrad des AHF wurde nicht durchgeführt. Folglich können auch keine Aussagen zu möglicherweise vorliegenden Gruppenunterschieden gemacht werden. Details zu jedem Einzelitem der auf fünf reduzierten Subskalen der DsQoL von Kindergartenkindern sind den Tabellen 3.1 bis 3.6 zu entnehmen.

Tabelle 3.1. *Einzelitems der Skala MeHeartImpairments (Kindergartenkinder: N = 46).*

	Median	trifft gar nicht zu	trifft nicht zu	weiß nicht	trifft zu	trifft voll zu
**MeHeartImpairments**						
FB:19 (1) Ich konnte alle körperlichen Aktivitäten machen wie ich wollte.	4	2,2	6,5	10,9	23,9	56,5
FB:20 (2) Ich konnte alles machen ohne außer Puste zu geraten.	3	2,2	28,3	6,5	21,7	41,3
FB:21 (3) Ich hatte keine körperlichen Schmerzen.	3	4,3	6,5	6,5	34,8	47,8
FB:22 (4) Ich habe mich selten krank gefühlt.	3	2,2	8,7	13	39,1	37
FB:23 (5) Ich konnte problemlos meine Hobbies machen.	3,5	4,3	4,3	10,9	30,4	50
FB:24 (6) Ich fühlte mich körperlich frisch und munter.	4	2,2	…	6,5	37	54,3
FB:25 (7) … schnell keine Puste mehr.	3	37	28,3	10,9	21,7	2,2
FB:27 (8) … häufig mit Schwindel zu tun.	4	73,9	21,7	2,2	2,2	…
FB:28 (9) … auch Schmerzen in der Brust.	4	76,1	13	2,2	8,7	…
FB:29 (10) … gemerkt, dass ich schnell müde werde.	3	45,7	26,1	17,4	10,9	…
FB:30 (11) … öfter komisches Herzklopfen.	3,5	50	21,7	21,7	6,5	…

Angabe von Median und Häufigkeiten in Prozent; FB:x = Die Angabe der Nummer der tatsächlichen Frage im Fragebogen

Tabelle 3.2. *Einzelitems der Skala MeHeartStigma (Kindergartenkinder: N = 46).*

	Median	trifft gar nicht zu	trifft nicht zu	weiß nicht	trifft zu	trifft voll zu
**MeHeartStigma**						
FB:4 (1) Ich habe mir Sorgen über meine Zukunft gemacht.	4	54,3	21,7	17,4	6,5	…
FB:5 (2) Ich habe mich hilflos und traurig gefühlt.	4	52,2	21,7	15,2	8,7	2,2
FB:7 (3) … meine Eltern viele Sorgen gemacht, das tut mir leid.	3	28,3	37	28,3	2,2	4,3
FB:8 (4) … in der Schule Lehrer und Mitschüler mir gegenüber komisch verhalten.*	4	63	19,6	13	4,3	…
FB:10 (5) … meine Eltern und andere Erwachsene übervorsichtig mir gegenüber verhalten.	3	39,1	32,6	17,4	10,9	…
FB:11 (6) … Lehrer und Mitschüler besonders um mich bemüht, ohne dass ich das wollte.*	3	43,5	34,8	19,6	2,2	…
FB:12 (7) … andere Kinder über mich lustig gemacht.	4	63	23,9	8,7	4,3	…

*Angabe von Median und Häufigkeiten in Prozent; *Die Kinder wurden bei diesen Fragen gebeten, „Schule" durch Kindergarten, „Lehrer" durch „Erzieher" und „Mitschüler" durch „andere Kindergartenkinder" zu ersetzen, sofern sie noch nicht zur Schule gehen; FB:x = Die Angabe der Nummer der tatsächlichen Frage im Fragebogen*

Tabelle 3.3. *Einzelitems der Skala MeHeartRecovery (Kindergartenkinder: N = 46).*

	Median	trifft gar nicht zu	trifft nicht zu	weiß nicht	trifft zu	trifft voll zu
**MeHeartRecovery**						
FB:31 (1) Ich konnte nicht gut einschlafen.	4	52,2	23,9	10,9	13	…
FB:32 (2) Ich bin nachts häufig aufgewacht.	3	39,1	41,3	10,9	6,5	2,2
FB:33 (3) Ich bin morgens schwer aus dem Bett gekommen.	3	34,8	32,6	10,9	19,6	2,2
FB:34 (4) Ich bin erholt aufgewacht.	3	…	6,5	8,7	52,2	32,6
FB:35 (5) Ich hatte einen ruhigen festen Schlaf.	3	…	10,9	4,3	54,3	30,4
FB:36 (6) Ich habe mich gut nach anstrengenden Tagen erholt.	3	…	…	8,7	56,5	34,8

Angabe von Median und Häufigkeiten in Prozent; FB:x = Die Angabe der Nummer der tatsächlichen Frage im Fragebogen

Tabelle 3.4. *Einzelitems der Skala MeHeartFriends (Kindergartenkinder: N = 46).*

	Median	trifft gar nicht zu	trifft nicht zu	weiß nicht	trifft zu	trifft voll zu
**MeHeartFriends**						
[FB:1] (1) Ich fühlte mich im Vergleich mit Freunden gleich gut.	4	…	4,3	19,6	19,6	56,5
[FB:2] (2) Ich habe mich wohl gefühlt wie ich bin.	4	…	2,2	…	34,8	63
[FB:3] (3) Ich konnte an Aktivitäten mit Freunden teilnehmen.	4	2,2	6,5	4,3	23,9	63
[FB:6] (4) Ich hatte Schwierigkeiten Freunde zu finden.	4	54,3	21,7	15,2	6,5	2,2

Angabe von Median und Häufigkeiten in Prozent; FB:x = Die Angabe der Nummer der tatsächlichen Frage im Fragebogen

Tabelle 3.5. *Einzelitems der Skala MeHeartTreatment (Kindergartenkinder: N = 46).*

	Median	trifft gar nicht zu	trifft nicht zu	weiß nicht	trifft zu	trifft voll zu
**MeHeartTreatment**						
[FB:38] (1) Vor dem Krankenhaus habe ich keine Angst.	1,5	13	37	2,2	30,4	17,4
[FB:40] (2) Ich finde Arztbesuche unangenehm.	3	10,9	45,7	6,5	21,7	15,2
[FB:41] (3) Ich mag es nicht am Herzen untersucht zu werden.	3	23,9	41,3	10,9	17,4	6,5

Angabe von Median und Häufigkeiten in Prozent; FB:x = Die Angabe der Nummer der tatsächlichen Frage im Fragebogen

Tabelle 3.6. *Indikator-Items/Single-Screening-Items (Kindergartenkinder: N = 46).*

	Median	habe keine/ nehme keine	trifft gar nicht zu	trifft nicht zu	weiß nicht	trifft zu	trifft voll zu
[FB:9] (1) ... meine Eltern mir gegenüber anders verhalten als meinen Geschwistern gegenüber.*	4	13	52,5	15	20	10	2,5
[FB:26] (2) ... abends öfter dicke Beine und Füße.	4		82,6	13	2,2	2,2	…
[FB:37] (3) Ich weiß gut Bescheid über mein Herz und was ihm fehlt.	2		8,7	17,4	30,4	30,4	13
[FB:39] (4) Ich vertrage meine Medikamente gut.*	4	50	…	…	8,7	30,4	60,9
[FB:42] (5) Ich finde es nervig Medikamente zu nehmen.*	2	45,7	12	32	16	32	8
[FB:43] (6) Ich finde meine Operationsnarbe sehr unangenehm.*	3	13	27,5	35	17,5	12,5	7,5

*Angabe von Median und Häufigkeiten in Prozent; *Bei diesen Items beziehen sich die Prozentangaben für die Kategorie „habe keine/nehme keine“ auf die Gesamtstichprobe, die Angaben zu Median und den Ratingkategorien (trifft gar nicht zu – trifft voll zu) beziehen sich entsprechend auf die reduzierten Gesamtstichproben; FB:x = Die Angabe der Nummer der tatsächlichen Frage im Fragebogen*

Einzelitems Jugendliche

In der Skala MeHeartImpairments fanden sich signifikante Geschlechtsunterschiede bei den Items 1-4, 6 und 8-11 ($p < .001$) sowie bei Item 5 ($p < .01$) und Item 7 ($p < .05$). Signifikante Unterschiede im Antwortverhalten fanden sich ebenfalls zwischen Patienten mit simplem und mit moderatem AHF bei den Items 1 ($p < .01$) und 5 ($p < .05$). Zwischen simplen und komplexen AHF fanden sich signifikante Gruppenunterschiede bei den Items 1, 2, 5 und 7 ($p < .001$) sowie den Items 3, 4 und 11 ($p < .01$). Auch im Vergleich zwischen dem Antwortverhalten von Befragten mit moderatem und komplexem AHF lagen signifikante Gruppenunterschiede bei den Items 2 und 7 ($p < .01$) sowie 1 und 3 ($p < .05$) vor. Eine Übersicht aller Einzelitems der Skala ist Tabelle 4.1 zu entnehmen.

In der Skala MeHeartStigma fanden sich signifikante Geschlechtsunterschiede bei Item 2 ($p < .01$) sowie bei den Items 5 und 12 ($p < .05$). Signifikante Unterschiede im Antwortverhalten fanden sich ebenfalls zwischen Patienten mit simplem und mit moderatem AHF bei den Items 1-3, 6, 7 und 10 (p

< .001), bei den Items 4, 9, 11 und 12 ($p < .01$) sowie bei den Items 5 und 8 ($p < .05$). Zwischen simplen und komplexen AHF fanden sich signifikante Gruppenunterschiede bei den Items 1-4, 6, 7, 9-12 ($p < .001$) sowie den Items 5 und 8 ($p < .01$). Im Vergleich zwischen dem Antwortverhalten von Befragten mit moderatem und komplexem AHF lagen signifikante Gruppenunterschiede bei den Items 9 und 10 ($p < .001$), den Items 3 und 6 ($p < .01$) sowie den Items 1 und 12 ($p < .05$) vor. Eine Übersicht aller Einzelitems der Skala ist Tabelle 4.2 zu entnehmen.

In der Skala MeHeartRecovery fanden sich signifikante Geschlechtsunterschiede bei den Items 1 und 6 ($p < .001$) sowie bei den Items 2, 4 und 5 ($p < .01$). Signifikante Unterschiede im Antwortverhalten fanden sich ebenfalls zwischen Patienten mit simplem und mit moderatem AHF bei den Items 1 und 2 ($p < .05$). Zwischen simplen und komplexen AHF fanden sich signifikante Gruppenunterschiede lediglich bei Item 5 ($p < .05$) und das Antwortverhalten von Befragten mit moderatem und komplexem AHF unterschied sich bei keinem der Items signifikant. Eine Übersicht aller Einzelitems der Skala ist Tabelle 4.3 zu entnehmen.

In der Skala MeHeartFriends fanden sich signifikante Geschlechtsunterschiede bei Item 1 ($p < .05$). Signifikante Unterschiede im Antwortverhalten fanden sich ebenfalls zwischen Patienten mit simplem und mit moderatem AHF bei Item 3 ($p < .001$). Zwischen simplen und komplexen AHF fanden sich signifikante Gruppenunterschiede bei Item 3 ($p < .001$) sowie Item 1 und 2 ($p < .05$) und das Antwortverhalten von Befragten mit moderatem und komplexem AHF unterschied sich bei Item 1 signifikant ($p < .05$). Eine Übersicht aller Einzelitems der Skala ist Tabelle 4.4 zu entnehmen.

In der Skala MeHeartTreatment fanden sich signifikante Geschlechtsunterschiede bei allen drei Items: Item 3 ($p < .001$), Item 1 und 2 ($p < .01$). Zwischen Patienten mit simplem und moderatem AHF fanden sich keine signifikanten Unterschiede im Antwortverhalten und auch zwischen Befragten mit simplem und komplexem AHF fanden sich keine signifikanten Unterschiede, während sich Patienten mit moderatem und komplexem AHF bei Item 3 ($p < .05$) signifikant unterschieden. Eine Übersicht aller Einzelitems der Skala ist Tabelle 4.5 zu entnehmen.

In der Skala MeHeartSchool fanden sich keine signifikanten Geschlechtsunterschiede bei den Einzelitems. Signifikante Unterschiede im Antwortverhalten fanden sich zwischen Patienten mit simplem und moderatem AHF bei Item 2 ($p < .05$) sowie zwischen Befragten mit simplem und komplexem AHF ebenfalls bei Item 2 ($p < .01$). Das Antwortverhalten von Befragten mit moderatem und komplexem AHF unterschied sich bei keinem der Items signifikant. Eine Übersicht aller Einzelitems der Skala ist Tabelle 4.6 zu entnehmen.

Bei den Indikator-Items/Single-Screening-Items fanden sich keine signifikanten Geschlechtsunterschiede. Signifikante Unterschiede im Antwortverhalten fanden sich zwischen Patienten mit simplem und moderatem AHF bei Item 4 ($p < .01$) und Item 1 ($p < .05$) sowie zwischen Befragten mit simplem und komplexem AHF bei Item 1, 4 ($p < .001$) sowie Item 6 ($p < .05$). Das Antwortverhalten von Befragten mit moderatem und komplexem AHF unterschied sich bei Item 4 signifikant ($p < .01$). Eine Übersicht aller Einzelitems der Skala ist Tabelle 4.7 zu entnehmen.

Tabelle 4.1. *Einzelitems der Skala MeHeartImpairments (Jugendliche: N = 625).*

	Median	trifft gar nicht zu	trifft nicht zu	weiß nicht	trifft zu	trifft voll zu
**MeHeartImpairments**						
[FB:19] (1) Ich konnte alle körperlichen Aktivitäten machen wie ich wollte.	3/4/3<br>4/3/3	3,8/2,7/4,9<br>2,6/2,9/6,1	13,8/10,4/16,8<br>6,7/14,5/19,4	5,8/4,4/7<br>4,6/6,8/4,2	26,9/26,2/27,5<br>21,5/27,1/32,1	49,8/56,4/43,7<br>64,6/48,8/38,2
[FB:20] (2) Ich konnte alles machen ohne außer Puste zu geraten.	3/3/2<br>3/3/1	9,1/6,4/11,6<br>4,6/8,7/12,7	27,4/23,5/30,9<br>20,5/23,2/38,8	11/9,1/12,8<br>13,3/11,1/8,5	23,7/23,8/23,5<br>24,6/27,1/19,4	28,8/37,2/21,1<br>36,9/30/20,6
[FB:21] (3) Ich hatte keine körperlichen Schmerzen.	3/4/3<br>4/4/3	4/1,7/6,1<br>2,1/3,4/4,2	14,7/10,4/18,7<br>9,7/13/21,2	9,4/7/11,6<br>10,8/8,7/7,9	23/23,2/22,9<br>22,1/21,7/25,5	48,8/57,7/40,7<br>55,4/53,1/41,2
[FB:22] (4) Ich habe mich selten krank gefühlt.	3/4/3<br>4/3/3	3,2/2,7/3,7<br>2,1/3,4/3	9,6/8,1/11<br>7,2/10,6/13,3	10,2/6/14,1<br>10,3/8,7/10,3	30,6/30,2/30,9<br>25,6/30/33,3	46,4/53/40,4<br>54,9/47,3/40
[FB:23] (5) Ich konnte problemlos meine Hobbies machen.	4/4/4<br>4/4/4	1,8/1/2,4<br>2,1/1,4/1,8	7/5/8,9<br>3,1/8,2/8,5	5,1/4,7/5,5<br>2,6/4,3/6,7	23/21,1/24,8<br>18,5/22,2/27,3	63/68,1/58,4<br>73,8/63,8/55,8
[FB:24] (6) Ich fühlte mich körperlich frisch und munter.	3/4/3<br>3/3/3	3/1,3/4,6<br>3,6/2,9/2,4	8/6/9,8<br>5,1/6,8/12,1	14,1/11,4/16,5<br>10,8/14,5/14,5	30,4/29,9/30,9<br>31,8/31,9/28,5	44,5/51,3/38,2<br>48,7/44/42,4
[FB:25] (7) … schnell keine Puste mehr.	3/3/3<br>3/3/3	38,9/41,9/36,1<br>49,2/39,1/27,9	23,5/25,5/21,7<br>21,5/27,1/24,8	11,7/10,1/13,1<br>12,3/11,6/10,9	19,2/17,8/20,5<br>12,3/16,4/29,7	6,7/4,7/8,6<br>4,6/5,8/6,7
[FB:27] (8) … häufig mit Schwindel zu tun.	4/4/3<br>4/4/4	55/61,4/49,2<br>55,4/56/55,8	20,8/21,5/20,2<br>20/21,3/22,4	7,2/6,7/7,6<br>6,7/8,2/4,8	12,8/10,1/15,3<br>10,3/13,5/13,9	4,2/0,3/7,6<br>7,7/1/3
[FB:28] (9) … auch Schmerzen in der Brust.	3/4/3<br>4/3/3	46,7/53,4/40,7<br>52,8/45,4/43	21,4/23,8/19,3<br>18,5/24,2/24,8	9,9/7,7/11,9<br>9,7/9,7/8,5	17,6/14,4/20,5<br>14,4/17,4/20,6	4,3/0,7/7,6<br>4,6/3,4/3
[FB:29] (10) … gemerkt, dass ich schnell müde werde.	3/4/3<br>4/3/3	46,4/57,4/36,4<br>51,3/48,3/41,8	17,3/14,8/19,6<br>18,5/15,9/21,8	11,8/8,1/15,3<br>12,3/13/8,5	19,2/17,1/21,1<br>13,3/17,4/23	5,3/2,7/7,6<br>4,6/5,3/4,8
[FB:30] (11) … öfter komisches Herzklopfen.	3/4/3<br>4/4/3	49/56,7/41,9<br>55,9/50,2/43	17,9/15,8/19,9<br>15,9/18,8/17	12,5/12,8/12,2<br>11,3/11,6/14,5	15,8/12,8/18,7<br>13,3/15,9/18,8	4,8/2/7,3<br>3,6/3,4/6,7

Angabe des Median und von Häufigkeiten in Prozent: gesamt (N = 625) / männlich (n = 298) / weiblich (n = 327) / simple AHF (n = 195) / moderate AHF (n = 207) / komplexe AHF (n = 165); FB:x = Die Angabe der Nummer der tatsächlichen Frage im Fragebogen

Tabelle 4.2. *Einzelitems der Skala MeHeartStigma (Jugendliche: N = 625).*

	Median	trifft gar nicht zu	trifft nicht zu	weiß nicht	trifft zu	trifft voll zu
**MeHeartStigma**						
[FB:4] (1) Ich habe mich gefragt, ob ich mit meinem Herzfehler eine/n feste/n Freund/in finde.	4/4/4<br>4/4/3	65,3/62,1/68,2<br>83,6/59,9/49,7	11,8/14,8/9,2<br>5,1/14,5/17	13,6/15,8/11,6<br>7,7/15,9/18,8	5,9/4,4/7,3<br>3,1/5,3/9,7	3,4/3/3,7<br>0,5/4,3/4,8
[FB:5] (2) Ich habe mich hilflos und traurig gefühlt.	4/4/4<br>4/4/3	55,4/60,7/50,5<br>68,7/50,7/46,7	21/21,5/20,5<br>15,4/23,7/24,8	9,4/7,4/11,3<br>7,2/10,1/9,7	10,6/8,4/12,5<br>5,6/11,6/15,8	3,7/2/5,2<br>3,1/3,9/3
[FB:6] (3) Ich fühlte mich durch meinen Herzfehler in meiner Selbstständigkeit eingeschränkt.	4/4/4<br>4/4/3	60,8/62,4/59,3<br>79,5/58,9/45,5	20/19,5/20,5<br>10,8/22,2/27,3	8,3/9,1/7,6<br>6,2/9,2/10,3	8,6/7,7/9,5<br>2,6/7,7/13,3	2,2/1,3/3,1<br>1/1,9/3,6
[FB:7] (4) … meine Eltern viele Sorgen gemacht, das tut mir leid.	4/4/4<br>4/4/3	58,6/59,7/57,5<br>70,8/56,5/49,1	16,5/17,8/15,3<br>11,8/18,4/18,8	10,2/9,1/11,3<br>6,7/12,1/11,5	10,1/9,7/10,4<br>7,2/9,7/14,5	4,6/3,7/5,5<br>3,6/3,4/6,1
[FB:8] (5) … in der Schule/Ausbildung Lehrer und Mitschüler mir gegenüber komisch verhalten.	4/4/4<br>4/4/4	76,2/79,5/73,1<br>83,1/74,4/70,3	13,1/13,1/13,1<br>8,2/15,5/17	5,9/5/6,7<br>6,2/4,3/6,7	4/2,3/5,5<br>2,1/4,3/5,5	0,8/…/1,5<br>0,5/1,4/0,6
[FB:10] (6) … meine Eltern und andere Erwachsene übervorsichtig mir gegenüber verhalten.	4/4/4<br>4/4/3	61,1/59,1/63<br>78,5/58,5/43,6	21,6/24,2/19,3<br>10,3/27,5/30,3	6,1/6/6,1<br>3,6/4,3/9,1	8,8/9,4/8,3<br>6,7/5,8/14,5	2,4/1,3/3,4<br>1/3,9/2,4
[FB:11] (7) … Lehrer und Mitschüler besonders um mich bemüht, ohne dass ich das wollte.	4/4/4<br>4/4/4	74,9/75,2/74,6<br>88,2/71,5/67,3	15,2/17,4/13,1<br>6,7/16,9/20,6	5,8/5/6,4<br>2,6/5,3/8,5	3,4/2,3/4,3<br>1,5/5,3/3,6	0,8/…/1,5<br>1/1/…
[FB:12] (8) … Andere über mich lustig gemacht.	4/4/4<br>4/4/4	80,5/79,5/81,3<br>88,2/79,2/74,5	10,6/12,8/8,6<br>5,1/12,1/13,9	4,6/4,4/4,9<br>4,6/2,9/5,5	3,7/3,4/4<br>2,1/4,8/4,8	0,6/…/1,2<br>…/1/1,2
[FB:13] (9) Ich habe viel Unterricht verpasst wegen Untersuchungen.	4/4/4<br>4/4/4	68,2/65,8/70,3<br>83,6/69,6/52,1	21,3/23,8/19<br>11,8/22,7/30,3	2,9/2,7/3,1<br>…/1,9/5,5	5,4/6/4,9<br>2,6/5,8/7,9	2,2/1,7/2,8<br>2,1/…/4,2
[FB:14] (10) Ich war besorgt, welchen Beruf ich mit meinem Herzfehler machen kann.	4/4/4<br>4/4/3	59,4/60,4/58,4<br>79,5/58/38,2	15,4/15,8/15<br>10,8/16,9/20,6	8,3/9,7/7<br>3,6/8,7/11,5	12,5/11,4/13,5<br>4,6/11,1/23,6	4,5/2,7/6,1<br>1,5/5,3/6,1
[FB:15] (11) Ich habe mir Gedanken gemacht, ob ich die Schule/Ausbildung schaffe.	4/4/4<br>4/4/4	61,8/62,4/61,2<br>73,8/59,4/50,3	15,7/18,8/12,8<br>11,3/15,5/22,4	5,8/6,4/5,2<br>3,6/6,8/6,7	11,8/9,1/14,4<br>8,2/11,1/17,6	5/3,4/6,4<br>3,1/7,2/3
[FB:16] (12) Ich habe mir überhaupt keine Sorgen um meine Zukunft gemacht.	3/3/3<br>4/3/2	9,9/7/12,5<br>6,7/8,7/13,3	17,1/18,1/16,2<br>12,3/19,3/21,2	11,5/10,4/12,5<br>9,2/10,6/17,6	22,1/20,5/23,5<br>18,5/25,1/18,2	39,4/44/35,2<br>53,3/36,2/29,7

Angabe des Median und von Häufigkeiten in Prozent: gesamt (N = 625) / männlich (n = 298) / weiblich (n = 327) / simple AHF (n = 195) / moderate AHF (n = 207) / komplexe AHF (n = 165); FB:x = Die Angabe der Nummer der tatsächlichen Frage im Fragebogen

Tabelle 4.3. *Einzelitems der Skala MeHeartRecovery (Jugendliche: N = 625).*

	Median	trifft gar nicht zu	trifft nicht zu	weiß nicht	trifft zu	trifft voll zu
**MeHeartRecovery**						
[FB:31] (1) Ich konnte nicht gut einschlafen.	4/4/3	50,1/57/43,7	26,7/26,5/26,9	7,2/5/9,2	11,4/9,7/12,8	4,6/1,7/7,3
	4/3/3	57,9/44,9/49,7	21/30,4/31,5	7,2/6,8/7,3	10,8/12,1/7,9	3,1/5,8/3,6
[FB:32] (2) Ich bin nachts häufig aufgewacht.	4/4/3	54,6/60,4/49,2	25,9/25,5/26,3	5,4/5/5,8	11,2/7,7/14,4	2,9/1,3/4,3
	4/4/4	63,1/51,7/53,9	20/28,5/26,1	5,6/4,8/4,2	8,2/12,1/14,5	3,1/2,9/1,2
[FB:33] (3) Ich bin morgens schwer aus dem Bett gekommen.	3/3/3	33,3/34,6/32,1	27,5/27,5/27,5	8/8,4/7,6	20,8/20,5/21,1	10,4/9,1/11,6
	3/3/3	37,4/30,9/30,3	25,6/29/32,1	8,2/7,7/7,3	20,5/23,2/18,2	8,2/9,2/12,1
[FB:34] (4) Ich bin erholt aufgewacht.	3/3/3	4,6/3,7/5,5	18,4/13,4/22,9	16/15,1/16,8	35,2/38,3/32,4	25,8/29,5/22,3
	3/3/3	5,1/3,4/3	16,4/21,7/16,4	16,4/14/17,6	31,8/35,3/42,4	30,3/25,6/20,6
[FB:35] (5) Ich hatte einen ruhigen festen Schlaf.	3/3/3	1,6/1/2,1	9,8/8,4/11	13,9/10,1/17,4	35/36,6/33,6	39,7/44/35,8
	3/3/3	1,5/1,9/1,2	6,2/12,1/9,7	16,4/12,1/12,1	28,7/31,4/48,5	47,2/42,5/28,5
[FB:36] (6) Ich habe mich gut nach anstrengenden Tagen erholt.	3/3/3	2,9/1,3/4,3	9/5,7/11,9	13,1/10,1/15,9	34,6/38,3/31,2	40,5/44,6/36,7
	3/3/3	2,6/4,3/1,2	9,2/8,7/6,1	10,8/15/12,7	32,3/30,9/44,8	45,1/41,1/35,2

Angabe des Median und von Häufigkeiten in Prozent: gesamt (N = 625) / männlich (n = 298) / weiblich (n = 327) / simple AHF (n = 195) / moderate AHF (n = 207) / komplexe AHF (n = 165); FB:x = Die Angabe der Nummer der tatsächlichen Frage im Fragebogen

Tabelle 4.4. *Einzelitems der Skala MeHeartFriends (Jugendliche: N = 625).*

	Median	trifft gar nicht zu	trifft nicht zu	weiß nicht	trifft zu	trifft voll zu
**MeHeartFriends**						
[FB:1] (1) Ich fühlte mich genauso selbstständig wie meine Freunde.	4/4/4	4,3/3,7/4,9	6,6/6/7	5,4/5,7/5,2	22,6/18,8/26	61,1/65,8/56,9
	4/4/4	5,1/4,8/3	5,1/6,3/8,5	3,1/2,9/10,3	21,5/21,3/23,6	65,1/64,7/54,5
[FB:2] (2) Ich hatte keine Schwierigkeiten Freunde zu finden.	4/4/4	4,3/5,4/3,4	9/7,7/10,1	8/7/8,9	21/20,5/21,4	57,8/59,4/56,3
	4/4/4	2,1/4,3/6,1	5,1/11,1/7,9	7,7/7,2/9,1	22,1/19,3/23,6	63,1/58/53,3
[FB:3] (3) Ich konnte an Aktivitäten mit Freunden teilnehmen.	4/4/4	1,6/1,3/1,8	4,3/4,7/4	4,2/3,7/4,6	26,2/24,8/27,5	63,7/65,4/62,1
	4/4/4	0,5/2,9/1,8	1,5/4,3/5,5	2,6/4,3/6,1	17,9/26,6/35,2	77,4/61,8/51,5

Angabe des Median und von Häufigkeiten in Prozent: gesamt (N = 625) / männlich (n = 298) / weiblich (n = 327) / simple AHF (n = 195) / moderate AHF (n = 207) / komplexe AHF (n = 165); FB:x = Die Angabe der Nummer der tatsächlichen Frage im Fragebogen

Tabelle 4.5. *Einzelitems der Skala MeHeartTreatment (Jugendliche: N = 625).*

	Median	trifft gar nicht zu	trifft nicht zu	weiß nicht	trifft zu	trifft voll zu
**MeHeartTreatment**						
[FB:38] (1) Vor dem Krankenhaus habe ich keine Angst.	3/3/3	5,9/4/7,6	14,1/11,4/16,5	11,5/10,4/12,5	25,8/27,9/23,9	42,7/46,3/39,4
	3/3/3	4,1/6,8/5,5	10,3/14,5/20	14,4/9,7/10,3	25,1/28/26,1	46,2/41,1/38,2
[FB:40] (2) Ich finde Arztbesuche unangenehm.	3/3/3	31,2/34,9/27,8	33,8/37,9/30	11,4/8,4/14,1	16,5/13,8/19	7,2/5/9,2
	3/3/3	30,8/30,9/30,3	36,9/28,5/37,6	12,8/11,6/7,3	15,4/18,4/17,6	4,1/10,6/7,3
[FB:41] (3) Ich mag es nicht am Herzen untersucht zu werden.	3/3/3	36,6/43/30,9	25,9/27,2/24,8	17,3/16,8/17,7	12,8/10,1/15,3	7,4/3/11,3
	3/3/3	35,9/33,8/42,4	26,2/24,2/27,3	22,6/16,9/10,3	10,3/15,5/10,9	5,1/9,7/9,1

Angabe des Median und von Häufigkeiten in Prozent: gesamt (N = 625) / männlich (n = 298) / weiblich (n = 327) / simple AHF (n = 195) / moderate AHF (n = 207) / komplexe AHF (n = 165); FB:x = Die Angabe der Nummer der tatsächlichen Frage im Fragebogen

Tabelle 4.6. *Einzelitems der Skala MeHeartSchool (Jugendliche: N = 625).*

	Median	trifft gar nicht zu	trifft nicht zu	weiß nicht	trifft zu	trifft voll zu
**MeHeartSchool**						
[FB:17] (1) Ich bin mit den Hausaufgaben gut zurechtgekommen.	4/4/4	2,9/2,7/3,1	8,3/6/10,4	7,7/8,4/7	27,7/30,5/25,1	53,4/52,3/54,4
	4/4/3	3,6/3,4/2,4	6,2/9,7/9,7	8,2/8,2/8,5	23,1/27,5/32,1	59/51,2/47,3
[FB:18] (2) Ich bin mit dem Unterrichtsstoff gut mitgekommen.	3/3/3	2,2/1,7/2,8	7,2/6/8,3	9,4/6,7/11,9	33/38,3/28,1	48,2/47,3/48,9
	4/3/3	4,1/1,9/1,2	6,2/8,2/7,3	7,7/10,6/11,5	24,1/34,8/40,6	57,9/44,4/39,4

Angabe des Median und von Häufigkeiten in Prozent: gesamt (N = 625) / männlich (n = 298) / weiblich (n = 327) / simple AHF (n = 195) / moderate AHF (n = 207) / komplexe AHF (n = 165); FB:x = Die Angabe der Nummer der tatsächlichen Frage im Fragebogen

Tabelle 4.7. *Indikator-Items/Single-Screening-Items (Jugendliche: N = 625).*

	Median	habe keine/ nehme keine	trifft gar nicht zu	trifft nicht zu	weiß nicht	trifft zu	trifft voll zu
[FB:9] (1) ... meine Eltern mir gegenüber anders verhalten als meinen Geschwistern gegenüber.*	4/4/4<br>4/4/4	9,4/9,7/9,2<br>8,2/10,6/7,9	74/76,2/72,1<br>83,2/73,5/67,1	14,1/14,5/13,8<br>8,9/15,7/15,8	4,6/3,7/5,4<br>3,9/4,3/3,9	5,7/4,5/6,7<br>2,8/4,9/10,5	1,6/1,1/2<br>1,1/1,6/2,6
[FB:26] (2) ... abends öfter dicke Beine und Füße.	4/4/4<br>4/4/4		77,4/79,2/75,8<br>80/79,2/75,8	14,1/13,4/14,7<br>12,8/13/16,4	5/4,7/5,2<br>3,6/4,8/4,2	2,9/2,3/3,4<br>3,1/2,9/2,4	0,6/0,3/0,9<br>0,5/…/1,2
[FB:37] (3) Ich weiß gut Bescheid über mein Herz und was ihm fehlt.	3/3/3<br>3/3/3		3,2/3/3,4<br>3,1/1,9/4,2	7/6,4/7,6<br>7,2/7,2/7,9	13,8/12,8/14,7<br>13,8/12,1/13,3	37/39,9/34,3<br>31,3/42,5/37,6	39/37,9/40,1<br>44,6/36,2/37
[FB:39] (4) Ich vertrage meine Medikamente gut.*	4/4/4<br>3/4/4	69,6/66,4/72,5<br>88,2/72,5/44,2	0,5/…/1,1<br>…/…/1,1	1,1/1/1,1<br>…/1,8/1,1	11,6/5/18,9<br>39,1/10,5/3,3	25,8/31/20<br>39,1/33,3/14,1	61,1/63/58,9<br>21,7/54,4/80,4
[FB:42] (5) Ich finde es nervig Medikamente zu nehmen.*	2/3/2<br>2,5/2/2	68,2/64,1/71,9<br>86,7/69,6/43,6	20,6/22,4/18,5<br>11,5/14,3/29	23,6/28/18,5<br>38,5/28,6/17,2	16,1/8,4/25<br>19,2/12,7/16,1	20,6/22,4/18,5<br>23,1/23,8/18,3	19,1/18,7/19,6<br>7,7/20,6/19,4
[FB:43] (6) Ich finde meine Operationsnarbe sehr unangenehm.*	3/3/3<br>4/3/3	37,6/32,9/41,9<br>77,9/26,1/3,6	40/41,5/38,4<br>53,5/39,9/35,2	29,5/34/24,7<br>25,6/30,7/29,6	12,1/9,5/14,7<br>11,6/11,1/13,2	9,5/8/11,1<br>4,7/9,2/11,9	9/7/11,1<br>4,7/9,2/10,1

*Angabe des Median und von Häufigkeiten in Prozent: gesamt (N = 625) / männlich (n = 298) / weiblich (n = 327) / simple AHF (n = 195) / moderate AHF (n = 207) / komplexe AHF (n = 165); *Bei diesen Items beziehen sich die Prozentangaben für die Kategorie „habe keine/nehme keine" auf die Gesamt- bzw. Teilstichproben, die Angaben zu Median und den Ratingkategorien (trifft gar nicht zu – trifft voll zu) beziehen sich entsprechend auf die reduzierten Gesamt- und Teilstichproben; FB:x = Die Angabe der Nummer der tatsächlichen Frage im Fragebogen*

8. SKALENPRÜFUNG

Reliabilitätsprüfung

Die Reliabilität wurde anhand der Split-Half-Reliabilität untersucht. Die 36/31 Items (Kinder/Kindergartenkinder) und die 37 Items (Jugendliche) der konstruierten Skalen wurden zur Berechnung jeweils in zwei Gruppen aufgeteilt. Die ungeraden Items wurden der ersten und die geraden Items der zweiten Testhälfte zugeordnet (odd-even-split).

Bei den 36 Items der sechs konstruierten Skalen der Kinderversion des CHDSI (N = 530) ist Cronbachs Alpha der ersten Testhälfte (18 Items, α = .900) ebenso wie Cronbachs Alpha der zweiten Testhälfte (18 Items, α = .885) als hoch zu bewerten und auch die Split-half-Reliabilität (Spearman-Brown-Koeffizient bei gleicher Testlänge) ist mit .956 als sehr gut zu interpretieren.

Auch bei der auf 31 Items und fünf Skalen reduzierten Version für Kindergartenkinder (N = 46) ist Cronbachs Alpha der ersten Testhälfte (16 Items, α = .856) ebenso wie Cronbachs Alpha der zweiten Testhälfte (15 Items, α = .886) als hoch zu bewerten und auch die Split-half-Reliabilität (Spearman-Brown-Koeffizient bei ungleicher Testlänge) ist mit .964 als sehr gut zu beurteilen.

Die Split-half-Reliabilität (Spearman-Brown-Koeffizient bei ungleicher Testlänge) der 37 Items der sechs konstruierten Skalen der Jugendversion des CHDSI (N = 625) ist mit .966 als sehr gut zu interpretieren. Cronbachs Alpha lag bei α = .890 (erste Testhälfte, 19 Items) bzw. α = .899 (zweite Testhälfte, 18 Items).

Prüfung von Interner Konsistenz, Trennschärfe und Homogenität

Wie Tabelle 5.1 zu entnehmen ist, weisen 31 der 36 Items des Kinderfragebogens mit einem Wert von > .50 eine hohe Trennschärfe auf, die Trennschärfe der drei weiteren Items ist mit einem Wert von > .40 als gut zu bewerten und für die zwei Items der Skale MeHeartSchool konnte keine Trennschärfe berechnet werden, da hierfür mindestens drei Items erforderlich sind. Cronbachs Alpha und die Split-half-Reliabilität für die Skala MeHeartSchool sind mit Werten von .729 bis .911 durchweg als gut bis exzellent zu beurteilen und der MIC liegt mit Werten zwischen .401 und .572 im erwarteten akzeptablen/guten Bereich.

Tabelle 5.1. *Skalengüte Kinder (N = 530).*

	TS	Alpha wIa	Alpha ges	MIC
**MeHeartImpairments**				
[FB:19] (1) Ich konnte alle körperlichen Aktivitäten machen wie ich wollte.	.579	.905		
[FB:20] (2) Ich konnte alles machen ohne außer Puste zu geraten.	.688	.899		
[FB:21] (3) Ich hatte keine körperlichen Schmerzen.	.718	.897		
[FB:22] (4) Ich habe mich selten krank gefühlt.	.652	.901		
[FB:23] (5) Ich konnte problemlos meine Hobbies machen.	.707	.899		
[FB:24] (6) Ich fühlte mich körperlich frisch und munter.	.747	.897	.911	.482
[FB:25] (7) … schnell keine Puste mehr.	.690	.899		
[FB:27] (8) … häufig mit Schwindel zu tun.	.572	.905		
[FB:28] (9) … auch Schmerzen in der Brust.	.583	.904		
[FB:29] (10) … gemerkt, dass ich schnell müde werde.	.663	.900		
[FB:30] (11) … öfter komisches Herzklopfen.	.653	.901		
**MeHeartStigma**				
[FB:4] (1) Ich habe mir Sorgen über meine Zukunft gemacht.	.478	.858		
[FB:5] (2) Ich habe mich hilflos und traurig gefühlt.	.556	.850		
[FB:7] (3) … meine Eltern viele Sorgen gemacht, das tut mir leid.	.591	.848		
[FB:8] (4) … in der Schule Lehrer und Mitschüler mir gegenüber komisch verhalten.	.656	.846		
[FB:10] (5) … meine Eltern und andere Erwachsene übervorsichtig mir gegenüber verhalten.	.610	.846	.862	.401
[FB:11] (6) … Lehrer und Mitschüler besonders um mich bemüht, ohne dass ich das wollte.	.628	.846		
[FB:12] (7) … andere Kinder über mich lustig gemacht.	.586	.849		
[FB:13] (8) Ich habe viel Unterricht verpasst wegen Untersuchungen.	.586	.849		
[FB:14] (9) Ich konnte im Sportunterricht nicht alles mitmachen.	.581	.851		
[FB:15] (10) Ich habe mir Gedanken gemacht, ob ich die Schule schaffe.	.568	.850		
**MeHeartRecovery**				
[FB:31] (1) Ich konnte nicht gut einschlafen.	.619	.824		
[FB:32] (2) Ich bin nachts häufig aufgewacht.	.633	.822		
[FB:33] (3) Ich bin morgens schwer aus dem Bett gekommen.	.505	.852	.847	.494
[FB:34] (4) Ich bin erholt aufgewacht.	.704	.808		
[FB:35] (5) Ich hatte einen ruhigen festen Schlaf.	.719	.805		
[FB:36] (6) Ich habe mich gut nach anstrengenden Tagen erholt.	.651	.822		
**MeHeartFriends**				
[FB:1] (1) Ich fühlte mich im Vergleich mit Freunden gleich gut.	.480	.703		
[FB:2] (2) Ich habe mich wohl gefühlt wie ich bin.	.570	.648	.729	.417
[FB:3] (3) Ich konnte an Aktivitäten mit Freunden teilnehmen.	.581	.639		
[FB:6] (4) Ich hatte Schwierigkeiten Freunde zu finden.	.484	.691		
**MeHeartTreatment**				
[FB:38] (1) Vor dem Krankenhaus habe ich keine Angst.	.566	.809		
[FB:40] (2) Ich finde Arztbesuche unangenehm.	.722	.641	.799	.572
[FB:41] (3) Ich mag es nicht am Herzen untersucht zu werden.	.649	.721		
**MeHeartSchool**				
[FB:16] (1) Ich bin mit dem Unterrichtsstoff gut mitgekommen.		Split-half-Reliabilität		
[FB:17] (2) Ich bin mit den Hausaufgaben gut zurechtgekommen.		.877		

TS = Trennschärfe; Alpha wIa = Alpha, wenn Item ausgeschossen; Alpha ges = Alpha der gesamten Skala; MIC = mittlere Inter-Item-Korrelation); FB:x = Die Angabe der Nummer der tatsächlichen Frage im Fragebogen

Auch die auf 31 Items und 5 Dimensionen reduzierte Fragebogenversion für Kinder mit AHF, die noch nicht die Schule besuchen weisen akzeptable bis sehr gute Werte auf. Wie Tabelle 5.2 zu entnehmen ist, weisen 26 der 31 Items des Kindergarten-Kinder-Fragebogens mit einem Wert von > .50 eine hohe Trennschärfe auf und auch die Trennschärfe der fünf weiteren Items ist mit einem Wert von > .30 als ausreichend hoch zu bewerten. Cronbachs Alpha ist mit Werten von .773 bis .911 durchweg als gut bis exzellent zu beurteilen und der MIC liegt mit Werten zwischen .358 und .587 im erwarteten akzeptablen/guten Bereich.

Tabelle 5.2. *Skalengüte Kindergartenkinder (N = 46).*

	TS	Alpha wIa	Alpha ges	MIC
**MeHeartImpairments**				
FB:19 (1) Ich konnte alle körperlichen Aktivitäten machen wie ich wollte.	.668	.902		
FB:20 (2) Ich konnte alles machen ohne außer Puste zu geraten.	.772	.897		
FB:21 (3) Ich hatte keine körperlichen Schmerzen.	.752	.898		
FB:22 (4) Ich habe mich selten krank gefühlt.	.690	.901		
FB:23 (5) Ich konnte problemlos meine Hobbies machen.	.708	.900		
FB:24 (6) Ich fühlte mich körperlich frisch und munter.	.740	.900	.911	.487
FB:25 (7) … schnell keine Puste mehr.	.649	.904		
FB:27 (8) … häufig mit Schwindel zu tun.	.503	.911		
FB:28 (9) … auch Schmerzen in der Brust.	.524	.909		
FB:29 (10) … gemerkt, dass ich schnell müde werde.	.698	.901		
FB:30 (11) … öfter komisches Herzklopfen.	.609	.905		
**MeHeartStigma**				
FB:4 (1) Ich habe mir Sorgen über meine Zukunft gemacht.	,571	,755		
FB:5 (2) Ich habe mich hilflos und traurig gefühlt.	,601	,749		
FB:7 (3) … meine Eltern viele Sorgen gemacht, das tut mir leid.	,412	,787		
FB:8 (4) … in der Schule Lehrer und Mitschüler mir gegenüber komisch verhalten.*	,573	,756	.792	.358
FB:10 (5) … meine Eltern und andere Erwachsene übervorsichtig mir gegenüber verhalten.	,424	,784		
FB:11 (6) … Lehrer und Mitschüler besonders um mich bemüht, ohne dass ich das wollte.*	,665	,742		
FB:12 (7) … andere Kinder über mich lustig gemacht.	,436	,780		
**MeHeartRecovery**				
FB:31 (1) Ich konnte nicht gut einschlafen.	,600	,746		
FB:32 (2) Ich bin nachts häufig aufgewacht.	,662	,730		
FB:33 (3) Ich bin morgens schwer aus dem Bett gekommen.	,330	,827	.792	.427
FB:34 (4) Ich bin erholt aufgewacht.	,674	,734		
FB:35 (5) Ich hatte einen ruhigen festen Schlaf.	,538	,761		
FB:36 (6) Ich habe mich gut nach anstrengenden Tagen erholt.	,630	,757		
**MeHeartFriends**				
FB:1 (1) Ich fühlte mich im Vergleich mit Freunden gleich gut.	,771	,608		
FB:2 (2) Ich habe mich wohl gefühlt wie ich bin.	,534	,756	.773	.483
FB:3 (3) Ich konnte an Aktivitäten mit Freunden teilnehmen.	,689	,653		
FB:6 (4) Ich hatte Schwierigkeiten Freunde zu finden.	,407	,823		
**MeHeartTreatment**				
FB:38 (1) Vor dem Krankenhaus habe ich keine Angst.	,648	,754		
FB:40 (2) Ich finde Arztbesuche unangenehm.	,772	,617	.810	.587
FB:41 (3) Ich mag es nicht am Herzen untersucht zu werden.	,570	,825		

*TS = Trennschärfe; Alpha wIa = Alpha, wenn Item ausgeschossen; Alpha ges = Alpha der gesamten Skala; MIC = mittlere Inter-Item-Korrelation); *Die Kinder wurden bei diesen Fragen gebeten, „Schule“ durch Kindergarten, „Lehrer“ durch „Erzieher“ und „Mitschüler“ durch „andere Kindergartenkinder“ zu ersetzen, sofern sie noch nicht zur Schule gehen; FB:x = Die Angabe der Nummer der tatsächlichen Frage im Fragebogen*

Wie Tabelle 5.3 zu entnehmen ist, weisen 32 der 37 Items der Jugendversion des CHDSI mit einem Wert von > .50 eine hohe Trennschärfe auf, die Trennschärfe der drei weiteren Items ist mit einem Wert von > .40 als gut zu bewerten und für die zwei Items der Skale MeHeartSchool konnte keine Trennschärfe berechnet werden, da hierfür mindestens drei Items erforderlich sind. Cronbachs Alpha und die Split-half-Reliabilität für die Skala MeHeartSchool sind mit Werten von .683 bis .914 durchweg als akzeptabel/gut, in vier Fällen sogar als sehr gut und in einem Fall als exzellent zu beurteilen und der MIC liegt mit Werten zwischen .409 und .587 im erwarteten akzeptablen/guten Bereich.

Tabelle 5.3. *Skalengüte Jugendliche (N = 625).*

	TS	Alpha wIa	Alpha ges	MIC
**MeHeartImpairments**				
[FB:19] (1) Ich konnte alle körperlichen Aktivitäten machen wie ich wollte.	.666	.906		
[FB:20] (2) Ich konnte alles machen ohne außer Puste zu geraten.	.746	.902		
[FB:21] (3) Ich hatte keine körperlichen Schmerzen.	.681	.905		
[FB:22] (4) Ich habe mich selten krank gefühlt.	.619	.908		
[FB:23] (5) Ich konnte problemlos meine Hobbies machen.	.644	.908		
[FB:24] (6) Ich fühlte mich körperlich frisch und munter.	.772	.901	.914	.495
[FB:25] (7) … schnell keine Puste mehr.	.686	.905		
[FB:27] (8) … häufig mit Schwindel zu tun.	.580	.910		
[FB:28] (9) … auch Schmerzen in der Brust.	.646	.907		
[FB:29] (10) … gemerkt, dass ich schnell müde werde.	.677	.906		
[FB:30] (11) … öfter komisches Herzklopfen.	.653	.907		
**MeHeartStigma**				
[FB:4] (1) Ich habe mich gefragt, ob ich mit meinem Herzfehler eine/n feste/n Freund/in finde.	.599	.878		
[FB:5] (2) Ich habe mich hilflos und traurig gefühlt.	.627	.877		
[FB:6] (3) Ich fühlte mich durch meinen Herzfehler in meiner Selbstständigkeit eingeschränkt.	.705	.872		
[FB:7] (4) … meine Eltern viele Sorgen gemacht, das tut mir leid.	.514	.884		
[FB:8] (5) … in der Schule/Ausbildung Lehrer und Mitschüler mir gegenüber komisch verhalten.	.621	.878	.888	.409
[FB:10] (6) … meine Eltern und andere Erwachsene übervorsichtig mir gegenüber verhalten.	.625	.877		
[FB:11] (7) … Lehrer und Mitschüler besonders um mich bemüht, ohne dass ich das wollte.	.570	.881		
[FB:12] (8) … Andere über mich lustig gemacht.	.559	.881		
[FB:13] (9) Ich habe viel Unterricht verpasst wegen Untersuchungen.	.494	.884		
[FB:14] (10) Ich war besorgt, welchen Beruf ich mit meinem Herzfehler machen kann.	.665	.874		
[FB:15] (11) Ich habe mir Gedanken gemacht, ob ich die Schule/Ausbildung schaffe.	.693	.872		
[FB:16] (12) Ich habe mir überhaupt keine Sorgen um meine Zukunft gemacht.	.531	.884		
**MeHeartRecovery**				
[FB:31] (1) Ich konnte nicht gut einschlafen.	.651	.841		
[FB:32] (2) Ich bin nachts häufig aufgewacht.	.652	.841		
[FB:33] (3) Ich bin morgens schwer aus dem Bett gekommen.	.583	.858	.863	.523
[FB:34] (4) Ich bin erholt aufgewacht.	.719	.828		
[FB:35] (5) Ich hatte einen ruhigen festen Schlaf.	.672	.838		
[FB:36] (6) Ich habe mich gut nach anstrengenden Tagen erholt.	.699	.833		
**MeHeartFriends**				
[FB:1] (1) Ich fühlte mich genauso selbstständig wie meine Freunde.	.489	.601		
[FB:2] (2) Ich hatte keine Schwierigkeiten Freunde zu finden.	.490	.607	.683	.429
[FB:3] (3) Ich konnte an Aktivitäten mit Freunden teilnehmen.	.534	.565		
**MeHeartTreatment**				
[FB:38] (1) Vor dem Krankenhaus habe ich keine Angst.	.578	.820		
[FB:40] (2) Ich finde Arztbesuche unangenehm.	.747	.645	.810	.587
[FB:41] (3) Ich mag es nicht am Herzen untersucht zu werden.	.658	.740		
**MeHeartSchool**				
[FB:17] (1) Ich bin mit den Hausaufgaben gut zurechtgekommen.		Split-half-Reliabilität		
[FB:18] (2) Ich bin mit dem Unterrichtsstoff gut mitgekommen.		.887		

TS = Trennschärfe; Alpha wIa = Alpha, wenn Item ausgeschossen; Alpha ges = Alpha der gesamten Skala; MIC = mittlere Inter-Item-Korrelation); FB:x = Die Angabe der Nummer der tatsächlichen Frage im Fragebogen

Gruppenunterschiede auf Gesamtscore- und Subskalen-Ebene

Für die Analyse möglicher Mittelwert-/Gruppenunterschiede auf Gesamtscore- und Subskalen-Ebene wurde entschieden, auch beim Vorliegen der Verletzung der Normalverteilung den t-Test zu verwenden. Dies stellt in der wissenschaftlichen Praxis ein übliches Vorgehen dar, sofern die zu vergleichenden Stichproben ausreichend groß sind. So liefert der t-Test beispielsweise beim Vorliegen sehr großer Effekte auch bei minimalen Stichprobenumfängen von 5 und Verletzung der Normalverteilung noch brauchbare Ergebnisse[132]. Zudem konnten Studien vielfach belegen, dass der t-Test als robust gegenüber einer Verletzung der Normalverteilung gelten kann[133-135], wenn die Stichprobe für jede der zu vergleichenden Gruppen entsprechend dem zentralen Grenzwertsatz > 30 ist[136-138]. Hierbei ist darauf hinzuweisen, dass häufig fälschlicherweise davon ausgegangen wird, dass die erhobenen Daten selbst normalverteilt sein müssen, was jedoch nicht korrekt ist. Vielmehr geht

es um die Normalverteilung der Residuen, also um die Normalverteilung der Differenz zwischen vorhergesagten und beobachteten Werten (Residual-Normalverteilung). Bei normalverteilten Daten sind die Residuen zwar stets normalverteilt, umgekehrt ist dies jedoch nicht zwingend der Fall. Abweichungen von der Residual-Normalverteilung haben allerdings oft keinen großen Einfluss auf die Analyseergebnisse[139]. Bühner und Ziegler beurteilen eine Verletzung der Normalverteilung der Residuen ab 100 Fällen als eher unproblematisch[140].

Mittelwertunterschiede Kinderversion

Bei den Kindern (N = 530; 248 männlich; 282 weiblich; 119 simple AHF; 194 moderate AHF; 173 komplexe AHF) unterscheiden sich die Summenscores weder bei der Gesamt-DsQoL noch bei einer der sechs Subskalen des CHDSI signifikant.

Patienten mit simplem und mit moderatem AHF unterscheiden sich signifikant im DsQoL-Gesamtscore ($p < .01$; Mittelwert simple AHF = 121,35±20,77; Mittelwert moderate AHF = 114±23,20) sowie in der Skala MeHeartImpairments ($p < .05$; Mittelwert simple AHF = 36,99±8,68; Mittelwert moderate AHF = 34,76±8,85), der Skala MeHeartStigma ($p < .001$; Mittelwert simple AHF = 36,26±5,29; Mittelwert moderate AHF = 33,45±6,90), der Skala MeHeartFriends ($p < .05$; Mittelwert simple AHF = 14,19±2,50; Mittelwert moderate AHF = 13,56±2,79) und der Skala MeHeartSchool ($p < .01$; Mittelwert simple AHF = 6,98±1,64; moderate AHF = 6,39±1,93).

Patienten mit simplem und mit komplexem AHF unterscheiden sich signifikant im DsQoL-Gesamtscore ($p < .001$; Mittelwert simple AHF = 121,35±20,77; Mittelwert komplexe AHF = 107,09±23,65) sowie in der Skala MeHeartImpairments ($p < .001$; Mittelwert simple AHF = 36,99±8,68; Mittelwert komplexe AHF = 31,93±9,29), der Skala MeHeartStigma ($p < .001$; Mittelwert simple AHF = 36,26±5,29; Mittelwert komplexe AHF = 31,27±7,16), der Skala MeHeartRecovery ($p < .01$; Mittelwert simple AHF = 18,60±5,18; Mittelwert komplexe AHF = 16,94±5,26), der Skala MeHeartFriends ($p < .001$; Mittelwert simple AHF = 14,19±2,50; Mittelwert komplexe AHF = 12,89±3,22) und der Skala MeHeartSchool ($p < .01$; Mittelwert simple AHF = 6,98±1,64; komplexe AHF = 6,29±1,97).

Patienten mit moderatem und mit komplexem AHF unterscheiden sich signifikant im DsQoL-Gesamtscore ($p < .01$; Mittelwert moderate AHF = 114±23,20; Mittelwert komplexe AHF = 107,09±23,65) sowie in der Skala MeHeartImpairments ($p < .01$; Mittelwert moderate AHF = 34,76±8,85; Mittelwert komplexe AHF = 31,93±9,29), der Skala MeHeartStigma ($p < .01$; Mittelwert moderate AHF = 33,45±6,90; Mittelwert komplexe AHF = 31,27±7,16) und der Skala MeHeartFriends ($p < .05$; Mittelwert moderate AHF = 13,56±2,79; Mittelwert komplexe AHF = 12,89±3,22).

Mittelwertunterschiede Jugendliche

Bei den Jugendlichen (N = 625; 298 männlich; 327 weiblich; 195 simple AHF; 207 moderate AHF; 165 komplexe AHF) unterscheiden sich die Summenscores zwischen Patientinnen und Patienten signifikant bei der Gesamt-DsQoL ($p < .001$; Mittelwert Patienten = 118,40±22,43; Mittelwert Patientinnen = 109,32±26,46), bei der Skala MeHeartImpairments ($p < .001$; Mittelwert Patienten = 34,57±8,70; Mittelwert Patientinnen = 30,18±10,50), bei der Skala MeHeartStigma ($p < .05$; Mittelwert Patienten = 40,17±8,01; Mittelwert Patientinnen = 38,79±9,41), bei der Skala MeHeartRecovery ($p < .001$; Mittelwert Patienten = 18,31±4,81; Mittelwert Patientinnen = 16,53±5,84) sowie bei der Skala MeHeartTreatment ($p < .001$; Mittelwert Patienten = 8,82±2,98; Mittelwert Patientinnen = 7,68±3,40).

Patienten mit simplem und mit moderatem AHF unterscheiden sich signifikant im DsQoL-Gesamtscore ($p < .001$; Mittelwert simple AHF = 120,70±22,27; Mittelwert moderate AHF = 113,09±24,30) sowie in der Skala MeHeartStigma ($p < .001$; Mittelwert simple AHF = 42,81±7,25; Mittelwert moderate AHF = 39,11±8,74) und der Skala MeHeartFriends ($p < .05$; Mittelwert simple AHF = 10,46±1,96; Mittelwert moderate AHF = 9,90±2,66).

Patienten mit simplem und mit komplexem AHF unterscheiden sich signifikant im DsQoL-Gesamtscore ($p < .001$; Mittelwert simple AHF = 120,70±22,27; Mittelwert komplexe AHF = 108,38±25,11) sowie in der Skala MeHeartImpairments ($p < .001$; Mittelwert simple AHF = 34,36±9,53; Mittelwert komplexe AHF = 30,48±9,79), der Skala MeHeartStigma ($p < .001$; Mittelwert simple AHF = 42,81±7,25; Mittelwert komplexe AHF = 36,46±9,09) und in der Skala MeHeartFriends ($p < .001$; Mittelwert simple AHF = 10,46±1,96; Mittelwert komplexe AHF = 9,58±2,64).

Patienten mit moderatem und mit komplexem AHF unterscheiden sich signifikant in der Skala MeHeartImpairments ($p < .05$; Mittelwert moderate AHF = 32,79±9,44; Mittelwert komplexe AHF = 30,48±9,79) sowie in der Skala MeHeartStigma ($p < .01$; Mittelwert moderate AHF = 39,11±8,74; Mittelwert komplexe AHF = 36,46±9,09).

Korrelationsanalysen

Es wurde überprüft, ob das Patientenalter, die Anzahl der Geschwisterkinder, das elterliche Berufs-/Bildungsniveau sowie das subjektiv angegebene Wissen über den eigenen Herzfehler einen signifikanten Zusammenhang mit der Gesamt-DsQoL und/oder einer der Subskalen aufweisen. Um einen möglichen Zusammenhang zwischen dem DsQoL-Gesamtscore sowie den Subskalen mit dem Berufs-/Bildungsniveau der Eltern zu untersuchen, wurden die Antwortmöglichkeiten der Variablen Schulabschluss, Berufsabschluss und Erwerbssituation für Mütter und Väter zu je drei Kategorien zusammengefasst (Schulabschluss: kein Schulabschluss = 0, niedriger Schulabschluss = 1, hoher Schulabschluss = 2; Berufsabschluss: kein Berufsabschluss = 0, nicht-akademischer Berufsabschluss = 1, akademischer Berufsabschluss = 2; Erwerbssituation: nicht erwerbstätig = 0, Teilzeit erwerbstätig = 1, Vollzeit erwerbstätig = 2), anschließend wurden alle sechs Einzelwerte addiert (Punktbereich 0-12) und in drei Gruppen unterteilt (niedriges Berufs-/Bildungsniveau: 0-7 Punkte; mittleres Berufs-/Bildungsniveau: 8-9 Punkte; hohes Berufs-/Bildungsniveau: 10-12 Punkte). Insgesamt lagen für 855 Elternpaare die zur Scoreberechnung erforderlichen Angaben vor (350 Kinderstichprobe, 34 Kindergartenstichprobe 471 Jugendstichprobe). Die Kindergartenkinder finden in den nachfolgenden Korrelationsanalysen aufgrund der geringen Fallzahl keine Berücksichtigung. Die 350 Eltern der Kinder mit bekanntem Berufs-/Bildungsniveau wiesen in 40,6 % ein niedriges, in 37,4 % ein mittleres und in 22 % ein hohes Berufs-/Bildungsniveau auf. Bei den 471 Eltern der Jugendlichen mit bekanntem Berufs-/Bildungsniveau lag bei 36,5 % der Elternpaare ein niedriges, in 34 % ein mittleres und in 29,5 % ein hohes Berufs-/Bildungsniveau vor. Es ist zu vermuten, dass der volle (Wieder)Eintritt ins Berufsleben ebenso wie Bildungs- und Berufsabschlüsse in einigen Fällen erst nach dem Kindergarten-/Grundschulalter erfolgen, was das etwas höhere Berufs-/Bildungsniveau der Eltern der Jugendstichprobe erklären könnte.

Korrelationsanalysen Kinderversion

Bei den Kindern (N = 530) lag lediglich ein signifikanter Zusammenhang ($p < .001$; r = .168) zwischen dem Alter des Patienten und dem subjektiven Herzfehlerwissen vor, was durchaus plausibel erscheint, da ältere Kinder auch besser in der Lage sind ihre angeborene Herzerkrankung zu verstehen, sich zu informieren und mit der Erkrankung auseinanderzusetzen (vgl. Abbildung 1). Zwischen der Anzahl der Geschwisterkinder und dem DsQoL-Gesamtscore ($p < .01$; r = -.118) sowie der Skala MeHeartImpairments ($p < .01$; r = -.132) und MeHeartSchool ($p < .05$; r = -.105) liegen leichte negative Zusammenhänge vor. Ein/mehrere Geschwisterkind/er scheinen mit teils niedrigeren Score-Ergebnissen assoziiert zu sein (vgl. Abbildung 2).

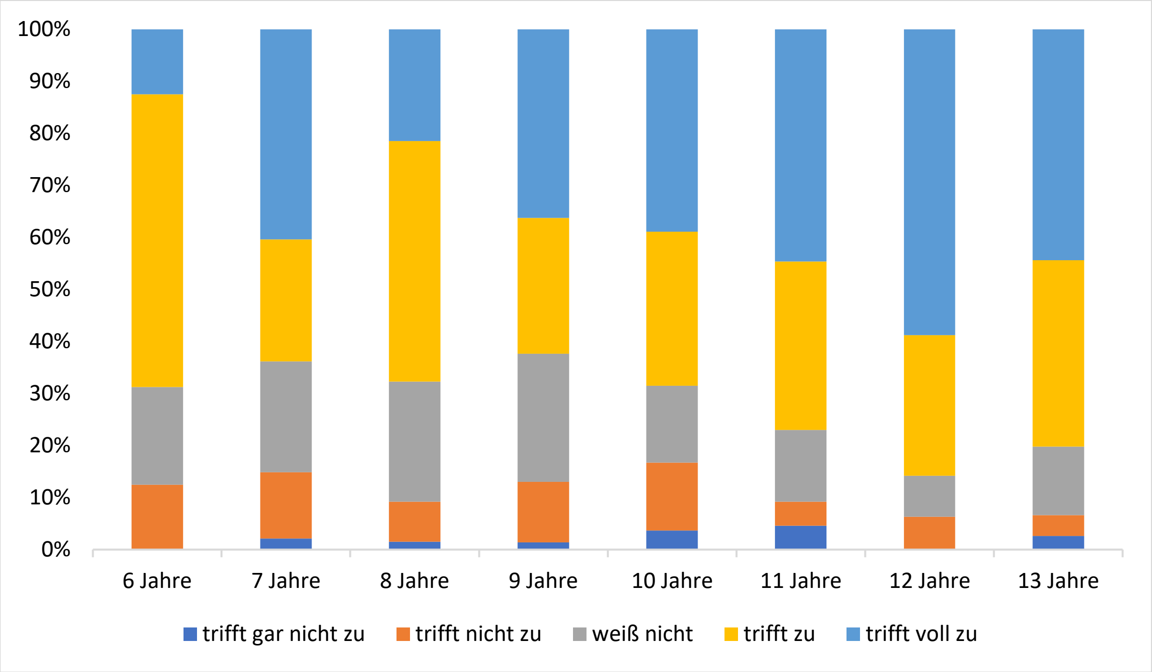

Abbildung 1. *Subjektives Herzfehlerwissen gruppiert nach Alter in Jahren; Kinder (N = 530).*

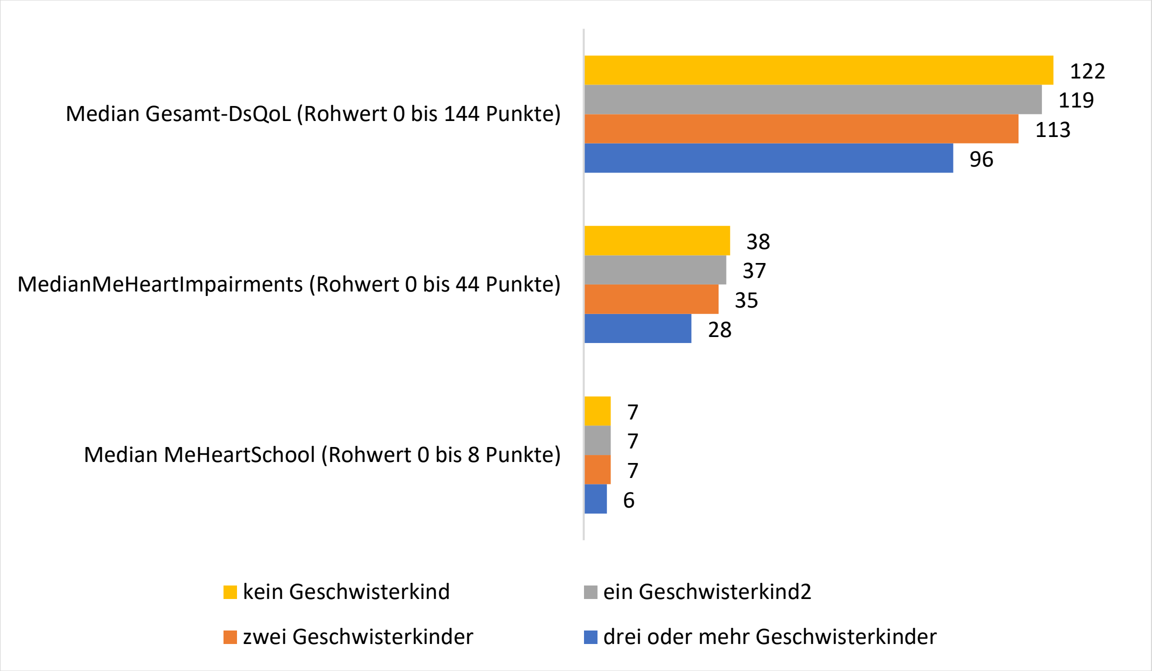

Abbildung 2. *DsQoL = Disease-spezific-Quality-of-Life; Darstellung des Median aufgrund der Korrelation zwischen der Geschwisteranzahl und der Gesamt-DsQoL sowie den Subskalen MeHeartImpairments und MeHeartSchool; Kinder (N = 530).*

Bei den Kindern (N = 350; reduzierte Stichprobe) lag ein leichter signifikanter Zusammenhang zwischen dem elterlichen Berufs-/Bildungsniveau und dem subjektiven Herzfehlerwissen ($p < .01$; r = .159) sowie dem DsQoL-Gesamtscore ($p < .001$; r = .256), der Skala MeHeartImpairments ($p < .001$; r = .265), der Skala MeHeartStigma ($p < .001$; r = .263), der Skala MeHeartRecovery ($p < .01$; r = .162), der Skala MeHeartFriends ($p < .01$; r = .163) und der Skala MeHeartSchool vor ($p < .01$; r = .176). Ein höheres elterliches Berufs-/Bildungsniveau geht mit einer höheren subjektiven Einschätzung des eigenen Herzfehlerwissens (vgl. Abbildung 3) sowie einem höheren DsQoL-Gesamtscore und in fünf der sechs Subskalen auch mit höheren Subskalenscores einher (vgl. Abbildung 4).

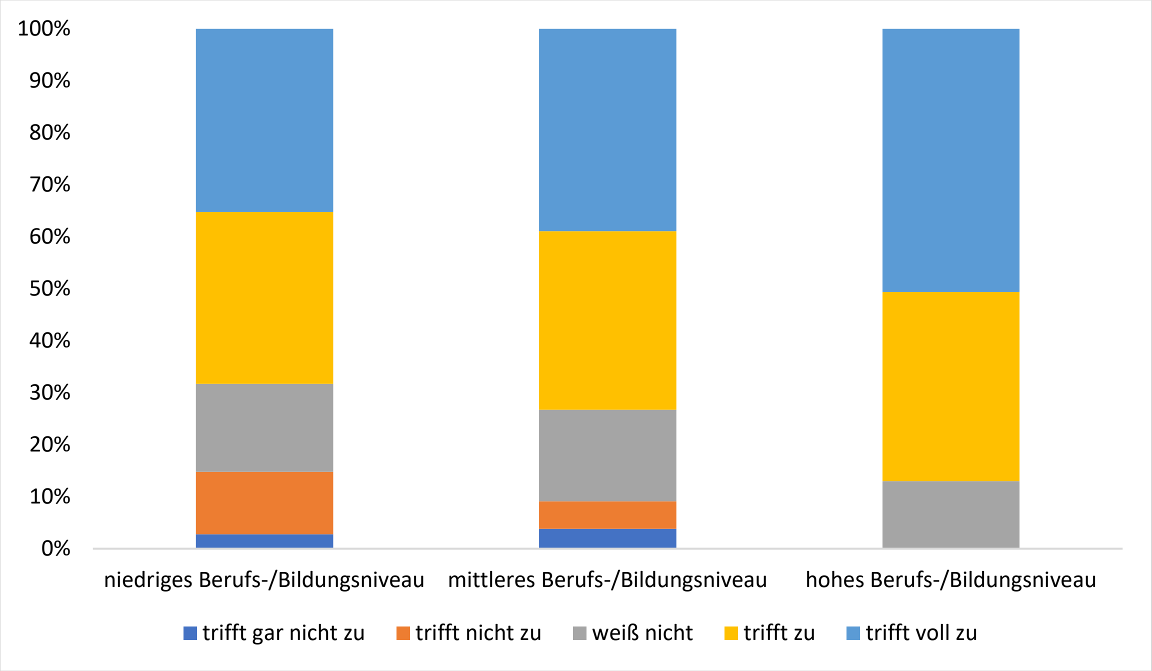

Abbildung 3. *Subjektives Herzfehlerwissen gruppiert nach dem elterlichen Berufs-/Bildungsniveau; Kinder (N = 350).*

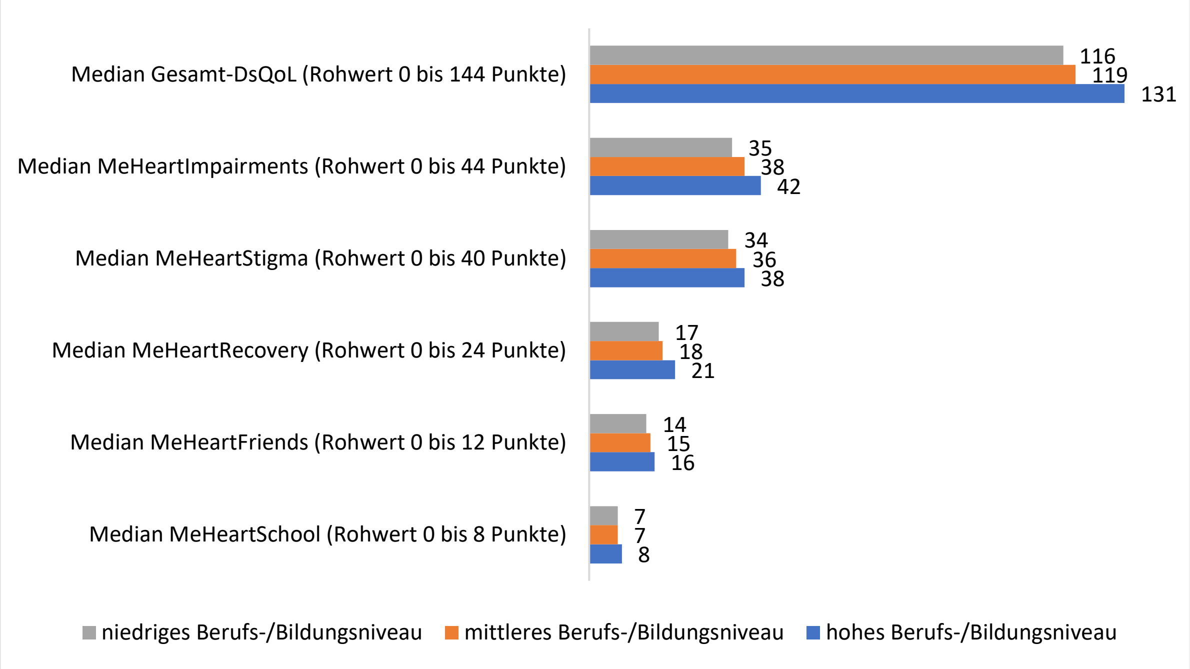

Abbildung 4. *DsQoL = Disease-spezific-Quality-of-Life; Darstellung des Median aufgrund der Korrelation zwischen dem elterlichen Berufs-/Bildungsniveaus und der Gesamt-DsQoL sowie den Subskalen MeHeartImpairments, MeHeartsStigma, MeHeartRecovery, MeHeartFriends und MeHeartSchool; Kinder (N = 350).*

Bei den Kindern (N = 530) lagen leichte bis mittlere signifikante Zusammenhänge zwischen dem subjektiven Herzfehlerwissen dem DsQoL-Gesamtscore ($p < .001$; r = .275), der Skala MeHeartImpairments ($p < .001$; r = .196), der Skala MeHeartStigma ($p < .001$; r = .162), der Skala MeHeartRecovery ($p < .001$; r = .276), der Skala MeHeartFriends ($p < .001$; r = .231), der Skala MeHeartTreatment ($p < .001$; r = .326) und der Skala MeHeartSchool ($p < .001$; r = .225) vor. Ein subjektiv höheres Herzfehlerwissen ist auch mit besseren Ergebnissen in der Gesamt-DsQoL sowie allen Subskalen assoziiert (vgl. Abbildung 5).

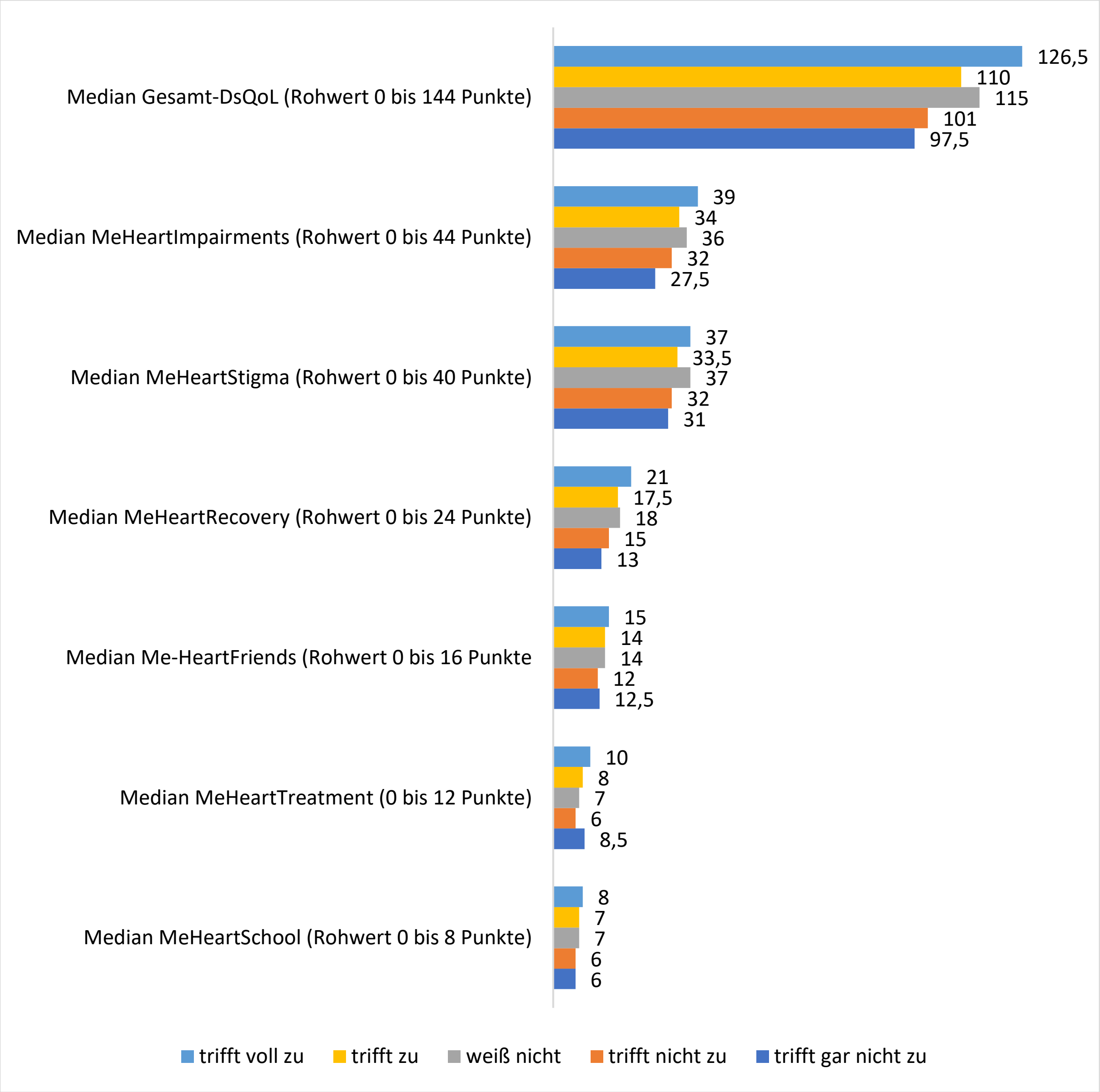

Abbildung 5. *DsQoL = Disease-spezific-Quality-of-Life; Darstellung des Median aufgrund der Korrelation zwischen dem subjektiven Herzfehlerwissen und der Gesamt-DsQoL sowie den Subskalen MeHeartImpairments, MeHeartsStigma, MeHeartRecovery, MeHeartFriends, MeHeartTreatment und MeHeartSchool; Kinder (N = 530).*

Korrelationsanalysen Jugendversion

Bei den Jugendlichen (N = 625) lag lediglich ein leichter negativer signifikanter Zusammenhang ($p < .01$; r = -.135) zwischen dem Alter des Patienten und der Subskala MeHeartStigma vor, was durchaus plausibel erscheint, da sich ältere Jugendliche bereits häufiger mit stigmatisierenden Ereignissen konfrontiert sahen und sich vermutlich bereits eingehender mit den Einschränkungen und Herausforderungen ihrer angeborenen Herzerkrankung auseinandersetzen mussten, was in geringem

Maß mit einem niedrigeren Scoring in der Subskala MeHeartStigma (Punktbereich 0-48 Punkte) führen kann (Median der 14jährigen = 44 Punkte; Median der 15jährigen = 43 Punkte; Median der 16 und 17 Jahre alten Patienten = 41). Zwischen der Anzahl der Geschwisterkinder und dem DsQoL-Gesamtscore oder einer der Subskalen lag in keinem Fall ein signifikanter Zusammenhang vor und das elterliche Berufs-/Bildungsniveau (N = 471; reduzierte Teilstichprobe) weist nur mit der Subskala MeHeartSchool einen leichten signifikanten Zusammenhang ($p < .001$; r = 170) auf (Punktbereich 0-8; Median niedriges Berufs-/Bildungsniveau = 6; Median mittleres Berufs-/Bildungsniveau = 7; Median hohes Berufs-/Bildungsniveau = 8).

Bei den Jugendlichen (N = 625) lagen leichte signifikante Zusammenhänge zwischen dem subjektiven Herzfehlerwissen und dem DsQoL-Gesamtscore ($p < .001$; r = .289), der Skala MeHeartImpairments ($p < .001$; r = .234), der Skala MeHeartStigma ($p < .001$; r = .184), der Skala MeHeartRecovery ($p < .001$; r = .240), der Skala MeHeartFriends ($p < .001$; r = .251), der Skala MeHeartTreatment ($p < .001$; r = .267) und der Skala MeHeartSchool vor ($p < .001$; r = .245). Ein subjektiv höheres Herzfehlerwissen scheint auch mit besseren Ergebnissen in der Gesamt-DsQoL sowie allen sechs Subskalen assoziiert zu sein (vgl. Abbildung 6).

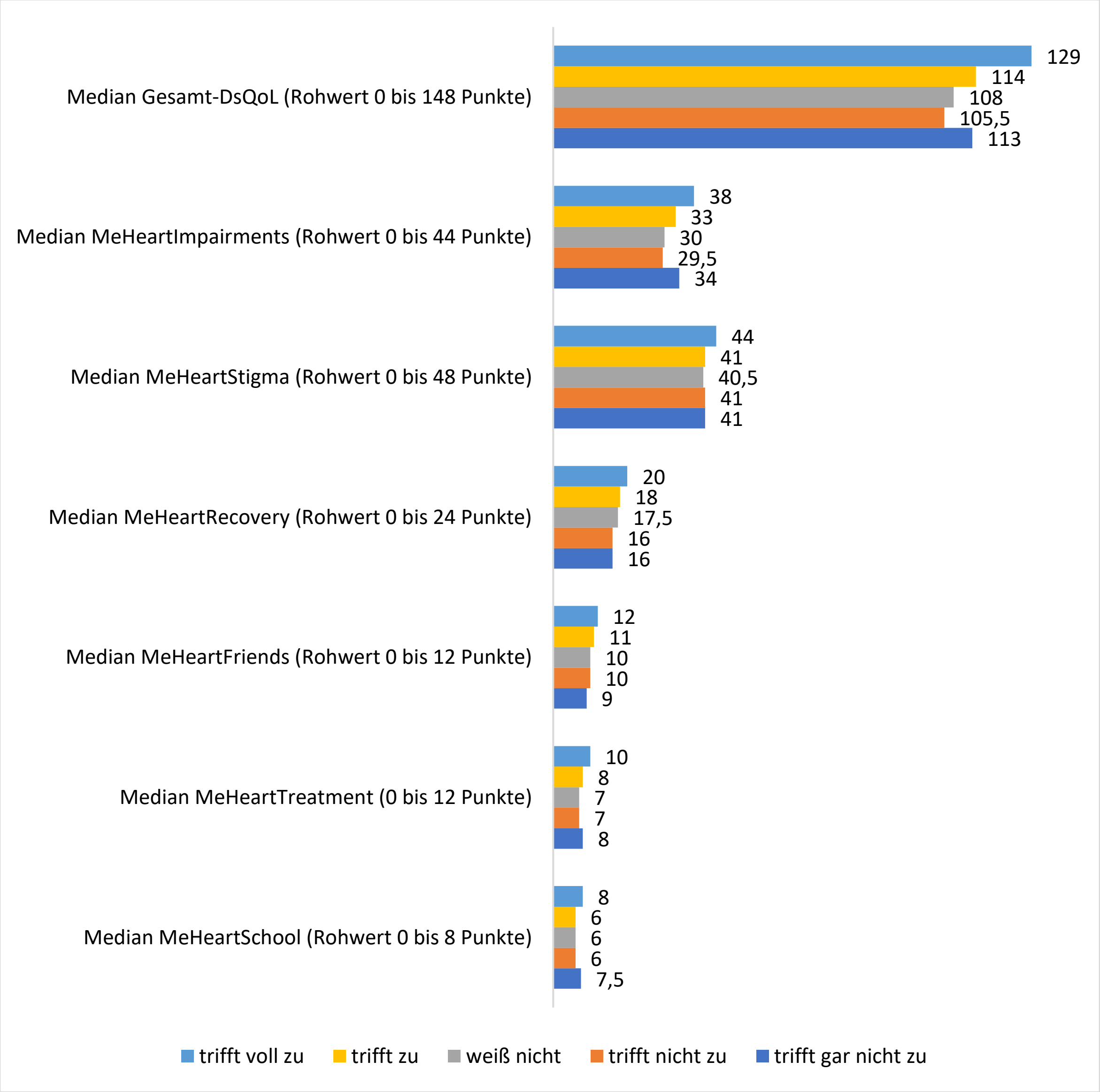

Abbildung 6. *DsQoL = Disease-spezific-Quality-of-Life; Darstellung des Median aufgrund der Korrelation zwischen dem subjektiven Herzfehlerwissen und der Gesamt-DsQoL sowie den Subskalen MeHeartImpairments, MeHeartsStigma, MeHeartRecovery, MeHeartFriends, MeHeartTreatment und MeHeartSchool; Jugendliche (N = 625).*

9. MODELLPRÜFUNG

Modellprüfung der Kinderversion des CHDSI

Um zu prüfen, ob sich die sechs postulierten Subskalen der Kinderversion des CHDSI bestätigen lassen, wurde eine CFA durchgeführt. Wie aus Tabelle 6.1 ersichtlich ist, fiel der χ^2-Test signifikant aus (χ^2 = 1276,050; df = 579; p < .001) und legte somit eine Ablehnung des hier spezifizierten Modells nahe. Allerdings können, wie bereits beschrieben, z. B. ein großer Stichprobenumfang und eine starke Abweichung von der Normalität zu so einer Ablehnung des Modells führen, obwohl es richtig spezifiziert ist. Der relative/normierte χ^2 (χ^2/df) lag mit einem Wert von 2,2 im guten Bereich und eine Ablehnung des Modells lediglich auf Grundlage eines signifikanten χ^2 scheint folglich nicht gerechtfertigt. Die Fit-Indizes zur Beurteilung der approximativen Modellpassung (CFI, TLI, RMSEA) lagen mit einem CFI und TLI von jeweils .993 und einem RMSEA von .048 in sehr guten Bereichen und auch die Fit-Indizes der robusten Modellschätzung waren durchweg als gut bis sehr gut zu bewerten. Alle Faktorladungen lagen mit Werten von .626 bis .932 im akzeptablen bis sehr guten Bereich (siehe Tabelle 6.2). Für die Kinderversion des CHDSI sprachen somit alle zur Beurteilung der Modellpassung herangezogenen Fit-Indizes wie auch der relative/normierte χ^2 für eine gute Passung des spezifizierten Modells.

Tabelle 6.1. *Modellgüte und Schätzparameter der CFA (Kinderversion des CHDSI; N = 530).*

Estimator	DWLS	
Optimization method	NLMINB	
Number of model parameters	195	
Number of observations	530	
**Model Test User Model:**		
	Standard	***Robust***
Test Statistic	1276.050	1492.876
Degrees of freedom	579	579
P-value (Chi-square)	<.001	<.001
Scaling correction factor		1.072
Shift parameter		302.352
**Model Test Baseline Model:**		
	Standard	***Robust***
Test statistic	103271.246	23869.066
Degrees of freedom	630	630
P-value	<.001	<.001
Scaling correction factor		4.417
**User Model versus Baseline Model:**		
	Standard	***Robust***
Comparative Fit Index (CFI)	0.993	0.961
Tucker-Lewis Index (TLI)	0.993	0.957
**Root Mean Square Error of Approximation:**		
	Standard	***Robust***
RMSEA	0.048	0.055
90 Percent confidence interval – lower	0.044	0.051
90 Percent confidence interval – upper	0.051	0.058
P-value RMSEA <= 0.05	0.855	0.013
**Standardized Root Mean Square Residual:**		
	Standard	***Robust***
SRMR	0.052	0.052
**Parameter Estimates:**		
Standard errors	Robust.sem	
Information	Expected	
Information saturated (h1) model	Unstructured	

Tabelle 6.2. *Faktorladungen der CFA: Item-Zuordnung zu latenten Konstrukten (Kinderversion des CHDSI; N = 530).*

	Estimate	Std.Err	z-value	P(>|z|)	Std.lv	Std.all
**MeHeartImpairments**						
[FB:19] (1) Ich konnte alle körperlichen Aktivitäten machen wie ich wollte.	0.751	0.025	30.548	<.001	0.751	0.751
[FB:20] (2) Ich konnte alles machen ohne außer Puste zu geraten.	0.770	0.021	35.915	<.001	0.770	0.770
[FB:21] (3) Ich hatte keine körperlichen Schmerzen.	0.818	0.020	40.754	<.001	0.818	0.818
[FB:22] (4) Ich habe mich selten krank gefühlt.	0.786	0.021	37.640	<.001	0.786	0.786
[FB:23] (5) Ich konnte problemlos meine Hobbies machen.	0.874	0.017	50.985	<.001	0.874	0.874
[FB:24] (6) Ich fühlte mich körperlich frisch und munter.	0.894	0.014	64.725	<.001	0.894	0.894
[FB:25] (7) … schnell keine Puste mehr.	0.793	0.022	35.404	<.001	0.793	0.793
[FB:27] (8) … häufig mit Schwindel zu tun.	0.706	0.030	23.911	<.001	0.706	0.706
[FB:28] (9) … auch Schmerzen in der Brust.	0.704	0.028	25.481	<.001	0.704	0.704
[FB:29] (10) … gemerkt, dass ich schnell müde werde.	0.779	0.023	34.525	<.001	0.779	0.779
[FB:30] (11) … öfter komisches Herzklopfen.	0.783	0.023	34.768	<.001	0.783	0.783
**MeHeartStigma**						
[FB:4] (1) Ich habe mir Sorgen über meine Zukunft gemacht.	0.626	0.034	18.276	<.001	0.626	0.626
[FB:5] (2) Ich habe mich hilflos und traurig gefühlt.	0.723	0.029	25.291	<.001	0.723	0.723
[FB:7] (3) … meine Eltern viele Sorgen gemacht, das tut mir leid.	0.686	0.029	23.773	<.001	0.686	0.686
[FB:8] (4) … in der Schule Lehrer und Mitschüler mir gegenüber komisch verhalten.	0.824	0.026	32.040	<.001	0.824	0.824
[FB:10] (5) … meine Eltern und andere Erwachsene übervorsichtig mir gegenüber verhalten.	0.750	0.026	28.949	<.001	0.750	0.750
[FB:11] (6) … Lehrer und Mitschüler besonders um mich bemüht, ohne dass ich das wollte.	0.775	0.025	30.385	<.001	0.775	0.775
[FB:12] (7) … andere Kinder über mich lustig gemacht.	0.780	0.030	26.207	<.001	0.780	0.780
[FB:13] (8) Ich habe viel Unterricht verpasst wegen Untersuchungen.	0.792	0.027	29.892	<.001	0.792	0.792
[FB:14] (9) Ich konnte im Sportunterricht nicht alles mitmachen.	0.833	0.023	36.934	<.001	0.833	0.833
[FB:15] (10) Ich habe mir Gedanken gemacht, ob ich die Schule schaffe.	0.734	0.029	25.212	<.001	0.734	0.734
**MeHeartRecovery**						
[FB:31] (1) Ich konnte nicht gut einschlafen.	0.747	0.028	26.737	<.001	0.747	0.747
[FB:32] (2) Ich bin nachts häufig aufgewacht.	0.814	0.024	33.662	<.001	0.814	0.814
[FB:33] (3) Ich bin morgens schwer aus dem Bett gekommen.	0.631	0.032	19.920	<.001	0.631	0.631
[FB:34] (4) Ich bin erholt aufgewacht.	0.783	0.025	30.783	<.001	0.783	0.783
[FB:35] (5) Ich hatte einen ruhigen festen Schlaf.	0.869	0.021	41.055	<.001	0.869	0.869
[FB:36] (6) Ich habe mich gut nach anstrengenden Tagen erholt.	0.844	0.024	35.429	<.001	0.844	0.844
**MeHeartFriends**						
[FB:1] (1) Ich fühlte mich im Vergleich mit Freunden gleich gut.	0.682	0.029	23.866	<.001	0.682	0.682
[FB:2] (2) Ich habe mich wohl gefühlt wie ich bin.	0.804	0.025	32.340	<.001	0.804	0.804
[FB:3] (3) Ich konnte an Aktivitäten mit Freunden teilnehmen.	0.841	0.024	35.595	<.001	0.841	0.841
[FB:6] (4) Ich hatte Schwierigkeiten Freunde zu finden.	0.679	0.035	19.548	<.001	0.679	0.679
**MeHeartTreatment**						
[FB:38] (1) Vor dem Krankenhaus habe ich keine Angst.	0.738	0.034	21.409	<.001	0.738	0.738
[FB:40] (2) Ich finde Arztbesuche unangenehm.	0.883	0.023	38.416	<.001	0.883	0.883
[FB:41] (3) Ich mag es nicht am Herzen untersucht zu werden.	0.832	0.028	30.202	<.001	0.832	0.832
**MeHeartSchool**						
[FB:16] (1) Ich bin mit dem Unterrichtsstoff gut mitgekommen.	0.932	0.021	44.212	<.001	0.932	0.932
[FB:17] (2) Ich bin mit den Hausaufgaben gut zurechtgekommen.	0.924	0.017	54.641	<.001	0.924	0.924

FB:x = Die Angabe der Nummer der tatsächlichen Frage im Fragebogen

Tabelle 6.3. *Kovarianzen zwischen latenten Faktoren der CFA (Kinderversion des CHDSI; N = 530).*

	Estimate	Std.Err	z-value	P(>|z|)	Std.lv	Std.all
**MeHeartImpairments**						
MeHeartStigma	0.816	0.020	40.702	<.001	0.816	0.816
MeHeartRecovry	0.725	0.023	31.544	<.001	0.725	0.725
MeHeartFriends	0.842	0.020	42.802	<.001	0.842	0.842
MeHeartTreatment	0.434	0.040	10.907	<.001	0.434	0.434
MeHeartSchool	0.551	0.034	16.019	<.001	0.551	0.551
**MeHeartStigma**						
MeHeartRecovry	0.626	0.030	20.815	<.001	0.626	0.626
MeHeartFriends	0.851	0.022	38.092	<.001	0.851	0.851
MeHeartTreatment	0.477	0.040	11.918	<.001	0.477	0.477
MeHeartSchool	0.608	0.035	17.437	<.001	0.608	0.608
**MeHeartRecovery**						
MeHeartFriends	0.659	0.033	20.121	<.001	0.659	0.659
MeHeartTreatment	0.501	0.037	13.554	<.001	0.501	0.501
MeHeartSchool	0.555	0.035	15.936	<.001	0.555	0.555
**MeHeartFriends**						
MeHeartTreatment	0.527	0.043	12.230	<.001	0.527	0.527
MeHeartSchool	0.597	0.039	15.318	<.001	0.597	0.597
**MeHeartTreatment**						
MeHeartSchool	0.372	0.045	8.275	<.001	0.372	0.372

Modellprüfung der Jugendversion des CHDSI

Um zu prüfen, ob sich die sechs postulierten Subskalen der Jugendversion des CHDSI bestätigen lassen, wurde eine CFA durchgeführt. Wie in Tabelle 7.1 dargestellt, fiel der χ^2-Test signifikant aus (χ^2 = 1580,638; df = 614; p < .001) und legte somit eine Ablehnung des hier spezifizierten Modells nahe. Allerdings liegt der relative/normierte χ^2 (χ^2/df) mit einem Wert von 2,6 im guten Bereich. Eine Ablehnung des Modells lediglich auf Grundlage eines signifikanten χ^2 scheint nicht gerechtfertigt. Die Fit-Indizes zur Beurteilung der approximativen Modellpassung (CFI, TLI, RMSEA) lagen mit einem CFI von .992, einem TLI von .991 und einem RMSEA von .05 in sehr guten bis guten Bereichen und auch die Fit-Indizes der robusten Modellschätzung waren durchweg im guten bis sehr guten Bereich. Alle Faktorladungen lagen mit Werten von .614 bis .939 im guten bis sehr guten Bereich (siehe Tabelle 7.2). Für die Jugendversion des CHDSI sprachen somit alle zur Beurteilung der Modellpassung herangezogenen Fit-Indizes wie auch der relative/normierte χ^2 für eine gute Passung des spezifizierten Modells.

Tabelle 7.1. *Modellgüte und Schätzparameter der CFA (Jugendversion des CHDSI; N = 625).*

Estimator	DWLS	
Optimization method	NLMINB	
Number of model parameters	200	
Number of observations	625	
**Model Test User Model:**		
	Standard	***Robust***
Test Statistic	1580.638	1806.846
Degrees of freedom	614	614
P-value (Chi-square)	<.001	<.001
Scaling correction factor		1.061
Shift parameter		316.623
**Model Test Baseline Model:**		
	Standard	***Robust***
Test statistic	120589.394	27437.643
Degrees of freedom	666	666
P-value	<.001	<.001
Scaling correction factor		4.479
**User Model versus Baseline Model:**		
	Standard	***Robust***
Comparative Fit Index (CFI)	0.992	0.955
Tucker-Lewis Index (TLI)	0.991	0.952
**Root Mean Square Error of Approximation:**		
	Standard	***Robust***
RMSEA	0.050	0.056
90 Percent confidence interval – lower	0.047	0.053
90 Percent confidence interval – upper	0.053	0.059
P-value RMSEA <= 0.05	0.446	0.001
**Standardized Root Mean Square Residual:**		
	Standard	***Robust***
SRMR	0.054	0.054
**Parameter Estimates:**		
Standard errors	Robust.sem	
Information	Expected	
Information saturated (h1) model	Unstructured	

Tabelle 7.2. *Faktorladungen der CFA: Item-Zuordnung zu latenten Konstrukten (Jugendversion des CHDSI; N = 625).*

	Estimate	Std.Err	z-value	P(>|z|)	Std.lv	Std.all
**MeHeartImpairments**						
FB:19 (1) Ich konnte alle körperlichen Aktivitäten machen wie ich wollte.	0.839	0.018	46.087	<.001	0.839	0.839
FB:20 (2) Ich konnte alles machen ohne außer Puste zu geraten.	0.841	0.015	56.082	<.001	0.841	0.841
FB:21 (3) Ich hatte keine körperlichen Schmerzen.	0.787	0.021	37.530	<.001	0.787	0.787
FB:22 (4) Ich habe mich selten krank gefühlt.	0.748	0.023	32.831	<.001	0.748	0.748
FB:23 (5) Ich konnte problemlos meine Hobbies machen.	0.849	0.020	42.741	<.001	0.849	0.849
FB:24 (6) Ich fühlte mich körperlich frisch und munter.	0.868	0.013	64.990	<.001	0.868	0.868
FB:25 (7) … schnell keine Puste mehr.	0.801	0.017	45.882	<.001	0.801	0.801
FB:27 (8) … häufig mit Schwindel zu tun.	0.680	0.028	24.727	<.001	0.680	0.680
FB:28 (9) … auch Schmerzen in der Brust.	0.702	0.024	28.800	<.001	0.702	0.702
FB:29 (10) … gemerkt, dass ich schnell müde werde.	0.797	0.020	39.515	<.001	0.797	0.797
FB:30 (11) … öfter komisches Herzklopfen.	0.747	0.023	32.166	<.001	0.747	0.747
**MeHeartStigma**						
FB:4 (1) Ich habe mich gefragt, ob ich mit meinem Herzfehler eine/n feste/n Freund/in finde.	0.718	0.028	25.840	<.001	0.718	0.718
FB:5 (2) Ich habe mich hilflos und traurig gefühlt.	0.802	0.022	36.319	<.001	0.802	0.802
FB:6 (3) Ich fühlte mich durch meinen Herzfehler in meiner Selbstständigkeit eingeschränkt.	0.854	0.019	44.888	<.001	0.854	0.854
FB:7 (4) ... meine Eltern viele Sorgen gemacht, das tut mir leid.	0.629	0.033	19.006	<.001	0.629	0.629
FB:8 (5) ... in der Schule/Ausbildung Lehrer und Mitschüler mir gegenüber komisch verhalten.	0.788	0.028	28.561	<.001	0.788	0.788
FB:10 (6) ... meine Eltern und andere Erwachsene übervorsichtig mir gegenüber verhalten.	0.727	0.025	28.564	<.001	0.727	0.727
FB:11 (7) ... Lehrer und Mitschüler besonders um mich bemüht, ohne dass ich das wollte.	0.743	0.030	25.079	<.001	0.743	0.743
FB:12 (8) ... Andere über mich lustig gemacht.	0.755	0.031	24.580	<.001	0.755	0.755
FB:13 (9) Ich habe viel Unterricht verpasst wegen Untersuchungen.	0.659	0.033	19.818	<.001	0.659	0.659
FB:14 (10) Ich war besorgt, welchen Beruf ich mit meinem Herzfehler machen kann.	0.760	0.024	31.964	<.001	0.760	0.760
FB:15 (11) Ich habe mir Gedanken gemacht, ob ich die Schule/Ausbildung schaffe.	0.848	0.020	42.917	<.001	0.848	0.848
FB:16 (12) Ich habe mir überhaupt keine Sorgen um meine Zukunft gemacht.	0.710	0.026	27.276	<.001	0.710	0.710
**MeHeartRecovery**						
FB:31 (1) Ich konnte nicht gut einschlafen.	0.778	0.024	32.950	<.001	0.778	0.778
FB:32 (2) Ich bin nachts häufig aufgewacht.	0.850	0.020	43.323	<.001	0.850	0.850
FB:33 (3) Ich bin morgens schwer aus dem Bett gekommen.	0.717	0.024	29.478	<.001	0.717	0.717
FB:34 (4) Ich bin erholt aufgewacht.	0.776	0.020	38.229	<.001	0.776	0.776
FB:35 (5) Ich hatte einen ruhigen festen Schlaf.	0.797	0.020	39.958	<.001	0.797	0.797
FB:36 (6) Ich habe mich gut nach anstrengenden Tagen erholt.	0.863	0.018	47.994	<.001	0.863	0.863
**MeHeartFriends**						
FB:1 (1) Ich fühlte mich genauso selbstständig wie meine Freunde.	0.726	0.031	23.622	<.001	0.726	0.726
FB:2 (2) Ich hatte keine Schwierigkeiten Freunde zu finden.	0.614	0.036	16.949	<.001	0.614	0.614
FB:3 (3) Ich konnte an Aktivitäten mit Freunden teilnehmen.	0.905	0.023	38.621	<.001	0.905	0.905
**MeHeartTreatment**						
FB:38 (1) Vor dem Krankenhaus habe ich keine Angst.	0.798	0.036	22.386	<.001	0.798	0.798
FB:40 (2) Ich finde Arztbesuche unangenehm.	0.927	0.025	36.913	<.001	0.927	0.927
FB:41 (3) Ich mag es nicht am Herzen untersucht zu werden.	0.765	0.031	24.955	<.001	0.765	0.765
**MeHeartSchool**						
FB:17 (1) Ich bin mit den Hausaufgaben gut zurechtgekommen.	0.931	0.019	48.861	<.001	0.931	0.931
FB:18 (2) Ich bin mit dem Unterrichtsstoff gut mitgekommen.	0.939	0.019	49.094	<.001	0.939	0.939

FB:x = Die Angabe der Nummer der tatsächlichen Frage im Fragebogen

Tabelle 7.3. *Kovarianzen zwischen latenten Faktoren der CFA (Jugendversion des CHDSI; N = 625).*

	Estimate	Std.Err	z-value	P(>|z|)	Std.lv	Std.all
**MeHeartImpairments**						
MeHeartStigma	0.765	0.021	35.677	<.001	0.765	0.765
MeHeartRecovry	0.742	0.021	35.231	<.001	0.742	0.742
MeHeartFriends	0.749	0.026	28.585	<.001	0.749	0.749
MeHeartTreatment	0.347	0.038	9.029	<.001	0.347	0.347
MeHeartSchool	0.509	0.032	15.772	<.001	0.509	0.509
**MeHeartStigma**						
MeHeartRecovry	0.643	0.029	22.532	<.001	0.643	0.643
MeHeartFriends	0.799	0.024	33.422	<.001	0.799	0.799
MeHeartTreatment	0.431	0.037	11.803	<.001	0.431	0.431
MeHeartSchool	0.610	0.030	20.300	<.001	0.610	0.610
**MeHeartRecovery**						
MeHeartFriends	0.598	0.034	17.619	<.001	0.598	0.598
MeHeartTreatment	0.371	0.040	9.380	<.001	0.371	0.371
MeHeartSchool	0.534	0.031	17.411	<.001	0.534	0.534
**MeHeartFriends**						
MeHeartTreatment	0.363	0.046	7.838	<.001	0.363	0.363
MeHeartSchool	0.585	0.034	16.963	<.001	0.585	0.585
**MeHeartTreatment**						
MeHeartSchool	0.314	0.043	7.369	<.001	0.314	0.314

10. NORMWERTE FÜR GESAMTSCORE UND SUBSKALEN

Die Normierung des CHDSI wurde anhand einer Stichprobe von 1.201 Personen (576 Kinder und 625 Jugendliche mit AHF im Alter zwischen 6 und < 18 Jahren) in ganz Deutschland onlinebasiert durchgeführt. Die Datenerhebung erfolgte im zweiten Quartal 2022. Das Geschlechtsverhältnis der 1.201 Patienten war annähernd gleich (52 % weiblich), die Kinder waren zum Befragungszeitpunkt zwischen 6 und < 14 Jahren alt (Mittelwert = 10,14±2,41; Minimum = 6; Maximum = 13) und die Jugendlichen waren zwischen 14 und < 18 Jahren alt (Mittelwert = 15,17±1,07; Minimum = 14; Maximum = 17). Der CHDSI ist somit nicht geeignet für Personen, die fünf Jahre oder jünger bzw. 18 Jahre oder älter sind.

Wie bereits beschrieben kann bei den Kindern ein Rohwert-Gesamtscore zwischen 0 und 144 Punkten (maximal 124 Punkte bei Kindergartenkindern) und bei Jugendlichen zwischen 0 und 148 Punkten erreicht werden. Zur Darstellung des individuellen Levels der DsQoL in Prozent wurde für jede Punktzahl des Rohwert-Gesamtscores ein dazugehöriger Prozentwert berechnet. Es ist also theoretisch möglich, dass eine Person mit 0 % die schlechtestmögliche DsQoL bzw. mit 100 % die bestmögliche DsQoL aufweist, wobei ein Wert von 0 % weder bei Kindern noch bei Jugendlichen gemessen wurde, wohingegen eine bestmögliche DsQoL von 100 % in beiden Stichproben mehrfach erreicht werden konnte.

Die Datenerhebung der Normierungsstichprobe wurde online durchgeführt. Für jede Aussage gaben die Kinder und Jugendlichen mit AHF auf einer fünfstufigen Likert-Skala an, wie gut die verschiedenen Aussagen auf sie zutrafen. Die Antwortmöglichkeiten reichten von „trifft gar nicht zu" bis „trifft voll zu". Die Umrechnung der Rohwerte in standardisierte Werte wurde mit SPSS entsprechend dem Vorgehen nach Tent und Stelzl durchgeführt[141] (Flächentransformation beim Nichtvorliegen einer Normalverteilung), wobei zunächst die Rohwerte in Prozentränge (PR) und dann in Stanine-Werte transformiert wurden.

Die Normierung eines Tests/Fragebogens ermöglicht es, individuelle Scores mit denen einer im Idealfall repräsentativen Normierungsstichprobe zu vergleichen[67], wobei die Normbereiche auf standardisierten/normalisierten Werten basieren. Entsprechend kann eine Einschätzung erfolgen, ob sich ein Patient im Vergleich mit anderen in einem kritischen Bereich der DsQoL befindet oder nicht. Hierbei ist klarzustellen, dass die Daten einer Normierungsstichprobe keinesfalls auch der Normalverteilung folgen müssen. Zwar sind viele natürliche Merkmale normalverteilt, wenn die Stichprobe groß genug ist, häufig liegt aber auch trotz großer Stichprobe keine Normalverteilung vor[142].

Die hier gewählte Form der Datentransformation ist die Umrechnung der Punktrohwerte in die sogenannte Stanine-Skala. Diese neunstufige Skala weist eine Normalverteilung mit einem Mittelwert von 5 und einer Standardabweichung von 2 auf. Der Bereich zwischen den Staninen 4 und 6 kann als durchschnittliches Ergebnis interpretiert werden, Werte von 2 oder 3 können als niedrig und ein Wert von 1 als sehr niedrig gelten, während Werte von 7 oder 8 als hoch und der Wert 9 als sehr hohe Ausprägung des Merkmals verstanden werden können[143,144].

Die Stanine-Bereiche wurden auf Grundlage der entsprechenden Prozentrangwerte (4 %, 11 %, 23 %, 40 %, 60 %, 77 %, 89 %, 96 %, 100 %) bestimmt[68,141]. Da trotz durchgeführter Datentransformation teils deutliche Abweichung von der Normalverteilung in den Subskalen bei der Kinder- und der Jugendversion des CHDSI vorliegen, kam es entsprechend vor, dass in einer oder mehreren Stanine-Kategorien keine oder nur wenige Fälle vorlagen. In diesen Fällen führt dies zwangsläufig zu einer Anteilserhöhung in den verbleibenden Stanine-Gruppen. Zudem findet durch die Umwandlung der Rohwerte in Stanine-Werte eine Vereinfachung der Daten statt und eine Reduktion des

Differenzierungsgrades der Werte lässt sich nicht vermeiden. Angesichts dieser Umstände wurde entschieden, die neunstufige Stanine-Skala in drei Bereiche aufzuteilen:

- niedriger Bereich (Stanine 1-3)
- normaler Bereich (Stanine 4-6)
- hoher Bereich (Stanine 7-9)

Im Anschluss an die detaillierte Darstellung der tatsächlich in der Normierungsstichprobe gemessenen Werte (vgl. Tabelle 8.1 und 8.4) leiten wir zwei Normwertbereiche ab:

- kritischer Bereich (Stanine 1-3)
- unkritischer Bereich (Stanine 4-9)

Die Normbereiche geben an, wie groß der Anteil der Personen der vorliegenden Normierungsstichprobe ist, die sich im jeweiligen Bereich befinden. Erzielt z. B. ein AHF-Patient einen nach Normtabelle kritischen DsQoL-Gesamtscore, kann mit hoher Sicherheit auch vom tatsächlichen Vorliegen einer kritischen DsQoL ausgegangen werden.

Der CHDSI ist ausdrücklich nicht als Diagnostik- sondern als Screeninginstrument zu verstehen. Beim Vorliegen einer mit dem CHDSI erhobenen kritischen DsQoL ist eine umfassende psychologische Diagnostik durchzuführen. Bei der Interpretation der Ergebnisse sollte das Augenmerk primär auf dem DsQoL-Gesamtscore liegen, der ein stabiles und umfassendes Maß für die subjektive, individuelle und ganzheitliche DsQoL darstellt. Die konstruierten Subskalen können als Orientierungshilfe bei tiefergehender Auseinandersetzung mit den verschiedenen Teilbereichen der DsQoL herangezogen werden.

Bei den Kindern lag der niedrigste gemessene prozentuale Gesamtscore der DsQoL bei 18,06 %, was einem Rohwert von 26 der 144 erreichbaren Punkte im Gesamtscore entspricht. Maximal wurde mit 100 % (144 Punkte) die höchstmögliche mit dem CHDSI erfassbare DsQoL gemessen. Die drei Stanine-Bereiche für den Gesamtscore und die Subskalen der Kinderversion des CHDSI sind im Detail in Tabelle 8.1 dargestellt. Die daraus abgeleiteten Normbereiche für die Interpretation der Rohpunktwerte von Gesamt-DsQoL und Subskalen sind Tabelle 8.2 zu entnehmen.

Tabelle 8.1. *Stanine-Bereiche im Detail Kinder (N = 530).*

	Reduzierte Stanine-Skala			Gemessene Werte in der Normierungsstichprobe	
	PR	*Stanine*	*Prozent*	*Stichprobenanteil in %*	*Rohwert-Punktzahl (zugehöriger Prozentwert)*
**DsQoL-Gesamtscore (0 – 144 Punkte)**					
niedriger Bereich	0 – 23	1-3	23 %	22,5/23,4/22,7<br>23,5/22,7/23,1	26-95(18,06-65,97 %)/36-96(25-66,67 %)/26-95(18,06-65,97 %)<br>57-108(39,58-75 %)/36-96(25-66,67 %)/26-89(18,06-61,81 %)
normaler Bereich	> 23 – 77	4-6	54 %	55,1/54/53,9<br>52,9/54,1/53,8	96-133(66,67-92,36 %)/98-132(68,06-91,67 %)/96-133(66,67-92,36 %)<br>109-136(75,69-94,44 %)/97-133(67,36-92,36 %)/90-126(62,5-87,5 %)
hoher Bereich	> 77 – 100	7-9	23 %	22,5/22,6/23,4<br>23,5/23,2/23,1	134-144(93,06-100 %)/133-144(92,36-100 %)/134-144(93,06-100 %)<br>137-144(95,14-100 %)/134-144(93,06-100 %)/127-144(88,19-100 %)
**MeHeartImpairments-Score (0 – 44 Punkte)**					
niedriger Bereich	0 – 23	1-3	23 %	23,2/22,2/21,6<br>21,8/22,7/23,7	2-27(4,55-61,36 %)/3-26(6,82-59,09 %)/2-27(4,55-61,36 %)<br>2-29(4,55-65,91 %)/7-28(15,91-63,64 %)/3-24(6,82-54,55 %)
normaler Bereich	> 23 – 77	4-6	54 %	53,4/57,7/52,1<br>47,9/54,6/54,3	28-42(63,64-95,45 %)/27-42(61,36-95,45 %)/28-42(63,64-95,45 %)<br>30-43(68,18-97,73 %)/29-42(65,91-95,45 %)/25-41(56,82-93,18 %)
hoher Bereich	> 77 – 100	7-9	23 %	23,4/20,2/26,2<br>30,3/22,7/22	43-44(97,73-100 %)/43-44(97,73-100 %)/43-44(97,73-100 %)<br>44(100 %)/43-44(97,73-100 %)/42-44(95,45-100 %)
**MeHeartStigma-Score (0 – 40 Punkte)**					
niedriger Bereich	0 – 23	1-3	23 %	22,5/21,4/23,4<br>21,8/23,7/22	7-28(17,5-70 %)/13-28(32,5-70 %)/7-28(17,5-70 %)<br>14-34(35-85 %)/7-29(17,5-72,5 %)/9-24(22,5-60 %)
normaler Bereich	> 23 – 77	4-6	54 %	58,9/52,4/55,3<br>47,1/55,7/56,1	29-39(72,5-97,5 %)/29-38(72,5-95 %)/29-39(72,5-97,5 %)<br>35-39(87,5-97,5 %)/30-39(75-97,5 %)/25-37(62,5-92,5 %)
hoher Bereich	> 77 – 100	7-9	23 %	18,7/26,2/21,3<br>31,1/20,6/22	40(100 %)/39-40(97,5-100 %)/40(100 %)<br>40(100 %)/40(100 %)/38-40(95-100 %)
**MeHeartRecovery-Score (0 – 24 Punkte)**					
niedriger Bereich	0 – 23	1-3	23 %	20,6/24,6/22<br>20,2/24,7/23,1	1-13(4,17-54,17 %)/1-14(4,17-58,33 %)/1-13(4,17-54,17 %)<br>3-14(12,5-58,33 %)/1-14(4,17-58,33 %)/1-13(4,17-54,17 %)
normaler Bereich	> 23 – 77	4-6	54 %	57/52,8/55,7<br>58/50,5/54,3	14-22(58,33-91,67 %)/15-22(62,5-91,67 %)/14-22(58,33-91,67 %)<br>15-23(62,5-95,83 %)/15-22(62,5-91,67 %)/14-21(58,33-87,5 %)
hoher Bereich	> 77 – 100	7-9	23 %	22,5/22,6/22,3<br>21,8/24,7/22,5	23-24(95,83-100 %)/23-24(95,83-100 %)/23-24(95,83-100 %)<br>24(100 %)/23-24(95,83-100 %)/22-24(91,67-100 %)
**MeHeartFriends-Score (0 – 16 Punkte)**					
niedriger Bereich	0 – 23	1-3	23 %	23,2/25,4/21,3<br>21/20,1/22,5	1-11(6,25-68,75 %)/3-11(18,75-68,75 %)/1-11(6,25-68,75 %)<br>5-12(31,25-75 %)/3-11(18,75-68,75 %)/1-10(6,25-62,5 %)
normaler Bereich	> 23 – 77	4-6	54 %	42,6/41,1/44<br>34,5/43,8/51,4	12-15(75-93,75 %)/12-15(75-93,75 %)/12-15(75-93,75 %)<br>13-15(81,25-93,75 %)/12-15(75-93,75 %)/11-15(68,75-93,75 %)
hoher Bereich	> 77 – 100	7-9	23 %	34,2/33,5/34,8<br>44,5/36,1/26	16(100 %)/16(100 %)/16(100 %)<br>16(100 %)/16(100 %)/16(100 %)
**MeHeartTreatment-Score (0 – 12 Punkte)**					
niedriger Bereich	0 – 23	1-3	23 %	23,6/24,6/22,7<br>21/23,7/24,9	0-5(0-41,67 %)/0-5(0-41,67 %)/0-5(0-41,67 %)<br>0-5(0-41,67 %)/0-5(0-41,67 %)/0-5(0-41,67 %)
normaler Bereich	> 23 – 77	4-6	54 %	49,8/55,6/54,3<br>57,1/48,5/52	6-10(50-83,33 %)/6-11(50-91,67 %)/6-10(50-83,33 %)<br>6-11(50-91,67 %)/6-10(50-83,33 %)/6-10(50-83,33 %)
hoher Bereich	> 77 – 100	7-9	23 %	26,6/19,8/23<br>21,8/27,8/23,1	11-12(91,67-100 %)/12(100 %)/11-12(91,67-100 %)<br>12(100 %)/11-12(91,67-100 %)/11-12(91,67-100 %)
**MeHeartSchool-Score (0 – 8 Punkte)**					
niedriger Bereich	0 – 23	1-3	23 %	19,6/23/16,7<br>30,3/21,6/23,1	0-5(0-62,5 %)/0-5(0-62,5 %)/0-5(0-62,5 %)<br>0-6(0-75 %)/0-5(0-62,5 %)/0-5(0-62,5 %)
normaler Bereich	> 23 – 77	4-6	54 %	34,3/34,3/83,3<br>69,7/35,1/37,6	6-7(75-87,5 %)/6-7(75-87,5 %)/6-8(75-100 %)<br>7-8(87,5-100%)/6-7(75-87,5 %)/6-7(75-87,5 %)
hoher Bereich	> 77 – 100	7-9	23 %	46/42,7/…<br>…/43,3/39,3	8(100 %)/8(100 %)/…<br>…/8(100 %)/8(100 %)

DsQoL = Disease-spezific-Quality-of-Life; PR = Prozentrang; gesamt (N = 530) / männlich (n = 248) / weiblich (n = 282) / simple AHF (n = 119) / moderate AHF (n = 194) / komplexe AHF (n = 173)

Tabelle 8.2. *Normtabelle Kinder (N = 530).*

	gesamt	männlich	weiblich	simple AHF	moderate AHF	komplexe AHF
DsQoL-Gesamtscore (0-144 Punkte)						
kritischer Bereich	0 – 95	0 – 96	0 – 95	0 – 108	0 – 96	0 – 89
unkritischer Bereich	96 – 144	97 – 144	96 – 144	109 – 144	97 – 144	90 – 144
MeHeartImpairments (0-44 Punkte)						
kritischer Bereich	0 – 27	0 – 26	0 – 27	0 – 29	0 – 28	0 – 24
unkritischer Bereich	28 – 44	27 – 44	28 – 44	30 – 44	29 – 44	25 – 44
MeHeartStigma (0-40 Punkte)						
kritischer Bereich	0 – 28	0 – 28	0 – 28	0 – 34	0 – 29	0 – 24
unkritischer Bereich	29 – 40	29 – 40	29 – 40	35 – 40	30 – 40	25 – 40
MeHeartRecovery (0-24 Punkte)						
kritischer Bereich	0 – 13	0 – 14	0 – 13	0 – 14	0 – 14	0 – 13
unkritischer Bereich	14 – 24	15 – 24	14 – 24	15 – 24	15 – 24	14 – 24
MeHeartFriends (0-16 Punkte)						
kritischer Bereich	0 – 11	0 – 11	0 – 11	0 – 12	0 – 11	0 – 10
unkritischer Bereich	12 – 16	12 – 16	12 – 16	13 – 16	12 – 16	11 – 16
MeHeartTreatment (0-12 Punkte)						
kritischer Bereich	0 – 5	0 – 5	0 – 5	0 – 5	0 – 5	0 – 5
unkritischer Bereich	6 – 12	6 – 12	6 – 12	6 – 12	6 – 12	6 – 12
MeHeartSchool (0-8 Punkte)						
kritischer Bereich	0 – 5	0 – 5	0 – 5	0 – 6	0 – 5	0 – 5
unkritischer Bereich	6 – 8	6 – 8	6 – 8	7 – 8	6 – 8	6 – 8

DsQoL = Disease-spezific-Quality-of-Life; gesamt (N = 530), männlich (n = 248), weiblich (n = 282), simple AHF (n = 119), moderate AHF (n = 194) / komplexe AHF (n = 173)

Bei den Kindergartenkindern lag der niedrigste gemessene prozentuale Gesamtscore der DsQoL bei 34,68 %, was einem Rohwert von 43 der 124 erreichbaren Punkte im Gesamtscore entspricht. Maximal wurde mit 99,19 % (123 Punkte) die höchste DsQoL gemessen. Aufgrund der geringen Fallzahl (N = 46) ist eine detaillierte Darstellung der drei Stanine-Bereiche sowie eine Aufteilung dieser Teilstichprobe nach Geschlecht oder Schweregrad des AHF nicht sinnvoll, so dass lediglich Vorschläge für die zwei Normbereiche (kritischer bzw. unkritischer Bereich) als Interpretationshilfe der Rohwertpunkte von Gesamt-DsQoL und Subskalen entsprechend Tabelle 8.3 vertretbar erscheinen.

Tabelle 8.3. *Normtabelle Kindergartenkinder (N = 46).*

	gesamt
**DsQoL-Gesamtscore (0-124 Punkte)**	
kritischer Bereich	0 – 84
unkritischer Bereich	85 – 124
**MeHeartImpairments (0-44 Punkte)**	
kritischer Bereich	0 – 28
unkritischer Bereich	29 – 44
**MeHeartStigma (0-28 Punkte)**	
kritischer Bereich	0 – 18
unkritischer Bereich	19 – 28
**MeHeartRecovery (0-24 Punkte)**	
kritischer Bereich	0 – 15
unkritischer Bereich	16 – 24
**MeHeartFriends (0-16 Punkte)**	
kritischer Bereich	0 – 11
unkritischer Bereich	12 – 16
**MeHeartTreatment (0-12 Punkte)**	
kritischer Bereich	0 – 4
unkritischer Bereich	5 – 12

DsQoL = Disease-spezific-Quality-of-Life; gesamt (N = 46)

Bei den Jugendlichen lag der niedrigste gemessene prozentuale Gesamtscore der DsQoL bei 15,54 %, was einem Rohwert von 23 der 148 erreichbaren Punkte im Gesamtscore entspricht. Maximal wurde mit 100 % (148 Punkte) die höchstmögliche mit dem CHDSI erfassbare DsQoL gemessen. Die drei Stanine-Bereiche für den Gesamtscore und die Subskalen der Jugendversion des CHDSI sind im Detail in Tabelle 8.4 dargestellt. Die daraus abgeleiteten Normbereiche für die Interpretation der Rohpunktwerte von Gesamt-DsQoL und Subskalen sind Tabelle 8.5 zu entnehmen.

Tabelle 8.4. *Stanine-Bereiche im Detail Jugendliche (N = 625).*

	**Reduzierte Stanine-Skala**			**Gemessene Werte in der Normierungsstichprobe**	
	PR	*Stanine*	*Prozent*	*Stichprobenanteil in %*	*Rohwert-Punktzahl (zugehöriger Prozentwert)*
**DsQoL-Gesamtscore (0 – 148 Punkte)**					
niedriger Bereich	0 – 23	1-3	23 %	22,7/22,1/23,2<br>22,1/23,2/23	23-93(15,54-62,84 %)/48-101(32,43-68,24 %)/23-88(15,54-59,46 %)<br>30-107(20,27-72,3 %)/30-93(20,27-62,84 %)/39-87(26,35-58,78 %)
normaler Bereich	> 23 – 77	4-6	54 %	54,2/54,7/53,5<br>54,4/54,6/53,3	94-134(63,51-90,54 %)/102-137(68,92-92,57 %)/89-131(60,14-88,51 %)<br>108-138(72,97-93,24 %)/94-133(63,51-89,86 %)/90-128(60,81-86,49 %)
hoher Bereich	> 77 – 100	7-9	23 %	23/23,2/23,2<br>23,6/22,2/23,6	135-148(91,22-100 %)/138-148(93,24-100 %)/132-148(89,19-100 %)<br>139-148(93,92-100 %)/134-148(90,54-100 %)/130-148(87,84-100 %)
**Impairments-Score (0 – 44 Punkte)**					
niedriger Bereich	0 – 23	1-3	23 %	23,7/22,1/22<br>21,5/23,2/24,8	1-24(2,27-54,55 %)/9-27(20,45-61,36 %)/1-21(2,27-47,73 %)<br>6-28(13,64-63,64 %)/5-24(11,36-54,55 %)/6-23(13,64-52,27 %)
normaler Bereich	> 23 – 77	4-6	54 %	53,4/54,4/54,4<br>52,8/53,6/52,1	25-41(56,82-93,18 %)/28-42(63,64-95,45 %)/22-39(50-88,64 %)<br>29-42(65,91-95,45 %)/25-41(56,82-93,18 %)/24-39(54,55-88,64 %)
hoher Bereich	> 77 – 100	7-9	23 %	22,9/23,5/23,5<br>25,6/23,2/23	42-44(95,45-100 %)/43-44(97,73-100 %)/40-44(90,91-100 %)<br>43-44(97,73-100 %)/42-44(95,45-100 %)/40-44(90,91-100 %)
**MeHeartStigma-Score (0 – 48 Punkte)**					
niedriger Bereich	0 – 23	1-3	23 %	23,8/22,5/23,5<br>23,6/24,6/24,8	7-33(14,58-68,75 %)/11-34(22,92-70,83 %)/7-32(14,58-66,67 %)<br>11-40(22,92-83,33 %)/7-33(14,58-68,75 %)/10-29(20,83-60,42 %)
normaler Bereich	> 23 – 77	4-6	54 %	49,8/57,7/51,7<br>47,2/52,2/51,5	34-46(70,83-95,83 %)/35-47(72,92-97,92 %)/33-46(68,75-95,83 %)<br>41-47(85,42-97,92 %)/34-46(70,83-95,83 %)/30-44(62,5-91,67 %)
hoher Bereich	> 77 – 100	7-9	23 %	26,4/19,8/24,8<br>29,2/23,2/23,6	47-48(97,92-100 %)/48(100 %)/47-48(97,92-100 %)<br>48(100 %)/47-48(97,92-100 %)/45-48(93,75-100 %)
**MeHeartRecovery-Score (0 – 24 Punkte)**					
niedriger Bereich	0 – 23	1-3	23 %	22,9/21,5/23,5<br>24,1/25,1/21,8	0-13(0-54,17 %)/3-14(12,5-58,33 %)/0-12(0-50 %)<br>0-14(0-58,33 %)/2-13(8,33-54,17 %)/1-13(4,17-54,17 %)
normaler Bereich	> 23 – 77	4-6	54 %	50,2/56,7/52,6<br>51,8/46,9/56,4	14-21(58,33-87,5 %)/15-22(62,5-91,67 %)/13-21(54,17-87,5 %)<br>15-22(62,5-91,67 %)/14-21(58,33-87,5 %)/14-21(58,33-87,5 %)
hoher Bereich	> 77 – 100	7-9	23 %	26,9/21,8/23,9<br>24,1/28/21,8	22-24(91,67-100 %)/23-24(95,83-100 %)/22-24(91,67-100 %)<br>23-24(95,83-100 %)/22-24(91,67-100 %)/22-24(91,67-100 %)
**MeHeartFriends-Score (0 – 12 Punkte)**					
niedriger Bereich	0 – 23	1-3	23 %	24,3/23,8/24,8<br>23,6/22,2/21,2	0-8(0-66,67 %)/2-8(16,67-66,67 %)/0-8(0-66,67 %)<br>2-9(16,67-75 %)/0-8(0-66,67 %)/2-7(16,67-58,33 %)
normaler Bereich	> 23 – 77	4-6	54 %	35,5/31,2/39,4<br>33,8/36,2/42,4	9-11(75-91,67 %)/9-11(75-91,67 %)/9-11(75-91,67 %)<br>10-11(83,33-91,67 %)/9-11(75-91,67 %)/8-11(66,67-91,67 %)
hoher Bereich	> 77 – 100	7-9	23 %	40,2/45/35,8<br>42,6/41,5/36,4	12(100 %)/12(100 %)/12(100 %)<br>12(100 %)/12(100 %)/12(100 %)
**MeHeartTreatment-Score (0 – 12 Punkte)**					
niedriger Bereich	0 – 23	1-3	23 %	21,6/19,8/19,9<br>23,1/18,8/23	0-5(0-41,67 %)/0-6(0-50 %)/0-4(0-33,33 %)<br>1-6(8,33-50 %)/0-4(0-33,33 %)/0-5(0-41,67 %)
normaler Bereich	> 23 – 77	4-6	54 %	58,1/57/54,1<br>57,4/60,4/56,4	6-11(50-91,67 %)/7-11(58,33-91,67 %)/5-10(41,67-83,33 %)<br>7-11(58,33-91,67 %)/5-11(41,67-91,67 %)/6-11(50-91,67 %)
hoher Bereich	> 77 – 100	7-9	23 %	20,3/23,2/26<br>19,5/20,8/20,6	12(100 %)/12(100 %)/11-12(91,67-100 %)<br>12(100 %)/12(100 %)/12(100 %)
**MeHeartSchool-Score (0 – 8 Punkte)**					
niedriger Bereich	0 – 23	1-3	23 %	22,7/19,8/25,4<br>20,5/25,6/24,8	0-5(0-62,5 %)/0-5(0-62,5 %)/0-5(0-62,5 %)<br>0-5(0-62,5 %)/0-5(0-62,5 %)/0-5(0-62,5 %)
normaler Bereich	> 23 – 77	4-6	54 %	31,5/35,2/28,1<br>79,5/30,9/38,8	6-7(75-87,5 %)/6-7(75-87,5 %)/6-7(75-87,5 %)<br>6-8(75-100 %)/6-7(75-87,5 %)/6-7(75-87,5 %)
hoher Bereich	> 77 – 100	7-9	23 %	45,8/45/46,5<br>…/43,5/36,4	8(100 %)/8(100 %)/8(100 %)<br>…/8(100 %)/8(100 %)

DsQoL = Disease-spezific-Quality-of-Life; PR = Prozentrang; gesamt (N = 625) / männlich (n = 298) / weiblich (n = 327) / simple AHF (n = 195) / moderate AHF (n = 207) / komplexe AHF (n = 165)

Tabelle 8.5. *Normtabelle Jugendliche (N = 625).*

	gesamt	männlich	weiblich	simple AHF	moderate AHF	komplexe AHF
DsQoL-Gesamtscore (0-144 Punkte)						
kritischer Bereich	0 – 93	0 – 101	0 – 88	0 – 107	0 – 93	0 – 87
unkritischer Bereich	94 – 148	102– 148	89– 148	108– 148	94– 148	88– 148
MeHeartImpairments (0-44 Punkte)						
kritischer Bereich	0 – 24	0 – 27	0 – 21	0 – 28	0 – 24	0 – 23
unkritischer Bereich	25 – 44	28 – 44	22 – 44	29 – 44	25 – 44	24 – 44
MeHeartStigma (0-40 Punkte)						
kritischer Bereich	0 – 33	0 – 34	0 – 32	0 – 40	0 – 33	0 – 29
unkritischer Bereich	34 – 48	35 – 48	33 – 48	41 – 48	34 – 48	30 – 48
MeHeartRecovery (0-24 Punkte)						
kritischer Bereich	0 – 13	0 – 14	0 – 12	0 – 14	0 – 13	0 – 13
unkritischer Bereich	14 – 24	15 – 24	13 – 24	15 – 24	14 – 24	14 – 24
MeHeartFriends (0-16 Punkte)						
kritischer Bereich	0 – 8	0 – 8	0 – 8	0 – 9	0 – 8	0 – 7
unkritischer Bereich	9 – 12	9 – 12	9 – 12	10 – 12	9 – 12	8 – 12
MeHeartTreatment (0-12 Punkte)						
kritischer Bereich	0 – 5	0 – 6	0 – 4	0 – 6	0 – 4	0 – 5
unkritischer Bereich	6 – 12	7 – 12	5 – 12	7 – 12	5 – 12	6 – 12
MeHeartSchool (0-8 Punkte)						
kritischer Bereich	0 – 5	0 – 5	0 – 5	0 – 5	0 – 5	0 – 5
unkritischer Bereich	6 – 8	6 – 8	6 – 8	6 – 8	6 – 8	6 – 8

DsQoL = Disease-spezific-Quality-of-Life; gesamt (N = 625), männlich (n = 298), weiblich (n = 327), simple AHF (n = 195), moderate AHF (n = 207), komplexe AHF (n = 165)

11. KURZVERSION DES CHDSI

Vor allem in der klinischen Praxis kann es unter Umständen sinnvoll und aufgrund begrenzter Zeit notwendig sein, möglichst schnell einen ersten Eindruck über den körperlichen und psychischen Zustand eines Patienten zu bekommen. Bei vielen Screening- und Diagnosefragebögen gibt es entsprechende Kurzversionen, die einem ausführlichen Screening bzw. einer umfassenden Diagnostik vorangestellt werden können. So lassen sich durch die Verwendung von Kurzversionen bei unauffälligen Befunden Zeitaufwand, Belastung für den Patienten sowie Auswertungskosten minimieren. Zusätzlich kann ggf. auf ein langes Screening/Diagnoseverfahren verzichtet werden. Auch im Rahmen von Studien kann es – je nach Anzahl der eingesetzten Fragebögen – gute Gründe geben, auf Kurzversionen zurückzugreifen und so unter vertretbarem Zeitaufwand beispielsweise noch weitere Fragebögen einsetzen zu können.

Bei der Entwicklung des CHDSI stand die Erarbeitung einer Kurzversion nicht im Vordergrund. Gleichwohl soll der Vollständigkeit halber auf die Möglichkeit hingewiesen werden, dass auf Grundlage der durchgeführten CFA je eine aus sechs Items bestehende Kurzversion (CHDSI-SF; SF = Short Form) für Schulkinder und Jugendliche plausibel erscheint. Entsprechend wurde für jede der sechs Subskalen das Item mit der höchsten Faktorladung als Repräsentanzitem für die entsprechende Subskala ausgewählt. Der reduzierte DsQoL-Gesamtscore kann somit sowohl bei Kindern als auch bei Jugendlichen im Bereich zwischen 0 und 24 Punkten liegen.

Cronbachs Alpha der aus sechs Items bestehenden vorgeschlagenen Kurzversion des CHDSI liegt für Kinder bei .749 und ist als ausreichend hoch zu bewerten. DsQoL-Gesamtscore der Lang- und Kurzversion des Kinder-CHDSI korrelieren mit einem Wert von $r = .916$ ($p < .01$) sehr hoch, was als gute Voraussetzung für eine zuverlässige Kinder-Kurzversion gelten kann. Zwischen Patientinnen und Patienten fanden sich bei den Kindern keine Unterschiede in der Cut-Off-Grenze der Rohwertpunkte, so dass bei den Kindern geschlechtsunabhängig eine erreichte Punktzahl von 0 bis 15 Punkte als ein kritisches und eine Punktzahl von 16 bis 24 Punkte als ein unkritisches Ergebnis bewertet werden kann.

Cronbachs Alpha der aus sechs Items bestehenden vorgeschlagenen Kurzversion des CHDSI liegt für Jugendliche bei .750 und ist als ausreichend hoch zu bewerten. DsQoL-Gesamtscore der Lang- und Kurzversion des Jugend-CHDSI korrelieren mit einem Wert von $r = .914$ ($p < .01$) sehr hoch, was als gute Voraussetzung für eine zuverlässige Jugend-Kurzversion interpretiert werden kann. Zwischen Patientinnen und Patienten fanden sich bei den Jugendlichen im Gegensatz zu den Kindern Unterschiede in der Cut-Off-Grenze der Rohwertpunkte. So liegt der kritische Bereich zwischen 0-16 Punkten (männlich), 0-14 Punkten (weiblich) bzw. 0-15 Punkte (geschlechtsunabhängige Betrachtung).

Weiterführende Analysen der vorgeschlagenen Möglichkeit zur Verkürzung der CHDSI-Versionen (z. B. Berücksichtigung des AHF-Schweregrades) wurden nicht durchgeführt, da es sich sowohl bei der vorgeschlagenen verkürzten Kinder- als auch Jugendkurzversion des CHDSI um eine erhebliche Informationsreduktion für die behandelnde und/oder das Ergebnis beurteilende Person handelt, die zwar mit ausreichender Güte Rückschlüsse auf den CHDSI-Gesamtscore der Langversionen erlaubt, gleichzeitig aber eine eingehendere Betrachtung der Teilbereiche der DsQoL sowie der Berücksichtigung der Krankheitsschwere nur unzureichend zulässt. Im Folgenden werden die beiden Kurzversionen für Kinder und Jugendliche dargestellt.

KINDER KURZVERSION

In den letzten Wochen hat mich mein Herz in meiner Selbstständigkeit beeinflusst
Welche Aussage trifft auf Dich zu?

	trifft voll zu	trifft zu	weiß nicht	trifft nicht zu	trifft gar nicht zu
Ich konnte an Aktivitäten mit Freunden teilnehmen.	☐	☐	☐	☐	☐

In den letzten Wochen hat mich mein Herz in der Schule beeinflusst
Welche Aussage trifft auf Dich zu?

	trifft voll zu	trifft zu	weiß nicht	trifft nicht zu	trifft gar nicht zu
Ich konnte im Sportunterricht nicht alles mitmachen.	☐	☐	☐	☐	☐
Ich bin mit dem Unterrichtsstoff gut mitgekommen.	☐	☐	☐	☐	☐

In den letzten Wochen hat mich mein Herz körperlich eingeschränkt
Welche Aussage trifft auf Dich zu?

	trifft voll zu	trifft zu	weiß nicht	trifft nicht zu	trifft gar nicht zu
Ich fühlte mich körperlich frisch und munter.	☐	☐	☐	☐	☐

Wie sieht es mit der Erholung von Dir und Deinem Herz in den letzten Wochen aus?
Welche Aussage trifft auf Dich zu?

	trifft voll zu	trifft zu	weiß nicht	trifft nicht zu	trifft gar nicht zu
Ich hatte einen ruhigen festen Schlaf.	☐	☐	☐	☐	☐

Du und Dein Herz allgemein
Welche Aussage trifft auf Dich zu?

	trifft voll zu	trifft zu	weiß nicht	trifft nicht zu	trifft gar nicht zu
Ich finde Arztbesuche unangenehm.	☐	☐	☐	☐	☐

JUGENDLICHE KURZVERSION

In den letzten Wochen hat mich mein Herz in meiner Selbstständigkeit beeinflusst

Welche Aussage trifft auf Dich zu?

	trifft voll zu	trifft zu	weiß nicht	trifft nicht zu	trifft gar nicht zu
Ich konnte an Aktivitäten mit Freunden teilnehmen.	☐	☐	☐	☐	☐
Ich fühlte mich durch meinen Herzfehler in meiner Selbstständigkeit eingeschränkt.	☐	☐	☐	☐	☐

In den letzten Wochen hat mich mein Herz in der Schule/Ausbildung beeinflusst

Welche Aussage trifft auf Dich zu?

	trifft voll zu	trifft zu	weiß nicht	trifft nicht zu	trifft gar nicht zu
Ich bin mit dem Unterrichtsstoff gut mitgekommen.	☐	☐	☐	☐	☐

In den letzten Wochen hat mich mein Herz körperlich eingeschränkt

Welche Aussage trifft auf Dich zu?

	trifft voll zu	trifft zu	weiß nicht	trifft nicht zu	trifft gar nicht zu
Ich fühlte mich körperlich frisch und munter.	☐	☐	☐	☐	☐

Wie sieht es mit der Erholung von Dir und Deinem Herz in den letzten Wochen aus?

Welche Aussage trifft auf Dich zu?

	trifft voll zu	trifft zu	weiß nicht	trifft nicht zu	trifft gar nicht zu
Ich habe mich gut nach anstrengenden Tagen erholt.	☐	☐	☐	☐	☐

Du und Dein Herz allgemein

Welche Aussage trifft auf Dich zu?

	trifft voll zu	trifft zu	weiß nicht	trifft nicht zu	trifft gar nicht zu
Ich finde Arztbesuche unangenehm.	☐	☐	☐	☐	☐

12. ZUSAMMENFASSUNG, LIMITATIONEN UND AUSBLICK

Zentrale Ergebnisse auf einen Blick

Mit dem CHDSI liegt nun erstmals ein deutschsprachiger validierter und normierter Fragebogen zur Erfassung der DsQoL von Kindern und Jugendlichen mit AHF bestehend aus jeweils 6 Subskalen vor. Die postulierten DsQoL-Subskalen weisen durchweg gute bis sehr gute Messwerte auf (Cronbachs Alpha, MIC, Trennschärfe etc.) und das vorgeschlagene Modell der Gesamt-DsQoL konnte sowohl für die Kinder- als auch für die Jugendversion des CHDSI mittels CFA und guten bis sehr guten Fit-Indizes bestätigt werden. Sowohl das Geschlecht als auch der AHF-Schweregrad können bei der Interpretation von DsQoL-Gesamtscores und bei der Beurteilung der Subskalensummenscores berücksichtigt werden. Interpretationshilfen zur Beurteilung der mit dem CHDSI erhobenen DsQoL werden in Form von Normtabellen bereitgestellt.

Als unter Umständen zusätzliche relevante Einflussfaktoren auf die individuelle DsQoL eines Patienten scheinen vor allem das Vorhandensein von Geschwisterkindern, das elterliche Bildungs-/Berufsniveau und das subjektive Herzfehlerwissen von Bedeutung zu sein, wobei es sich bis auf eine Ausnahme um eher geringe Zusammenhänge handelt.

Der Stichprobenumfang der zwei Normierungsstichproben ist mit 530 (Kinder) bzw. 625 (Jugendliche) im sehr guten Bereich. Bei der Verwendung vor allem der Normbereiche für Patienten mit simplem, moderatem oder schwerem AHF ist der Stichprobenumfang zwar noch als ausreichend groß zu beurteilen, allerdings sollte der AHF-Schweregrad nur als Zusatzinformation und nicht als Primärnorm verstanden werden. Zusätzlich können bei Bedarf auch die Einzelitems der Subskalen sowie die Indikator-/Singleitems für ein tiefergehendes Verständnis der DsQoL eines jungen oder jugendlichen AHF-Patienten herangezogen werden. Zwar werden verschiedene Messwertergebnisse zur Interpretation der DsQoL auf Einzelitem-Ebene bereitgestellt, diese sind gleichwohl nicht als Normwerte der Einzelitems zu verwenden. Die Normwertbereiche haben ausdrücklich nur für den DsQoL-Gesamtscore sowie die Subskalensummenscores Geltung.

Für Kindergartenkinder besteht die Möglichkeit, eine reduzierte DsQoL (5 Subskalen, 31 Items) zu erheben. Die hier der Vollständigkeit halber präsentierten Normwerte sind aber aufgrund der geringen Fallzahl (46 Kindergartenkinder im Alter von 6 Jahren) nur als Orientierung, nicht aber als tatsächliche Normempfehlung zu begreifen.

Der Einsatz einer aus sechs Items vorgeschlagenen CHDSI-Kurzform kann bei guter inhaltlicher Begründung gerechtfertigt sein, um mit minimalem Aufwand einen zuverlässigen ersten Eindruck der DsQoL von Kindern und Jugendlichen mit AHF zu bekommen, die Kurzversion ist aber ausdrücklich kein gleichwertiger Ersatz für die Langversionen des CHDSI.

Limitationen

Die Validierung und Normierung des CHDSI wurde online durchgeführt. Ob sich die ermittelten Normbereiche auch in anderen Situationen wie z. B. im Rahmen eines Klinikaufenthalts, beim Ausfüllen des CHDSI im Wartezimmer oder unmittelbar vor/nach einem medizinischen Eingriff bestätigen lassen oder ob es hierfür angepasster Normbereiche bedarf, kann erst der Einsatz des CHDSI in zukünftigen Studien und im klinischen Alltag zeigen. Auch eine unterschiedliche Wahrnehmung eines Onlinefragebogens im Vergleich zu einem Papier-Bleistift-Fragebogen und somit möglicherweise ein von der Normstichprobe abweichendes Antwortverhalten kann nicht ausgeschlossen werden, da alle Fragebögen online und vermutlich in der Regel von zu Hause aus bearbeitet wurden. Gleichwohl scheint das mit dem CHDSI messbare Modell der DsQoL robust zu

sein, so dass die vorgeschlagenen Normbereiche grundsätzlich als zuverlässig interpretiert werden können, denn es ist davon auszugehen, dass zwar das Befragungsmedium (Onlinefragebogen) gleich, die Lebensumstände der aus ganz Deutschland stammenden Studienteilnehmer zum Befragungszeitpunkt jedoch in ausreichendem Maß verschieden waren.

Bei der Entwicklung des CHDSI wurden kulturelle und religiöse Themen nicht berücksichtigt. Es können daher keine Aussagen darüber gemacht werden, ob der CHDSI in allen Kulturen oder Religionsgemeinschaften mit den Normbereichen vergleichbare Ergebnisse liefert. Da die Stichproben jedoch in ganz Deutschland rekrutiert wurden, ist von kulturell und religiös heterogenen Teilnehmerinnen und Teilnehmern auszugehen, die für die Population der Kinder und Jugendlichen mit AHF in Deutschland als repräsentativ gelten können.

Der CHDSI wurde in der deutschen Sprache entwickelt und die Validierung/Normierung erfolgte in Deutschland. Eine Übertragbarkeit der gefundenen Ergebnisse auf andere deutschsprachige Länder ist – genau wie auf nicht deutschsprachige Länder – nicht ohne weiteres sinnvoll/zulässig. Vielmehr wird empfohlen, vor dem Einsatz des CHDSI eine eigene Validierung/Normierung für das jeweilige Land und bei Bedarf die Übersetzung des CHDSI in die entsprechende Landesprache vorzunehmen.

Ausblick

Der CHDSI soll zukünftig dabei helfen, die DsQoL von jungen und jugendlichen AHF-Patienten in der klinischen Praxis stärker zu berücksichtigen. Er kann unter anderem bei der Evaluation, Erstellung und Weiterentwicklung von Behandlungs-, Interventions- und Unterstützungsprogrammen Verwendung finden. Die Einsatzgebiete des CHDSI sind vielfältig und ein bundesweiter Einsatz des CHDSI in Forschung und klinischer Praxis über einen längeren Zeitraum hinweg – im Idealfall mit der festen Implementierung in den Ablauf regelmäßiger Routineuntersuchungen – wird wichtige Daten für ein tieferes Verständnis der DsQoL liefern. Die Übersetzung des CHDSI in andere Sprachen und die Normierung des CHDSI in anderen Ländern für nicht-kommerzielle Zwecke ist grundsätzlich willkommen, sofern zuvor Rücksprache mit den Autoren gehalten wird.

13. LITERATURVERZEICHNIS

1. Geigle R, Jones SB. Outcomes measurement: a report from the front. *Inquiry.* 1990;27(1):7-13.
2. Codman EA. The product of a hospital. 1914. *Archives of Ppathology & Laboratory Medicine.* 1990;114(11):1106-1111.
3. Silver GA. Paul Anthony Lembcke, MD, MPH: a pioneer in medical care evaluation. *American Journal of Public Health.* 1990;80(3):342-348.
4. McDermott W. Absence of indicators of the influence of its physicians on a society's health: Impact of physician care on society. *The American Journal of Medicine.* 1981;70(4):833-843.
5. Almy T. Comprehensive functional assessment for elderly patients: position paper by Health and Public Policy Committee, American College of Physicians. *Annals of Internal Medicine.* 1988;109(1):70-72.
6. Cluff LE. Chronic disease, function and the quality of care. *Journal of Chronic Diseases.* 1981;34(7):299-304.
7. Schroeder SA. Outcome assessment 70 years later: are we ready? *The New England Journal of Medicine.* 1987;316(3):160-162.
8. Ellwood PM. Shattuck lecture--outcomes management. A technology of patient experience. *The New England Journal of Medicine.* 1988;318(23):1549-1556.
9. Tarlov AR. Shattuck lecture--the increasing supply of physicians, the changing structure of the health-services system, and the future practice of medicine. *The New England Journal of Medicine.* 1983;308(20):1235-1244.
10. Hays RD, Stewart AL. The structure of self-reported health in chronic disease patients. *Psychological Assessment: A Journal of Consulting and Clinical Psychology.* 1990;2(1):22-30.
11. Liang J. Self-Reported Physical Health Among Aged Adults. *Journal of Gerontology.* 1986;41(2):248-260.
12. Ware Jr JE, Brook RH, Davies AR, Lohr KN. Choosing measures of health status for individuals in general populations. *American Journal of Public Health.* 1981;71(6):620-625.
13. Bombardier C, Ware J, Russell I, Larson M, Chalmers A, Read J. Auranofin therapy and quality of life in patients with rheumatoid arthritis. Results of a multicenter trial. *The American Journal of Medicine.* 1986;81(4):565-578.
14. Croog S, Levine S, Testa M. The effects of antihypertensive therapy on the quality of life. *The New England Journal of Medicine.* 1986;314(26):1657-1664.
15. Fowler FJ, Wennberg JE, Timothy RP, Barry MJ, Mulley AG, Hanley D. Symptom status and quality of life following prostatectomy. *JAMA.* 1988;259(20):3018-3022.
16. Radoschewski M. Gesundheitsbezogene Lebensqualität – Konzepte und Maße. *Bundesgesundheitsblatt - Gesundheitsforschung - Gesundheitsschutz.* 2000;43(3):165-189.
17. Johnston DF, Carley MJ. Social Measurement and Social Indicators: Issues of Policy and Theory. *The Annals of the American Academy of Political and Social Science.* 1981;453:237-253.
18. Noack H. Conceptualizing and measuring health. *WHO Regional Publications European Series.* 1991;37:85-112.
19. Mehrez A, Gafni A. uality-adjusted life years, utility theory, and healthy-years equivalents. *Medical Decision Making.* 1989;9(2):142-149.
20. Pedroni G, Zweifel P. *Wie mißt man Gesundheit?* Basel: Pharma Information; 1990.
21. Katz S. The science of quality of life. *Journal of Chronic Diseases.* 1987;40(6):459-463.
22. WHO. Constitution of the World Health Organization. 2006. https://www.who.int/publications/m/item/constitution-of-the-world-health-organization.
23. Spitzer WO, Dobson AJ, Hall J, et al. Measuring the quality of life of cancer patients: a concise QL-index for use by physicians. *Journal of Chronic Diseases.* 1981;34(12):585-597.
24. Ravens-Sieberer U, Bullinger M. Assessing health-related quality of life in chronically ill children with the German KINDL: first psychometric and content analytical results. *Quality of Life Research.* 1998;7(5):399-407.

25. Ravens-Sieberer U, Bullinger M. News from the KINDL-Questionnaire – A new version for adolescents. *Quality of Life Research.* 1998;7:653.
26. Post MWM. Definitions of quality of life: what has happened and how to move on. *Topics in Spinal Cord Injury Rehabilitation.* 2014;20(3):167-180.
27. von der Lippe E, Rattay P. Association of partner, parental, and employment statuses with self-rated health among German women and men. *SSM - Population Health.* 2016;28(2):390-398.
28. Cohen SR, Mount BM, MacDonald N. Defining quality of life. *European Journal of Cancer (Oxford, England : 1990).* 1996;32A(5):753-754.
29. Bullinger M. Erfassung der gesundheitsbezogenen Lebensqualität mit dem SF-36 Health Survey [Assessment of health related quality of life with the SF-36 Health Survey]. *Die Rehabilitation.* 1996;35(3):XVII-XXVII; quiz XXVII-XXIX.
30. Schumacher G, Hess J, Bühlmeyer K. *Klinische Kinderkardiologie: Diagnostik und Therapie der angeborenen Herzfehler.* Springer; 2007.
31. Kaltman JR, Burns KM, Pearson GD. Perspective on Congenital Heart Disease Research. *Circulation Research.* 2017;120(6):898-900.
32. Hinton RB, Ware SM. Heart Failure in Pediatric Patients With Congenital Heart Disease. *Circulation Research.* 2017;120(6):978-994.
33. Schwedler G, Lindinger A, Lange PE, et al. Frequency and spectrum of congenital heart defects among live births in Germany : a study of the Competence Network for Congenital Heart Defects. *Clinical Research in Cardiology.* 2011;100(12):1111-1117.
34. Diller G-P, Breithardt G, Baumgartner H. Congenital heart defects in adulthood. *Deutsches Ärzteblatt International.* 2011;108(26):452-459.
35. Moons P, Bovijn L, Budts W, Belmans A, Gewillig M. Temporal trends in survival to adulthood among patients born with congenital heart disease from 1970 to 1992 in Belgium. *Circulation.* 2010;122(22):2264-2272.
36. Marelli AJ, Mackie AS, Ionescu-Ittu R, Rahme E, Pilote L. Congenital heart disease in the general population: changing prevalence and age distribution. *Circulation.* 2007;115(2):163-172.
37. Kovacs AH, Verstappen A. The whole adult congenital heart disease patient. *Progress in Cardiovascular Diseases.* 2011;53(4):247-253.
38. Sable C, Foster E, Uzark K, et al. Best practices in managing transition to adulthood for adolescents with congenital heart disease: the transition process and medical and psychosocial issues: a scientific statement from the American Heart Association. *Circulation.* 2011;123(13):1454-1485.
39. Marian AJ. Congenital Heart Disease: The Remarkable Journey From the "Post-Mortem Room" to Adult Clinics. *Circulation Research.* 2017;120(6):895-897.
40. Dulfer K, Helbing WA, Duppen N, Utens EM. Associations between exercise capacity, physical activity, and psychosocial functioning in children with congenital heart disease: a systematic review. *European Journal of Preventive Cardiology.* 2014;21(10):1200-1215.
41. Holst KA, Said SM, Nelson TJ, Cannon BC, Dearani JA. Current Interventional and Surgical Management of Congenital Heart Disease: Specific Focus on Valvular Disease and Cardiac Arrhythmias. *Circulation Research.* 2017;120(6):1027-1044.
42. Bouma BJ, Mulder BJ. Changing Landscape of Congenital Heart Disease. *Circulation Research.* 2017;120(6):908-922.
43. Voss C, Harris KC. Physical activity evaluation in children with congenital heart disease. *Heart.* 2017;103(18):1408-1412.
44. Amedro P, Dorka R, Moniotte S, et al. Quality of Life of Children with Congenital Heart Diseases: A Multicenter Controlled Cross-Sectional Study. *Pediatric Cardiology.* 2015;36(8):1588-1601.
45. Mellion K, Uzark K, Cassedy A, et al. Health-related quality of life outcomes in children and adolescents with congenital heart disease. *The Journal of Pediatrics.* 2014;164(4):781-788.

46. Drakouli M, Petsios K, Giannakopoulou M, Patiraki E, Voutoufianaki I, Matziou V. Determinants of quality of life in children and adolescents with CHD: a systematic review. *Cardiology in the Young.* 2015;25(6):1027-1036.
47. Fredriksen PM, Diseth TH, Thaulow E. Children and adolescents with congenital heart disease: assessment of behavioural and emotional problems. *European Child & Adolescent Psychiatry.* 2009;18(5):292-300.
48. Meyer M, Oberhoffer R, Hock J, Giegerich T, Müller J. Health-related quality of life in children and adolescents: Current normative data, determinants and reliability on proxy-report. *Journal of Paediatrics and Child Health.* 2016;52(6):628-631.
49. Müller J, Hess J, Hager A. General anxiety of adolescents and adults with congenital heart disease is comparable with that in healthy controls. *International Journal of Cardiology.* 2013;165(1):142-145.
50. Moons P, Van Deyk K, Marquet K, et al. Individual quality of life in adults with congenital heart disease: a paradigm shift. *European Heart Journal.* 2005;26(3):298-307.
51. Vigl M, Niggemeyer E, Hager A, Schwedler G, Kropf S, Bauer U. The importance of socio-demographic factors for the quality of life of adults with congenital heart disease. *Quality of Life Research.* 2011;20(2):169-177.
52. Lesch W, Specht K, Lux A, Frey M, Utens E, Bauer U. Disease-specific knowledge and information preferences of young patients with congenital heart disease. *Cardiology in the Young.* 2014;24(2):321-330.
53. Hemingway H, Nicholson A, Stafford M, Roberts R, Marmot M. The impact of socioeconomic status on health functioning as assessed by the SF-36 questionnaire: the Whitehall II Study. *American Journal of Public Health.* 1997;87(9):1484-1490.
54. Helm PC, Sticker EJ, Keuchen R, et al. Is having a job a protective factor? Employment status and state of medical care as subjectively perceived by adults with CHD in Germany. *Cardiology in the Young.* 2017;27(6):1110-1117.
55. Hager A, Hess J. Lebensqualität nach Operation angeborener Herzfehler. *Monatsschrift Kinderheilkunde.* 2006;154(7):639-643.
56. Helm PC, Koerten M-A, Abdul-Khaliq H, Baumgartner H, Kececioglu D, Bauer UM. Representativeness of the German National Register for Congenital Heart Defects: a clinically oriented analysis. *Cardiology in the Young.* 2016;26(5):921-926.
57. Reng C-M, Debold P, Specker C, Pommerening K. *Generische Lösungen der TMF zum Datenschutz für die Forschungsnetze in der Medizin.* Medizinisch Wissenschaftliche Verlagsgesellschaft; 2006.
58. Mayer HO. *Interview und schriftliche Befragung. Entwicklung, Durchführung und Auswertung.* München: Oldenburg Wissenschaftsverlag; 2006.
59. Cohen J. *Statistical Power Analysis for the Behavioral Sciences.* New York: Erlbaum; 1988.
60. Peter JP. Construct Validity: A Review of Basic Issues and Marketing Practices. *Journal of Marketing Research.* 1981;18(2):133-145.
61. Diamantopoulos A. The C-OAR-SE procedure for scale development in marketing: a comment. *International Journal of Research in Marketing.* 2005;22(1):1-9.
62. AERA, APA, NCME. *Standards for Educational and Psychological Testing.* American Educational Research Association; 2005.
63. Peterson RA. A Meta-analysis of Cronbach's Coefficient Alpha. *Journal of Consumer Research.* 1994;21(2):381-391.
64. Cronbach LJ. Coefficient alpha and the internal structure of tests. *Psychometrika.* 1951;16(3):297-334.
65. Frey BB. *The SAGE Encyclopedia of Educational Research, Measurement, and Evaluation.* Thousand Oaks, California: SAGE Publications, Inc.; 2018.
66. Köhler T. *Statistik für Psychologen, Pädagogen und Mediziner: Ein Lehrbuch.* Kohlhammer; 2004.
67. Amelang M, Schmidt-Atzert L. *Psychologische Diagnostik und Intervention.* 4 ed. Berlin/Heidelberg: Springer; 2006.

68. Bühner M. *Einführung in die Test- und Fragebogenkonstruktion.* München: Pearson; 2011.
69. Schermelleh-Engel K, Werner C. Methoden der Reliabilitätsbestimmung. In: Moosbrugger H, Kelava A, eds. *Testtheorie und Fragebogenkonstruktion*. 2., aktualisierte und überarbeitete Auflage ed. Heidelberg: Springer; 2011.
70. Schermelleh-Engel K, Schweizer K. Multitrait-Multimethod-Analysen. In: Moosbrugger H, Kelava A, eds. *Testtheorie und Fragebogenkonstruktion*. Heidelberg: Springer; 2007.
71. Lang FR, Wendt V. Entwicklungspsychologische Grundlagen der Diagnostik. In: Petermann F, Eid M, eds. *Handbuch der Psychologischen Diagnostik*. Göttingen: Hogrefe Verlag; 2006:57-67.
72. Bortz J, Döring N. *Forschungsmethoden und Evaluation für Human- und Sozialwissenschaftler.* Heidelberg: Springer Medizin Verlag; 2006.
73. Cortina JM. What is Coefficient Alpha? An Examination of Theory and Applications. *Journal of Applied Psychology.* 1993;78(1):98-104.
74. Schmitt N. Uses and abuses of coefficient alpha. *Psychological Assessment.* 1996;8(4):350-353.
75. van Griethuijsen RALF, van Eijck MW, Haste H, et al. Global patterns in students' views of science and interest in science. *Research in Science Education.* 2014;45(4):581-603.
76. Fisseni HJ. *Lehrbuch der psychologischen Diagnostik.* Göttingen: Hogrefe; 1997.
77. Briggs SR, Cheek JM. The role of factor analysis in the development and evaluation of personality scales. *Journal of Personality.* 1986;54(1):106–148.
78. Clark LA, Watson D. Constructing validity. Basic issues in objective scale development. *Psychological Assessment.* 1995;7(3):309–319.
79. Lothar SA, Manfred A, Thomas F. *Psychologische Diagnostik.* 2012.
80. Smith GT. On Construct Validity: Issues of Method and Measurement. *Psychological Assessment.* 2005;17(4):396-408.
81. Messick S. Foundations of validity: Meaning and consequences in psychological assessment. *European Journal of Psychological Assessment.* 1994;10(1):1-9.
82. Cook TD, Campbell DT, Shadish W. *Experimental and Quasi-Experimental Designs for Generalized Causal Inference.* Boston: Houghton Mifflin; 2003.
83. Zumbo BD. Validity: Foundational Issues and Statistical Methodology. In: Rao CR, Sinharay S, eds. *Handbook of Statistics.* Vol 26. Amsterdam: Elsevier Science; 2007.
84. Mulaik SA. *The Foundations of Factor Analysis.* New York: McGraw-Hill; 1972.
85. Finney S, DiStefano C, Kopp J. Overview of estimation methods and preconditions for their application with structural equation modeling. In: Schweizer K, DiStefano C, eds. *Principles and methods of test construction: Standards and recent advances*. Boston: Hogrefe; 2016:135-165.
86. DiStefano C. The impact of categorization with confirmatory factor analysis. *Structural Equation Modeling.* 2002;9(3):327-346.
87. Sharma S, Mukherjee S, Kumar A, Dillon WR. A simulation study to investigate the use of cutoff values for assessing model fit in covariance structure models. *Journal of Business Research.* 2005;58(7):935-943.
88. Coenders G, Saris WE. Categorization and measurement quality. The choice between Pearson and Polychoric correlations. In: Saris WE, Münnich A, eds. *The Multitrait-Multimethod approach to evaluate measurement instruments*. Budapest: Eötvös University Press; 1995:125-144.
89. Flora DB, Curran PJ. An empirical evaluation of alternative methods of estimation for confirmatory factor analysis with ordinal data. *Psychological Methods.* 2004;9(4):466-491.
90. Freiberg A, Stover JA, De la Iglesia G, Fernández M. Correlaciones policóricas y tetracóricas en estudios factoriales exploratorios y confirmatorios. *Ciencias Psicológicas.* 2013;7(2):151-164.
91. Brown TA. *Confirmatory factor analysis for applied research.* New York: Guildford Press; 2006.
92. O'Brien RM. The relationship between ordinal measures and their underlying values: Why all the disagreement? *Quality and Quantity.* 1985;19(3):265-277.

93. Saris WE, van Wijk T, Scherpenzeel A. Validity and reliability of subjective social indicators: “The effect of different measures of association”. *Social Indicators Research.* 1998;45(1/3):173-199.
94. Holgado-Tello FP, Chacón-Moscoso S, Barbero-García I, Vila-Abad E. Polychoric versus Pearson correlations in exploratory and confirmatory factor analysis of ordinal variables. *Quality and Quantity.* 2010;44(1):153-166.
95. Boomsma A, Hoogland JJ. The robustness of lisrel modeling revisited. In: Cudeck R, du Toit S, Sörbom D, eds. *Structural equation models: Present and future. A festschrift in honor of Karl Jöreskog*. Chicago: Scientific Software International; 2001:139-168.
96. Muthén LK, Muthén BO. How to use a Monte Carlo study to decide on sample size and determine power. *Structural Equation Modeling.* 2002;9(4):599-620.
97. Kaplan D. Evaluating and modifying covariance structure models: A review and recommendation. *Multivariate Behavioral Research.* 1990;25(2):137-155.
98. Yang-Wallentin F, Jöreskog KG, Luo H. Confirmatory factor analysis of ordinal variables with misspecified models. *Structural Equation Modeling.* 2010;17(3):392-423.
99. Bollen KA. *Structural equations with latent variables.* New York: Wiley; 1989.
100. MacCallum RC, analysis F. Factor analysis. In: Millsap RE, Maydeu-Olivares A, eds. *The SAGE handbook of quantitative methods in psychology*. Thousand Oaks: Sage Publications Ltd; 2009:123-147.
101. Bandalos DL. Relative Performance of Categorical Diagonally Weighted Least Squares and Robust Maximum Likelihood Estimation. *Structural Equation Modeling: A Multidisciplinary Journal.* 2014;21(1):102-116.
102. Muthén BO, du Toit SHC, Spisic D. Robust Infoerince using Weighted Least Squares and Quadratic Estimating Equations in Latent Variable Modeling with Categorical and Continuous Outcomes. 1997. https://www.statmodel.com/download/Article_075.pdf. Accessed 14.05.2022.
103. Rhemtulla M, Brosseau-Liard PÉ, Savalei V. When can categorical variables be treated as continuous? A comparison of robust continuous and categorical SEM estimation methods under suboptimal conditions. *Psychological Methods.* 2012;17(3):354-373.
104. Hu L-t, Bentler PM. Cutoff Criteria for Fit Indexes in Covariance Structure Analysis. Conventional Criteria Versus New Alternatives. *Structural Equation Modeling: A Multidisciplinary Journal.* 1999;6(1):1–55.
105. Yu C-Y. *Evaluating cutoff criteria of model fit indices for latent variable models with binary and continuous outcomes.* University of California, Los Angeles; 2002.
106. Bentler PM, Chou CP. Practical issues in structural modeling. *Sociological Methods & Research.* 1987;16:78-117.
107. Comrey A, Lee H. *A first course in factor analysis.* Hillsdale, NJ: Erlbaum; 1992.
108. Hu L-t, Bentler PM. Fit indices in covariance structure modeling: sensitivity to underparameterized model misspecification. *Psychological Methods.* 1998;3(4):424-453.
109. Barrett P. Structural equation modeling: Adjudging model fit. *Personality and Individual Differences.* 2007;42(5):815-824.
110. Kline RB. *Principles and practice of structural equation modeling.* New York: Guilford Press; 2011.
111. Mulaik SA, James LR, van Alstine J, Bennet N, Lind S, Stilwell CD. Evaluation of Goodness-of-Fit Indices for Structural Equation Models. *Psychological Bulletin.* 1989;105(3):430-445.
112. McIntosh C. Rethinking fit assessment in structural equation modelling: A commentary and elaboration on Barrett (2007). *Personality and Individual Differences.* 2006;42(5):859-867.
113. Bentler PM, Bonett DG. Significance tests and goodness of fit in the analysis of covariance structures. *Psychological Bulletin.* 1980; 88(3):588–606.
114. Jöreskog KG, Sörbom D. *PRELIS2: User’s reference guide.* Chicago: Scientific Software International; 1996.
115. Kenny DA, McCoach DB. Effect of the Number of Variables on Measures of Fit in Structural Equation Modeling. *Structural Equation Modeling.* 2003;10(3):333-351.

116. Wheaton B, Muthen B, Alwin DF, Summers G. Assessing Reliability and Stability in Panel Models. *Sociological Methodology.* 1977;8(1):84-136.
117. Tabachnick BG, Fidell LS. *Using Multivariate Statistics.* Boston: Allyn and Bacon; 2013.
118. Steiger JH. Structural Model Evaluation and Modification: An Interval Estimation Approach. *Multivariate Behavioral Research.* 1990;25(2):173–180.
119. Byrne BM. *Structural Equation Modeling with LISREL, PRELIS, and SIMPLIS: Basic Concepts, Applications, and Programming.* New York: Psychology Press; 1998.
120. Diamantopoulos A, Siguaw JA. *Introducing LISREL.* London: SAGE Publications, Inc; 2000.
121. MacCallum RC, Browne MW, Sugawara HM. Power analysis and determination of sample size for covariance structure modeling of fit involving a particular measure of model. *Psychological Methods.* 1996;13(2):130–149.
122. Steiger JH. Understanding the limitations of global fit assessment in structural equation modeling. *Personality and Individual Differences.* 2007;42(5):893-898.
123. Fan X, Thompson B, Wang L. Effects of sample size, estimation methods, and model specification on structural equation modeling fit indexes. *Structural Equation Modeling: A Multidisciplinary Journal.* 1999;6(1):56-83.
124. Raykov T. On the large-sample bias, variance, and mean squared error of the conventional noncentrality parameter estimator of covariance structure models. *Structural Equation Modeling.* 2000;7(3):431-441.
125. Raykov T. Bias-corrected estimation of noncentrality parameters of covariance structure models. *Structural Equation Modeling.* 2005;12(1):120-129.
126. Portela DMP. *Contributo das técnicas de análise fatorial para o estudo do programa" ocupação científica de jovens nas férias"*. Lisboa, Universidade Aberta; 2012.
127. Nevitt J, Hancock GR. Performance of Bootstrapping Approaches to Model Test Statistics and Parameter Standard Error Estimation in Structural Equation Modeling. *Structural Equation Modeling: A Multidisciplinary Journal.* 2001;8(3):353-377.
128. Beauducel A, Herzberg PY. On the Performance of Maximum Likelihood Versus Means and Variance Adjusted Weighted Least Squares Estimation in CFA. *Structural Equation Modeling.* 2006;13(2):186–203.
129. Hutchinson SR, Olmos A. Behavior of descriptive fit indexes in confirmatory factor analysis using ordered categorical data. *Structural Equation Modeling: A Multidisciplinary Journal.* 1998;5(4):344-364.
130. Finney SJ, DiStefano C. Non-normal and categorical data in structural equation modeling. In: Hancock GR, Mueller RO, eds. *Structural equation modeling: A second course*. 2nd Edition ed. Charlotte, NC: Information Age Publishing; 2013:439-492.
131. Hair JF, Black Jr. WC, Babin BJ, Anderson RE. *Multivariate data analysis.* 7th ed. ed. Upper Saddle River, NJ: Pearson Prentice Hall; 2009.
132. De Winter JC. Using the Student's t-test with extremely small sample sizes. *Practical Assessment, Research, and Evaluation.* 2013;18(1):10.
133. Glass GV, Peckham PD, Sanders JR. Consequences of failure to meet assumptions underlying the fixed effects analyses of variance and covariance. *Review of educational research.* 1972;42(3):237-288.
134. Harwell MR, Rubinstein EN, Hayes WS, Olds CC. Summarizing Monte Carlo results in methodological research: The one-and two-factor fixed effects ANOVA cases. *Journal of educational statistics.* 1992;17(4):315-339.
135. Lix LM, Keselman JC, Keselman HJ. Consequences of assumption violations revisited: A quantitative review of alternatives to the one-way analysis of variance F test. *Review of educational research.* 1996;66(4):579-619.
136. Bortz J, Schuster C. Statistik für Human- und Sozialwissenschaftler. 2010.
137. Herzog MH, G F, Clarke A. *Understanding Statistics and Experimental Design: How to Not Lie with Statistics.* Cham, Switzerland: Springer Nature; 2019.
138. Stone ER. t Test, Independent Samples. In: Salkind NJ, ed. *Encyclopedia of research design*. Los Angeles: SAGE; 2010:1551–1556.

139. Norušis MJ. *SPSS 14.0. Statistical Procedures Companion.* Upper Saddle River, NJ: Prentice Hall; 2005.
140. Bühner M, Ziegler M. *Statistik für Psychologen und Sozialwissenschaftler.* München: Pearson; 2009.
141. Tent L, Stelzl I, Hogrefe.) P-pDTumGG. *Pädagogisch-psychologische Diagnostik: Theoretische und methodische Grundlagen.* Göttingen: Hogrefe; 1993.
142. Wilcox RR. *Statistics for the social sciences.* San Diego: Academic Press; 1995.
143. Amelang M, Schmidt-Atzert L. *Psychologische Diagnostik und Intervention.* 5 ed. Berlin/Heidelberg: Springer; 2012.
144. Schelten A. *Testbeurteilung und Testerstellung – Grundlagen der Teststatistik und Testtheorie für Pädagogen und Ausbilder in der Praxis.* 2 ed. Stuttgart: Franz Steiner Verlag; 1997.

14. APPENDIX A: CHDSI Kinder (Langversion)

CHDSI

Congenital **H**eart **D**isease **S**pecific **I**nventory
Krankheitsspezifische Lebensqualität von Kindern und Jugendlichen mit angeborenem Herzfehler
von 6 bis 13 Jahre

Vorname: ____________________ Nachname: ________________________

Alter: ________ Geschlecht: ________ Geschwister: Mädchen ____ Alter: ________
Junge ______ Alter: ________

Kindergartenjahre: Schulart: ________________ Klasse: Ausbildung: ____________

Schulabschluss Eltern: Mama __________________ Papa ____________________

Berufsausbildung Eltern: Mama __________________ Papa ____________________

Berufstätig: Mama ◯ Papa ◯ beide ◯ keiner ◯

Berufstätigkeit Eltern: Vollzeit Teilzeit Hausfrau/-mann Andere: ______________
Mama ◯ Mama ◯ Mama ◯
Papa ◯ Papa ◯ Papa ◯

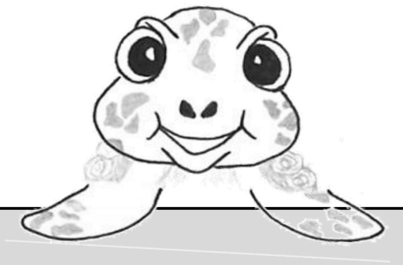

Hier oben steht immer ein Bereich um den es in den Fragen geht

Dann folgt die Frage:

Welche Aussage trifft auf Dich zu?

Dann machst Du Dein Kreuz wo Du es am Besten passend findest

	trifft voll zu	trifft zu	weiß nicht	trifft nicht zu	trifft gar nicht zu
Ich esse gerne Eis	X	◯	◯	◯	◯
Ich lese gerne	◯	◯	X	◯	◯

Vorsicht manche Fragen sind umgedreht, das erkennst Du an den Smileys:

Welche Aussage trifft auf Dich zu?

	trifft voll zu	trifft zu	weiß nicht	trifft nicht zu	trifft gar nicht zu
Ich gehe nicht gerne spazieren	◯	X	◯	◯	◯
Ich bin oft traurig	◯	◯	◯	X	◯

Copyright © 2025 by J. Remmele & P. C. Helm

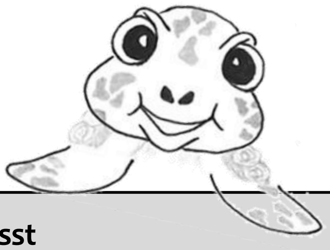

In den letzten Wochen hat mich mein Herz in meiner Selbstständigkeit beeinflusst

**Welche Aussage trifft auf Dich zu?**	trifft gar nicht zu	trifft nicht zu	weiß nicht	trifft zu	trifft voll zu
1. Ich fühlte mich im Vergleich mit Freunden gleich gut.	○	○	○	○	○
2. Ich habe mich wohl gefühlt wie ich bin.	○	○	○	○	○
3. Ich konnte an Aktivitäten mit Freunden teilnehmen.	○	○	○	○	○

	trifft voll zu	trifft zu	weiß nicht	trifft nicht zu	trifft gar nicht zu
4. Ich habe mir Sorgen über meine Zukunft gemacht.	○	○	○	○	○
5. Ich habe mich hilflos und traurig gefühlt.	○	○	○	○	○
6. Ich hatte Schwierigkeiten Freunde zu finden.	○	○	○	○	○

Wegen meinem Herz haben sich in den letzten Wochen …

Welche Aussage trifft auf Dich zu?
Wenn Du noch nicht in die Schule gehst, beziehen sich die Aussagen zur Schule natürlich auf die Erzieher und die anderen Kindergartenkinder.

	trifft voll zu	trifft zu	weiß nicht	trifft nicht zu	trifft gar nicht zu
7. … meine Eltern viele Sorgen gemacht, das tut mir leid.	○	○	○	○	○
8. … in der Schule Lehrer und Mitschüler mir gegenüber komisch verhalten.	○	○	○	○	○
9. …meine Eltern mir gegenüber anders verhalten als meinen Geschwistern gegenüber. ○ habe keine	○	○	○	○	○
10. … meine Eltern und andere Erwachsene übervorsichtig mir gegenüber verhalten.	○	○	○	○	○
11. … Lehrer und Mitschüler besonders um mich bemüht, ohne dass ich das wollte.	○	○	○	○	○
12. … andere Kinder über mich lustig gemacht.	○	○	○	○	○

Copyright © 2025 by J. Remmele & P. C. Helm

In den letzten Wochen hat mich mein Herz in der Schule beeinflusst

Ich gehe noch nicht in die Schule , ○ dann brauchst Du diesen Block nicht ausfüllen!

**Welche Aussage trifft auf Dich zu?**	trifft voll zu	trifft zu	weiß nicht	trifft nicht zu	trifft gar nicht zu
13. Ich habe viel Unterricht verpasst wegen Untersuchungen.	○	○	○	○	○
14. Ich konnte im Sportunterricht nicht alles mitmachen.	○	○	○	○	○
15. Ich habe mir Gedanken gemacht, ob ich die Schule schaffe.	○	○	○	○	○

	trifft gar nicht zu	trifft nicht zu	weiß nicht	trifft zu	trifft voll zu
16. Ich bin mit dem Unterrichtsstoff gut mitgekommen.	○	○	○	○	○
17. Ich bin mit den Hausaufgaben gut zurechtgekommen.	○	○	○	○	○
18. Ich gehe grundsätzlich gerne zur Schule.	○	○	○	○	○

In den letzten Wochen hat mich mein Herz körperlich eingeschränkt

**Welche Aussage trifft auf Dich zu?**	trifft gar nicht zu	trifft nicht zu	weiß nicht	trifft zu	trifft voll zu
19. Ich konnte alle körperlichen Aktivitäten machen wie ich wollte.	○	○	○	○	○
20. Ich konnte alles machen ohne außer Puste zu geraten.	○	○	○	○	○
21. Ich hatte keine körperlichen Schmerzen.	○	○	○	○	○
22. Ich habe mich selten krank gefühlt.	○	○	○	○	○
23. Ich konnte problemlos meine Hobbies machen.	○	○	○	○	○
24. Ich fühlte mich körperlich frisch und munter.	○	○	○	○	○

Copyright © 2025 by J. Remmele & P. C. Helm

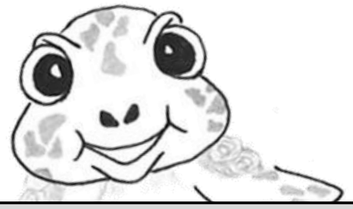

In den letzten Wochen hatte ich wegen meinem Herz …

Welche Aussage trifft auf Dich zu?	trifft voll zu	trifft zu	weiß nicht	trifft nicht zu	trifft gar nicht zu
25. … schnell keine Puste mehr.	○	○	○	○	○
26. … abends öfter dicke Beine und Füße .	○	○	○	○	○
27. … häufig mit Schwindel zu tun.	○	○	○	○	○
28. … auch Schmerzen in der Brust.	○	○	○	○	○
29. … gemerkt, dass ich schnell müde werde.	○	○	○	○	○
30. … öfter komisches Herzklopfen.	○	○	○	○	○

Wie sieht es mit der Erholung von Dir und Deinem Herz in den letzten Wochen aus?

Welche Aussage trifft auf Dich zu?	trifft voll zu	trifft zu	weiß nicht	trifft nicht zu	trifft gar nicht zu
31. Ich konnte nicht gut einschlafen.	○	○	○	○	○
32. Ich bin nachts häufig aufgewacht.	○	○	○	○	○
33. Ich bin morgens schwer aus dem Bett gekommen.	○	○	○	○	○

	trifft gar nicht zu	trifft nicht zu	weiß nicht	trifft zu	trifft voll zu
34. Ich bin erholt aufgewacht.	○	○	○	○	○
35. Ich hatte einen ruhigen festen Schlaf.	○	○	○	○	○
36. Ich habe mich gut nach anstrengenden Tagen erholt.	○	○	○	○	○

Copyright © 2025 by J. Remmele & P. C. Helm

Du und Dein Herz allgemein

**Welche Aussage trifft auf Dich zu?**		trifft gar nicht zu	trifft nicht zu	weiß nicht	trifft zu	trifft voll zu
37. Ich weiß gut Bescheid über mein Herz und was ihm fehlt.		○	○	○	○	○
38. Vor dem Krankenhaus habe ich keine Angst.		○	○	○	○	○
39. Ich vertrage meine Medikamente gut.	○ nehme keine	○	○	○	○	○

		trifft voll zu	trifft zu	weiß nicht	trifft nicht zu	trifft gar nicht zu
40. Ich finde Arztbesuche unangenehm.		○	○	○	○	○
41. Ich mag es nicht am Herzen untersucht zu werden.		○	○	○	○	○
42. Ich finde es nervig Medikamente zu nehmen.	○ nehme keine	○	○	○	○	○
43. Ich finde meine Operationsnarbe sehr unangenehm.	○ habe keine	○	○	○	○	○

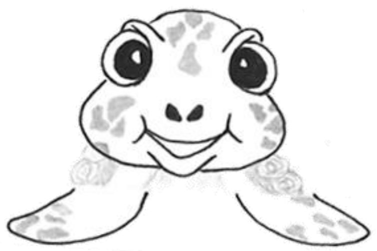

Gibt es etwas, das Du wichtig findest und hier noch nicht dabei war?

____
____
____
____
____
____
____

Vielen Dank, dass du die Fragen beantwortet hast!

Copyright © 2025 by J. Remmele & P. C. Helm

15. APPENDIX B: CHDSI Kinder (Kurzversion)

CHDSI Kurzversion

Congenital **H**eart **D**isease **S**pecific **I**nventory
Krankheitsspezifische Lebensqualität von Kindern und Jugendlichen mit angeborenem Herzfehler
von 6 bis 13 Jahre

Vorname: ______________________ Nachname: ___________________________

Alter: ________ Geschlecht: ________ Geschwister: Mädchen ___ Alter: ________
Junge _____ Alter: ________

Kindergartenjahre: Schulart: ________________ Klasse: Ausbildung: ____________

Schulabschluss Eltern: Mama ____________________ Papa ______________________

Berufsausbildung Eltern: Mama ____________________ Papa ______________________

Berufstätig: Mama ○ Papa ○ beide ○ keiner ○

Berufstätigkeit Eltern: Vollzeit Teilzeit Hausfrau/ -mann Andere: ________________
Mama ○ Mama ○ Mama ○
Papa ○ Papa ○ Papa ○

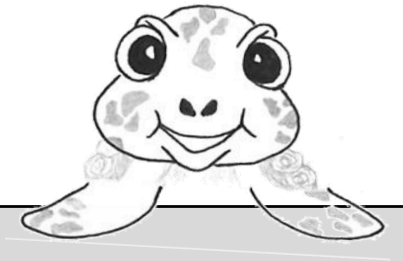

Hier oben steht immer ein Bereich um den es in den Fragen geht

Dann folgt die Frage:

Welche Aussage trifft auf Dich zu?

Dann machst Du Dein Kreuz wo Du es am Besten passend findest

	trifft voll zu	trifft zu	weiß nicht	trifft nicht zu	trifft gar nicht zu
Ich esse gerne Eis	X	○	○	○	○
Ich lese gerne	○	○	X	○	○

Vorsicht manche Fragen sind umgedreht, das erkennst Du an den Smileys:

Welche Aussage trifft auf Dich zu?

	trifft voll zu	trifft zu	weiß nicht	trifft nicht zu	trifft gar nicht zu
Ich gehe nicht gerne spazieren	○	X	○	○	○
Ich bin oft traurig	○	○	○	X	○

Copyright © 2025 by J. Remmele & P. C. Helm

In den letzten Wochen hat mich mein Herz in meiner Selbstständigkeit beeinflusst

Welche Aussage trifft auf Dich zu?	trifft gar nicht zu	trifft nicht zu	weiß nicht	trifft zu	trifft voll zu
Ich konnte an Aktivitäten mit Freunden teilnehmen.	○	○	○	○	○

In den letzten Wochen hat mich mein Herz in der Schule beeinflusst

Welche Aussage trifft auf Dich zu?	trifft voll zu	trifft zu	weiß nicht	trifft nicht zu	trifft gar nicht zu
Ich konnte im Sportunterricht nicht alles mitmachen.	○	○	○	○	○

Welche Aussage trifft auf Dich zu?	trifft gar nicht zu	trifft nicht zu	weiß nicht	trifft zu	trifft voll zu
Ich bin mit dem Unterrichtsstoff gut mitgekommen.	○	○	○	○	○

In den letzten Wochen hat mich mein Herz körperlich eingeschränkt

Welche Aussage trifft auf Dich zu?	trifft gar nicht zu	trifft nicht zu	weiß nicht	trifft zu	trifft voll zu
Ich fühlte mich körperlich frisch und munter.	○	○	○	○	○

Wie sieht es mit der Erholung von Dir und Deinem Herz in den letzten Wochen aus?

Welche Aussage trifft auf Dich zu?	trifft gar nicht zu	trifft nicht zu	weiß nicht	trifft zu	trifft voll zu
Ich hatte einen ruhigen festen Schlaf.	○	○	○	○	○

Du und Dein Herz allgemein

Welche Aussage trifft auf Dich zu?	trifft voll zu	trifft zu	weiß nicht	trifft nicht zu	trifft gar nicht zu
Ich finde Arztbesuche unangenehm.	○	○	○	○	○

Copyright © 2025 by J. Remmele & P. C. Helm

16. APPENDIX C: CHDSI Jugendliche (Langversion)

CHDSI

Congenital **H**eart **D**isease **S**pecific **I**nventory
Krankheitsspezifische Lebensqualität von Kindern und Jugendlichen mit angeborenem Herzfehler
von 14 bis 17 Jahre

Vorname: ____________________ Nachname: ________________________

Alter: ________ Geschlecht: ________ Geschwister: Mädchen ____ Alter: ________
Junge ______ Alter: ________

Kindergartenjahre: Schulart: ________________ Klasse: Ausbildung: ____________

Schulabschluss Eltern: Mama ____________________ Papa ______________________

Berufsausbildung Eltern: Mama ____________________ Papa ______________________

Berufstätig: Mama ◯ Papa ◯ beide ◯ keiner ◯

Berufstätigkeit Eltern: Vollzeit Teilzeit Hausfrau/-mann Andere: ________________
Mama ◯ Mama ◯ Mama ◯ ◯
Papa ◯ Papa ◯ Papa ◯ ◯

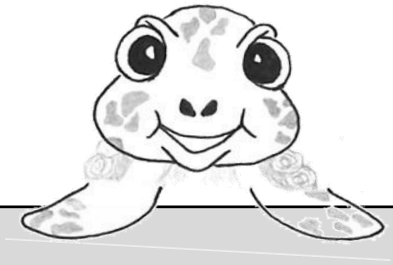

Hier oben steht immer ein Bereich um den es in den Fragen geht

<u>**Dann folgt die Frage:**</u> **Welche Aussage trifft auf Dich zu?**	trifft voll zu	trifft zu	weiß nicht	trifft nicht zu	trifft gar nicht zu
Ich esse gerne Eis	X	◯	◯	◯	◯
Ich lese gerne	◯	◯	X	◯	◯

Dann machst Du Dein Kreuz wo Du es am Besten passend findest

<u>**Vorsicht manche Fragen sind umgedreht, das erkennst Du an den Smileys:**</u> **Welche Aussage trifft auf Dich zu?**	trifft voll zu	trifft zu	weiß nicht	trifft nicht zu	trifft gar nicht zu
Ich gehe nicht gerne spazieren	◯	X	◯	◯	◯
Ich bin oft traurig	◯	◯	◯	X	◯

Copyright © 2025 by J. Remmele & P. C. Helm

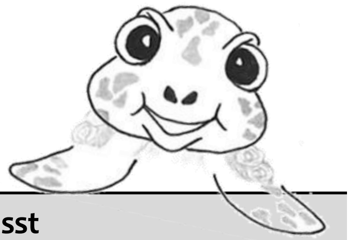

In den letzten Wochen hat mich mein Herz in meiner Selbstständigkeit beeinflusst

Welche Aussage trifft auf Dich zu?	trifft gar nicht zu	trifft nicht zu	weiß nicht	trifft zu	trifft voll zu
1. Ich fühlte mich genauso selbstständig wie meine Freunde.	○	○	○	○	○
2. Ich hatte keine Schwierigkeiten Freunde zu finden.	○	○	○	○	○
3 Ich konnte an Aktivitäten mit Freunden teilnehmen.	○	○	○	○	○
	trifft voll zu	trifft zu	weiß nicht	trifft nicht zu	trifft gar nicht zu
4. Ich habe mich gefragt ob ich mit meinem Herzfehler eine/n feste/n Freund/in finde.	○	○	○	○	○
5. Ich habe mich hilflos und traurig gefühlt.	○	○	○	○	○
6. Ich fühlte mich durch meinen Herzfehler in meiner Selbstständigkeit eingeschränkt.	○	○	○	○	○

Wegen meinem Herz haben sich in den letzten Wochen …

Welche Aussage trifft auf Dich zu?		trifft voll zu	trifft zu	weiß nicht	trifft nicht zu	trifft gar nicht zu
7. … meine Eltern viele Sorgen gemacht, das tut mir leid.		○	○	○	○	○
8. … in der Schule/Ausbildung Lehrer und Mitschüler mir gegenüber komisch verhalten.		○	○	○	○	○
9. … meine Eltern mir gegenüber anders verhalten als meinen Geschwistern gegenüber.	○ habe keine	○	○	○	○	○
10. … meine Eltern und andere Erwachsene übervorsichtig mir gegenüber verhalten.		○	○	○	○	○
11. … Lehrer und Mitschüler besonders um mich bemüht, ohne dass ich das wollte.		○	○	○	○	○
12. … Andere über mich lustig gemacht.		○	○	○	○	○

Copyright © 2025 by J. Remmele & P. C. Helm

In den letzten Wochen hat mich mein Herz in der Schule / Ausbildung beeinflusst

Welche Aussage trifft auf Dich zu?	trifft voll zu	trifft zu	weiß nicht	trifft nicht zu	trifft gar nicht zu
13. Ich habe viel Unterricht verpasst wegen Untersuchungen.	○	○	○	○	○
14. Ich war besorgt, welchen Beruf ich mit meinem Herzfehler machen kann.	○	○	○	○	○
15. Ich habe mir Gedanken gemacht, ob ich die Schule / Ausbildung schaffe.	○	○	○	○	○

	trifft gar nicht zu	trifft nicht zu	weiß nicht	trifft zu	trifft voll zu
16. Ich habe mir überhaupt keine Sorgen um meine Zukunft gemacht.	○	○	○	○	○
17. Ich bin mit den Hausaufgaben gut zurechtgekommen.	○	○	○	○	○
18. Ich bin mit dem Unterrichtsstoff gut mitgekommen.	○	○	○	○	○

In den letzten Wochen hat mich mein Herz körperlich eingeschränkt

Welche Aussage trifft auf Dich zu?	trifft gar nicht zu	trifft nicht zu	weiß nicht	trifft zu	trifft voll zu
19. Ich konnte alle körperlichen Aktivitäten machen wie ich wollte.	○	○	○	○	○
20. Ich konnte alles machen ohne außer Puste zu geraten.	○	○	○	○	○
21. Ich hatte keine körperlichen Schmerzen.	○	○	○	○	○
22. Ich habe mich selten krank gefühlt.	○	○	○	○	○
23. Ich konnte problemlos meine Hobbies machen.	○	○	○	○	○
24. Ich fühlte mich körperlich frisch und munter.	○	○	○	○	○

Copyright © 2025 by J. Remmele & P. C. Helm

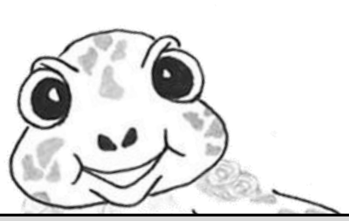

In den letzten Wochen hatte ich wegen meinem Herz …

**Welche Aussage trifft auf Dich zu?**	trifft voll zu	trifft zu	weiß nicht	trifft nicht zu	trifft gar nicht zu
25. … schnell keine Puste mehr.	○	○	○	○	○
26. … abends öfter dicke Beine und Füße.	○	○	○	○	○
27. … häufig mit Schwindel zu tun.	○	○	○	○	○
28. … auch Schmerzen in der Brust.	○	○	○	○	○
29. … gemerkt, dass ich schnell müde werde.	○	○	○	○	○
30. … öfter komisches Herzklopfen.	○	○	○	○	○

Wie sieht es mit der Erholung von Dir und Deinem Herz in den letzten Wochen aus?

**Welche Aussage trifft auf Dich zu?**	trifft voll zu	trifft zu	weiß nicht	trifft nicht zu	trifft gar nicht zu
31. Ich konnte nicht gut einschlafen.	○	○	○	○	○
32. Ich bin nachts häufig aufgewacht.	○	○	○	○	○
33. Ich bin morgens schwer aus dem Bett gekommen.	○	○	○	○	○

	trifft gar nicht zu	trifft nicht zu	weiß nicht	trifft zu	trifft voll zu
34. Ich bin erholt aufgewacht.	○	○	○	○	○
35. Ich hatte einen ruhigen festen Schlaf.	○	○	○	○	○
36. Ich habe mich gut nach anstrengenden Tagen erholt.	○	○	○	○	○

Copyright © 2025 by J. Remmele & P. C. Helm

Du und Dein Herz allgemein

Welche Aussage trifft auf Dich zu?		trifft gar nicht zu	trifft nicht zu	weiß nicht	trifft zu	trifft voll zu
37. Ich weiß gut Bescheid über mein Herz und was ihm fehlt.		○	○	○	○	○
38. Vor dem Krankenhaus habe ich keine Angst.		○	○	○	○	○
39. Ich vertrage meine Medikamente gut.	○ nehme keine	○	○	○	○	○

		trifft voll zu	trifft zu	weiß nicht	trifft nicht zu	trifft gar nicht zu
40. Ich finde Arztbesuche unangenehm.		○	○	○	○	○
41. Ich mag es nicht am Herzen untersucht zu werden.		○	○	○	○	○
42. Ich finde es nervig Medikamente zu nehmen.	○ nehme keine	○	○	○	○	○
43. Ich finde meine Operationsnarbe sehr unangenehm.	○ habe keine	○	○	○	○	○

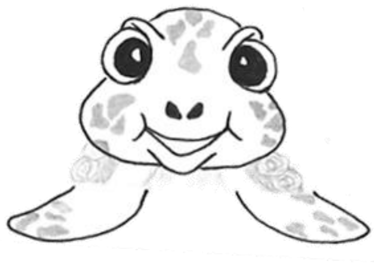

Gibt es etwas, das Du wichtig findest und hier noch nicht dabei war?

____

____

____

____

____

____

____

Vielen Dank, dass du die Fragen beantwortet hast!

Copyright © 2025 by J. Remmele & P. C. Helm

17. APPENDIX D: CHDSI Jugendliche (Kurzversion)

CHDSI Kurzversion

Congenital **H**eart **D**isease **S**pecific **I**nventory
Krankheitsspezifische Lebensqualität von Kindern und Jugendlichen mit angeborenem Herzfehler
von 14 bis 17 Jahre

Vorname: ______________________ Nachname: ___________________________

Alter: ________ Geschlecht: ________ Geschwister: Mädchen ___ Alter: _______
Junge _____ Alter: _______

Kindergartenjahre: Schulart: _______________ Klasse: Ausbildung: ____________

Schulabschluss Eltern: Mama ____________________ Papa ______________________

Berufsausbildung Eltern: Mama ___________________ Papa ______________________

Berufstätig: Mama ◯ Papa ◯ beide ◯ keiner ◯

Berufstätigkeit Eltern: Vollzeit Teilzeit Hausfrau/-mann Andere: ________________
Mama ◯ Mama ◯ Mama ◯
Papa ◯ Papa ◯ Papa ◯

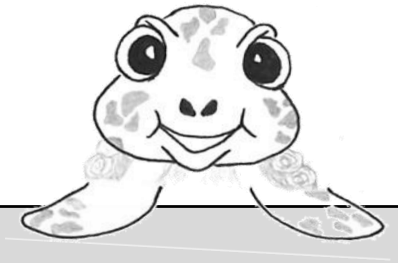

Hier oben steht immer ein Bereich um den es in den Fragen geht

**Dann folgt die Frage:** **Welche Aussage trifft auf Dich zu?** (Dann machst Du Dein Kreuz wo Du es am Besten passend findest)	trifft voll zu	trifft zu	weiß nicht	trifft nicht zu	trifft gar nicht zu
Ich esse gerne Eis	X	◯	◯	◯	◯
Ich lese gerne	◯	◯	X	◯	◯

**Vorsicht manche Fragen sind umgedreht, das erkennst Du an den Smileys:** **Welche Aussage trifft auf Dich zu?**	trifft voll zu	trifft zu	weiß nicht	trifft nicht zu	trifft gar nicht zu
Ich gehe nicht gerne spazieren	◯	X	◯	◯	◯
Ich bin oft traurig	◯	◯	◯	X	◯

Copyright © 2025 by Julia Remmele

In den letzten Wochen hat mich mein Herz in meiner Selbstständigkeit beeinflusst

**Welche Aussage trifft auf Dich zu?**	trifft gar nicht zu	trifft nicht zu	weiß nicht	trifft zu	trifft voll zu
Ich konnte an Aktivitäten mit Freunden teilnehmen.	○	○	○	○	○

**Welche Aussage trifft auf Dich zu?**	trifft voll zu	trifft zu	weiß nicht	trifft nicht zu	trifft gar nicht zu
Ich fühlte mich durch meinen Herzfehler in meiner Selbstständigkeit eingeschränkt.	○	○	○	○	○

In den letzten Wochen hat mich mein Herz in der Schule / Ausbildung beeinflusst

**Welche Aussage trifft auf Dich zu?**	trifft gar nicht zu	trifft nicht zu	weiß nicht	trifft zu	trifft voll zu
Ich bin mit dem Unterrichtsstoff gut mitgekommen.	○	○	○	○	○

In den letzten Wochen hat mich mein Herz körperlich eingeschränkt

**Welche Aussage trifft auf Dich zu?**	trifft gar nicht zu	trifft nicht zu	weiß nicht	trifft zu	trifft voll zu
Ich fühlte mich körperlich frisch und munter.	○	○	○	○	○

Wie sieht es mit der Erholung von Dir und Deinem Herz in den letzten Wochen aus?

**Welche Aussage trifft auf Dich zu?**	trifft gar nicht zu	trifft nicht zu	weiß nicht	trifft zu	trifft voll zu
Ich habe mich gut nach anstrengenden Tagen erholt.	○	○	○	○	○

Du und Dein Herz allgemein

**Welche Aussage trifft auf Dich zu?**	trifft voll zu	trifft zu	weiß nicht	trifft nicht zu	trifft gar nicht zu
Ich finde Arztbesuche unangenehm.	○	○	○	○	○

Copyright © 2025 by J. Remmele & P. C. Helm
