# Supplementary material for: Development and Validation of the CHDSI Questionnaire: A New Tool for Measuring Disease-Specific Quality of Life in Children and Adolescents with Congenital Heart Defects
Source: Medicina (Kaunas). 2025 Jul 21;61(7):1311. doi: 10.3390/medicina61071311 (PMC12297989; doi:10.3390/medicina61071311)
Supplement: Supplementary file 1 [file medicina-61-01311-s001.zip › 8. Supplemental Material 1a - CHDSI Questionaire (Long - Children) in German.pdf]

CHDSI

Congenital **H**eart **D**isease **S**pecific **I**nventory
Krankheitsspezifische Lebensqualität von Kindern und Jugendlichen mit angeborenem Herzfehler
von 6 bis 13 Jahre

Vorname: ______________ Nachname: ______________

Alter: ______ Geschlecht: ______ Geschwister: Mädchen ___ Alter: ______
Junge ___ Alter: ______

Kindergartenjahre: ___ Schulart: ______________ Klasse: ___ Ausbildung: ______________

Schulabschluss Eltern: Mama ______________ Papa ______________

Berufsausbildung Eltern: Mama ______________ Papa ______________

Berufstätig: Mama ○ Papa ○ beide ○ keiner ○

Berufstätigkeit Eltern:	Vollzeit	Teilzeit	Hausfrau/-mann	Andere: ______________
	Mama ○	Mama ○	Mama ○	
	Papa ○	Papa ○	Papa ○	

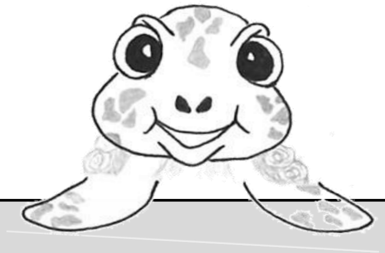

Hier oben steht immer ein Bereich um den es in den Fragen geht

<u>**Dann folgt die Frage:**</u> **Welche Aussage trifft auf Dich zu?**	trifft voll zu	trifft zu	weiß nicht	trifft nicht zu	trifft gar nicht zu
Ich esse gerne Eis	X	○	○	○	○
Ich lese gerne	○	○	X	○	○

Dann machst Du Dein Kreuz wo Du es am Besten passend findest

<u>**Vorsicht manche Fragen sind umgedreht, das erkennst Du an den Smileys:**</u> **Welche Aussage trifft auf Dich zu?**	trifft voll zu	trifft zu	weiß nicht	trifft nicht zu	trifft gar nicht zu
Ich gehe nicht gerne spazieren	○	X	○	○	○
Ich bin oft traurig	○	○	○	X	○

Copyright © 2025 by J. Remmele & P. C. Helm

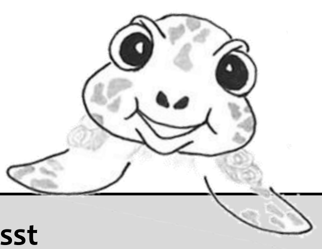

In den letzten Wochen hat mich mein Herz in meiner Selbstständigkeit beeinflusst

**Welche Aussage trifft auf Dich zu?**	trifft gar nicht zu	trifft nicht zu	weiß nicht	trifft zu	trifft voll zu
1. Ich fühlte mich im Vergleich mit Freunden gleich gut.	○	○	○	○	○
2. Ich habe mich wohl gefühlt wie ich bin.	○	○	○	○	○
3. Ich konnte an Aktivitäten mit Freunden teilnehmen.	○	○	○	○	○

	trifft voll zu	trifft zu	weiß nicht	trifft nicht zu	trifft gar nicht zu
4. Ich habe mir Sorgen über meine Zukunft gemacht.	○	○	○	○	○
5. Ich habe mich hilflos und traurig gefühlt.	○	○	○	○	○
6. Ich hatte Schwierigkeiten Freunde zu finden.	○	○	○	○	○

Wegen meinem Herz haben sich in den letzten Wochen …

Welche Aussage trifft auf Dich zu?
Wenn Du noch nicht in die Schule gehst, beziehen sich die Aussagen zur Schule natürlich auf die Erzieher und die anderen Kindergartenkinder.

	trifft voll zu	trifft zu	weiß nicht	trifft nicht zu	trifft gar nicht zu
7. … meine Eltern viele Sorgen gemacht, das tut mir leid.	○	○	○	○	○
8. … in der Schule Lehrer und Mitschüler mir gegenüber komisch verhalten.	○	○	○	○	○
9. … meine Eltern mir gegenüber anders verhalten als meinen Geschwistern gegenüber. ○ habe keine	○	○	○	○	○
10. … meine Eltern und andere Erwachsene übervorsichtig mir gegenüber verhalten.	○	○	○	○	○
11. … Lehrer und Mitschüler besonders um mich bemüht, ohne dass ich das wollte.	○	○	○	○	○
12. … andere Kinder über mich lustig gemacht.	○	○	○	○	○

Copyright © 2025 by J. Remmele & P. C. Helm

In den letzten Wochen hat mich mein Herz in der Schule beeinflusst

Ich gehe noch nicht in die Schule ○ , dann brauchst Du diesen Block nicht ausfüllen!

**Welche Aussage trifft auf Dich zu?**	trifft voll zu	trifft zu	weiß nicht	trifft nicht zu	trifft gar nicht zu
13. Ich habe viel Unterricht verpasst wegen Untersuchungen.	○	○	○	○	○
14. Ich konnte im Sportunterricht nicht alles mitmachen.	○	○	○	○	○
15. Ich habe mir Gedanken gemacht, ob ich die Schule schaffe.	○	○	○	○	○

	trifft gar nicht zu	trifft nicht zu	weiß nicht	trifft zu	trifft voll zu
16. Ich bin mit dem Unterrichtsstoff gut mitgekommen.	○	○	○	○	○
17. Ich bin mit den Hausaufgaben gut zurechtgekommen.	○	○	○	○	○
18. Ich gehe grundsätzlich gerne zur Schule.	○	○	○	○	○

In den letzten Wochen hat mich mein Herz körperlich eingeschränkt

**Welche Aussage trifft auf Dich zu?**	trifft gar nicht zu	trifft nicht zu	weiß nicht	trifft zu	trifft voll zu
19. Ich konnte alle körperlichen Aktivitäten machen wie ich wollte.	○	○	○	○	○
20. Ich konnte alles machen ohne außer Puste zu geraten.	○	○	○	○	○
21. Ich hatte keine körperlichen Schmerzen.	○	○	○	○	○
22. Ich habe mich selten krank gefühlt.	○	○	○	○	○
23. Ich konnte problemlos meine Hobbies machen.	○	○	○	○	○
24. Ich fühlte mich körperlich frisch und munter.	○	○	○	○	○

Copyright © 2025 by J. Remmele & P. C. Helm

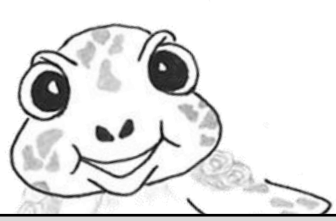

In den letzten Wochen hatte ich wegen meinem Herz …

Welche Aussage trifft auf Dich zu?	trifft voll zu	trifft zu	weiß nicht	trifft nicht zu	trifft gar nicht zu
25. … schnell keine Puste mehr.	○	○	○	○	○
26. … abends öfter dicke Beine und Füße.	○	○	○	○	○
27. … häufig mit Schwindel zu tun.	○	○	○	○	○
28. … auch Schmerzen in der Brust.	○	○	○	○	○
29. … gemerkt, dass ich schnell müde werde.	○	○	○	○	○
30. … öfter komisches Herzklopfen.	○	○	○	○	○

Wie sieht es mit der Erholung von Dir und Deinem Herz in den letzten Wochen aus?

Welche Aussage trifft auf Dich zu?	trifft voll zu	trifft zu	weiß nicht	trifft nicht zu	trifft gar nicht zu
31. Ich konnte nicht gut einschlafen.	○	○	○	○	○
32. Ich bin nachts häufig aufgewacht.	○	○	○	○	○
33. Ich bin morgens schwer aus dem Bett gekommen.	○	○	○	○	○

	trifft gar nicht zu	trifft nicht zu	weiß nicht	trifft zu	trifft voll zu
34. Ich bin erholt aufgewacht.	○	○	○	○	○
35. Ich hatte einen ruhigen festen Schlaf.	○	○	○	○	○
36. Ich habe mich gut nach anstrengenden Tagen erholt.	○	○	○	○	○

Copyright © 2025 by J. Remmele & P. C. Helm

Du und Dein Herz allgemein

**Welche Aussage trifft auf Dich zu?**		trifft gar nicht zu	trifft nicht zu	weiß nicht	trifft zu	trifft voll zu
37. Ich weiß gut Bescheid über mein Herz und was ihm fehlt.		○	○	○	○	○
38. Vor dem Krankenhaus habe ich keine Angst.		○	○	○	○	○
39. Ich vertrage meine Medikamente gut.	○ nehme keine	○	○	○	○	○

		trifft voll zu	trifft zu	weiß nicht	trifft nicht zu	trifft gar nicht zu
40. Ich finde Arztbesuche unangenehm.		○	○	○	○	○
41. Ich mag es nicht am Herzen untersucht zu werden.		○	○	○	○	○
42. Ich finde es nervig Medikamente zu nehmen.	○ nehme keine	○	○	○	○	○
43. Ich finde meine Operationsnarbe sehr unangenehm.	○ habe keine	○	○	○	○	○

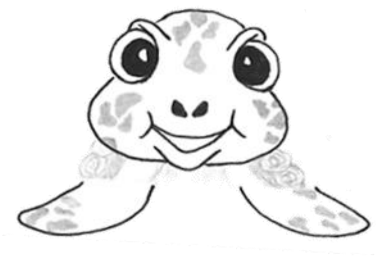

Gibt es etwas, das Du wichtig findest und hier noch nicht dabei war?

____
____
____
____
____
____
____

Vielen Dank, dass du die Fragen beantwortet hast!

Copyright © 2025 by J. Remmele & P. C. Helm
