# Supplementary material for: Development and Validation of the CHDSI Questionnaire: A New Tool for Measuring Disease-Specific Quality of Life in Children and Adolescents with Congenital Heart Defects
Source: Medicina (Kaunas). 2025 Jul 21;61(7):1311. doi: 10.3390/medicina61071311 (PMC12297989; doi:10.3390/medicina61071311)
Supplement: Supplementary file 1 [file medicina-61-01311-s001.zip › 9. Supplemental Material 1b - CHDSI-SF Questionaire (Short - Children) in German.pdf]

CHDSI Kurzversion

Congenital **H**eart **D**isease **S**pecific **I**nventory
Krankheitsspezifische Lebensqualität von Kindern und Jugendlichen mit angeborenem Herzfehler
von 6 bis 13 Jahre

Vorname: ______________ Nachname: ______________

Alter: ______ Geschlecht: ______ Geschwister: Mädchen ___ Alter: ______
Junge ___ Alter: ______

Kindergartenjahre: ___ Schulart: ______________ Klasse: ___ Ausbildung: ______________

Schulabschluss Eltern: Mama ______________ Papa ______________

Berufsausbildung Eltern: Mama ______________ Papa ______________

Berufstätig: Mama ○ Papa ○ beide ○ keiner ○

Berufstätigkeit Eltern:	Vollzeit	Teilzeit	Hausfrau/-mann	Andere: ______________
	Mama ○	Mama ○	Mama ○	
	Papa ○	Papa ○	Papa ○	

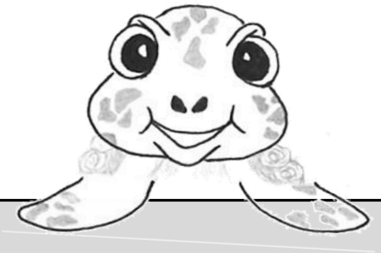

Hier oben steht immer ein Bereich um den es in den Fragen geht

<u>Dann folgt die Frage:</u>

Dann machst Du Dein Kreuz wo Du es am Besten passend findest

**Welche Aussage trifft auf Dich zu?**	**trifft voll zu**	**trifft zu**	**weiß nicht**	**trifft nicht zu**	**trifft gar nicht zu**
Ich esse gerne Eis	X	○	○	○	○
Ich lese gerne	○	○	X	○	○

<u>Vorsicht manche Fragen sind umgedreht, das erkennst Du an den Smileys:</u>

**Welche Aussage trifft auf Dich zu?**	**trifft voll zu**	**trifft zu**	**weiß nicht**	**trifft nicht zu**	**trifft gar nicht zu**
Ich gehe nicht gerne spazieren	○	X	○	○	○
Ich bin oft traurig	○	○	○	X	○

Copyright © 2025 by J. Remmele & P. C. Helm

In den letzten Wochen hat mich mein Herz in meiner Selbstständigkeit beeinflusst

Welche Aussage trifft auf Dich zu?	trifft gar nicht zu	trifft nicht zu	weiß nicht	trifft zu	trifft voll zu
Ich konnte an Aktivitäten mit Freunden teilnehmen.	○	○	○	○	○

In den letzten Wochen hat mich mein Herz in der Schule beeinflusst

Welche Aussage trifft auf Dich zu?	trifft voll zu	trifft zu	weiß nicht	trifft nicht zu	trifft gar nicht zu
Ich konnte im Sportunterricht nicht alles mitmachen.	○	○	○	○	○

Welche Aussage trifft auf Dich zu?	trifft gar nicht zu	trifft nicht zu	weiß nicht	trifft zu	trifft voll zu
Ich bin mit dem Unterrichtsstoff gut mitgekommen.	○	○	○	○	○

In den letzten Wochen hat mich mein Herz körperlich eingeschränkt

Welche Aussage trifft auf Dich zu?	trifft gar nicht zu	trifft nicht zu	weiß nicht	trifft zu	trifft voll zu
Ich fühlte mich körperlich frisch und munter.	○	○	○	○	○

Wie sieht es mit der Erholung von Dir und Deinem Herz in den letzten Wochen aus?

Welche Aussage trifft auf Dich zu?	trifft gar nicht zu	trifft nicht zu	weiß nicht	trifft zu	trifft voll zu
Ich hatte einen ruhigen festen Schlaf.	○	○	○	○	○

Du und Dein Herz allgemein

Welche Aussage trifft auf Dich zu?	trifft voll zu	trifft zu	weiß nicht	trifft nicht zu	trifft gar nicht zu
Ich finde Arztbesuche unangenehm.	○	○	○	○	○

Copyright © 2025 by J. Remmele & P. C. Helm
